# A prediction of mutations in infectious viruses using artificial intelligence

**DOI:** 10.1186/s44342-024-00019-y

**Published:** 2024-10-08

**Authors:** Won Jong Choi, Jongkeun Park, Do Young Seong, Dae Sun Chung, Dongwan Hong

**Affiliations:** 1 Department of Precision Medicine and Big Data, College of Medicine, The Catholic University of Korea, Seoul, 06591 Republic of Korea; 2 Department of Medical Informatics, The Catholic University of Korea, Seoul, 06591 Republic of Korea; 3 Department of Medical Sciences, Graduate Schoolof, College of Medicine , The Catholic University of Korea, Seoul, 06591 Republic of Korea; 4 Precision Medicine Research Center, College of Medicine, The Catholic University of Korea, Seoul, 06591 Republic of Korea; 5 Cancer Evolution Research Center, College of Medicine, The Catholic University of Korea, Seoul, 06591 Republic of Korea; 6 College of Medicine, CMC Institute for Basic Medical Science, The Catholic University of Korea, Seoul, 06591 Republic of Korea

**Keywords:** Machine learning, Deep learning, SARS-CoV-2, Clade, Mutation, Prediction

## Abstract

Many subtypes of SARS-CoV-2 have emerged since its early stages, with mutations showing regional and racial differences. These mutations significantly affected the infectivity and severity of the virus. This study aimed to predict the mutations that occur during the evolution of SARS-CoV-2 and identify the key characteristics for making these predictions. We collected and organized data on the lineage, date, clade, and mutations of SARS-CoV-2 from publicly available databases and processed them to predict the mutations. In addition, we utilized various artificial intelligence models to predict newly emerging mutations and created various training sets based on clade information. Using only mutation information resulted in low performance of the learning models, whereas incorporating clade differentiation resulted in high performance in machine learning models, including XGBoost (accuracy: 0.999). However, mutations fixed in the receptor-binding motif (RBM) region of Omicron resulted in decreased predictive performance. Using these models, we predicted potential mutation positions for 24C, following the recently emerged 24A and 24B clades. We identified a mutation at position Q493 in the RBM region. Our study developed effective artificial intelligence models and characteristics for predicting new mutations in continuously evolving infectious viruses.

**Supplementary Information:**

The online version contains supplementary material available at 10.1186/s44342-024-00019-y.

## Background

Coronavirus disease 2019 (COVID-19) has become prevalent worldwide since 2019. Although its infectivity has decreased recently, it still occurs frequently (https://ourworldindata.org/). The World Health Organization (WHO) distinguishes pathological differences by lineage based on combinations of mutation types, categorizing SARS-CoV-2 into variants of concern (VOCs), variants of interest (VOIs), and variants being monitored (VBMs). Through its continuous evolution, severe acute respiratory syndrome coronavirus 2 (SARS-CoV-2) has continuously produced mutations, resulting in 40 clades. These mutations are associated with disease severity and transmission to humans [1, 2].

Mutations that have occurred during the evolution of SARS-CoV-2 have mainly been observed in the receptor-binding domain (RBD) of the spike protein. These mutations facilitate immune evasion, bind to the host cell’s ACE2 receptor, and are key targets for vaccines and treatments, necessitating close monitoring [3–5].

A substantial amount of epidemiological, genetic, and vaccine-related data has been accumulated for COVID-19. Numerous studies have been conducted to utilize these data effectively for diagnosis, prevention, and treatment. Mathematical and statistical models have been used to quantify the virulence and transmissibility of SARS-CoV-2 and to predict the spread of the Omicron variant [6, 7]. Artificial intelligence is also used to build literature-based learning models for diagnosis and prognosis related to SARS-CoV-2 or to utilize clinical markers and clinical information for severity prediction or diagnosis of COVID-19 [8–10]. However, many of these studies exhibit regional and racial biases in the data, which can lead to challenges in generalization and increase the risk of overfitting. Furthermore, the frequent mutations in SARS-CoV-2 make it substantially more difficult to develop treatments or vaccines compared to previous infectious diseases, making it necessary to predict these mutations.

Obermeyer et al. used machine learning to identify mutations occurring in different structures of SARS-CoV-2 and predict new lineages [11]. Additionally, they employed a phylogenetic tree-based sampling method that integrated temporal and sequence information to predict mutations [12]. Ultimately, accurately predicting the exact mutations that branch from a phylogenetic tree is challenging. Studies on mutation prediction using deep learning have also declined (Supplementary Fig. 1). Moreover, using mutation data from the early Omicron variant for training resulted in a lower accuracy in predicting recent mutations.

To enhance the accuracy of mutation prediction, it is crucial to carefully select the features used in the analysis and the correlations between mutations. We aimed to provide precise mutation information by leveraging key data, including mutation details, time-series information, and insights into the phylogenetic relationships between lineages for prediction (Supplementary Fig. 2).

## Methods

### Data collection

We utilized the spike glycoprotein (UniProt ID: P0DTC2, SPIKE_SARS2) from severe acute respiratory syndrome coronavirus 2 (SARS-CoV-2), as referenced in the UniProt database. Specifically, the amino acid sequence between the N-terminal region (NSNNLDSKVGGNYNYLYRLFRKSNLKPFERDISTEIYQAGSTPCN) and the C-terminal region (GVEGFNCYFPLQSYGFQPTNGVGYQPY) was used for our study.

### Preprocessing for learning

We retrieved metadata from Nextstrain’s nCoV open data page (https://data.nextstrain.org/files/ncov/open/metadata.tsv.zst). The data includes the host and collection date of virus samples, the collection region, the gender and age of the sample, lineage, and mutation information. We filtered and processed the data using the host, collection date, lineage, and mutation information to create the training data.

**Fig. 1:**
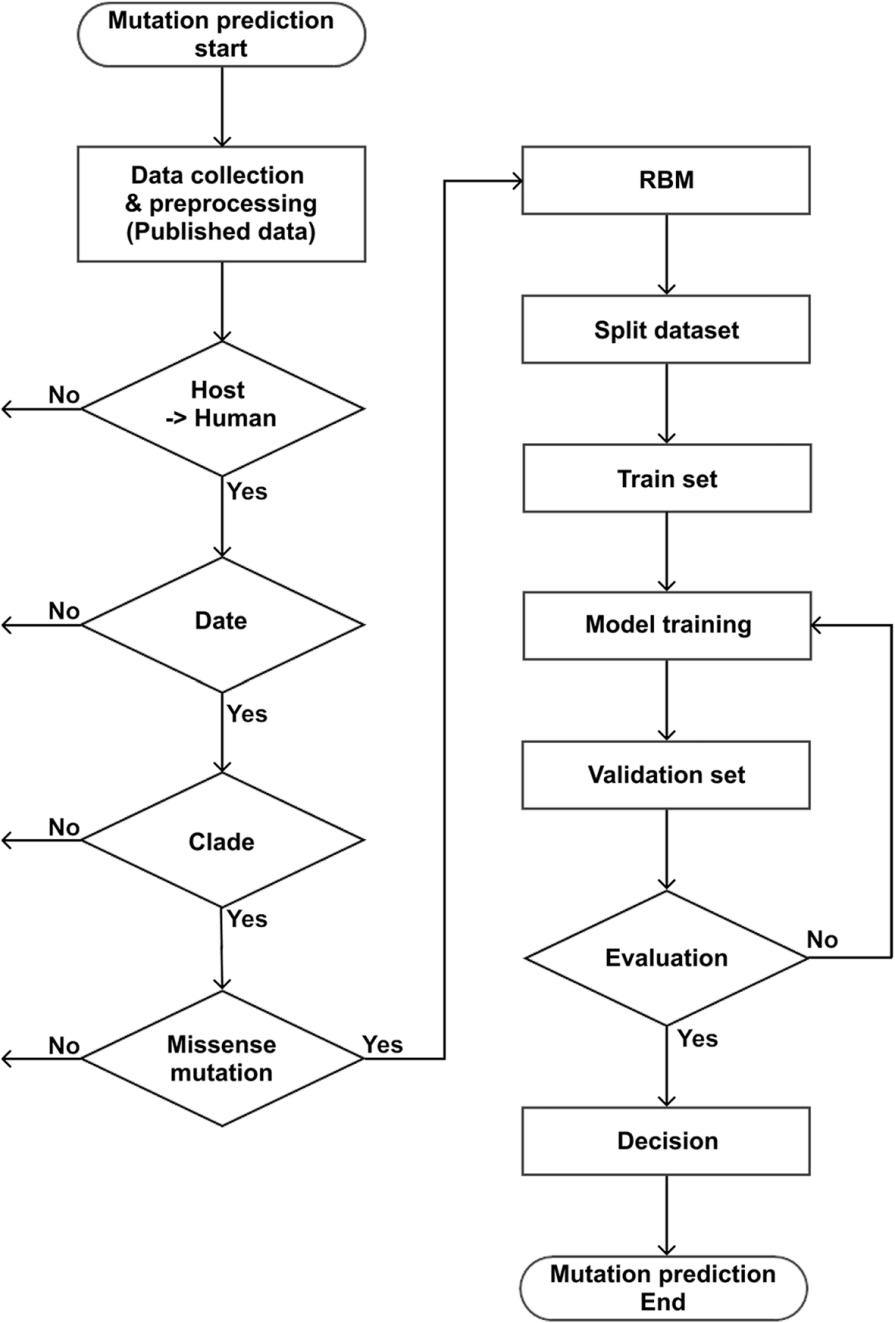
Flowchart for accurate prediction of the next mutation in the evolution of infectious diseases (COVID-19)

Before the learning process, we filtered and standardized the data as follows. We formatted the dates in the YYYY-MM-DD format. We specified the host as “human.” For the Pango lineage and Nextstrain clade, we removed Not a Number (NaN) and “?” values. For amino acid substitutions, we filtered and only used mutations found in the RBM (437–508) region. Through the filtering process, we extracted 8,411,025 samples from a total of 8,586,162 samples (Fig. 1).

Secondly, we performed the following steps for data preprocessing. We converted the date information to the number of days elapsed since the initial collection date and then normalized it to a range of 0–1 using a min–max scaler [13, 14]. We used the mutation information from the RBM region of the spike protein (P0DTC2) to create a 72-position mutation sequence. We connected the parent clade and corresponding subclades involved in the clade diversification to create datasets (data available at https://github.com/Honglab-Research/Covid-mutation-probability).

### Data processing

We used various models for the learning process, including machine learning models (LightGBM [15], XGBoost [16], and random forest [17]) and a deep learning model (gated recurrent unit) (Fig. 1) (https://github.com/Honglab-Research/Covid-mutation-probability).

We performed the training set process as follows, considering the clade’s timeline, mutations, and clade branching points. In the mutation prediction process, we created training datasets from three perspectives, considering temporal information and the differentiation process of SARS-CoV-2.

First, we conducted training using only mutation information, without considering any additional information such as time and differentiation data. We used a random state to randomly generate the training set, validation set, and test set from the entire dataset.

Second, we created the training set and test set with temporal information. We investigated the outbreak periods (waves) of SARS-CoV-2 to generate the datasets: wave 1 (March 2020-June 2020), wave 2 (September 2020-January 2021), wave 3 with the Alpha variant (January 2021-June 2021), wave 4 with the Delta variant (July 2021-October 2021), and wave 5 with the onset of the Omicron variant (from November 2021). Based on these periods, we organized three datasets. The first training set used wave 1 as the training set and wave 2 as the test set, training to predict wave 3. The second training set used wave 1 and wave 2 as the training set and wave 3 as the test set, training to predict wave 4. The third training set used wave 1 as the training set and wave 2 as the test set, training to predict wave 4.

**Fig. 2:**
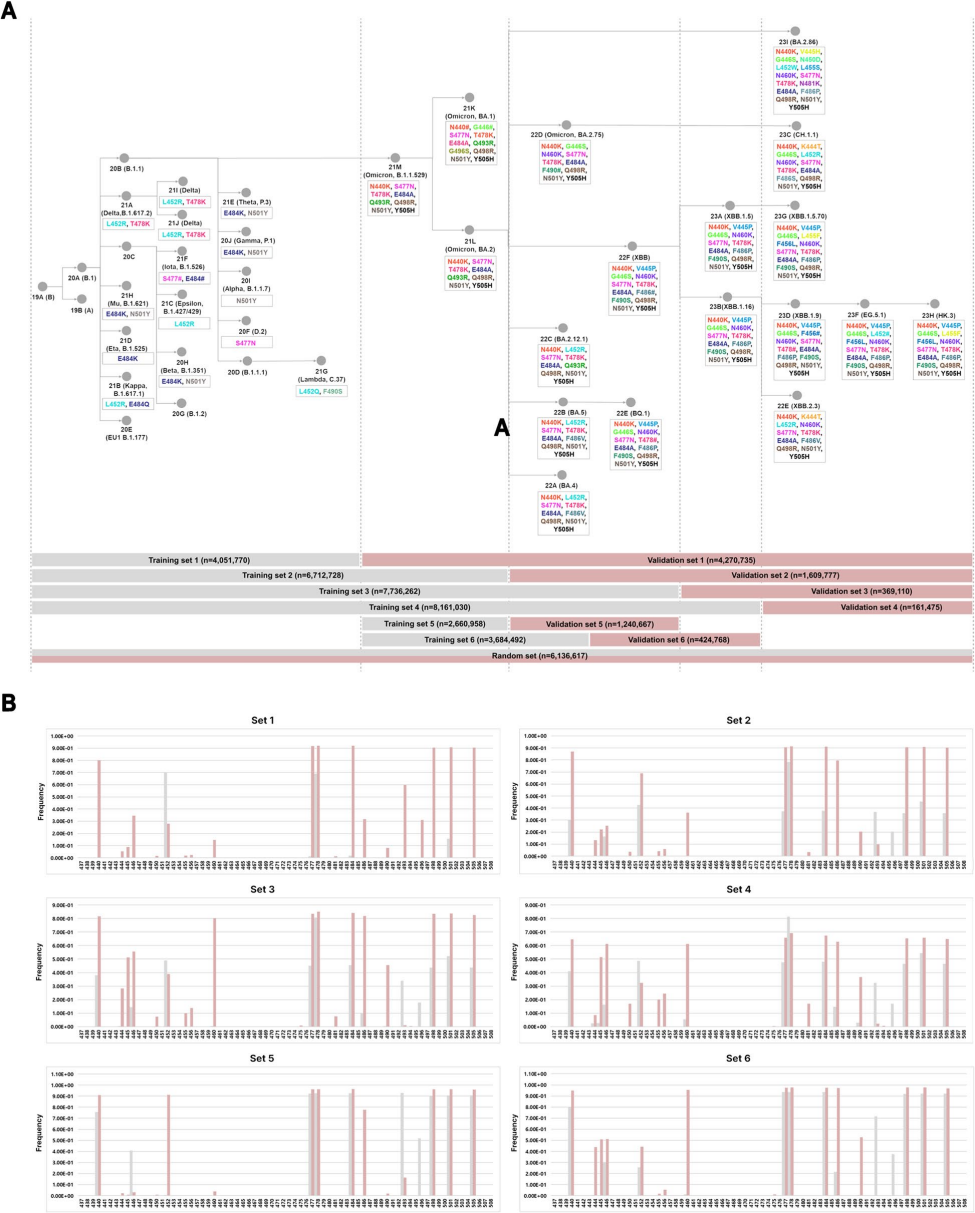
Phylogenetic tree based on clades, mutation information, and model training data composition. **A** Nextstrain data collected from December 23, 2019, to February 27, 2024, is visualized by clade, WHO-designated name, and Pango nomenclature lineage. Mutation information found with high frequency (80%) at 72 positions within the receptor binding motif (RBM) of the spike protein is also displayed. Training sets and validation sets are constructed for each clade, forming six stages of training sets. **B** For the training set and validation set, the mutation rates at each RBM position are displayed

Third, we created training datasets based on the clade differentiation process. The first training set utilized clades prior to 21 M (Omicron B.1.1.529), including 19A, 19B, 20A, 20B, 20C, 20D, 20E (B.1.177), 20F (D.2), 20G, 20H, 20I, 20 J, 21A (Delta, B.1.617.2), 21B (Kappa, B.1.617.1), 21C (Epsilon, B.1.427, B.1.429), 21D (Eta, B.1.525), 21E (Theta P.3), 21F (Iota, B.1.526), 21G (Lambda, C.37), 21H (Mu, B.1.621), 21I (Delta), and 21 J (delta). The validation set was trained using clades after 21 M (Omicron B.1.1.529). The second training set was created by adding clades 21 K (Omicron BA.1) and 21L (Omicron BA.2) to the first training set data. The validation set was trained using clades 22A (Omicron BA.4), 22B (Omicron BA.5), 22C (Omicron BA.2.12.1), 22D (Omicron BA.2.75), 22E (Omicron BQ.1), 22F (Omicron XBB), 23A (Omicron XBB.1.15), 23B (Omicron XBB.1.16), 23C (Omicron CH.1.1), 23D (Omicron XBB.1.9), 23E (Omicron XBB.2.3), 23F (Omicron EG.5.1), 23G (Omicron XBB.1.5.70), 23H (Omicron HK.3), and 23I (Omicron BA.2.86). The third training set was created by adding clades 22A (Omicron BA.4), 22B (Omicron BA.5), 22C (Omicron BA.2.12.1), and 22D (Omicron BA.2.75) to the second training set data. The validation set was trained using clades 22E (Omicron BQ.1), 22F (Omicron XBB), 23A (Omicron XBB.1.15), 23B (Omicron XBB.1.16), 23C (Omicron CH.1.1), 23D (Omicron XBB.1.9), 23E (Omicron XBB.2.3), 23F (Omicron EG.5.1), 23G (Omicron XBB.1.5.70), 23H (Omicron HK.3), and 23I (Omicron BA.2.86). The fourth training set was created by adding clades 22E (Omicron BQ.1), 22F (Omicron XBB), 23A (Omicron XBB.1.15), and 23B (Omicron XBB.1.16) to the third training set data. The validation set was trained using clades 23C (Omicron CH.1.1), 23D (Omicron XBB.1.9), 23E (Omicron XBB.2.3), 23F (Omicron EG.5.1), 23G (Omicron XBB.1.5.70), 23H (Omicron HK.3), and 23I (Omicron BA.2.86). Fifth, we used early Omicron variants 21 M, 21 K, and 21L for the training dataset and then used subsequent variants 22A, 22B, 22C, and 22D for the validation dataset. Sixth, we used 21 M, 21 K, 21L, 22A, 22B, 22C, and 22D as the training dataset and 22E, 22F, 23A, and 23B as the test dataset for training (Fig. 2A, Supplementary Table 2) (https://github.com/Honglab-Research/Covid-mutation-probability).

### Data analysis

The criteria for predicting the next mutation through the learning model were defined based on the lineage pipeline rules from Pangolin. A lineage-defining mutation is considered to occur when it appears in the first 80% of records for that lineage as logged by Pangolin. This study also defines new mutations according to this criterion (https://cov-lineages.org/resources/pangolin.html and https://ncbiinsights.ncbi.nlm.nih.gov/2024/05/02/automated-lineage-definitions-ncbi-virus-sars-cov-2-variants-overview/). Each model receives information about the presence or absence of mutations at position 72 in the RBM region as input. The model then predicts and outputs positions where future mutations are likely to occur. The performance of the model is evaluated by comparing predicted mutation positions with the actual mutation positions in samples from the evolution of SARS-CoV-2. Therefore, based on this criterion, mutation predictions were performed using the learning model. To evaluate efficiency, accuracy, precision, recall, and F-score were utilized [18].

## Results

### Data investigation for SARS-CoV-2 mutation prediction

We investigated the clades and lineages of SARS-CoV-2 from its outbreak to the present, organizing the data necessary for training based on the mutation frequency [19]. We focused on the RBM of the spike protein, which showed the highest mutation frequency (Supplementary Fig. 3).

**Fig. 3:**
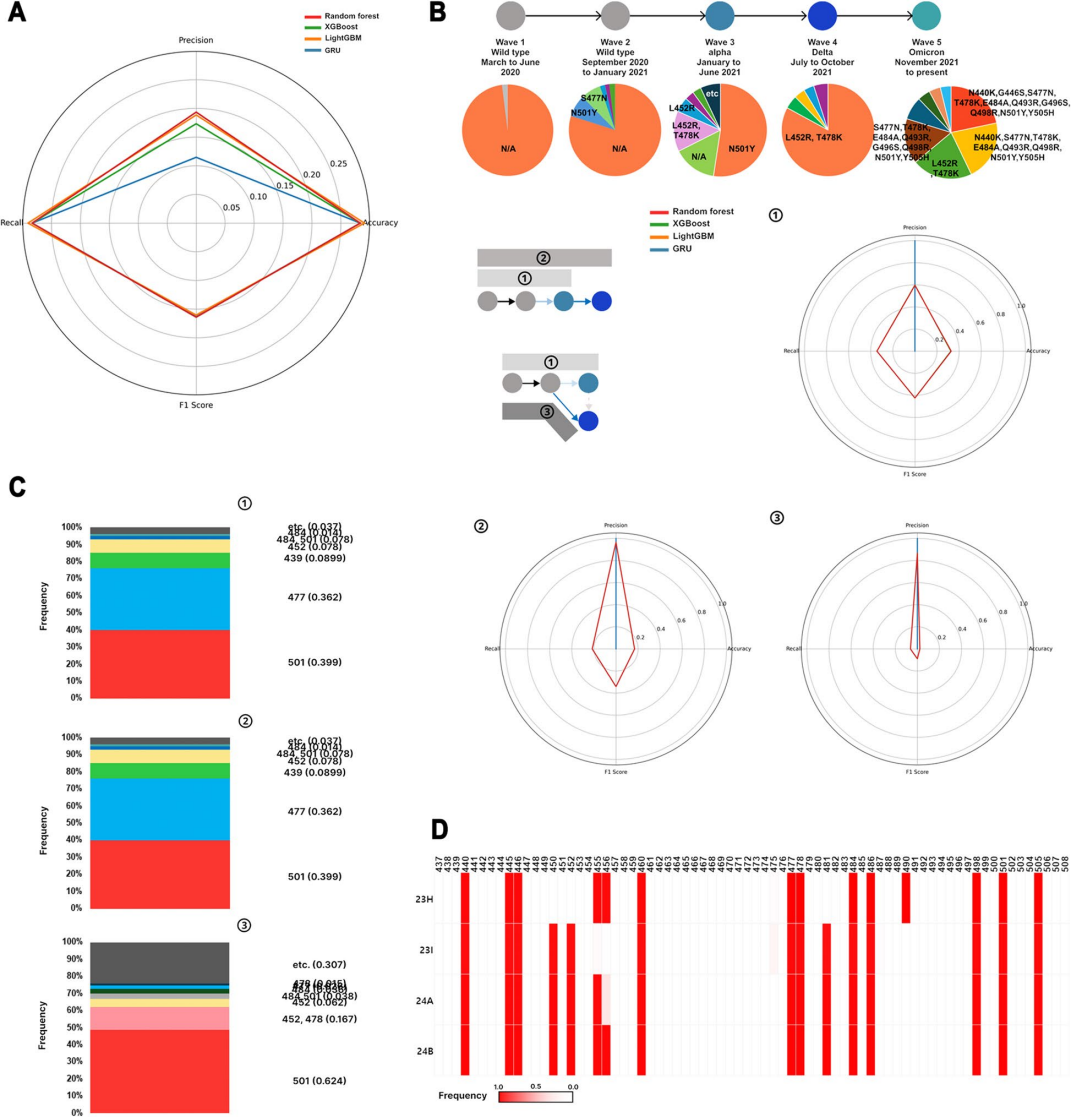
Data composition for accurate mutation prediction in the RBM region of SARS-CoV-2. **A** Model performance when trained with a dataset created using random state from clades collected from December 23, 2019, to May 22, 2023. **B** Pre-training of accurate mutations using the pandemic wave periods, mutations found in the RBM, and the regional information of the collected samples. Models trained to predict wave 3 from wild-type waves 1 and 2; wave 4 from wild-type waves 1, 2, and 3; and wave 4 (delta) from wild-type waves 1 and 2. **C** Frequency of mutation locations that may occur in the next wave predicted by the XGBoost model (waves 1, 2 −  > wave 3: ①, waves 1, 2, 3 −  > wave 4: ②, waves 1, 2 −  > wave 4: ③). **D** For current Omicron variants including wave 5, mutations in specific RBM regions become fixed. Red indicates higher frequency of mutations at that position, while white indicates lower frequency (23H: *n* = 5830, 23I: *n* = 7452, 24A: *n* = 86,318, 24B: *n* = 9922)

The datasets required for training were structured as training sets that included the entirety of the SARS-CoV-2 clades (training sets 1, 2, 3, and 4) and training sets constructed using omicron clades with the highest number of mutations (training sets 5 and 6). Finally, datasets were created using a random state method for all the clades that emerged (Fig. 3A). In the training sets, the mutation occurrence positions were weighted towards specific locations within each set, showing a high frequency only at those positions. Therefore, refined data suitable for training were required (Fig. 2B).

### Data construction for accurate predictions of SARS-CoV-2 mutations

Using clades from 2019 to May 2023, we aimed to predict SARS-CoV-2 mutations using machine learning (random forest, XGBoost, and LightGBM) and deep learning (GRU) models. Randomly extracted clades were used to predict potential mutations. The results showed low accuracy in both machine learning models (random forest, XGBoost, and LightGBM) and the deep learning model (GRU) (Fig. 3A). For accurate mutation prediction, the time and region were considered in addition to the mutation information. We generated datasets and performed modeling based on the timing of pandemic waves, their prevalence periods, and mutation information. From the data collected in Nextstrain, time information refers to the collection date rather than the initial report date. In addition, we confirmed that most of the sequence collection and location data were from North America and Europe (Supplementary Fig. 4).

Wave 1 and wave 2 featured the wild type, whereas an Alpha variant with N501Y characterized wave 3. Wave 4 was dominated by the delta variant with L452R and T478K, whereas wave 5 saw the emergence of the Omicron variant. To verify the causal relationships between mutations, we used pandemic wave data to predict subsequent mutations (Fig. 3B). We constructed three prediction models for the analysis: model ① predicting wave 3 using waves 1 and 2; model ② predicting wave 4 using waves 1, 2, and 3; and model ③ predicting wave 4 using waves 1 and 2. The wave 3 prediction model ① showed an accuracy of approximately 0.32 using XGBoost. The wave 4 prediction model ② showed an accuracy of approximately 0.168 using XGBoost. Finally, the wave 4 prediction model showed an accuracy of approximately 0.022 using XGBoost (Fig. 3B).

In waves 1 and 2, approximately 40% of the mutation information required for training included the mutation AA 501 in the Alpha variant in wave 3. In contrast, the mutations at positions 452 and 478 of the delta variant in wave 4 had a frequency of approximately 16.7%, making accurate mutation prediction challenging (Fig. 3C).

For the Omicron variant in wave 5, mutations were found at various locations, making it difficult to make predictions using only information from waves 1, 2, 3, and 4 (Fig. 3B). Additionally, for recent clades 23I, 23H, 24A, and 24 B, the mutation rates in the RBM were fixed at specific locations, presenting challenges for predicting new mutation sites (Fig. 3D).

### Prediction of new SARS-CoV-2 mutations

**Fig. 4:**
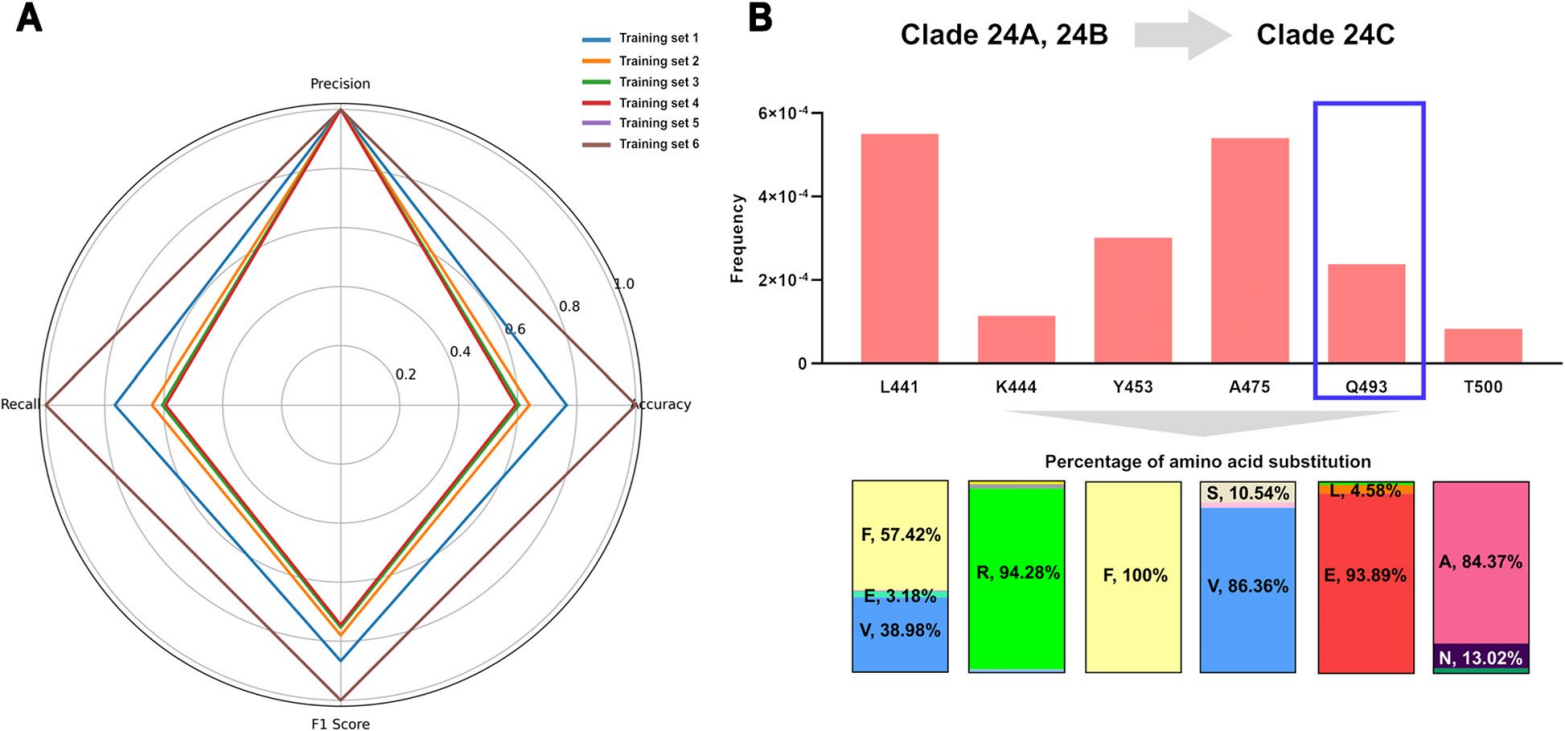
Prediction of future mutations by each learning model after constructing optimal training data for learning. **A** The performance of the models trained using the XGBoost method was evaluated, and the highest accuracy was observed in the models trained on datasets from just after the Omicron variant (wave 5), specifically training set 5 and training set 6. Training set 5 (accuracy: 0.999957, precision: 0.999995, recall: 0.999995, F1 score: 0.999995), training set 6 (accuracy: 0.999977, precision: 0.999998, recall: 0.999997, F1 score: 0.999997). **B** The mutation occurrence positions of 24C (KP.3), which was not collected by Nextstrain (no sequencing information of SARS-CoV-2), were predicted and analyzed through the clade data of 24A and 24B, which recently emerged and were not used in the training process of the existing models. Through this analysis, the mutation positions in the RBM region that may appear in 23C were identified, and amino acid substitutions were confirmed. Positions 441, 444, 453, 475, 493, and 500 were predicted as positions for potential new mutations, and 24C (KP.3) was confirmed as a mutation that occurred at the position marked by the blue box (493). The new predicted mutations were predicted with very low frequencies, with position 441 having the highest value of 0.00055 and position 493, which actually occurred, showing a probability of 0.00024. For amino acid substitutions, L441F and Q493E are likely to occur at the respective locations with Q493E actually occurring. However, considering that the amino acid substitution Q493R had previously occurred, it is possible that it may temporarily appear and then disappear again due to its low predicted rate

We created training data with mutation, collection time, and clade information and trained each model accordingly using XGBoost. Earlier training sets had low accuracy (training set 1, accuracy: 0.765; training set 2, accuracy: 0.639; training set 3, accuracy: 0.605; training set 4, accuracy: 0.593). In contrast, training sets composed solely of Omicron data showed very high accuracy, with training sets 5 and 6 achieving an accuracy of 0.999 (Fig. 4A). Random forest and LightGBM showed similar results to XGBoost, whereas GRU showed a lower performance (Supplementary Fig. 5 and Supplementary Table 5).

Using information from recently reported clades 23H, 23I, 24A, and 24 B, we investigated mutations that could occur in the RBM region. Using information from the recently reported lineages 24A and 24B, we predicted potential mutations in the RBM region. Mutations likely occurred at positions 441, 444, 453, 475, 493, and 500. Mutations at position 493 were also observed in the 23C lineage (Fig. 4B).

The inclusion of the period in which numerous mutations occurred in Omicron, and the temporal and differentiation periods, led to improved mutation prediction performance. Compared to machine learning models such as XGBoost, LightGBM, and random forest, the GRU model showed a relatively lower performance.

### Configuration of a new mutation prediction algorithm in SARS-CoV-2 infectivity prediction

We incorporated an algorithm for predicting new SARS-CoV-2 mutations into our preexisting infectivity prediction system (Artificial Intelligence Analytics Toolkit for predicting viral mutations in protEin: AIVE). To run this algorithm within the system, we set up a server equipped with 96 CPU cores, 256-GB RAM, and 3 RTX 8000 GPUs.

**Fig. 5:**
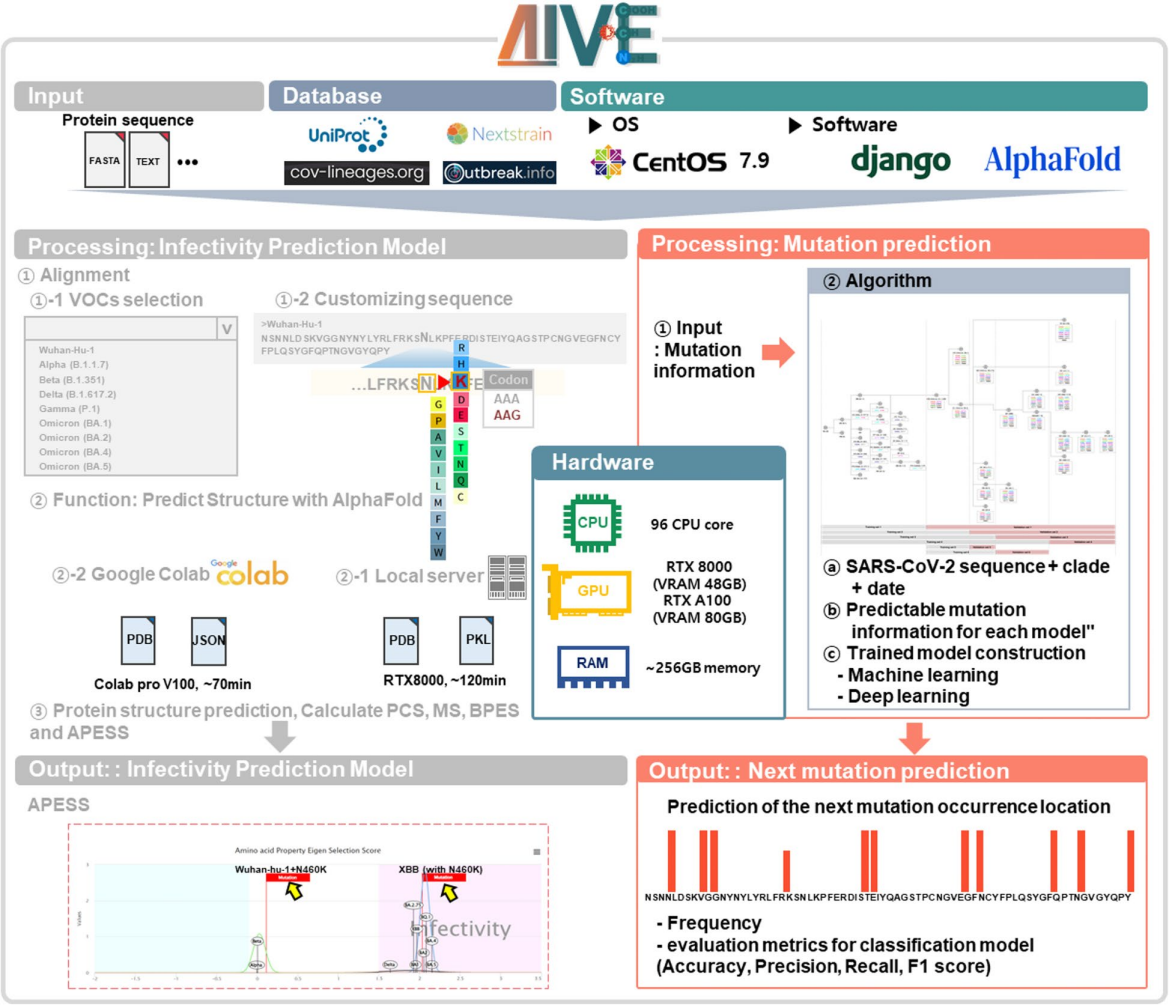
AIVE prediction module

To use the mutation prediction feature, users can access the mutation prediction page and input the RBM sequence they wish to analyze. This process involves using the sequence of the RBM region of the spike protein (P0DTC2) as a reference, with users being able to modify the sequence to generate mutation sequences. Once the desired sequence was generated, the user submitted a task and sent it to the server. The server receives the request, checks the available resources, and allocates them accordingly. The sequence is encoded using the CPU, and then predictions of the mutation positions are performed using pregenerated learning models on the GPU. Upon task completion, the server sent the results back to the user, providing information on the positions of the mutation predictions and model performance (Fig. 5).

## Discussion

Previous infectious diseases either surged briefly, disappeared, or were primarily limited to specific regions [20–22]. Despite the implementation of stringent legal restrictions and limitations on interregional travel to curb the initial spread of COVID-19, more potent lineages have emerged over time [23–26]**.**

SARS-CoV-2 has given rise to numerous lineages and clades, each distinguished by mutations that significantly accumulate as these lineages continue to evolve [27, 28]. Even in infectious diseases such as severe acute respiratory syndrome-associated coronavirus (SARS) and Middle East respiratory syndrome coronavirus (MERS), mutations occur during the epidemic period. However, these infectious diseases do not cause many mutations because of their relatively short durations or regional limitations [29–31].

The numerous mutations that occurred in SARS-CoV-2 resulted in the initially designed COVID-19 vaccines sometimes being ineffective against the Omicron lineage [32, 33]. Additionally, there have been reports of some treatments being ineffective despite the development of appropriate therapeutics owing to mutations [34–36]. Therefore, it is crucial to quickly and accurately predict mutations that occur during the evolution of infectious diseases.

Recently, research using artificial intelligence methods to analyze genomes has become increasingly prominent. Numerous studies have focused on predicting and preventing SARS-CoV-2 infection using genomic information.

Bhowmick et al. proposed two new mutations (P499S and T500R) based on a protein three-dimensional structure prediction algorithm. They estimated binding affinity through the interaction between the receptor-binding domain (RBD) and host cell receptor ACE2 [37]. However, these mutations were inferred based on the original Wuhan-Hu-1 sequence, using physicochemical binding interactions. Therefore, they did not predict the mutations included in the current major lineages or consider clade differentiation and temporal elements of clades and lineages.

Saldivar-Espinoza et al. used machine learning to predict that mutations occurring multiple times independently throughout the evolution of the virus are more likely to result from host deaminase activity than from replication errors [38]. They also predicted the occurrence of mutations based on the SARS-CoV-2 structure. They calculated the values based on whether the changes and mutations in nucleotides belonged to a lineage-associated clade.

In these predictions, the pattern of amino acid substitutions in SARS-CoV-2 was missed because of results at the nucleotide level. They failed to predict new mutations in the spike protein, which has the most mutations that bind to host ACE2.

Moreover, in the early lineages and clades, a small number of mutations occurred in the RBM region. When examining clades 19A-21E that predate Omicron, the major amino acid substitution sites were L452, S477, T478, E484(K), F490, and N501. The number of mutations was also limited to one or two, making accurate predictions challenging.

The temporary emergence and disappearance of mutations have made it more challenging to determine the correlation between mutations, except for those at major substitution sites. This limitation affects the accuracy of predicting the location of the mutation. Analysis of the 12 clades that emerged by 2020 revealed that 8 clades other than 20F, 20H, 20I, and 20 J did not have mutation sides with a frequency above 0.8. The highest frequency of the mutation sites did not exceed 0.3. Only five mutation sites had a frequency greater than 0.1, which made it challenging to identify the characteristics of certain mutations.

On the other hand, after the Omicron variant, specifically clade 21 M, in addition to the existing amino acid substitution sites, mutations occurred at various positions such as N440, V445, G446, F456, N460, E484(A), F486, F490, Q493, Q498, N501, and Y505. Because of these diverse mutations and the availability of numerous samples, the predictions were more accurate (Fig. 2). In the case of recent Omicron variants, the prediction accuracy of mutations decreased because of the increased similarity of mutations (Fig. 4).

In the predicted 24C clade, the Q493E mutation occurred at position 493, where the mutation had previously occurred and then disappeared. Given the mutation frequency data up to clade 23B clade, the likelihood of mutations occurring at position 493 gradually decreased. Considering that the predicted frequency was not high, the Q493E mutation was considered a transient mutation that may disappear again (Supplementary Fig. 6).

As the frequency of mutations becomes increasingly fixed at specific positions, future mutations are likely to occur either as temporary additional mutations at fixed locations or as changes in the type of amino acid in the existing mutations. Over time, the mutation process results in only transient or minor changes.

During the initial stages of the pandemic, mutations sporadically appeared and then disappeared, making it difficult to identify correlations between mutations. However, in recent samples, the difficulty in distinguishing between mutations arose from the increased similarity between samples. This difficulty can lead to overfitting in the prediction models and reduce the accuracy of the predictions.

Our study had several limitations. First, our study focused on the RBM region of the spike protein, where many significant mutations were found. Our study could not fully assess mutation probabilities in other regions. Second, the data used in our study correspond to the time of genome sequencing rather than the initial occurrence of the mutation, indicating that the date information was not entirely reflective of evolutionary continuity. Third, the limited mutation information available before Omicron makes it difficult to predict the mutations that occur in Omicron.

## Conclusions

Our study proposes an accurate method for predicting mutations in infectious diseases using mutation information and time data based on artificial intelligence. This approach aims to enhance the precision of the predictions.

## Supplementary Information

Supplementary figures: Supplementary Figure 1. Analysis of Published Papers Related to COVID-19 from 2020 to 2024. Supplementary Figure 2. This illustrates the workflow for the study, covering the data used, prediction methods, and the resulting prediction. Supplementary Figure 3. Frequency of Mutation Counts per Length of SARS-CoV-2 Domains. Supplementary Figure 4. Regional Distribution of Collected Data Across Different SARS-CoV-2 Wave Periods. Supplementary Figure 5. Comparison of random forest, LightGBM, and GRU model efficiency by training dataset. Supplementary Figure 6. Evaluation of Viral Infectivity Associated with Mutations at Residue Q493. Supplementary tables: Supplementary Table 1. The information that can be provided by Nextstrain, Supplementary Table 2. The types of clades included in each training set and validation set. Supplementary Table 3. The prediction of upcoming mutations using various models during different pandemic wave periods. Supplementary Table 4. The combinations and frequencies of predicted mutations for each data set. Supplementary Table 5. The classification performance metrics results for each training set across different models. Supplementary Table 6. The mutation frequency for each clade in the RBM region. Supplementary methods: The mutation frequency for each clade in the RBM region.

## Data Availability

The data is available for access at GitHub (https://github.com/Honglab-Research/Covid-mutation-probability).
