# Supplementary material for: A prediction of mutations in infectious viruses using artificial intelligence
Source: Genomics Inform. 2024 Oct 8;22:15. doi: 10.1186/s44342-024-00019-y (PMC11463117; doi:10.1186/s44342-024-00019-y)
Supplement: Supplementary file 1 — Supplementary figures: Supplementary Figure 1. Analysis of Published Papers Related to COVID-19 from 2020 to 2024. Supplementary Figure 2. This illustrates the workflow for the study, covering the data used, prediction methods, and the resulting prediction. Supplementary Figure 3. Frequency of Mutation Counts per Length of SARS-CoV-2 Domains. Supplementary Figure 4. Regional Distribution of Collected Data Across Different SARS-CoV-2 Wave Periods. Supplementary Figure 5. Comparison of random forest, LightGBM, and GRU model efficiency by training dataset. Supplementary Figure 6. Evaluation of Viral Infectivity Associated with Mutations at Residue Q493. Supplementary tables: Supplementary Table 1. The information that can be provided by Nextstrain, Supplementary Table 2. The types of clades included in each training set and validation set. Supplementary Table 3. The prediction of upcoming mutations using various models during different pandemic wave periods. Supplementary Table 4. The combinations and frequencies of predicted mutations for each data set. Supplementary Table 5. The classification performance metrics results for each training set across different models. Supplementary Table 6. The mutation frequency for each clade in the RBM region. Supplementary methods: The mutation frequency for each clade in the RBM region. [file 44342_2024_19_MOESM1_ESM.docx]

**A prediction of mutations in infectious viruses using artificial intelligence**

Won Jong Choi^1,2,*^, Jongkeun Park^2,*^, Do Young Seong^1,2^, Dae Sun Chung^2,3^ and
Dongwan Hong^1,2,3,4,5,6,#^

Affiliation:

1. Department of Precision Medicine and Big data, The Catholic University of Korea, College of Medicine, Seoul 06591, Republic of Korea
2. Department of Medical Informatics, The Catholic University of Korea, Seoul 06591, Republic of Korea
3. Department of Medical Sciences, Graduate School of The Catholic University of Korea, College of Medicine, Seoul 06591, Republic of Korea
4. Precision Medicine Research Center, The Catholic University of Korea, College of Medicine, Seoul 06591, Republic of Korea
5. Cancer Evolution Research Center, The Catholic University of Korea, College of Medicine, Seoul 06591, Republic of Korea
6. CMC Institute for Basic Medical Science, The Catholic University of Korea, College of Medicine, Seoul 06591, Republic of Korea

* Co-first authors with equal contribution

# Address correspondence to:

**First corresponding author:** Dongwan Hong, PhD

Department of Medical Informatics, College of Medicine, Catholic University of Korea, Seoul 06591, Republic of Korea

Phone: +82-2-3147-8424; Fax: +82-2-2258-7749

e-mail: dwhong@catholic.ac.kr

**Contents**

**Supplementary Figures**

**Supplementary Figure 1.** Analysis of Published Papers Related to COVID-19 from 2020 to 2024 4

**Supplementary Figure 2.** This illustrates the workflow for the study, covering the data used, prediction methods, and the resulting prediction 5

**Supplementary Figure 3.** Frequency of Mutation Counts per Length of SARS-CoV-2 Domains 6

**Supplementary Figure 4.** Regional Distribution of Collected Data Across Different SARS-CoV-2 Wave Periods 8

**Supplementary Figure 5.** Comparison of random forest, LightGBM, and GRU model efficiency by training dataset 9

**Supplementary Figure 6.** Evaluation of Viral Infectivity Associated with Mutations at Residue Q493 11

**Supplementary Tables**

**Supplementary Table 1.** The information that can be provided by Nextstrain 12

**Supplementary Table 2.** The types of clades included in each training set and validation set 16

**Supplementary Table 3.** The prediction of upcoming mutations using various models during different pandemic wave periods 17

**Supplementary Table 4.** The combinations and frequencies of predicted mutations for each data set 41

**Supplementary Table 5.** The classification performance metrics results for each training set across different models 119

**Supplementary Table 6.** The mutation frequency for each clade in the RBM region 120

**Supplementary Methods.** 121


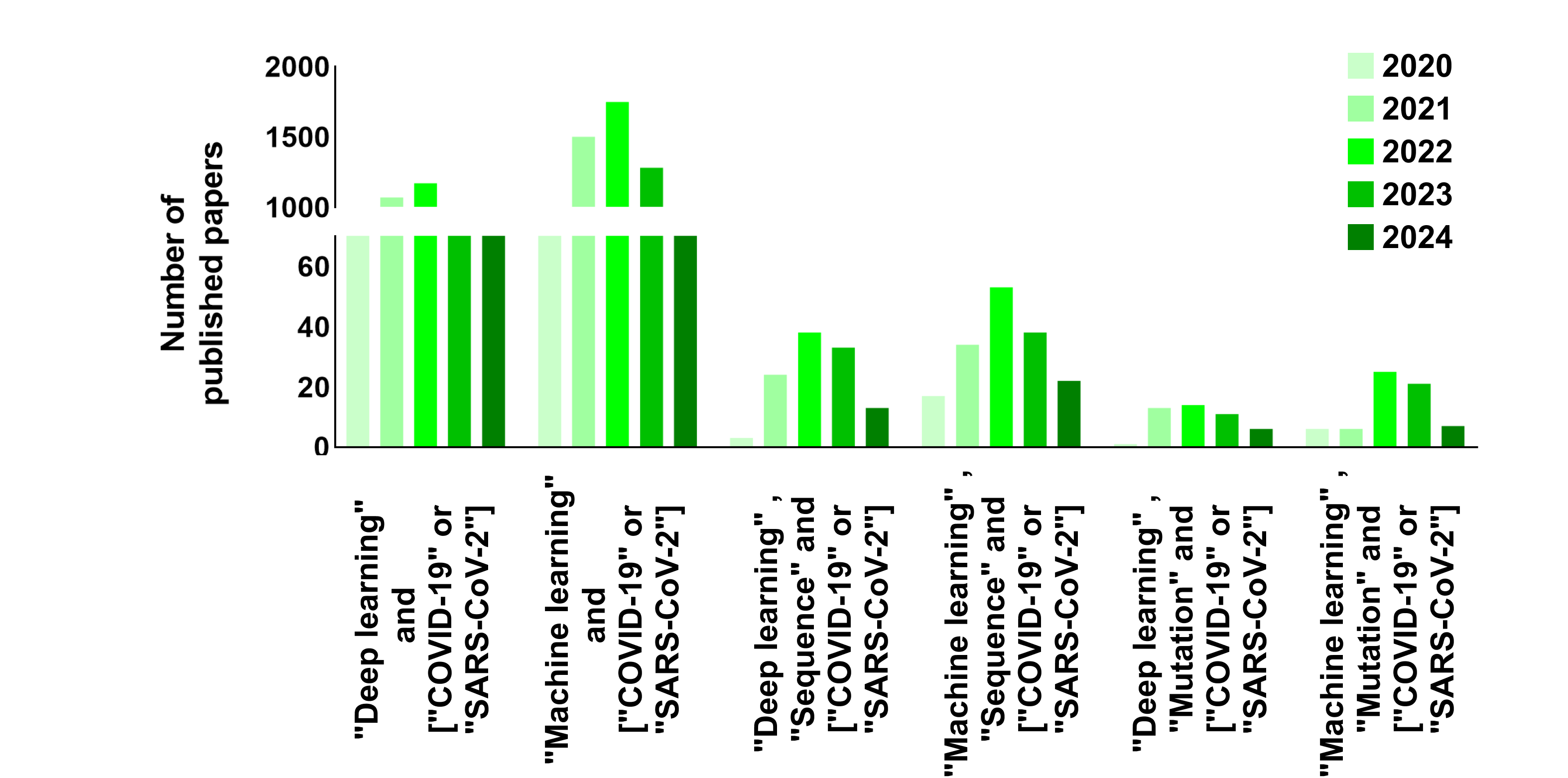


**Supplementary Figure 1. Analysis of Published Papers Related to COVID-19 from 2020 to 2024**.

A systematic review was conducted using PubMed to identify publications related to COVID-19 or SARS-CoV-2 to explore the intersection of artificial intelligence and genomics. The search used the following key terms: “Deep learning,” “Machine learning,” “Sequence,” and “Mutation.” The number of publications identified using the combination of "Deep learning" or "Machine learning" with "mutation" consistently remained below 30 per year. Over the five-year period from 2020 to 2024, the number of papers published with the keywords "Deep learning," "mutation," and ["COVID-19" or "SARS-CoV-2"] were 1, 13, 14, 11, and 6, respectively. Similarly, papers with the keywords "Machine learning," "mutation," and ["COVID-19" or "SARS-CoV-2"] were published in the quantities of 6, 6, 25, and 21 per year, respectively.


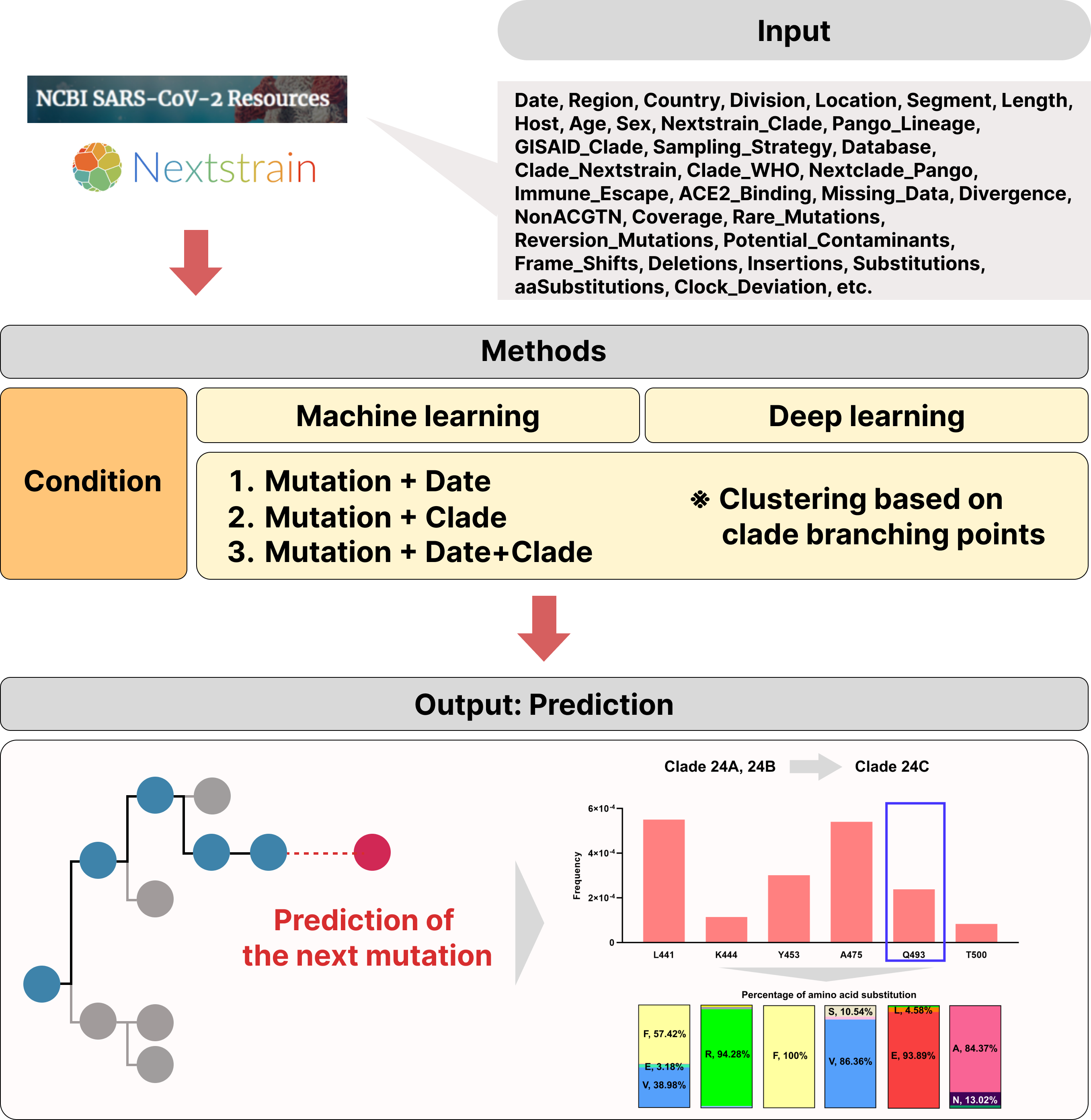


**Supplementary Figure 2. This illustrates the workflow for the study, covering the data used, prediction methods, and the resulting predictions.**


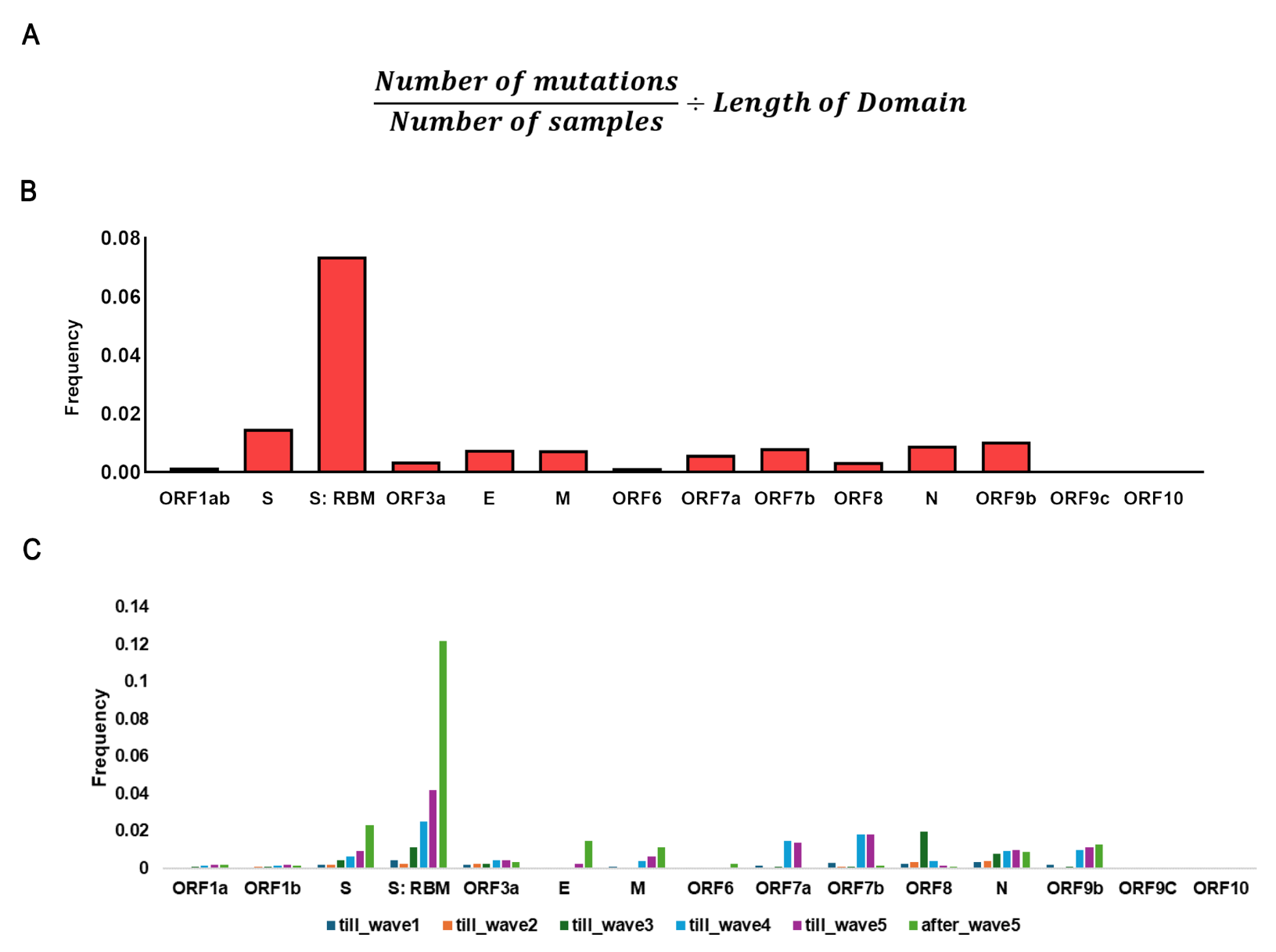


**Supplementary Figure 3. Frequency of Mutation Counts per Length of SARS-CoV-2 Domains**.

A) To determine which domains of SARS-CoV-2 should be prioritized for mutation analysis, we compared the frequency of mutations across various domains. The mutation frequency was calculated as the number of mutations per sample for each domain, and normalization was performed using the length of each domain to ensure an equitable comparison.

B) Using the collected dataset, we identified the domains with the highest frequency of mutations. The Receptor Binding Motif (RBM) of the Spike glycoprotein (S) showed a significantly higher mutation frequency compared to other regions. The frequency of mutations per amino acid (AA) length was calculated for each protein domain of SARS-CoV-2. The mutation frequencies are as follows: ORF1ab: 0.001614038, S: 0.014776418, S:RBM: 0.073600262, ORF3a: 0.003646988, E: 0.007598734, M: 0.007463049, ORF6: 0.001310539, ORF7a: 0.005979663, ORF7b: 0.00810612, ORF8: 0.003463883, N: 0.009054728, ORF9b: 0.010394056, ORF9c: 0, ORF10: 0. Abbreviations: ORF, open reading frame; S, spike protein; RBM, receptor binding motif; E, envelope; M, membrane; N, nucleocapsid.

C) To compare the positivity rate of mutations across different waves of SARS-CoV-2, the same analysis was performed using data corresponding to each wave period. Consistent with the results from the entire dataset, the Receptor Binding Motif (RBM) of the Spike glycoprotein exhibited a higher mutation frequency than other domains in all waves. Notably, following wave 5, driven by the Omicron variant, the mutation frequency in the RBM region progressively increased over time.


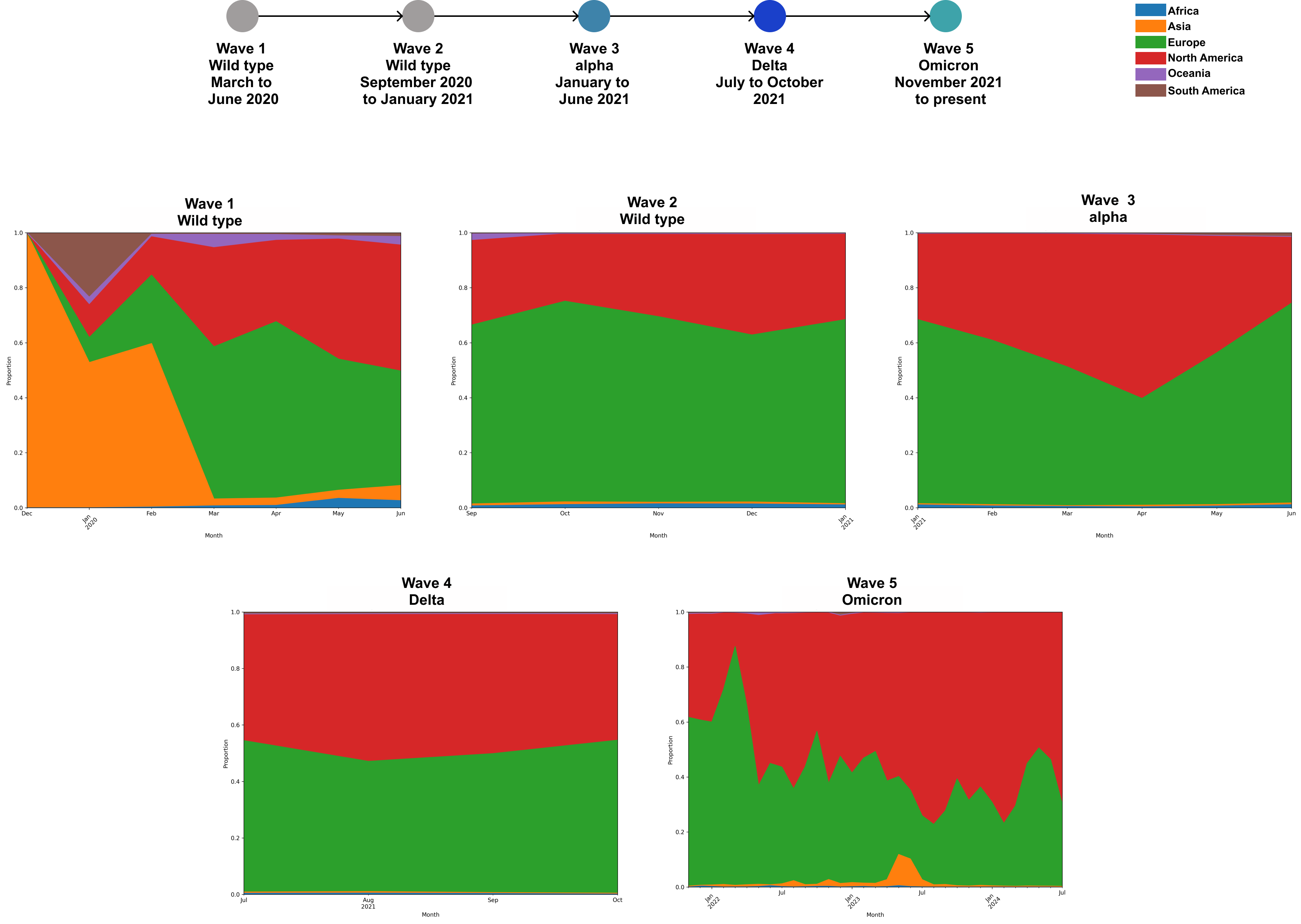


**Supplementary Figure 4. Regional Distribution of Collected Data Across Different SARS-CoV-2 Wave Periods.**

The majority of data collected from Wave 2 to the present consists of NGS sequencing samples, primarily from Europe and North America.


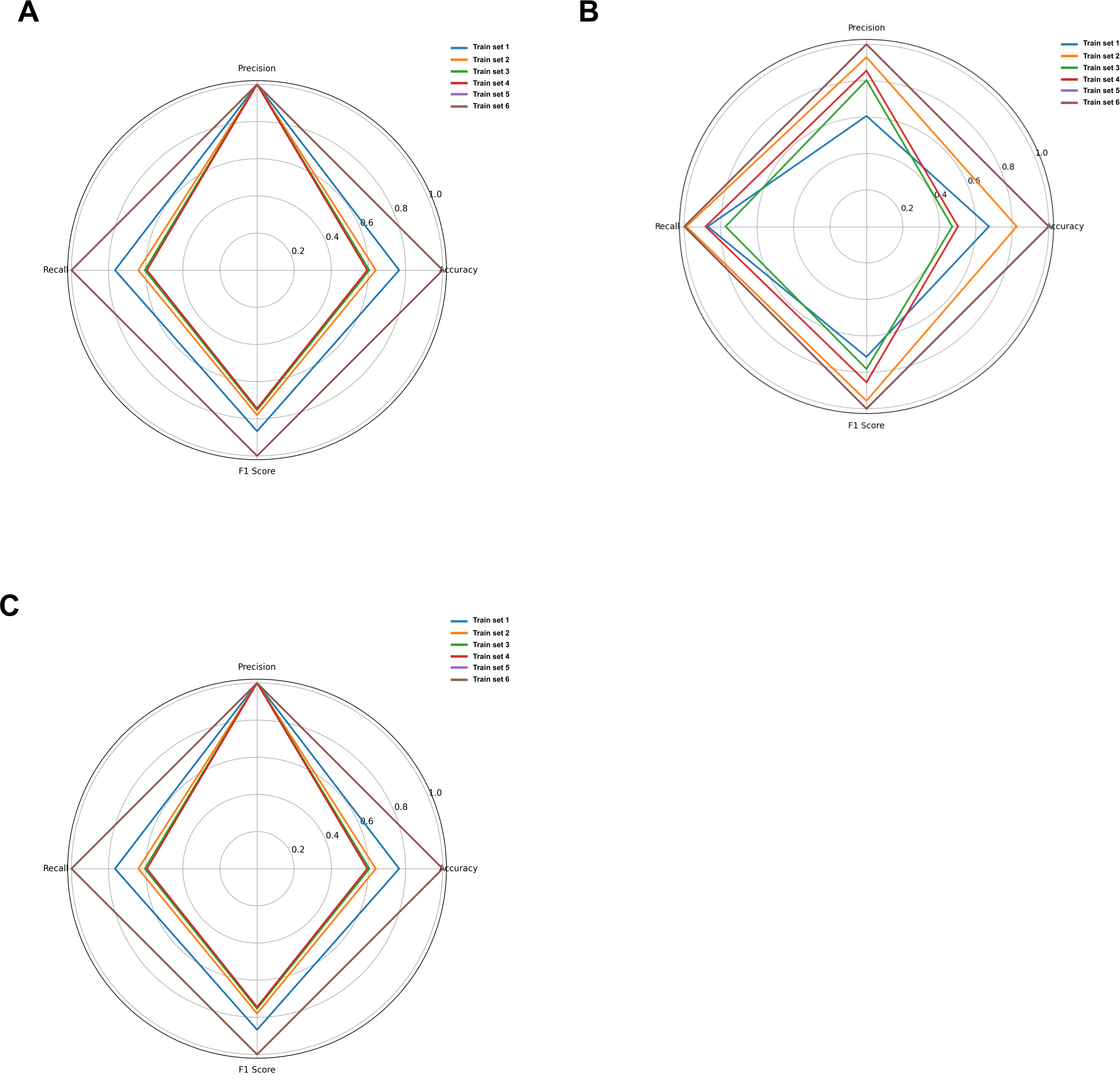


**Supplementary Figure 5. Comparison of random forest, LightGBM, and GRU model efficiency by training dataset**.

A) Random Forest model : Training Set 1 (Accuracy: 0.978, Precision: 0.733, Recall: 0.713, and F1 Score: 0.654), Training Set 2 (Accuracy: 0.994, Precision: 0.936, Recall: 0.916, and F1 Score: 0.910), Training Set 3 (Accuracy: 0.996, Precision: 0.954, Recall: 0.935, and F1 Score: 0.933), Training Set 4 (Accuracy: 0.997, Precision: 0.946, Recall: 0.929, and F1 Score: 0.924), Training Set 5 (Accuracy: 0.998, Precision: 0.997, Recall: 0.998, and F1 Score: 0.998), Training Set 6 (Accuracy: 0.999, Precision: 0.986, Recall: 0.987, and F1 Score: 0.986)

B) GRU model : Training Set 1 (Accuracy: 0.766, Precision: 1.000, Recall: 0.766, and F1 Score: 0.867), Training Set 2 (Accuracy: 0.640, Precision: 1.000, Recall: 0.640, and F1 Score: 0.780), Training Set 3 (Accuracy: 0.605, Precision: 1.000, Recall: 0.605, and F1 Score: 0.754), Training Set 4 (Accuracy: 0.593, Precision: 1.000, Recall: 0.593, and F1 Score: 0.745), Training Set 5 (Accuracy: 1.000, Precision: 1.000, Recall: 1.000, and F1 Score: 1.000), Training Set 6 (Accuracy: 1.000, Precision: 1.000, Recall: 1.000, and F1 Score: 1.000)

C) LightGBM model : Training Set 1 (Accuracy: 0.978, Precision: 0.733, Recall: 0.713, and F1 Score: 0.654), Training Set 2 (Accuracy: 0.994, Precision: 0.936, Recall: 0.916, and F1 Score: 0.909), Training Set 3 (Accuracy: 0.996, Precision: 0.953, Recall: 0.935, and F1 Score: 0.933), Training Set 4 (Accuracy: 0.997, Precision: 0.945, Recall: 0.929, and F1 Score: 0.924), Training Set 5 (Accuracy: 0.998, Precision: 0.997, Recall: 0.998, and F1 Score: 0.998), Training Set 6 (Accuracy: 0.999, Precision: 0.986, Recall: 0.987, and F1 Score: 0.986)


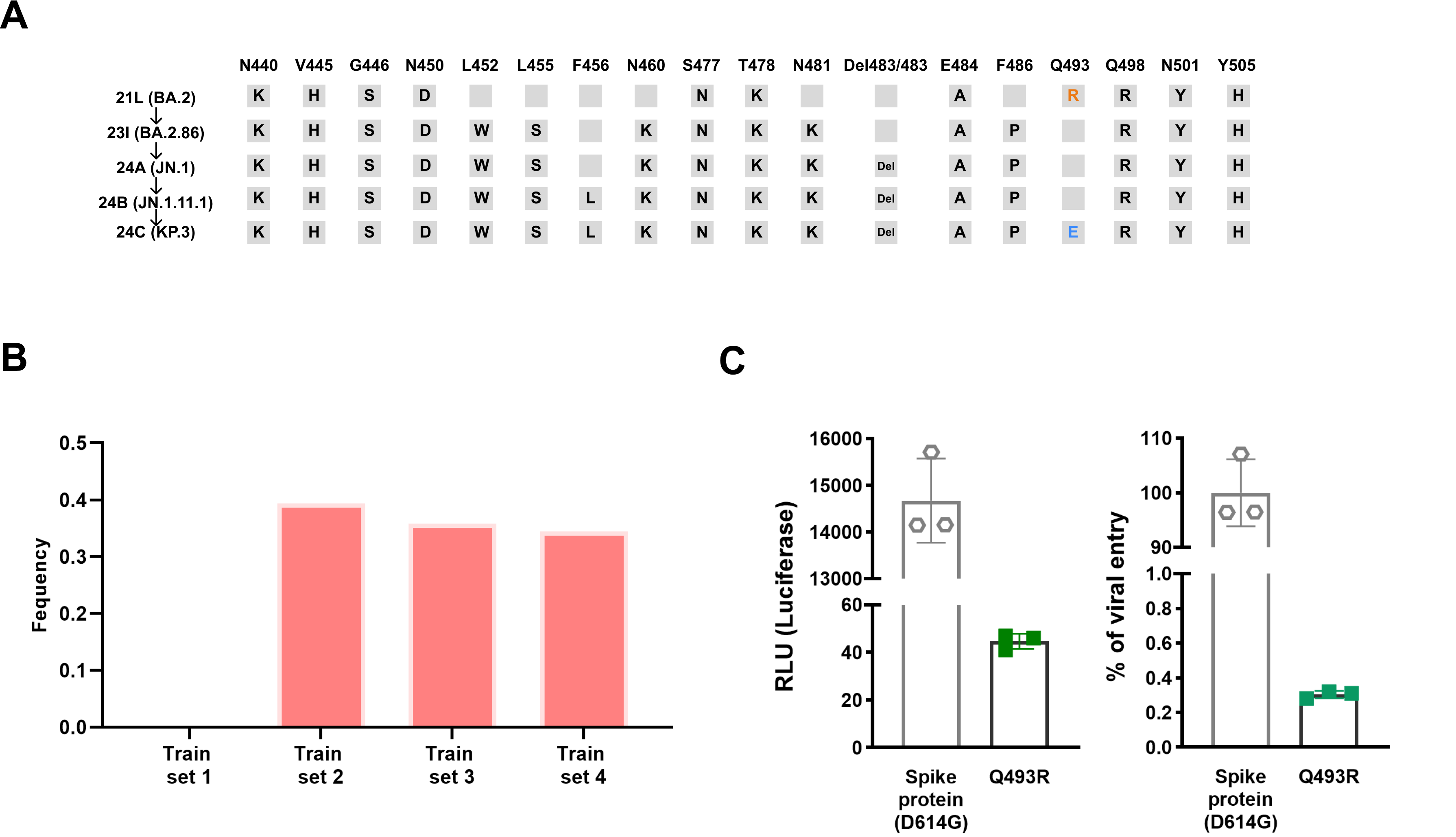


**Supplementary Figure 6. Evaluation of Viral Infectivity Associated with Mutations at Residue Q493.**

A) The Q493 residue underwent a substitution from Q to R in the early Omicron variant 21L (BA.2). This mutation subsequently disappeared during further evolutionary stages but has recently reappeared as a substitution to E at the same residue in the 24C (KP.3) clade.

B) Analysis of evolutionary trends based on clade differentiation indicates a decreasing prevalence of mutations at residue Q493 over time.

C) Experimental assays evaluating the previously identified Q493R mutation demonstrated minimal impact on viral infectivity.

Given these observations and the low frequency of the Q493E mutation in predictive models, it is plausible that the Q493E mutation may be transient and likely to disappear again.

**Supplementary Table 1. The information that can be provided by Nextstrain**.

| **index** | **Description** | **example value** |
| --- | --- | --- |
| **Strain** | Identifier of the virus sample. | GBR/SFK-USAFSAM-S22161/2023 |
| **Virus** | Type of virus (e.g., SARS-CoV-2). | ncov |
| **GISAID_EPI_ISL** | Unique identifier in the GISAID database. | EPI_ISL_18873924 |
| **GenBank_Accession** | Unique accession number in the GenBank database. | PP317667 |
| **GenBank_Accession_Rev** | Reverse accession number in the GenBank database. | PP317667.1 |
| **SRA_Accession** | Accession number in the NCBI Sequence Read Archive (SRA) database. | SRR27944759 |
| **Date** | Date when the sample was collected. | 2023-12-26 |
| **Region** | Continent where the sample was collected. | Europe |
| **Country** | Country where the sample was collected. | United Kingdom |
| **Division** | Sub-national region where the sample was collected. | England |
| **Location** | City or municipality where the sample was collected. | Suffolk |
| **Region_Exposure** | Continent where the virus exposure occurred. | Europe |
| **Country_Exposure** | Country where the virus exposure occurred. | United Kingdom |
| **Division_Exposure** | Sub-national region where the virus exposure occurred. | England |
| **Segment** | Refers to a specific segment of the viral genome; some viruses have genomes composed of multiple segments. | genome |
| **Length** | Length of the viral genome, expressed in base pairs. | 29740 |
| **Host** | Host organism of the virus. | Homo sapiens |
| **Age** | Age of the host. | 2 |
| **Sex** | Sex of the host. | Male |
| **Nextstrain_Clade** | Clade as defined by Nextstrain. | 23I (Omicron) |
| **Pango_Lineage** | Lineage as defined by Pango. | JN.2 |
| **GISAID_Clade** | Clade as defined by GISAID. | ? |
| **Originating_Lab** | Laboratory where the virus sample was originally collected. | LAKENHEATH AB |
| **Submitting_Lab** | Laboratory that submitted the data to the database. | United States Air Force School of Aerospace Medicine Public Health Submission Group, 711th Human Performance Wing |
| **Authors** | Researchers who submitted the data. | Fries et al |
| **URL** | URL where the virus data is stored. | https://www.ncbi.nlm.nih.gov/nuccore/PP317667 |
| **Title** | Title of the related research paper. |  |
| **Paper_URL** | URL of the related research paper. | ? |
| **Date_Submitted** | Date when the data was submitted to the database. | 2024-02-09 |
| **Date_Updated** | Date when the data was last updated. | 2024-02-09 |
| **Sampling_Strategy** | Sampling strategy used (e.g., random sampling, targeted sampling). | ? |
| **Database** | Name of the database where the data is stored. | genbank |
| **Clade_Nextstrain** | Clade as defined by Nextstrain. | 23I |
| **Clade_WHO** | Clade as defined by the WHO. | Omicron |
| **Nextclade_Pango** | Lineage as defined by Nextclade and Pango. | JN.2 |
| **Immune_Escape** | Whether the virus exhibits immune escape properties. | 1.283 |
| **ACE2_Binding** | Whether the virus binds to the ACE2 receptor. | 1.048 |
| **Missing_Data** | Amount of missing data. | 593.0 |
| **Divergence** | Degree of genomic variation. | 115.0 |
| **NonACGTN** | Number of bases in the genome sequence that are not A, C, G, T, or N. | 1.0 |
| **Coverage** | Coverage of the genome sequence, indicating the completeness of the sampled genome. | 0.976992275022573 |
| **Rare_Mutations** | Number of rare mutations. | 4.0 |
| **Reversion_Mutations** | Number of reversion mutations. | 0.0 |
| **Potential_Contaminants** | Presence of potential contaminants. | 0.0 |
| **QC_Missing_Data** | Data missing during the QC process. | good |
| **QC_Mixed_Sites** | Information on mixed sites during the QC process. | good |
| **QC_Rare_Mutations** | Rare mutations identified during the QC process. | good |
| **QC_SNP_Clusters** | SNP clusters identified during the QC process. | good |
| **QC_Frame_Shifts** | Frame shift mutations identified during the QC process. | good |
| **QC_Stop_Codons** | Stop codons identified during the QC process. | good |
| **QC_Overall_Score** | Overall score from the QC process, used to evaluate data quality. | 2.177627 |
| **QC_Overall_Status** | Overall status from the QC process, with possible values 'bad,' 'good,' 'mediocre,' or 'nan'. | good |
| **Frame_Shifts** | Frame shift mutations found in the genome. | ORF7a:102-122 |
| **Deletions** | Deletion mutations found in the genome. | 11288-11296,21633-21641,21765-21770,21992-21994,22194-22196,23009-23011,27682-27694,28362-28370,29734-29759 |
| **Insertions** | Insertion mutations found in the genome. | 21608:TCATGCCGCTGT |
| **Substitutions** | Substitution mutations found in the genome. | C241T,G332A,T670G,C897A,A2127G,C2245T,C2790T,C3037T,G3431T,G4184A,C4321T,A6183G,A7842G,C8293T,G8393A,C9344T,A9424G,C9534T,C10029T,C10198T,G10447A,C10449A,G11042T,C12789T,C12815T,C12880T,T13339C,C14408T,C14741T,C15714T,T15756A,C17410T,A18163G,A18492G,C19955T,A20055G,C21618T,C21622T,G21624C,C21711T,G21941T,G21987A,T22032C,C22033A,A22034G,T22200G,C22208T,C22295A,C22353A,A22556G,G22577C,G22578A,A22629C,C22674T,T22679C,C22686T,A22688G,G22770A,G22775A,A22786C,G22813T,T22882G,G22895C,T22896A,G22898A,A22910G,C22916T,T22917G,T22942A,G22992A,C22995A,T23005A,G23012A,T23018C,T23019C,A23055G,A23063T,T23075C,G23222A,C23271T,A23403G,C23423T,C23525T,T23599G,C23604G,C23854A,G23948T,C24378T,A24424T,T24469A,C24990T,C25000T,C25207T,C25584T,C26060T,C26270T,G26529C,C26577G,C26681T,G26709A,C26833T,C26858T,A27259C,G27382C,A27383T,T27384C,C27807T,A28271T,C28311T,G28373A,G28881A,G28882A,G28883C,C28958A,A29510C |
| **aaSubstitutions** | Amino acid substitution mutations. | E:T9I,M:D3H,M:Q19E,M:A63T,M:A104V,N:P13L,N:G34R,N:R203K,N:G204R,N:Q229K,N:S413R,ORF1a:V23I,ORF1a:S135R,ORF1a:A211D,ORF1a:Y621C,ORF1a:T842I,ORF1a:V1056L,ORF1a:G1307S,ORF1a:K1973R,ORF1a:N2526S,ORF1a:A2710T,ORF1a:L3027F,ORF1a:T3090I,ORF1a:T3255I,ORF1a:P3395H,ORF1a:V3593F,ORF1a:T4175I,ORF1b:P314L,ORF1b:S425F,ORF1b:R1315C,ORF1b:I1566V,ORF1b:T2163I,ORF3a:T223I,ORF6:D61L,ORF9b:P10S,S:T19I,S:R21T,S:A27S,S:S50L,S:V127F,S:G142D,S:F157S,S:R158G,S:L212I,S:V213G,S:L216F,S:H245N,S:A264D,S:I332V,S:G339H,S:K356T,S:S371F,S:S373P,S:S375F,S:T376A,S:R403K,S:D405N,S:R408S,S:K417N,S:N440K,S:V445H,S:G446S,S:N450D,S:L452W,S:N460K,S:S477N,S:T478K,S:N481K,S:E484K,S:F486P,S:Q498R,S:N501Y,S:Y505H,S:E554K,S:A570V,S:D614G,S:P621S,S:H655Y,S:N679K,S:P681R,S:N764K,S:D796Y,S:S939F,S:Q954H,S:N969K,S:P1143L |
| **Clock_Deviation** | Deviation in the molecular clock, indicating variation in the rate of genomic mutation. This is an important factor in viral phylogenetic studies. | 2.0 |

**Supplementary Table 2. The types of clades included in each training set and validation set**

|  | **Clades included in the Training set** | **Clades included in the Validation set** |
| --- | --- | --- |
| **Set 1** | '19A, 19B, 20A, 20B, 20C, 20D, 20E, 20F, 20G, 20H, 20I, 20J, 21A, 21B, 21C, 21D, 21E, 21F, 21G, 21H, 21I, 21J | 21K, 21L, 21M, 22A, 22B, 22C, 22D, 22E, 22F, 23A, 23B, 23C, 23D, 23E, 23F, 23G, 23H, 23I |
| **Set 2** | 19A, 19B, 20A, 20B, 20C, 20D, 20E, 20F, 20G, 20H, 20I, 20J, 21A, 21B, 21C, 21D, 21E, 21F, 21G, 21H, 21I, 21J, 21K, 21L, 21M | 22A, 22B, 22C, 22D, 22E, 22F, 23A, 23B, 23C, 23D, 23E, 23F, 23G, 23H, 23I |
| **Set 3** | 19A, 19B, 20A, 20B, 20C, 20D, 20E, 20F, 20G, 20H, 20I, 20J, 21A, 21B, 21C, 21D, 21E, 21F, 21G, 21H, 21I, 21J, 21K, 21L, 21M, 22A, 22B, 22C, 22D, 22E, 22F | 23A, 23B, 23C, 23D, 23E, 23F, 23G, 23H, 23I |
| **Set 4** | 19A, 19B, 20A, 20B, 20C, 20D, 20E, 20F, 20G, 20H, 20I, 20J, 21A, 21B, 21C, 21D, 21E, 21F, 21G, 21H, 21I, 21J, 21K, 21L, 21M, 22A, 22B, 22C, 22D, 22E, 22F, 23A, 23B | 23C, 23D, 23E, 23F, 23G, 23H, 23I |
| **Set 5** | 21K, 21L, 21M | 22A, 22B, 22C, 22D, 22E, 22F |
| **Set 6** | 21K, 21L, 21M, 22A, 22B, 22C, 22D | 22E, 22F, 23A, 23B |

**Supplementary Table 3. The prediction of upcoming mutations using various models during different pandemic wave periods. ① training set: wave 1,2 → validation set: wave 3, ② training set: wave 1,2,3 → validation set: wave 4 , and ③ training set: wave 1,2 → validation set: wave 4**

| **XGBoost** | | | | | |
| --- | --- | --- | --- | --- | --- |
| **①** | | **②** | | **③** | |
| **mutation combination** | **frequency** | **mutation combination** | **frequency** | **mutation combination** | **frequency** |
| 501 | 0.39506692 | 501 | 0.624459271 | 501 | 0.39506692 |
| 477 | 0.361149711 | 452, 478 | 0.166372756 | 477 | 0.361149711 |
| 439 | 0.089830323 | 452 | 0.061685103 | 439 | 0.089830323 |
| 452 | 0.078384407 | 484, 501 | 0.038275571 | 452 | 0.078384407 |
| 484, 501 | 0.018905873 | 484 | 0.036021946 | 484, 501 | 0.018905873 |
| 484 | 0.013530315 | 477 | 0.025508784 | 484 | 0.013530315 |
| N/A | 0.008246179 | 478 | 0.015297608 | N/A | 0.008246179 |
| 494 | 0.006106926 | 494, 501 | 0.005176577 | 494 | 0.006106926 |
| 478 | 0.005722958 | 494 | 0.004423866 | 478 | 0.005722958 |
| 494, 501 | 0.003931105 | 439 | 0.004405837 | 494, 501 | 0.003931105 |
| 452, 478 | 0.003857968 | 490, 501 | 0.002187143 | 452, 478 | 0.003857968 |
| 446 | 0.002102684 | 452, 490 | 0.001551621 | 446 | 0.002102684 |
| 475 | 0.001883274 | 452, 484 | 0.001168505 | 475 | 0.001883274 |
| 455 | 0.0013896 | 452, 501 | 0.001033287 | 455 | 0.0013896 |
| 479 | 0.001316463 | 440, 484 | 0.000747077 | 479 | 0.001316463 |
| 483 | 0.001115337 | 452, 477 | 0.00053749 | 483 | 0.001115337 |
| 460 | 0.001023916 | 440, 501 | 0.00053749 | 460 | 0.001023916 |
| 440, 446, 477, 478, 484, 493, 496, 498, 501, 505 | 0.000859358 | 440 | 0.000528475 | 440, 446, 477, 478, 484, 493, 496, 498, 501, 505 | 0.000859358 |
| 490 | 0.000786221 | 478, 501 | 0.000525095 | 490 | 0.000786221 |
| 470 | 0.000493674 | 450 | 0.000475515 | 470 | 0.000493674 |
| 476 | 0.000475389 | 455, 501 | 0.000449598 | 476 | 0.000475389 |
| 475, 501 | 0.000329116 | 477, 501 | 0.000446218 | 475, 501 | 0.000329116 |
| 446, 477 | 0.000274263 | 446, 452, 478 | 0.000441711 | 446, 477 | 0.000274263 |
| 493 | 0.000237695 | 496, 501 | 0.000367341 | 493 | 0.000237695 |
| 440, 445, 446, 460, 477, 478, 484, 486, 490, 498, 501, 505 | 0.000219411 | 452, 503 | 0.00033241 | 440, 445, 446, 460, 477, 478, 484, 486, 490, 498, 501, 505 | 0.000219411 |
| 440, 477, 478, 484, 493, 498, 501, 505 | 0.000164558 | 475, 501 | 0.000274942 | 440, 477, 478, 484, 493, 498, 501, 505 | 0.000164558 |
| 477, 478, 484, 493, 498, 501, 505 | 0.000146274 | 471, 501 | 0.000264801 | 477, 478, 484, 493, 498, 501, 505 | 0.000146274 |
| 477, 494 | 0.000127989 | 439, 484 | 0.000252406 | 477, 494 | 0.000127989 |
| 452, 477 | 0.000127989 | 493, 501 | 0.000241138 | 452, 477 | 0.000127989 |
| 477, 493 | 0.000109705 | 470 | 0.000226489 | 477, 493 | 0.000109705 |
| 477, 501 | 9.1421E-05 | 483, 501 | 0.000221982 | 477, 501 | 9.1421E-05 |
| 440, 452, 477, 478, 484, 493, 498, 501, 505 | 9.1421E-05 | 477, 484 | 0.000211841 | 440, 452, 477, 478, 484, 493, 498, 501, 505 | 9.1421E-05 |
| 452, 501 | 9.1421E-05 | 459 | 0.000210714 | 452, 501 | 9.1421E-05 |
| 439, 478 | 9.1421E-05 | 446, 501 | 0.000192685 | 439, 478 | 9.1421E-05 |
| 477, 483 | 9.1421E-05 | 479, 501 | 0.000182544 | 477, 483 | 9.1421E-05 |
| 477, 484 | 9.1421E-05 | 452, 478, 484 | 0.000181417 | 477, 484 | 9.1421E-05 |
| 478, 501 | 9.1421E-05 | 479 | 0.000179163 | 478, 501 | 9.1421E-05 |
| 440, 452, 477, 478, 484, 486, 498, 501, 505 | 7.31368E-05 | 490 | 0.000160007 | 440, 452, 477, 478, 484, 486, 498, 501, 505 | 7.31368E-05 |
| 477, 478, 484, 486, 490, 498, 501, 505 | 7.31368E-05 | 484, 494 | 0.000160007 | 477, 478, 484, 486, 490, 498, 501, 505 | 7.31368E-05 |
| 478, 484 | 7.31368E-05 | 440, 452 | 0.00013071 | 478, 484 | 7.31368E-05 |
| 490, 501 | 7.31368E-05 | 452, 477, 478 | 0.000129583 | 490, 501 | 7.31368E-05 |
| 446, 452, 478 | 5.48526E-05 | 444, 452 | 0.000116062 | 446, 452, 478 | 5.48526E-05 |
| 485 | 5.48526E-05 | 444, 452, 478 | 0.000113808 | 485 | 5.48526E-05 |
| 505 | 5.48526E-05 | 476, 501 | 0.000111554 | 505 | 5.48526E-05 |
| 476, 501 | 5.48526E-05 | 444, 501 | 0.000108174 | 476, 501 | 5.48526E-05 |
| 479, 490, 501 | 5.48526E-05 | 458, 501 | 0.000101413 | 479, 490, 501 | 5.48526E-05 |
| 440, 484 | 5.48526E-05 | 485, 501 | 9.92E-05 | 440, 484 | 5.48526E-05 |
| 446, 501 | 5.48526E-05 | 508 | 9.01E-05 | 446, 501 | 5.48526E-05 |
| 483, 484 | 3.65684E-05 | 452, 478, 479 | 8.79E-05 | 483, 484 | 3.65684E-05 |
| 452, 478, 490 | 3.65684E-05 | 452, 494 | 8.23E-05 | 452, 478, 490 | 3.65684E-05 |
| 477, 505 | 3.65684E-05 | 446 | 8.00E-05 | 477, 505 | 3.65684E-05 |
| 439, 494 | 3.65684E-05 | 470, 501 | 8.00E-05 | 439, 494 | 3.65684E-05 |
| 483, 501 | 3.65684E-05 | 452, 478, 501 | 6.54E-05 | 483, 501 | 3.65684E-05 |
| 477, 478, 484, 493, 496, 498, 501, 505 | 3.65684E-05 | 450, 501 | 6.20E-05 | 477, 478, 484, 493, 496, 498, 501, 505 | 3.65684E-05 |
| 475, 477 | 1.82842E-05 | 452, 478, 493 | 6.20E-05 | 475, 477 | 1.82842E-05 |
| 452, 476, 478 | 1.82842E-05 | 484, 490, 501 | 6.08E-05 | 452, 476, 478 | 1.82842E-05 |
| 477, 490 | 1.82842E-05 | N/A | 5.86E-05 | 477, 490 | 1.82842E-05 |
| 439, 446 | 1.82842E-05 | 478, 484 | 5.41E-05 | 439, 446 | 1.82842E-05 |
| 440, 477, 478, 484, 486, 498, 501, 505 | 1.82842E-05 | 468, 501 | 5.18E-05 | 440, 477, 478, 484, 486, 498, 501, 505 | 1.82842E-05 |
| 498, 501, 505 | 1.82842E-05 | 440, 446, 477, 478, 484, 493, 496, 498, 501, 505 | 4.96E-05 | 498, 501, 505 | 1.82842E-05 |
| 440, 446, 477, 478, 484, 493, 496, 498, 501 | 1.82842E-05 | 452, 483 | 4.85E-05 | 440, 446, 477, 478, 484, 493, 496, 498, 501 | 1.82842E-05 |
| 496, 498, 501, 505 | 1.82842E-05 | 501, 503 | 4.85E-05 | 496, 498, 501, 505 | 1.82842E-05 |
| 476, 484, 501 | 1.82842E-05 | 483, 484 | 4.62E-05 | 476, 484, 501 | 1.82842E-05 |
| 446, 478 | 1.82842E-05 | 452, 478, 483 | 4.62E-05 | 446, 478 | 1.82842E-05 |
| 446, 455, 498, 505 | 1.82842E-05 | 483 | 4.39E-05 | 446, 455, 498, 505 | 1.82842E-05 |
| 446, 452 | 1.82842E-05 | 445, 501 | 4.39E-05 | 446, 452 | 1.82842E-05 |
| 440, 446, 477, 478, 484, 493, 496 | 1.82842E-05 | 481, 501 | 4.28E-05 | 440, 446, 477, 478, 484, 493, 496 | 1.82842E-05 |
| 440, 445, 446, 450, 452, 460, 477, 478, 481, 484, 486, 498, 501, 505 | 1.82842E-05 | 444 | 4.17E-05 | 440, 445, 446, 450, 452, 460, 477, 478, 481, 484, 486, 498, 501, 505 | 1.82842E-05 |
| 470, 501 | 1.82842E-05 | 499, 501 | 4.17E-05 | 470, 501 | 1.82842E-05 |
| 496, 501 | 1.82842E-05 | 475 | 4.06E-05 | 496, 501 | 1.82842E-05 |
| 446, 493, 496, 498, 501 | 1.82842E-05 | 446, 452 | 4.06E-05 | 446, 493, 496, 498, 501 | 1.82842E-05 |
| 479, 501 | 1.82842E-05 | 476 | 3.94E-05 | 479, 501 | 1.82842E-05 |
| 452, 455, 481 | 1.82842E-05 | 452, 478, 494 | 3.83E-05 | 452, 455, 481 | 1.82842E-05 |
| 452, 475 | 1.82842E-05 | 452, 478, 503 | 3.72E-05 | 452, 475 | 1.82842E-05 |
| 440, 446, 477, 484, 493, 496, 498, 501, 505 | 1.82842E-05 | 440, 446, 477, 478, 484, 493 | 3.49E-05 | 440, 446, 477, 484, 493, 496, 498, 501, 505 | 1.82842E-05 |
| 452, 490 | 1.82842E-05 | 501, 507 | 3.38E-05 | 452, 490 | 1.82842E-05 |
| 477, 478 | 1.82842E-05 | 452, 455 | 3.38E-05 | 477, 478 | 1.82842E-05 |
| 501, 505 | 1.82842E-05 | 478, 494 | 3.38E-05 | 501, 505 | 1.82842E-05 |
| 460, 477, 478, 484, 486, 490, 498, 501, 505 | 1.82842E-05 | 471 | 3.27E-05 | 460, 477, 478, 484, 486, 490, 498, 501, 505 | 1.82842E-05 |
| 452, 484 | 1.82842E-05 | 463, 501 | 3.27E-05 | 452, 484 | 1.82842E-05 |
| 478, 479 | 1.82842E-05 | 501, 508 | 3.16E-05 | 478, 479 | 1.82842E-05 |
| 476, 478, 501 | 1.82842E-05 | 472, 501 | 3.04E-05 | 476, 478, 501 | 1.82842E-05 |
| 485, 501 | 1.82842E-05 | 452, 476, 478 | 3.04E-05 | 485, 501 | 1.82842E-05 |
| 455, 490 | 1.82842E-05 | 503 | 2.93E-05 | 455, 490 | 1.82842E-05 |
|  |  | 501, 505 | 2.93E-05 |  |  |
|  |  | 468 | 2.93E-05 |  |  |
|  |  | 446, 477 | 2.93E-05 |  |  |
|  |  | 493 | 2.70E-05 |  |  |
|  |  | 455 | 2.70E-05 |  |  |
|  |  | 452, 476 | 2.59E-05 |  |  |
|  |  | 452, 455, 478 | 2.48E-05 |  |  |
|  |  | 468, 490, 501 | 2.48E-05 |  |  |
|  |  | 477, 494 | 2.37E-05 |  |  |
|  |  | 486, 501 | 2.37E-05 |  |  |
|  |  | 452, 479 | 2.37E-05 |  |  |
|  |  | 452, 458, 478 | 2.14E-05 |  |  |
|  |  | 452, 455, 501 | 1.92E-05 |  |  |
|  |  | 446, 484 | 1.92E-05 |  |  |
|  |  | 452, 463, 478 | 1.80E-05 |  |  |
|  |  | 452, 453 | 1.69E-05 |  |  |
|  |  | 477, 478, 484, 493, 496, 498, 501, 505 | 1.58E-05 |  |  |
|  |  | 481 | 1.58E-05 |  |  |
|  |  | 460 | 1.58E-05 |  |  |
|  |  | 450, 477 | 1.58E-05 |  |  |
|  |  | 482, 501 | 1.58E-05 |  |  |
|  |  | 483, 484, 501 | 1.58E-05 |  |  |
|  |  | 476, 484 | 1.35E-05 |  |  |
|  |  | 452, 478, 490 | 1.35E-05 |  |  |
|  |  | 452, 478, 499 | 1.35E-05 |  |  |
|  |  | 445 | 1.24E-05 |  |  |
|  |  | 452, 470 | 1.24E-05 |  |  |
|  |  | 477, 484, 501 | 1.24E-05 |  |  |
|  |  | 440, 452, 478 | 1.24E-05 |  |  |
|  |  | 499 | 1.13E-05 |  |  |
|  |  | 439, 494 | 1.13E-05 |  |  |
|  |  | 498, 501 | 1.13E-05 |  |  |
|  |  | 485 | 1.13E-05 |  |  |
|  |  | 478, 484, 501 | 1.13E-05 |  |  |
|  |  | 445, 452, 484 | 1.13E-05 |  |  |
|  |  | 444, 452, 490 | 1.13E-05 |  |  |
|  |  | 452, 475, 478 | 1.13E-05 |  |  |
|  |  | 463 | 1.01E-05 |  |  |
|  |  | 452, 475 | 1.01E-05 |  |  |
|  |  | 439, 501 | 1.01E-05 |  |  |
|  |  | 459, 477 | 1.01E-05 |  |  |
|  |  | 452, 481 | 1.01E-05 |  |  |
|  |  | 470, 484, 501 | 1.01E-05 |  |  |
|  |  | 452, 471, 478 | 1.01E-05 |  |  |
|  |  | 452, 478, 485 | 1.01E-05 |  |  |
|  |  | 452, 478, 508 | 1.01E-05 |  |  |
|  |  | 439, 455 | 9.01E-06 |  |  |
|  |  | 452, 493 | 9.01E-06 |  |  |
|  |  | 471, 484, 501 | 9.01E-06 |  |  |
|  |  | 475, 484, 501 | 9.01E-06 |  |  |
|  |  | 484, 490 | 9.01E-06 |  |  |
|  |  | 445, 452, 478 | 9.01E-06 |  |  |
|  |  | 455, 484, 501 | 7.89E-06 |  |  |
|  |  | 490, 494 | 7.89E-06 |  |  |
|  |  | 452, 463 | 7.89E-06 |  |  |
|  |  | 477, 490 | 7.89E-06 |  |  |
|  |  | 445, 484, 501 | 7.89E-06 |  |  |
|  |  | 452, 460, 478 | 7.89E-06 |  |  |
|  |  | 459, 501 | 6.76E-06 |  |  |
|  |  | 459, 490, 501 | 6.76E-06 |  |  |
|  |  | 466, 501 | 6.76E-06 |  |  |
|  |  | 455, 477 | 6.76E-06 |  |  |
|  |  | 452, 484, 499 | 6.76E-06 |  |  |
|  |  | 478, 483 | 6.76E-06 |  |  |
|  |  | 498 | 6.76E-06 |  |  |
|  |  | 477, 483 | 6.76E-06 |  |  |
|  |  | 452, 478, 481 | 6.76E-06 |  |  |
|  |  | 477, 499 | 6.76E-06 |  |  |
|  |  | 452, 470, 478 | 6.76E-06 |  |  |
|  |  | 440, 445, 446, 460, 477, 478, 484, 486, 490, 498, 501, 505 | 5.63E-06 |  |  |
|  |  | 439, 475 | 5.63E-06 |  |  |
|  |  | 453 | 5.63E-06 |  |  |
|  |  | 452, 499 | 5.63E-06 |  |  |
|  |  | 452, 471, 490 | 5.63E-06 |  |  |
|  |  | 484, 501, 508 | 5.63E-06 |  |  |
|  |  | 460, 501 | 5.63E-06 |  |  |
|  |  | 476, 477 | 5.63E-06 |  |  |
|  |  | 478, 508 | 5.63E-06 |  |  |
|  |  | 476, 484, 501 | 5.63E-06 |  |  |
|  |  | 493, 494, 501 | 5.63E-06 |  |  |
|  |  | 460, 484 | 5.63E-06 |  |  |
|  |  | 444, 484, 501 | 5.63E-06 |  |  |
|  |  | 452, 459 | 5.63E-06 |  |  |
|  |  | 440, 477, 478, 484, 493, 498, 501, 505 | 4.51E-06 |  |  |
|  |  | 484, 494, 501 | 4.51E-06 |  |  |
|  |  | 472 | 4.51E-06 |  |  |
|  |  | 440, 478 | 4.51E-06 |  |  |
|  |  | 455, 484 | 4.51E-06 |  |  |
|  |  | 477, 478 | 4.51E-06 |  |  |
|  |  | 452, 462 | 4.51E-06 |  |  |
|  |  | 445, 484 | 4.51E-06 |  |  |
|  |  | 450, 477, 494 | 4.51E-06 |  |  |
|  |  | 439, 444 | 4.51E-06 |  |  |
|  |  | 470, 477 | 4.51E-06 |  |  |
|  |  | 440, 484, 501 | 4.51E-06 |  |  |
|  |  | 452, 484, 501 | 4.51E-06 |  |  |
|  |  | 484, 485, 501 | 4.51E-06 |  |  |
|  |  | 452, 478, 482 | 4.51E-06 |  |  |
|  |  | 452, 478, 486 | 4.51E-06 |  |  |
|  |  | 440, 490 | 3.38E-06 |  |  |
|  |  | 440, 444, 452, 460, 477, 478, 484, 486, 498, 501, 505 | 3.38E-06 |  |  |
|  |  | 458 | 3.38E-06 |  |  |
|  |  | 477, 478, 484, 493, 498, 501, 505 | 3.38E-06 |  |  |
|  |  | 450, 484, 501 | 3.38E-06 |  |  |
|  |  | 452, 459, 478 | 3.38E-06 |  |  |
|  |  | 452, 460 | 3.38E-06 |  |  |
|  |  | 477, 508 | 3.38E-06 |  |  |
|  |  | 462, 501 | 3.38E-06 |  |  |
|  |  | 470, 490 | 3.38E-06 |  |  |
|  |  | 479, 484, 501 | 3.38E-06 |  |  |
|  |  | 476, 478 | 3.38E-06 |  |  |
|  |  | 475, 477 | 3.38E-06 |  |  |
|  |  | 475, 478 | 3.38E-06 |  |  |
|  |  | 494, 508 | 3.38E-06 |  |  |
|  |  | 478, 490 | 3.38E-06 |  |  |
|  |  | 444, 477 | 3.38E-06 |  |  |
|  |  | 477, 481 | 3.38E-06 |  |  |
|  |  | 490, 493 | 3.38E-06 |  |  |
|  |  | 453, 501 | 3.38E-06 |  |  |
|  |  | 462, 484, 501 | 3.38E-06 |  |  |
|  |  | 484, 493, 501 | 3.38E-06 |  |  |
|  |  | 490, 501, 503 | 3.38E-06 |  |  |
|  |  | 440, 494 | 3.38E-06 |  |  |
|  |  | 458, 484, 501 | 3.38E-06 |  |  |
|  |  | 452, 490, 501 | 3.38E-06 |  |  |
|  |  | 452, 478, 496 | 3.38E-06 |  |  |
|  |  | 481, 484, 501 | 3.38E-06 |  |  |
|  |  | 452, 478, 498 | 3.38E-06 |  |  |
|  |  | 452, 478, 507 | 3.38E-06 |  |  |
|  |  | 478, 479 | 2.25E-06 |  |  |
|  |  | 452, 477, 478, 484, 493, 496, 498, 501, 505 | 2.25E-06 |  |  |
|  |  | 452, 472 | 2.25E-06 |  |  |
|  |  | 440, 452, 477, 478, 484, 493, 498, 501, 505 | 2.25E-06 |  |  |
|  |  | 452, 508 | 2.25E-06 |  |  |
|  |  | 483, 494, 501 | 2.25E-06 |  |  |
|  |  | 459, 484 | 2.25E-06 |  |  |
|  |  | 468, 477 | 2.25E-06 |  |  |
|  |  | 462 | 2.25E-06 |  |  |
|  |  | 470, 478 | 2.25E-06 |  |  |
|  |  | 494, 499 | 2.25E-06 |  |  |
|  |  | 446, 478 | 2.25E-06 |  |  |
|  |  | 452, 485 | 2.25E-06 |  |  |
|  |  | 494, 496, 501 | 2.25E-06 |  |  |
|  |  | 439, 446 | 2.25E-06 |  |  |
|  |  | 485, 494, 501 | 2.25E-06 |  |  |
|  |  | 490, 494, 501 | 2.25E-06 |  |  |
|  |  | 452, 507 | 2.25E-06 |  |  |
|  |  | 475, 484 | 2.25E-06 |  |  |
|  |  | 453, 478 | 2.25E-06 |  |  |
|  |  | 452, 477, 484 | 2.25E-06 |  |  |
|  |  | 446, 452, 501 | 2.25E-06 |  |  |
|  |  | 483, 494 | 2.25E-06 |  |  |
|  |  | 478, 498 | 2.25E-06 |  |  |
|  |  | 446, 484, 501 | 2.25E-06 |  |  |
|  |  | 484, 486, 501 | 2.25E-06 |  |  |
|  |  | 452, 478, 483, 484 | 2.25E-06 |  |  |
|  |  | 439, 440, 452, 493, 501 | 2.25E-06 |  |  |
|  |  | 452, 453, 478 | 2.25E-06 |  |  |
|  |  | 460, 484, 501 | 2.25E-06 |  |  |
|  |  | 453, 484 | 2.25E-06 |  |  |
|  |  | 440, 444, 482, 484, 485, 486, 496, 498, 501 | 2.25E-06 |  |  |
|  |  | 446, 455, 501 | 2.25E-06 |  |  |
|  |  | 463, 484, 501 | 2.25E-06 |  |  |
|  |  | 475, 478, 501 | 2.25E-06 |  |  |
|  |  | 452, 468, 478 | 2.25E-06 |  |  |
|  |  | 440, 477, 478, 484, 493 | 1.13E-06 |  |  |
|  |  | 479, 490, 501 | 1.13E-06 |  |  |
|  |  | 460, 477, 478, 484, 486, 490, 498, 501, 505 | 1.13E-06 |  |  |
|  |  | 446, 453, 484 | 1.13E-06 |  |  |
|  |  | 440, 444, 445, 452, 477, 478, 484, 486, 498, 501, 505 | 1.13E-06 |  |  |
|  |  | 440, 446, 477, 478, 484 | 1.13E-06 |  |  |
|  |  | 493, 496, 498, 501, 505 | 1.13E-06 |  |  |
|  |  | 440, 445, 446, 460, 477, 478, 484, 490, 498, 501, 505 | 1.13E-06 |  |  |
|  |  | 446, 494 | 1.13E-06 |  |  |
|  |  | 484, 493, 496, 498, 501, 505 | 1.13E-06 |  |  |
|  |  | 440, 446, 505 | 1.13E-06 |  |  |
|  |  | 452, 453, 455 | 1.13E-06 |  |  |
|  |  | 477, 479 | 1.13E-06 |  |  |
|  |  | 452, 482 | 1.13E-06 |  |  |
|  |  | 439, 496 | 1.13E-06 |  |  |
|  |  | 458, 478 | 1.13E-06 |  |  |
|  |  | 459, 478 | 1.13E-06 |  |  |
|  |  | 482 | 1.13E-06 |  |  |
|  |  | 463, 494 | 1.13E-06 |  |  |
|  |  | 484, 499 | 1.13E-06 |  |  |
|  |  | 439, 490 | 1.13E-06 |  |  |
|  |  | 439, 450 | 1.13E-06 |  |  |
|  |  | 476, 494 | 1.13E-06 |  |  |
|  |  | 440, 452, 477, 478, 484, 486, 498, 501, 505 | 1.13E-06 |  |  |
|  |  | 496, 501, 505 | 1.13E-06 |  |  |
|  |  | 446, 477, 478, 484, 493, 496, 498, 501, 505 | 1.13E-06 |  |  |
|  |  | 440, 477 | 1.13E-06 |  |  |
|  |  | 475, 477, 501 | 1.13E-06 |  |  |
|  |  | 439, 471 | 1.13E-06 |  |  |
|  |  | 493, 494 | 1.13E-06 |  |  |
|  |  | 507 | 1.13E-06 |  |  |
|  |  | 458, 471, 501 | 1.13E-06 |  |  |
|  |  | 484, 507 | 1.13E-06 |  |  |
|  |  | 452, 496 | 1.13E-06 |  |  |
|  |  | 453, 490 | 1.13E-06 |  |  |
|  |  | 485, 494 | 1.13E-06 |  |  |
|  |  | 450, 484 | 1.13E-06 |  |  |
|  |  | 479, 485 | 1.13E-06 |  |  |
|  |  | 440, 484, 485, 486, 496, 501 | 1.13E-06 |  |  |
|  |  | 458, 484 | 1.13E-06 |  |  |
|  |  | 479, 494 | 1.13E-06 |  |  |
|  |  | 484, 494, 499 | 1.13E-06 |  |  |
|  |  | 478, 493 | 1.13E-06 |  |  |
|  |  | 478, 483, 501 | 1.13E-06 |  |  |
|  |  | 452, 498 | 1.13E-06 |  |  |
|  |  | 440, 452, 460 | 1.13E-06 |  |  |
|  |  | 455, 463, 501 | 1.13E-06 |  |  |
|  |  | 475, 493, 501 | 1.13E-06 |  |  |
|  |  | 445, 478 | 1.13E-06 |  |  |
|  |  | 455, 478 | 1.13E-06 |  |  |
|  |  | 452, 490, 503 | 1.13E-06 |  |  |
|  |  | 452, 494, 501 | 1.13E-06 |  |  |
|  |  | 446, 452, 453 | 1.13E-06 |  |  |
|  |  | 452, 458 | 1.13E-06 |  |  |
|  |  | 440, 446, 484 | 1.13E-06 |  |  |
|  |  | 472, 477 | 1.13E-06 |  |  |
|  |  | 460, 484, 493 | 1.13E-06 |  |  |
|  |  | 446, 452, 483 | 1.13E-06 |  |  |
|  |  | 478, 507 | 1.13E-06 |  |  |
|  |  | 453, 484, 501 | 1.13E-06 |  |  |
|  |  | 446, 452, 484 | 1.13E-06 |  |  |
|  |  | 477, 507 | 1.13E-06 |  |  |
|  |  | 446, 452, 478, 499 | 1.13E-06 |  |  |
|  |  | 477, 478, 484 | 1.13E-06 |  |  |
|  |  | 453, 494, 501 | 1.13E-06 |  |  |
|  |  | 468, 486, 501 | 1.13E-06 |  |  |
|  |  | 445, 452 | 1.13E-06 |  |  |
|  |  | 440, 484, 486 | 1.13E-06 |  |  |
|  |  | 452, 468 | 1.13E-06 |  |  |
|  |  | 452, 460, 490 | 1.13E-06 |  |  |
|  |  | 479, 484 | 1.13E-06 |  |  |
|  |  | 484, 508 | 1.13E-06 |  |  |
|  |  | 484, 496, 501 | 1.13E-06 |  |  |
|  |  | 444, 460, 501 | 1.13E-06 |  |  |
|  |  | 445, 477 | 1.13E-06 |  |  |
|  |  | 452, 485, 508 | 1.13E-06 |  |  |
|  |  | 440, 484, 494 | 1.13E-06 |  |  |
|  |  | 452, 471 | 1.13E-06 |  |  |
|  |  | 478, 482 | 1.13E-06 |  |  |
|  |  | 440, 455, 484 | 1.13E-06 |  |  |
|  |  | 470, 494 | 1.13E-06 |  |  |
|  |  | 477, 490, 501 | 1.13E-06 |  |  |
|  |  | 439, 455, 494 | 1.13E-06 |  |  |
|  |  | 446, 452, 455 | 1.13E-06 |  |  |
|  |  | 440, 476, 484 | 1.13E-06 |  |  |
|  |  | 439, 440 | 1.13E-06 |  |  |
|  |  | 475, 490, 501 | 1.13E-06 |  |  |
|  |  | 484, 501, 503 | 1.13E-06 |  |  |
|  |  | 452, 466, 478 | 1.13E-06 |  |  |
|  |  | 452, 471, 478, 494 | 1.13E-06 |  |  |
|  |  | 484, 501, 507 | 1.13E-06 |  |  |
|  |  | 450, 452, 478, 490 | 1.13E-06 |  |  |
|  |  | 439, 452, 490 | 1.13E-06 |  |  |
|  |  | 446, 484, 494 | 1.13E-06 |  |  |
|  |  | 452, 472, 478 | 1.13E-06 |  |  |
| **LightGBM** | | | | | |
| **①** | | **②** | | **③** | |
| **mutation combination** | **frequency** | **mutation combination** | **frequency** | **mutation combination** | **frequency** |
| 501 | 0.395432604 | 501 | 0.624725199 | 501 | 0.395432604 |
| 477 | 0.361277701 | 452, 478 | 0.166435858 | 477 | 0.361277701 |
| 439 | 0.089830323 | 452 | 0.061709893 | 439 | 0.089830323 |
| 452 | 0.078420976 | 484, 501 | 0.038285712 | 452 | 0.078420976 |
| 484, 501 | 0.018905873 | 484 | 0.036025326 | 484, 501 | 0.018905873 |
| 484 | 0.013585168 | 477 | 0.025517799 | 484 | 0.013585168 |
| N/A | 0.013274336 | 478 | 0.015300989 | N/A | 0.013274336 |
| 494 | 0.006106926 | 494, 501 | 0.005176577 | 494 | 0.006106926 |
| 478 | 0.005722958 | 494 | 0.004427247 | 478 | 0.005722958 |
| 494, 501 | 0.003931105 | 439 | 0.004405837 | 494, 501 | 0.003931105 |
| 452, 478 | 0.003857968 | 490, 501 | 0.002187143 | 452, 478 | 0.003857968 |
| 446 | 0.002102684 | 452, 490 | 0.001551621 | 446 | 0.002102684 |
| 479 | 0.001316463 | 452, 484 | 0.001175266 | 479 | 0.001316463 |
| 483 | 0.001115337 | 452, 501 | 0.001033287 | 483 | 0.001115337 |
| 440, 446, 477, 478, 484, 493, 496, 498, 501, 505 | 0.000859358 | 440, 484 | 0.000747077 | 440, 446, 477, 478, 484, 493, 496, 498, 501, 505 | 0.000859358 |
| 490 | 0.000804505 | 452, 477 | 0.00053749 | 490 | 0.000804505 |
| 476 | 0.000475389 | 440, 501 | 0.00053749 | 476 | 0.000475389 |
| 477, 478, 484, 493, 496, 498, 501, 505 | 0.000365684 | 440 | 0.000528475 | 477, 478, 484, 493, 496, 498, 501, 505 | 0.000365684 |
| 446, 477 | 0.000274263 | 478, 501 | 0.000525095 | 446, 477 | 0.000274263 |
| 440, 445, 446, 460, 477, 478, 484, 486, 490, 498, 501, 505 | 0.000219411 | 450 | 0.000475515 | 440, 445, 446, 460, 477, 478, 484, 486, 490, 498, 501, 505 | 0.000219411 |
| 440, 452, 477, 478, 484, 486, 498, 501, 505 | 0.000164558 | 455, 501 | 0.000450725 | 440, 452, 477, 478, 484, 486, 498, 501, 505 | 0.000164558 |
| 477, 494 | 0.000127989 | 477, 501 | 0.000446218 | 477, 494 | 0.000127989 |
| 452, 477, 486 | 0.000127989 | 446, 452, 478 | 0.000442837 | 452, 477, 486 | 0.000127989 |
| 477, 501 | 9.1421E-05 | 496, 501 | 0.000367341 | 477, 501 | 9.1421E-05 |
| 452, 501 | 9.1421E-05 | 452, 503 | 0.00033241 | 452, 501 | 9.1421E-05 |
| 439, 478 | 9.1421E-05 | 475, 501 | 0.000274942 | 439, 478 | 9.1421E-05 |
| 477, 483 | 9.1421E-05 | 471, 501 | 0.000265928 | 477, 483 | 9.1421E-05 |
| 460, 477, 478, 484, 486, 490, 498, 501, 505 | 9.1421E-05 | 439, 484 | 0.000252406 | 460, 477, 478, 484, 486, 490, 498, 501, 505 | 9.1421E-05 |
| 477, 484 | 9.1421E-05 | 493, 501 | 0.000241138 | 477, 484 | 9.1421E-05 |
| 478, 501 | 9.1421E-05 | 470 | 0.000226489 | 478, 501 | 9.1421E-05 |
| 478, 484 | 7.31368E-05 | 483, 501 | 0.000221982 | 478, 484 | 7.31368E-05 |
| 490, 501 | 7.31368E-05 | 477, 484 | 0.000211841 | 490, 501 | 7.31368E-05 |
| 446, 452, 478 | 5.48526E-05 | 459 | 0.000210714 | 446, 452, 478 | 5.48526E-05 |
| 485 | 5.48526E-05 | 446, 501 | 0.000192685 | 485 | 5.48526E-05 |
| 445, 460, 505 | 5.48526E-05 | 479, 501 | 0.000182544 | 445, 460, 505 | 5.48526E-05 |
| 476, 501 | 5.48526E-05 | 452, 478, 484 | 0.000181417 | 476, 501 | 5.48526E-05 |
| 479, 490, 501 | 5.48526E-05 | 479 | 0.000179163 | 479, 490, 501 | 5.48526E-05 |
| 446, 501 | 5.48526E-05 | 484, 494 | 0.000161134 | 446, 501 | 5.48526E-05 |
| 493, 496, 498, 501, 505 | 3.65684E-05 | 490 | 0.000160007 | 493, 496, 498, 501, 505 | 3.65684E-05 |
| 483, 484 | 3.65684E-05 | 440, 452 | 0.00013071 | 483, 484 | 3.65684E-05 |
| 452, 478, 490 | 3.65684E-05 | 452, 477, 478 | 0.000129583 | 452, 478, 490 | 3.65684E-05 |
| 477, 505 | 3.65684E-05 | 444, 452 | 0.000116062 | 477, 505 | 3.65684E-05 |
| 439, 494 | 3.65684E-05 | 444, 452, 478 | 0.000113808 | 439, 494 | 3.65684E-05 |
| 483, 501 | 3.65684E-05 | 476, 501 | 0.000111554 | 483, 501 | 3.65684E-05 |
| 452, 476, 478 | 1.82842E-05 | 444, 501 | 0.000108174 | 452, 476, 478 | 1.82842E-05 |
| 477, 486, 490 | 1.82842E-05 | 485, 501 | 9.91595E-05 | 477, 486, 490 | 1.82842E-05 |
| 439, 446 | 1.82842E-05 | N/A | 9.23986E-05 | 439, 446 | 1.82842E-05 |
| 446, 477, 478, 484, 493, 496, 498, 501 | 1.82842E-05 | 508 | 9.0145E-05 | 446, 477, 478, 484, 493, 496, 498, 501 | 1.82842E-05 |
| 476, 484, 501 | 1.82842E-05 | 452, 478, 479 | 8.78914E-05 | 476, 484, 501 | 1.82842E-05 |
| 446, 478 | 1.82842E-05 | 452, 494 | 8.22573E-05 | 446, 478 | 1.82842E-05 |
| 446, 493, 496, 498, 505 | 1.82842E-05 | 446 | 8.00037E-05 | 446, 493, 496, 498, 505 | 1.82842E-05 |
| 446, 452 | 1.82842E-05 | 470, 501 | 8.00037E-05 | 446, 452 | 1.82842E-05 |
| 446, 477, 478, 484 | 1.82842E-05 | 452, 478, 501 | 6.53551E-05 | 446, 477, 478, 484 | 1.82842E-05 |
| 440, 445, 446, 450, 452, 455, 460, 477, 478, 481, 484, 486, 498, 501, 505 | 1.82842E-05 | 450, 501 | 6.19747E-05 | 440, 445, 446, 450, 452, 455, 460, 477, 478, 481, 484, 486, 498, 501, 505 | 1.82842E-05 |
| 446, 493, 496, 498, 501 | 1.82842E-05 | 452, 478, 493 | 6.19747E-05 | 446, 493, 496, 498, 501 | 1.82842E-05 |
| 479, 501 | 1.82842E-05 | 484, 490, 501 | 6.08479E-05 | 479, 501 | 1.82842E-05 |
| 446, 477, 484, 493, 496, 498, 501, 505 | 1.82842E-05 | 478, 484 | 5.4087E-05 | 446, 477, 484, 493, 496, 498, 501, 505 | 1.82842E-05 |
| 452, 490 | 1.82842E-05 | 458, 468, 501 | 5.18334E-05 | 452, 490 | 1.82842E-05 |
| 477, 478 | 1.82842E-05 | 440, 446, 477, 478, 484, 493, 496, 498, 501, 505 | 4.95798E-05 | 477, 478 | 1.82842E-05 |
| 501, 505 | 1.82842E-05 | 452, 483 | 4.84529E-05 | 501, 505 | 1.82842E-05 |
| 452, 484 | 1.82842E-05 | 501, 503 | 4.84529E-05 | 452, 484 | 1.82842E-05 |
| 478, 479 | 1.82842E-05 | 483, 484 | 4.61993E-05 | 478, 479 | 1.82842E-05 |
| 476, 478, 501 | 1.82842E-05 | 452, 478, 483 | 4.61993E-05 | 476, 478, 501 | 1.82842E-05 |
| 485, 501 | 1.82842E-05 | 483 | 4.39457E-05 | 485, 501 | 1.82842E-05 |
|  |  | 445, 501 | 4.39457E-05 |  |  |
|  |  | 481, 501 | 4.28189E-05 |  |  |
|  |  | 444 | 4.16921E-05 |  |  |
|  |  | 475 | 4.05653E-05 |  |  |
|  |  | 446, 452 | 4.05653E-05 |  |  |
|  |  | 476 | 3.94384E-05 |  |  |
|  |  | 452, 478, 494 | 3.83116E-05 |  |  |
|  |  | 452, 478, 503 | 3.71848E-05 |  |  |
|  |  | 440, 446, 477, 478, 484, 493 | 3.49312E-05 |  |  |
|  |  | 452, 455 | 3.38044E-05 |  |  |
|  |  | 478, 494 | 3.38044E-05 |  |  |
|  |  | 471 | 3.26776E-05 |  |  |
|  |  | 501, 508 | 3.15508E-05 |  |  |
|  |  | 452, 476, 478 | 3.04239E-05 |  |  |
|  |  | 503 | 2.92971E-05 |  |  |
|  |  | 501, 505 | 2.92971E-05 |  |  |
|  |  | 468 | 2.92971E-05 |  |  |
|  |  | 446, 477 | 2.92971E-05 |  |  |
|  |  | 493 | 2.70435E-05 |  |  |
|  |  | 455 | 2.70435E-05 |  |  |
|  |  | 452, 476 | 2.59167E-05 |  |  |
|  |  | 452, 455, 478 | 2.47899E-05 |  |  |
|  |  | 458, 468, 490, 501 | 2.47899E-05 |  |  |
|  |  | 477, 494 | 2.36631E-05 |  |  |
|  |  | 486, 501 | 2.36631E-05 |  |  |
|  |  | 452, 479 | 2.36631E-05 |  |  |
|  |  | 452, 455, 501 | 1.91558E-05 |  |  |
|  |  | 446, 484 | 1.91558E-05 |  |  |
|  |  | 452, 453 | 1.69022E-05 |  |  |
|  |  | 477, 478, 484, 493, 496, 498, 501, 505 | 1.57754E-05 |  |  |
|  |  | 481 | 1.57754E-05 |  |  |
|  |  | 460 | 1.57754E-05 |  |  |
|  |  | 450, 477 | 1.57754E-05 |  |  |
|  |  | 483, 484, 501 | 1.57754E-05 |  |  |
|  |  | 476, 484 | 1.35218E-05 |  |  |
|  |  | 452, 478, 490 | 1.35218E-05 |  |  |
|  |  | 445 | 1.23949E-05 |  |  |
|  |  | 452, 470 | 1.23949E-05 |  |  |
|  |  | 477, 484, 501 | 1.23949E-05 |  |  |
|  |  | 440, 452, 478 | 1.23949E-05 |  |  |
|  |  | 439, 494 | 1.12681E-05 |  |  |
|  |  | 498, 501 | 1.12681E-05 |  |  |
|  |  | 485 | 1.12681E-05 |  |  |
|  |  | 478, 484, 501 | 1.12681E-05 |  |  |
|  |  | 445, 452, 484 | 1.12681E-05 |  |  |
|  |  | 444, 452, 490 | 1.12681E-05 |  |  |
|  |  | 452, 475, 478 | 1.12681E-05 |  |  |
|  |  | 452, 475 | 1.01413E-05 |  |  |
|  |  | 439, 501 | 1.01413E-05 |  |  |
|  |  | 459, 477 | 1.01413E-05 |  |  |
|  |  | 452, 481 | 1.01413E-05 |  |  |
|  |  | 470, 484, 501 | 1.01413E-05 |  |  |
|  |  | 452, 471, 478 | 1.01413E-05 |  |  |
|  |  | 452, 478, 485 | 1.01413E-05 |  |  |
|  |  | 452, 478, 508 | 1.01413E-05 |  |  |
|  |  | 439, 455 | 9.0145E-06 |  |  |
|  |  | 452, 493 | 9.0145E-06 |  |  |
|  |  | 471, 484, 501 | 9.0145E-06 |  |  |
|  |  | 475, 484, 501 | 9.0145E-06 |  |  |
|  |  | 484, 490 | 9.0145E-06 |  |  |
|  |  | 445, 452, 478 | 9.0145E-06 |  |  |
|  |  | 455, 484, 501 | 7.88769E-06 |  |  |
|  |  | 490, 494 | 7.88769E-06 |  |  |
|  |  | 477, 490 | 7.88769E-06 |  |  |
|  |  | 445, 484, 501 | 7.88769E-06 |  |  |
|  |  | 452, 460, 478 | 7.88769E-06 |  |  |
|  |  | 459, 501 | 6.76088E-06 |  |  |
|  |  | 459, 490, 501 | 6.76088E-06 |  |  |
|  |  | 455, 477 | 6.76088E-06 |  |  |
|  |  | 478, 483 | 6.76088E-06 |  |  |
|  |  | 498 | 6.76088E-06 |  |  |
|  |  | 477, 483 | 6.76088E-06 |  |  |
|  |  | 452, 478, 481 | 6.76088E-06 |  |  |
|  |  | 452, 470, 478 | 6.76088E-06 |  |  |
|  |  | 440, 445, 446, 460, 477, 478, 484, 486, 490, 498, 501, 505 | 5.63406E-06 |  |  |
|  |  | 439, 475 | 5.63406E-06 |  |  |
|  |  | 453 | 5.63406E-06 |  |  |
|  |  | 452, 471, 490 | 5.63406E-06 |  |  |
|  |  | 484, 501, 508 | 5.63406E-06 |  |  |
|  |  | 460, 501 | 5.63406E-06 |  |  |
|  |  | 476, 477 | 5.63406E-06 |  |  |
|  |  | 478, 508 | 5.63406E-06 |  |  |
|  |  | 476, 484, 501 | 5.63406E-06 |  |  |
|  |  | 493, 494, 501 | 5.63406E-06 |  |  |
|  |  | 460, 484 | 5.63406E-06 |  |  |
|  |  | 444, 484, 501 | 5.63406E-06 |  |  |
|  |  | 452, 459 | 5.63406E-06 |  |  |
|  |  | 440, 477, 478, 484, 493, 498, 501, 505 | 4.50725E-06 |  |  |
|  |  | 484, 494, 501 | 4.50725E-06 |  |  |
|  |  | 440, 478, 499 | 4.50725E-06 |  |  |
|  |  | 455, 484 | 4.50725E-06 |  |  |
|  |  | 477, 478 | 4.50725E-06 |  |  |
|  |  | 445, 484 | 4.50725E-06 |  |  |
|  |  | 450, 477, 494 | 4.50725E-06 |  |  |
|  |  | 439, 444 | 4.50725E-06 |  |  |
|  |  | 470, 477 | 4.50725E-06 |  |  |
|  |  | 440, 484, 501 | 4.50725E-06 |  |  |
|  |  | 452, 484, 501 | 4.50725E-06 |  |  |
|  |  | 484, 485, 501 | 4.50725E-06 |  |  |
|  |  | 452, 478, 486 | 4.50725E-06 |  |  |
|  |  | 440, 490 | 3.38044E-06 |  |  |
|  |  | 440, 444, 452, 460, 477, 478, 484, 486, 498, 501, 505 | 3.38044E-06 |  |  |
|  |  | 477, 478, 484, 493, 498, 501, 505 | 3.38044E-06 |  |  |
|  |  | 450, 484, 501 | 3.38044E-06 |  |  |
|  |  | 452, 459, 478 | 3.38044E-06 |  |  |
|  |  | 452, 460 | 3.38044E-06 |  |  |
|  |  | 477, 508 | 3.38044E-06 |  |  |
|  |  | 470, 490 | 3.38044E-06 |  |  |
|  |  | 479, 484, 501 | 3.38044E-06 |  |  |
|  |  | 476, 478 | 3.38044E-06 |  |  |
|  |  | 475, 477 | 3.38044E-06 |  |  |
|  |  | 475, 478 | 3.38044E-06 |  |  |
|  |  | 494, 508 | 3.38044E-06 |  |  |
|  |  | 478, 490 | 3.38044E-06 |  |  |
|  |  | 444, 477 | 3.38044E-06 |  |  |
|  |  | 477, 481 | 3.38044E-06 |  |  |
|  |  | 490, 493 | 3.38044E-06 |  |  |
|  |  | 453, 501 | 3.38044E-06 |  |  |
|  |  | 484, 493, 501 | 3.38044E-06 |  |  |
|  |  | 490, 501, 503 | 3.38044E-06 |  |  |
|  |  | 440, 494 | 3.38044E-06 |  |  |
|  |  | 452, 490, 501 | 3.38044E-06 |  |  |
|  |  | 452, 478, 496 | 3.38044E-06 |  |  |
|  |  | 481, 484, 501 | 3.38044E-06 |  |  |
|  |  | 452, 478, 498 | 3.38044E-06 |  |  |
|  |  | 478, 479 | 2.25363E-06 |  |  |
|  |  | 452, 477, 478, 484, 493, 496, 498, 501, 505 | 2.25363E-06 |  |  |
|  |  | 440, 452, 477, 478, 484, 493, 498, 501, 505 | 2.25363E-06 |  |  |
|  |  | 452, 506, 508 | 2.25363E-06 |  |  |
|  |  | 483, 494, 501 | 2.25363E-06 |  |  |
|  |  | 459, 484 | 2.25363E-06 |  |  |
|  |  | 458, 468, 477 | 2.25363E-06 |  |  |
|  |  | 470, 478 | 2.25363E-06 |  |  |
|  |  | 446, 478 | 2.25363E-06 |  |  |
|  |  | 452, 485 | 2.25363E-06 |  |  |
|  |  | 494, 496, 501 | 2.25363E-06 |  |  |
|  |  | 439, 446 | 2.25363E-06 |  |  |
|  |  | 485, 494, 501 | 2.25363E-06 |  |  |
|  |  | 490, 494, 501 | 2.25363E-06 |  |  |
|  |  | 475, 484 | 2.25363E-06 |  |  |
|  |  | 453, 478 | 2.25363E-06 |  |  |
|  |  | 452, 477, 484 | 2.25363E-06 |  |  |
|  |  | 446, 452, 501 | 2.25363E-06 |  |  |
|  |  | 483, 494 | 2.25363E-06 |  |  |
|  |  | 478, 498 | 2.25363E-06 |  |  |
|  |  | 446, 484, 501 | 2.25363E-06 |  |  |
|  |  | 484, 486, 501 | 2.25363E-06 |  |  |
|  |  | 452, 478, 483, 484 | 2.25363E-06 |  |  |
|  |  | 439, 440, 452, 493, 501 | 2.25363E-06 |  |  |
|  |  | 452, 453, 478 | 2.25363E-06 |  |  |
|  |  | 460, 484, 501 | 2.25363E-06 |  |  |
|  |  | 453, 484 | 2.25363E-06 |  |  |
|  |  | 440, 444, 484, 485, 486, 496, 498, 501 | 2.25363E-06 |  |  |
|  |  | 446, 455, 501 | 2.25363E-06 |  |  |
|  |  | 475, 478, 501 | 2.25363E-06 |  |  |
|  |  | 452, 458, 468, 478 | 2.25363E-06 |  |  |
|  |  | 440, 477, 478, 484, 493 | 1.12681E-06 |  |  |
|  |  | 479, 490, 501 | 1.12681E-06 |  |  |
|  |  | 460, 477, 478, 484, 486, 490, 498, 501, 505 | 1.12681E-06 |  |  |
|  |  | 446, 453, 484 | 1.12681E-06 |  |  |
|  |  | 440, 444, 445, 452, 477, 478, 484, 486, 498, 501, 505 | 1.12681E-06 |  |  |
|  |  | 440, 446, 477, 478, 484 | 1.12681E-06 |  |  |
|  |  | 493, 496, 498, 501, 505 | 1.12681E-06 |  |  |
|  |  | 440, 445, 446, 460, 477, 478, 484, 490, 498, 501, 505 | 1.12681E-06 |  |  |
|  |  | 446, 494 | 1.12681E-06 |  |  |
|  |  | 484, 493, 496, 498, 501, 505 | 1.12681E-06 |  |  |
|  |  | 440, 446, 505 | 1.12681E-06 |  |  |
|  |  | 452, 453, 455 | 1.12681E-06 |  |  |
|  |  | 477, 479 | 1.12681E-06 |  |  |
|  |  | 439, 496 | 1.12681E-06 |  |  |
|  |  | 459, 478 | 1.12681E-06 |  |  |
|  |  | 439, 490 | 1.12681E-06 |  |  |
|  |  | 439, 450 | 1.12681E-06 |  |  |
|  |  | 476, 494 | 1.12681E-06 |  |  |
|  |  | 440, 452, 477, 478, 484, 486, 498, 501, 505 | 1.12681E-06 |  |  |
|  |  | 496, 501, 505 | 1.12681E-06 |  |  |
|  |  | 446, 477, 478, 484, 493, 496, 498, 501, 505 | 1.12681E-06 |  |  |
|  |  | 440, 477 | 1.12681E-06 |  |  |
|  |  | 475, 477, 501 | 1.12681E-06 |  |  |
|  |  | 439, 471 | 1.12681E-06 |  |  |
|  |  | 493, 494 | 1.12681E-06 |  |  |
|  |  | 452, 496 | 1.12681E-06 |  |  |
|  |  | 453, 490 | 1.12681E-06 |  |  |
|  |  | 485, 494 | 1.12681E-06 |  |  |
|  |  | 450, 484 | 1.12681E-06 |  |  |
|  |  | 479, 485 | 1.12681E-06 |  |  |
|  |  | 440, 484, 485, 486, 496, 501 | 1.12681E-06 |  |  |
|  |  | 479, 494 | 1.12681E-06 |  |  |
|  |  | 478, 493 | 1.12681E-06 |  |  |
|  |  | 478, 483, 501 | 1.12681E-06 |  |  |
|  |  | 452, 498 | 1.12681E-06 |  |  |
|  |  | 440, 452, 460 | 1.12681E-06 |  |  |
|  |  | 475, 493, 501 | 1.12681E-06 |  |  |
|  |  | 445, 478 | 1.12681E-06 |  |  |
|  |  | 455, 478 | 1.12681E-06 |  |  |
|  |  | 452, 490, 503 | 1.12681E-06 |  |  |
|  |  | 452, 494, 501 | 1.12681E-06 |  |  |
|  |  | 446, 452, 453 | 1.12681E-06 |  |  |
|  |  | 440, 446, 484 | 1.12681E-06 |  |  |
|  |  | 460, 484, 493 | 1.12681E-06 |  |  |
|  |  | 446, 452, 483 | 1.12681E-06 |  |  |
|  |  | 453, 484, 501 | 1.12681E-06 |  |  |
|  |  | 446, 452, 484 | 1.12681E-06 |  |  |
|  |  | 477, 478, 484 | 1.12681E-06 |  |  |
|  |  | 453, 494, 501 | 1.12681E-06 |  |  |
|  |  | 458, 468, 486, 501 | 1.12681E-06 |  |  |
|  |  | 445, 452 | 1.12681E-06 |  |  |
|  |  | 440, 484, 486 | 1.12681E-06 |  |  |
|  |  | 452, 458, 468 | 1.12681E-06 |  |  |
|  |  | 452, 460, 490 | 1.12681E-06 |  |  |
|  |  | 479, 484 | 1.12681E-06 |  |  |
|  |  | 484, 508 | 1.12681E-06 |  |  |
|  |  | 484, 496, 501 | 1.12681E-06 |  |  |
|  |  | 444, 460, 501 | 1.12681E-06 |  |  |
|  |  | 445, 477 | 1.12681E-06 |  |  |
|  |  | 452, 485, 506, 508 | 1.12681E-06 |  |  |
|  |  | 440, 484, 494 | 1.12681E-06 |  |  |
|  |  | 452, 471 | 1.12681E-06 |  |  |
|  |  | 440, 455, 484 | 1.12681E-06 |  |  |
|  |  | 470, 494 | 1.12681E-06 |  |  |
|  |  | 477, 490, 501 | 1.12681E-06 |  |  |
|  |  | 439, 455, 494 | 1.12681E-06 |  |  |
|  |  | 446, 452, 455 | 1.12681E-06 |  |  |
|  |  | 440, 476, 484 | 1.12681E-06 |  |  |
|  |  | 439, 440 | 1.12681E-06 |  |  |
|  |  | 475, 490, 501 | 1.12681E-06 |  |  |
|  |  | 484, 501, 503 | 1.12681E-06 |  |  |
|  |  | 452, 471, 478, 494 | 1.12681E-06 |  |  |
|  |  | 450, 452, 478, 490 | 1.12681E-06 |  |  |
|  |  | 439, 452, 490 | 1.12681E-06 |  |  |
|  |  | 446, 484, 494 | 1.12681E-06 |  |  |
| **Random Forest** | | | | | |
| **①** | | **②** | | **③** | |
| **mutation combination** | **frequency** | **mutation combination** | **frequency** | **mutation combination** | **frequency** |
| 501 | 0.399729394 | 501 | 0.624531387 | 501 | 0.399729394 |
| 477 | 0.362137058 | 452, 478 | 0.166950811 | 477 | 0.362137058 |
| 439 | 0.089940028 | 452 | 0.062308231 | 439 | 0.089940028 |
| 452 | 0.078420976 | 484, 501 | 0.038460368 | 452 | 0.078420976 |
| 484, 501 | 0.018924157 | 484 | 0.036247308 | 484, 501 | 0.018924157 |
| 484 | 0.013603452 | 477 | 0.025549349 | 484 | 0.013603452 |
| 494 | 0.006106926 | 478 | 0.015334793 | 494 | 0.006106926 |
| 478 | 0.005942368 | 494, 501 | 0.005193479 | 478 | 0.005942368 |
| 452, 478 | 0.003967674 | 439 | 0.004669511 | 452, 478 | 0.003967674 |
| N/A | 0.002541505 | 494 | 0.004484714 | N/A | 0.002541505 |
| 446 | 0.002120968 | 490, 501 | 0.002222074 | 446 | 0.002120968 |
| 475 | 0.001883274 | 452, 490 | 0.001570777 | 475 | 0.001883274 |
| 459 | 0.001773568 | 452, 484 | 0.001187661 | 459 | 0.001773568 |
| 479 | 0.001316463 | 452, 501 | 0.001035541 | 479 | 0.001316463 |
| 483 | 0.001115337 | 449, 501 | 0.000768486 | 483 | 0.001115337 |
| 460 | 0.001023916 | 440, 484 | 0.000752711 | 460 | 0.001023916 |
| 440, 446, 477, 478, 484, 493, 496, 498, 501, 505 | 0.000895926 | 452, 477 | 0.00053749 | 440, 446, 477, 478, 484, 493, 496, 498, 501, 505 | 0.000895926 |
| 508 | 0.000859358 | 440, 501 | 0.00053749 | 508 | 0.000859358 |
| 490 | 0.000822789 | 440 | 0.000528475 | 490 | 0.000822789 |
| 444 | 0.000749653 | 478, 501 | 0.000528475 | 444 | 0.000749653 |
| 481 | 0.000639947 | 450 | 0.000475515 | 481 | 0.000639947 |
| 470 | 0.000493674 | 455, 501 | 0.000450725 | 470 | 0.000493674 |
| 476 | 0.000475389 | 477, 501 | 0.000447345 | 476 | 0.000475389 |
| 471 | 0.000457105 | 446, 452, 478 | 0.000442837 | 471 | 0.000457105 |
| 468 | 0.000402253 | 496, 501 | 0.000367341 | 468 | 0.000402253 |
| 450 | 0.000383968 | 475, 501 | 0.000277196 | 450 | 0.000383968 |
| 455 | 0.000310832 | 470 | 0.000226489 | 455 | 0.000310832 |
| 499 | 0.000274263 | 483, 501 | 0.000221982 | 499 | 0.000274263 |
| 503 | 0.000255979 | 477, 484 | 0.000211841 | 503 | 0.000255979 |
| 440, 477, 478, 484, 493, 498, 501, 505 | 0.000255979 | 459 | 0.000210714 | 440, 477, 478, 484, 493, 498, 501, 505 | 0.000255979 |
| 493 | 0.000237695 | 446, 501 | 0.000194939 | 493 | 0.000237695 |
| 472 | 0.000219411 | 479, 501 | 0.000182544 | 472 | 0.000219411 |
| 440, 445, 446, 460, 477, 478, 484, 486, 490, 498, 501, 505 | 0.000219411 | 452, 478, 484 | 0.000181417 | 440, 445, 446, 460, 477, 478, 484, 486, 490, 498, 501, 505 | 0.000219411 |
| 466 | 0.000201126 | 479 | 0.00018029 | 466 | 0.000201126 |
| 458 | 0.000182842 | 490 | 0.000167895 | 458 | 0.000182842 |
| 477, 478, 484, 493, 496, 498, 501, 505 | 0.000182842 | 441, 501 | 0.000145359 | 477, 478, 484, 493, 496, 498, 501, 505 | 0.000182842 |
| 463 | 0.000164558 | 440, 452 | 0.000131837 | 463 | 0.000164558 |
| 440, 452, 477, 478, 484, 486, 498, 501, 505 | 9.1421E-05 | 452, 477, 478 | 0.000129583 | 440, 452, 477, 478, 484, 486, 498, 501, 505 | 9.1421E-05 |
| 445 | 9.1421E-05 | 444, 452 | 0.000116062 | 445 | 9.1421E-05 |
| 477, 478, 484, 486, 498, 501, 505 | 7.31368E-05 | 476, 501 | 0.000111554 | 477, 478, 484, 486, 498, 501, 505 | 7.31368E-05 |
| 485 | 5.48526E-05 | 485, 501 | 9.91595E-05 | 485 | 5.48526E-05 |
| 507 | 5.48526E-05 | 508 | 9.0145E-05 | 507 | 5.48526E-05 |
| 482 | 5.48526E-05 | 446 | 8.00037E-05 | 482 | 5.48526E-05 |
| 441 | 3.65684E-05 | 470, 501 | 8.00037E-05 | 441 | 3.65684E-05 |
| 500 | 3.65684E-05 | 452, 478, 501 | 6.53551E-05 | 500 | 3.65684E-05 |
| 493, 496, 498, 501, 505 | 3.65684E-05 | 448, 501 | 6.42283E-05 | 493, 496, 498, 501, 505 | 3.65684E-05 |
| 449 | 3.65684E-05 | 478, 484 | 5.63406E-05 | 449 | 3.65684E-05 |
| 439, 494 | 3.65684E-05 | 440, 446, 477, 478, 484, 493, 496, 498, 501, 505 | 4.95798E-05 | 439, 494 | 3.65684E-05 |
| 498, 501, 505 | 1.82842E-05 | 483, 484 | 4.61993E-05 | 498, 501, 505 | 1.82842E-05 |
| 446, 498 | 1.82842E-05 | 483 | 4.39457E-05 | 446, 498 | 1.82842E-05 |
| 473 | 1.82842E-05 | 446, 452 | 4.39457E-05 | 473 | 1.82842E-05 |
| 446, 477, 478, 484, 493, 496, 498 | 1.82842E-05 | 445, 501 | 4.39457E-05 | 446, 477, 478, 484, 493, 496, 498 | 1.82842E-05 |
| 440, 445, 446, 450, 452, 455, 460, 477, 478, 481, 484, 486, 498, 501, 505 | 1.82842E-05 | 444 | 4.16921E-05 | 440, 445, 446, 450, 452, 455, 460, 477, 478, 481, 484, 486, 498, 501, 505 | 1.82842E-05 |
| 504 | 1.82842E-05 | 475 | 4.05653E-05 | 504 | 1.82842E-05 |
| 477, 478 | 1.82842E-05 | 476 | 3.94384E-05 | 477, 478 | 1.82842E-05 |
| 460, 477, 478, 484, 486, 490, 498, 501, 505 | 1.82842E-05 | 440, 446, 477, 478, 484, 493 | 3.49312E-05 | 460, 477, 478, 484, 486, 490, 498, 501, 505 | 1.82842E-05 |
| 469 | 1.82842E-05 | 501, 507 | 3.38044E-05 | 469 | 1.82842E-05 |
|  |  | 471 | 3.26776E-05 |  |  |
|  |  | 501, 508 | 3.15508E-05 |  |  |
|  |  | 452, 476, 478 | 3.04239E-05 |  |  |
|  |  | 503 | 2.92971E-05 |  |  |
|  |  | 501, 505 | 2.92971E-05 |  |  |
|  |  | 468 | 2.92971E-05 |  |  |
|  |  | 446, 477 | 2.92971E-05 |  |  |
|  |  | 477, 494 | 2.81703E-05 |  |  |
|  |  | 493 | 2.70435E-05 |  |  |
|  |  | 455 | 2.70435E-05 |  |  |
|  |  | 486, 501 | 2.47899E-05 |  |  |
|  |  | 465, 501 | 2.14094E-05 |  |  |
|  |  | 452, 455, 501 | 1.91558E-05 |  |  |
|  |  | 477, 478, 484, 493, 496, 498, 501, 505 | 1.8029E-05 |  |  |
|  |  | 441 | 1.57754E-05 |  |  |
|  |  | 481 | 1.57754E-05 |  |  |
|  |  | 460 | 1.57754E-05 |  |  |
|  |  | N/A | 1.57754E-05 |  |  |
|  |  | 452, 478, 490 | 1.46486E-05 |  |  |
|  |  | 439, 494 | 1.23949E-05 |  |  |
|  |  | 445 | 1.23949E-05 |  |  |
|  |  | 477, 484, 501 | 1.23949E-05 |  |  |
|  |  | 499 | 1.12681E-05 |  |  |
|  |  | 485 | 1.12681E-05 |  |  |
|  |  | 478, 484, 501 | 1.12681E-05 |  |  |
|  |  | 463 | 1.01413E-05 |  |  |
|  |  | 452, 475 | 1.01413E-05 |  |  |
|  |  | 452, 481 | 1.01413E-05 |  |  |
|  |  | 456, 501 | 9.0145E-06 |  |  |
|  |  | 439, 455 | 9.0145E-06 |  |  |
|  |  | 443 | 7.88769E-06 |  |  |
|  |  | 477, 490 | 7.88769E-06 |  |  |
|  |  | 440, 445, 446, 460, 477, 478, 484, 486, 490, 498, 501, 505 | 6.76088E-06 |  |  |
|  |  | 504 | 6.76088E-06 |  |  |
|  |  | 459, 501 | 6.76088E-06 |  |  |
|  |  | 455, 477 | 6.76088E-06 |  |  |
|  |  | 477, 483 | 6.76088E-06 |  |  |
|  |  | 477, 499 | 6.76088E-06 |  |  |
|  |  | 440, 452, 477, 478, 484, 486, 498, 501, 505 | 5.63406E-06 |  |  |
|  |  | 453 | 5.63406E-06 |  |  |
|  |  | 476, 484, 501 | 5.63406E-06 |  |  |
|  |  | 440, 477, 478, 484, 493, 498, 501, 505 | 4.50725E-06 |  |  |
|  |  | 472 | 4.50725E-06 |  |  |
|  |  | 440, 478 | 4.50725E-06 |  |  |
|  |  | 455, 484 | 4.50725E-06 |  |  |
|  |  | 477, 478 | 4.50725E-06 |  |  |
|  |  | 452, 504 | 4.50725E-06 |  |  |
|  |  | 439, 444 | 4.50725E-06 |  |  |
|  |  | 440, 490 | 3.38044E-06 |  |  |
|  |  | 458 | 3.38044E-06 |  |  |
|  |  | 477, 478, 484, 493, 498, 501, 505 | 3.38044E-06 |  |  |
|  |  | 477, 508 | 3.38044E-06 |  |  |
|  |  | 462, 501 | 3.38044E-06 |  |  |
|  |  | 465 | 3.38044E-06 |  |  |
|  |  | 475, 477 | 3.38044E-06 |  |  |
|  |  | 444, 477 | 3.38044E-06 |  |  |
|  |  | 477, 481 | 3.38044E-06 |  |  |
|  |  | 452, 490, 501 | 3.38044E-06 |  |  |
|  |  | 478, 479 | 2.25363E-06 |  |  |
|  |  | 440, 452, 477, 478, 484, 493, 498, 501, 505 | 2.25363E-06 |  |  |
|  |  | 456 | 2.25363E-06 |  |  |
|  |  | 477, 478, 484 | 2.25363E-06 |  |  |
|  |  | 438 | 2.25363E-06 |  |  |
|  |  | 462 | 2.25363E-06 |  |  |
|  |  | 474 | 2.25363E-06 |  |  |
|  |  | 446, 478 | 2.25363E-06 |  |  |
|  |  | 439, 446 | 2.25363E-06 |  |  |
|  |  | 452, 477, 484 | 2.25363E-06 |  |  |
|  |  | 452, 474 | 2.25363E-06 |  |  |
|  |  | 495, 501 | 2.25363E-06 |  |  |
|  |  | 484, 486, 501 | 2.25363E-06 |  |  |
|  |  | 453, 484 | 2.25363E-06 |  |  |
|  |  | 440, 477, 478, 484 | 1.12681E-06 |  |  |
|  |  | 479, 490, 501 | 1.12681E-06 |  |  |
|  |  | 460, 477, 478, 484, 486, 490, 498, 501, 505 | 1.12681E-06 |  |  |
|  |  | 491 | 1.12681E-06 |  |  |
|  |  | 493, 496, 498, 501, 505 | 1.12681E-06 |  |  |
|  |  | 437 | 1.12681E-06 |  |  |
|  |  | 484, 493, 496, 498, 501, 505 | 1.12681E-06 |  |  |
|  |  | 440, 446, 505 | 1.12681E-06 |  |  |
|  |  | 500 | 1.12681E-06 |  |  |
|  |  | 442 | 1.12681E-06 |  |  |
|  |  | 482 | 1.12681E-06 |  |  |
|  |  | 451 | 1.12681E-06 |  |  |
|  |  | 449 | 1.12681E-06 |  |  |
|  |  | 473 | 1.12681E-06 |  |  |
|  |  | 496, 501, 505 | 1.12681E-06 |  |  |
|  |  | 446, 477, 478, 484, 493, 496, 498, 501, 505 | 1.12681E-06 |  |  |
|  |  | 502 | 1.12681E-06 |  |  |
|  |  | 439, 471 | 1.12681E-06 |  |  |
|  |  | 507 | 1.12681E-06 |  |  |
|  |  | 457 | 1.12681E-06 |  |  |
|  |  | 477, 500 | 1.12681E-06 |  |  |
|  |  | 477, 504 | 1.12681E-06 |  |  |
|  |  | 472, 477 | 1.12681E-06 |  |  |
|  |  | 484, 508 | 1.12681E-06 |  |  |
|  |  | 452, 471 | 1.12681E-06 |  |  |
|  |  | 477, 490, 501 | 1.12681E-06 |  |  |
| **GRU** | | | | | |
| **①** | | **②** | | **③** | |
| **mutation combination** | **frequency** | **mutation combination** | **frequency** | **mutation combination** | **frequency** |
| N/A | 1 | N/A | 1 | N/A | 1 |

**Supplementary Table 4. The combinations and frequencies of predicted mutations for each data set.**

| **Training set 1** | |
| --- | --- |
| **mutation combination** | **frequency** |
| 440, 445, 446, 450, 452, 455, 460, 477, 478, 481, 484, 486, 498, 501, 505 | 0.740295096 |
| 440, 445, 446, 450, 452, 455, 456, 460, 477, 478, 481, 484, 486, 498, 501, 505 | 0.182689111 |
| N/A | 0.033686617 |
| 477, 478, 481, 484, 486, 498, 501, 505 | 0.006494181 |
| 440, 445, 446, 450, 452, 455, 460, 477, 478, 481, 484, 486, 498, 501 | 0.004280964 |
| 440, 445, 446, 450, 452, 455, 460, 477, 478, 481, 486, 498, 501, 505 | 0.001818371 |
| 440, 445, 446, 450, 452, 455, 460, 477, 478, 481, 484, 486 | 0.001724855 |
| 440, 445, 446, 450, 452, 460, 477, 478, 481, 484, 486, 498, 501, 505 | 0.001704073 |
| 498, 501, 505 | 0.001381962 |
| 440, 445, 446, 450, 452, 455, 460, 477, 478, 498, 501, 505 | 0.000997506 |
| 440, 441, 445, 446, 450, 452, 455, 460, 477, 478, 481, 484, 486, 498, 501, 505 | 0.000924771 |
| 440, 444, 445, 446, 450, 452, 455, 460, 477, 478, 481, 484, 486, 498, 501, 505 | 0.000696176 |
| 440, 445, 446, 450, 452, 455, 456, 460, 477, 481, 484, 486, 498, 501, 505 | 0.000613051 |
| 440, 441, 445, 446, 450, 452, 455, 456, 460, 477, 478, 481, 484, 486, 498, 501, 505 | 0.000550707 |
| 440, 445, 446, 450, 452, 455, 456, 460, 475, 477, 478, 481, 484, 486, 498, 501, 505 | 0.000540316 |
| 440, 445, 446, 450, 452, 455, 460, 477, 478, 486, 498, 501, 505 | 0.000509144 |
| 440, 445, 446, 450, 452, 455, 460, 477, 478, 481, 483, 484, 486, 498, 501, 505 | 0.000498753 |
| 440, 445, 446, 450, 452, 455, 460, 477, 478, 484, 486, 490, 498, 501, 505 | 0.000477972 |
| 440, 445, 446, 450, 452, 455, 456, 460, 477, 478, 481, 484, 486 | 0.000477972 |
| 460, 477, 478, 481, 484, 486, 498, 501, 505 | 0.00045719 |
| 440, 445, 446, 450, 452, 455, 460, 475, 477, 478, 481, 484, 486, 498, 501, 505 | 0.000426018 |
| 440, 445, 446, 450, 452, 455, 460, 477, 478, 484, 486, 498, 501, 505 | 0.000415628 |
| 477, 478, 486, 498, 501, 505 | 0.000415628 |
| 440, 446, 450, 452, 455, 460, 477, 478, 481, 484, 486, 498, 501, 505 | 0.000405237 |
| 505 | 0.000405237 |
| 440, 445, 446, 450, 452, 455, 456, 460, 477, 478, 481, 484, 486, 498, 501 | 0.000384456 |
| 440, 445, 446, 450, 452, 455, 460, 477, 478, 481 | 0.000363674 |
| 440, 445, 446, 450, 455, 460, 477, 478, 481, 484, 486, 498, 501, 505 | 0.000353283 |
| 440, 445, 446, 450, 452, 455, 460, 477, 478, 481, 484, 498, 501, 505 | 0.000342893 |
| 440, 445, 446, 450, 452, 455, 477, 478, 481, 484, 486, 498, 501, 505 | 0.000332502 |
| 440, 445, 446, 450, 452, 455, 460, 477, 481, 484, 486, 498, 501, 505 | 0.000322111 |
| 450, 452, 455, 460, 477, 478, 481, 484, 486, 498, 501, 505 | 0.000311721 |
| 440, 450, 452, 455, 460, 477, 478, 481, 484, 486, 498, 501, 505 | 0.00030133 |
| 440, 444, 445, 446, 450, 452, 453, 455, 460, 477, 478, 481, 484, 486, 498, 501, 505 | 0.00030133 |
| 477, 478, 498, 501, 505 | 0.000290939 |
| 440, 445, 446, 450, 452, 455, 460 | 0.000280549 |
| 440, 445, 446, 450, 452, 455, 460, 498, 501, 505 | 0.000270158 |
| 445, 446, 450, 452, 455, 460, 477, 478, 481, 484, 486, 498, 501, 505 | 0.000270158 |
| 440, 445, 446, 450, 452, 455, 460, 477, 478, 482, 483, 484, 486, 498, 501, 505 | 0.000259767 |
| 440, 445, 446, 450, 452, 455, 460, 477, 478, 479, 481, 484, 486, 498, 501, 505 | 0.000238986 |
| 440, 445, 446, 450, 452, 455, 456, 460, 477, 478, 481, 484, 486, 493, 498, 501, 505 | 0.000238986 |
| 440, 445, 446, 450, 452, 455, 456, 460, 477, 478, 481 | 0.000228595 |
| 440, 445, 446, 450, 452, 455, 460, 477, 478, 481, 498, 501, 505 | 0.000218204 |
| 460, 477, 478, 481, 484, 486 | 0.000218204 |
| 440, 477, 478, 481, 484, 486, 498, 501, 505 | 0.000207814 |
| 440, 460 | 0.000207814 |
| 477, 478, 481, 484, 486 | 0.000207814 |
| 440, 445, 446, 455, 460, 477, 478, 481, 484, 486, 498, 501, 505 | 0.000166251 |
| 440, 445, 446, 450, 452, 455, 460, 477, 478, 482, 486, 498, 501, 505 | 0.00015586 |
| 440, 445, 446, 450, 452, 455, 460, 477, 478, 481, 484, 486, 498, 500, 501, 505 | 0.00015586 |
| 477, 478 | 0.00014547 |
| 440, 445, 446, 450, 452, 455, 460, 476, 477, 478, 481, 484, 486, 498, 501, 505 | 0.00014547 |
| 498, 501 | 0.00014547 |
| 455, 460, 477, 478, 481, 484, 486, 498, 501, 505 | 0.00014547 |
| 440, 445, 446, 450, 452, 455, 456, 460, 477, 478, 498, 501, 505 | 0.000135079 |
| 440, 460, 498 | 0.000135079 |
| 440, 445, 446, 450, 452, 455, 460, 477, 478, 481, 484, 486, 498 | 0.000114298 |
| 440, 445, 446, 477, 478, 481, 484, 486, 498, 501, 505 | 0.000114298 |
| 501, 505 | 0.000114298 |
| 440, 445, 446, 450, 452, 455, 460, 477, 478, 482, 484, 486, 498, 501, 505 | 0.000114298 |
| 440, 445, 446, 450, 455, 456, 460, 477, 478, 481, 498, 501, 505 | 0.000114298 |
| 440, 444, 445, 446, 450, 452, 455, 456, 460, 477, 478, 481, 484, 486, 498, 501, 505 | 0.000114298 |
| 440, 445, 446, 450, 452, 455, 460, 477, 478 | 0.000103907 |
| 450, 452, 455, 456, 460, 477, 478, 481, 484, 486, 498, 501, 505 | 0.000103907 |
| 440, 445, 446, 450, 452, 455, 460, 477, 478, 481, 484, 486, 498, 501, 503, 505 | 9.35162E-05 |
| 460 | 9.35162E-05 |
| 440, 445, 446, 450, 460, 477, 478, 481, 484, 486, 498, 501, 505 | 9.35162E-05 |
| 501 | 9.35162E-05 |
| 440, 445, 446, 450, 452, 455, 456, 460, 477, 478, 481, 484, 498, 501, 505 | 9.35162E-05 |
| 440, 445, 446, 450, 452, 455, 456, 460, 477, 478, 481, 486, 498, 501, 505 | 9.35162E-05 |
| 440, 445, 446, 477, 478, 486, 498, 501, 505 | 8.31255E-05 |
| 440, 445, 446, 450, 452, 455, 460, 470, 477, 478, 481, 484, 486, 498, 501, 505 | 8.31255E-05 |
| 440, 460, 477, 478, 481, 484, 486, 498, 501, 505 | 8.31255E-05 |
| 440, 445, 446, 450, 452, 455, 460, 477, 478, 481, 484, 485, 486, 498, 501, 505 | 8.31255E-05 |
| 440, 445, 446, 450, 452, 455, 456, 460, 477, 478, 481, 484, 486, 498, 500, 501, 505 | 8.31255E-05 |
| 450, 477, 478, 481, 484, 486, 498, 501, 505 | 7.27348E-05 |
| 440, 455, 460, 477, 478, 481, 484, 486, 498, 501, 505 | 7.27348E-05 |
| 498 | 7.27348E-05 |
| 440, 445, 446, 450, 452, 455, 477, 478, 498, 501, 505 | 7.27348E-05 |
| 440 | 7.27348E-05 |
| 440, 445, 446, 450, 452, 455, 458, 460, 477, 478, 481, 484, 486, 498, 501, 505 | 7.27348E-05 |
| 440, 460, 498, 505 | 7.27348E-05 |
| 455, 460, 477, 478, 498, 501, 505 | 6.23441E-05 |
| 440, 445, 446, 450, 452, 455, 498, 501, 505 | 6.23441E-05 |
| 455, 460, 477, 478, 481, 484, 486, 498, 501 | 6.23441E-05 |
| 455, 460, 477, 478, 481, 484, 486 | 6.23441E-05 |
| 440, 445, 446, 450, 452, 455, 460, 462, 477, 478, 481, 484, 486, 498, 501, 505 | 6.23441E-05 |
| 460, 477, 478 | 6.23441E-05 |
| 440, 445, 446, 450, 452, 455, 460, 477, 478, 481, 484, 486, 501, 505 | 6.23441E-05 |
| 455, 498, 501, 505 | 6.23441E-05 |
| 440, 446, 450, 452, 455, 456, 460, 477, 478, 481, 484, 486, 498, 501, 505 | 6.23441E-05 |
| 440, 445, 446, 450, 452, 455, 456, 460, 477, 478, 482, 483, 484, 486, 498, 501, 505 | 6.23441E-05 |
| 440, 445, 446, 450, 455, 460, 477, 478, 486, 498, 501, 505 | 5.19534E-05 |
| 460, 477, 478, 481 | 5.19534E-05 |
| 440, 445, 477, 478, 481, 484, 486, 498, 501, 505 | 5.19534E-05 |
| 440, 445, 446, 450, 452, 455, 460, 477, 478, 483, 484, 486, 498, 501, 505 | 5.19534E-05 |
| 440, 445, 446, 450, 452, 477, 478, 481, 484, 486, 498, 501, 505 | 5.19534E-05 |
| 440, 445, 446, 452, 455, 460, 477, 478, 481, 484, 486, 498, 501, 505 | 5.19534E-05 |
| 450, 455, 460, 477, 478, 481, 484, 486, 498, 501, 505 | 5.19534E-05 |
| 440, 445, 446, 460, 477, 478, 481, 484, 486, 498, 501, 505 | 5.19534E-05 |
| 440, 450, 460, 477, 478, 481, 484, 486, 498, 501, 505 | 5.19534E-05 |
| 477, 478, 481 | 5.19534E-05 |
| 440, 460, 505 | 5.19534E-05 |
| 445, 446, 450, 452, 455, 456, 460, 477, 478, 481, 484, 486, 498, 501, 505 | 5.19534E-05 |
| 440, 445, 446, 450, 452, 455, 456, 459, 460, 477, 478, 481, 484, 486, 498, 501, 505 | 5.19534E-05 |
| 440, 445, 446, 460, 477, 478, 486, 498, 501, 505 | 4.15628E-05 |
| 445, 446, 450, 452, 455, 460, 477, 478, 481, 484, 486 | 4.15628E-05 |
| 450, 455, 498, 501, 505 | 4.15628E-05 |
| 440, 445, 446, 450, 452, 455 | 4.15628E-05 |
| 477, 478, 481, 498, 501, 505 | 4.15628E-05 |
| 440, 445, 446, 450, 452, 455, 460, 477, 478, 481, 484, 486, 487, 498, 501, 505 | 4.15628E-05 |
| 460, 477, 478, 498, 501, 505 | 4.15628E-05 |
| 477, 478, 481, 484, 486, 498, 501 | 4.15628E-05 |
| 440, 450, 455, 460, 477, 478, 481, 484, 486, 498, 501, 505 | 4.15628E-05 |
| 440, 445, 446, 450, 452, 498, 501, 505 | 4.15628E-05 |
| 440, 445, 446, 450, 455, 460, 477, 478, 484, 486, 498, 501, 505 | 4.15628E-05 |
| 440, 445, 446, 450, 452, 455, 460, 477, 478, 481, 484, 486, 494, 498, 501, 505 | 4.15628E-05 |
| 440, 450, 455, 460, 477, 478, 498, 501, 505 | 4.15628E-05 |
| 450, 452, 455, 460, 477, 478, 498, 501, 505 | 4.15628E-05 |
| 440, 445, 446, 450, 452, 455, 460, 477, 498, 501, 505 | 4.15628E-05 |
| 440, 445, 446, 450, 452, 456, 460, 477, 478, 481, 484, 486, 498, 501, 505 | 4.15628E-05 |
| 440, 445, 446, 450, 452, 455, 456, 460, 477, 478, 481, 498, 501, 505 | 4.15628E-05 |
| 440, 445, 446, 450, 452, 455, 460, 477, 478, 484, 498, 501, 505 | 4.15628E-05 |
| 440, 450, 452, 455, 456, 460, 477, 478, 481, 484, 486, 498, 501, 505 | 4.15628E-05 |
| 440, 445, 446, 450, 452, 455, 456, 460, 477, 478, 484, 486, 498, 501, 505 | 4.15628E-05 |
| 440, 445, 446, 450, 460, 477, 478, 486, 498, 501, 505 | 3.11721E-05 |
| 440, 445, 460, 477, 478, 481, 484, 486, 498, 501, 505 | 3.11721E-05 |
| 440, 445, 446, 456, 460, 477, 478, 484, 486, 490, 498, 501, 505 | 3.11721E-05 |
| 440, 445, 446, 452, 460, 477, 478, 484, 486, 498, 501, 505 | 3.11721E-05 |
| 440, 445, 446, 452, 456, 460, 477, 478, 484, 486, 490, 498, 501, 505 | 3.11721E-05 |
| 440, 460, 477, 478, 486, 498, 501, 505 | 3.11721E-05 |
| 455, 460, 498, 501, 505 | 3.11721E-05 |
| 440, 445, 446, 450, 452, 455, 459, 460, 477, 478, 481, 484, 486, 498, 501, 505 | 3.11721E-05 |
| 440, 445, 446, 450, 452, 498, 501 | 3.11721E-05 |
| 440, 445, 446, 450, 452, 455, 456, 460, 477, 478, 484, 486, 490, 498, 501, 505 | 3.11721E-05 |
| 455 | 3.11721E-05 |
| 440, 450, 452, 455, 460, 477, 478 | 3.11721E-05 |
| 450, 452, 455, 460, 477, 478, 481, 484, 486 | 3.11721E-05 |
| 440, 445, 446, 450, 477, 478, 481, 484, 486, 498, 501, 505 | 3.11721E-05 |
| 477, 498, 501, 505 | 3.11721E-05 |
| 440, 445, 446, 450, 452, 455, 460, 474, 477, 478, 481, 484, 486, 498, 501, 505 | 3.11721E-05 |
| 440, 450, 477, 478, 481, 484, 486, 498, 501, 505 | 3.11721E-05 |
| 450, 452, 455, 460, 477, 478, 481, 484, 486, 498, 501 | 3.11721E-05 |
| 440, 443, 445, 446, 450, 452, 455, 460, 477, 478, 481, 484, 486, 498, 501, 505 | 3.11721E-05 |
| 440, 450, 452, 455, 460, 477, 478, 481, 484, 486, 498, 501 | 3.11721E-05 |
| 450, 452, 455, 460, 477, 478 | 3.11721E-05 |
| 440, 445, 446, 450, 452, 455, 456, 460, 477, 478, 481, 483, 484, 486, 498, 501, 505 | 3.11721E-05 |
| 450, 455, 460, 477, 478, 498, 501, 505 | 3.11721E-05 |
| 440, 445, 446, 450, 452, 477, 478, 498, 501, 505 | 3.11721E-05 |
| 440, 445, 446, 450, 452, 477, 478, 486, 498, 501, 505 | 3.11721E-05 |
| 440, 445, 446, 450, 452, 453, 455, 460, 477, 478, 481, 484, 486, 498, 501, 505 | 3.11721E-05 |
| 440, 445, 446, 450, 452, 455, 456, 460, 476, 477, 478, 481, 484, 486, 498, 501, 505 | 3.11721E-05 |
| 440, 445, 446, 450, 452, 455, 456, 460, 477, 478, 481, 484, 485, 486, 498, 501, 505 | 3.11721E-05 |
| 440, 445, 446, 455, 456, 460, 477, 478, 484, 486, 490, 498, 501, 505 | 2.07814E-05 |
| 455, 460, 477, 478 | 2.07814E-05 |
| 440, 445, 446, 450, 452, 455, 460, 505 | 2.07814E-05 |
| 440, 455, 460, 477, 478, 498, 501, 505 | 2.07814E-05 |
| 440, 445, 446, 455, 460, 477, 478, 484, 486, 490, 498, 501, 505 | 2.07814E-05 |
| 440, 445, 446, 455, 456, 460, 477, 478, 481, 484, 486, 498, 501, 505 | 2.07814E-05 |
| 450, 452, 455, 477, 478, 481, 484, 486, 498, 501, 505 | 2.07814E-05 |
| 440, 445, 446, 450, 455, 460, 477, 478, 481, 484, 486, 498, 501 | 2.07814E-05 |
| 477, 478, 484, 486, 498, 501, 505 | 2.07814E-05 |
| 440, 445, 446, 450, 477, 478, 486, 498, 501, 505 | 2.07814E-05 |
| 481, 484, 486, 498, 501, 505 | 2.07814E-05 |
| 440, 445, 446, 460, 477, 478, 484, 486, 498, 501, 505 | 2.07814E-05 |
| 440, 445, 446, 450, 452, 460, 477, 478, 486, 498, 501, 505 | 2.07814E-05 |
| 486, 498, 501, 505 | 2.07814E-05 |
| 440, 445, 446, 450, 452, 455, 460, 477, 478, 498, 501 | 2.07814E-05 |
| 450, 452 | 2.07814E-05 |
| 440, 445, 446, 450, 452, 455, 460, 477, 478, 481, 484, 486, 502, 503 | 2.07814E-05 |
| 460, 498, 501, 505 | 2.07814E-05 |
| 460, 477, 478, 481, 484, 486, 498, 501 | 2.07814E-05 |
| 440, 445, 446, 452, 460, 477, 478, 486, 498, 501, 505 | 2.07814E-05 |
| 440, 450, 455, 460, 477, 478, 481, 484, 486, 498 | 2.07814E-05 |
| 440, 445, 446, 450, 452, 455, 460, 477, 478, 501, 505 | 2.07814E-05 |
| 450, 452, 455, 460, 477, 478, 481 | 2.07814E-05 |
| 455, 477, 478, 481, 484, 486, 498, 501, 505 | 2.07814E-05 |
| 440, 450, 452, 460, 477, 478 | 2.07814E-05 |
| 450, 460, 477, 478 | 2.07814E-05 |
| 445, 446, 450, 452, 455, 477, 478, 481, 484, 486, 498, 501, 505 | 2.07814E-05 |
| 440, 445, 446, 450, 452, 455, 460, 477, 478, 481, 484, 486, 505 | 2.07814E-05 |
| 450, 460, 477, 478, 481, 484, 486, 498, 501, 505 | 2.07814E-05 |
| 440, 445, 450, 452, 455, 460, 477, 478, 481, 484, 486, 498, 501, 505 | 2.07814E-05 |
| 450, 452, 477, 478, 481, 484, 486, 498, 501, 505 | 2.07814E-05 |
| 440, 446, 450, 452, 455, 460, 477, 478, 498, 501, 505 | 2.07814E-05 |
| 445, 446, 450, 452, 455, 460, 477, 478, 481, 498, 501, 505 | 2.07814E-05 |
| 477, 478, 498, 501 | 2.07814E-05 |
| 440, 445, 446, 450, 452, 455, 460, 477, 478, 481, 484, 486, 501 | 2.07814E-05 |
| 440, 498, 501, 505 | 2.07814E-05 |
| 440, 445, 446, 450, 452, 455, 460, 486, 498, 501, 505 | 2.07814E-05 |
| 455, 460 | 2.07814E-05 |
| 455, 460, 477, 478, 481, 498, 501, 505 | 2.07814E-05 |
| 440, 445, 446, 450, 452, 455, 460, 477, 478, 481, 485, 498, 501, 505 | 2.07814E-05 |
| 460, 498 | 2.07814E-05 |
| 440, 450, 455, 477, 478, 481, 484, 486, 498, 501, 505 | 2.07814E-05 |
| 477, 478, 481, 486, 498, 501, 505 | 2.07814E-05 |
| 477, 478, 501 | 2.07814E-05 |
| 440, 445, 446, 450, 452, 455, 460, 477, 478, 481, 484, 486, 498, 501, 505, 508 | 2.07814E-05 |
| 440, 450, 452, 455, 477, 478, 481, 484, 486, 498, 501, 505 | 2.07814E-05 |
| 440, 445, 446, 450, 452, 460, 477, 478, 481, 484, 498, 501, 505 | 2.07814E-05 |
| 440, 445, 446, 450, 452, 455, 477, 478, 486, 498, 501, 505 | 2.07814E-05 |
| 445, 446, 450, 452, 498, 501, 505 | 2.07814E-05 |
| 440, 445, 446, 450, 452, 477, 478, 484, 486, 498, 501, 505 | 2.07814E-05 |
| 450, 452, 455, 460, 498, 501, 505 | 2.07814E-05 |
| 440, 445, 446, 450, 452, 455, 460, 477, 478, 481, 484, 486, 498, 501, 504, 505 | 2.07814E-05 |
| 440, 477, 478, 498, 501, 505 | 2.07814E-05 |
| 477, 478, 481, 484, 498, 501, 505 | 2.07814E-05 |
| 440, 445, 446, 450, 452, 455, 460, 477, 478, 481, 484, 486, 493, 498, 501, 505 | 2.07814E-05 |
| 450, 452, 455, 456, 460, 477, 478, 498, 501, 505 | 2.07814E-05 |
| 440, 445, 446, 450, 452, 455, 456, 460, 477, 478, 482, 484, 486, 498, 501, 505 | 2.07814E-05 |
| 455, 456, 460 | 2.07814E-05 |
| 455, 456, 460, 477, 478, 481, 484, 486, 498, 501, 505 | 2.07814E-05 |
| 440, 445, 446, 455, 456, 460, 477, 478, 481, 498, 501, 505 | 2.07814E-05 |
| 440, 445, 446, 450, 452, 453, 455, 456, 460, 477, 478, 481, 484, 486, 498, 501, 505 | 2.07814E-05 |
| 440, 445, 446, 450, 452, 455, 456, 498, 501, 505 | 2.07814E-05 |
| 440, 445, 446, 450, 452, 455, 456, 460, 468, 477, 478, 481, 484, 486, 498, 501, 505 | 2.07814E-05 |
| 450, 452, 455, 456, 498, 501, 505 | 2.07814E-05 |
| 440, 445, 446, 450, 452, 455, 456, 460, 477, 498, 501, 505 | 2.07814E-05 |
| 440, 445, 446, 450, 455, 456, 460, 477, 478, 481, 484, 486, 498, 501, 505 | 2.07814E-05 |
| 456, 460, 477, 478, 481, 484, 486, 498, 501, 505 | 2.07814E-05 |
| 440, 445, 446, 450, 452, 455, 456, 460, 477, 478, 479, 481, 484, 486, 498, 501, 505 | 2.07814E-05 |
| 440, 445, 446, 450, 452, 460, 477, 478, 498, 501, 505 | 1.03907E-05 |
| 440, 445, 446, 456, 460, 484, 486, 490, 498, 501, 505 | 1.03907E-05 |
| 440, 452, 460, 477, 478, 481, 484, 486 | 1.03907E-05 |
| 440, 445, 446, 450, 452, 455, 460, 477, 478, 481, 484, 501, 505 | 1.03907E-05 |
| 440, 445, 446, 477, 486, 498, 501, 505 | 1.03907E-05 |
| 440, 445, 446, 450, 452, 455, 460, 498, 501 | 1.03907E-05 |
| 440, 445, 477 | 1.03907E-05 |
| 440, 445, 446, 460, 477, 478, 501, 505 | 1.03907E-05 |
| 440, 450, 460, 477, 478, 486, 498, 501, 505 | 1.03907E-05 |
| 445, 446, 450, 452, 455, 460, 477, 478, 481, 484, 486, 498 | 1.03907E-05 |
| 440, 445, 446, 477, 478, 498, 501, 505 | 1.03907E-05 |
| 440, 450, 452, 455, 460, 477, 478, 481, 484, 486 | 1.03907E-05 |
| 440, 445, 446, 477, 498, 501, 505 | 1.03907E-05 |
| 455, 456, 475, 477, 478, 484, 486, 490 | 1.03907E-05 |
| 440, 441, 445, 446, 450, 455, 460, 477, 478, 481, 484, 486, 498, 501, 505 | 1.03907E-05 |
| 440, 445, 446, 450, 452, 455, 460, 475, 477, 478, 498, 501, 505 | 1.03907E-05 |
| 440, 445, 446, 450, 452, 455, 477, 498, 501, 505 | 1.03907E-05 |
| 450, 455, 477, 478, 481, 484, 486, 498, 501, 505 | 1.03907E-05 |
| 440, 445, 446, 452, 477, 478, 481, 484, 486, 498, 501, 505 | 1.03907E-05 |
| 440, 450, 455, 498, 501, 505 | 1.03907E-05 |
| 440, 445, 446, 452, 455, 460, 477, 478, 484, 486, 498, 501, 505 | 1.03907E-05 |
| 440, 445, 446, 450, 477, 486, 498, 501, 505 | 1.03907E-05 |
| 452, 455, 460, 477, 478, 481, 484, 486 | 1.03907E-05 |
| 440, 445, 446, 452, 455, 477, 478, 481, 484, 486, 498, 501, 505 | 1.03907E-05 |
| 440, 455, 460, 477, 478, 481, 486, 498, 501, 505 | 1.03907E-05 |
| 450, 452, 455, 498, 501 | 1.03907E-05 |
| 440, 445, 446, 450, 452, 455, 475, 477, 478, 481, 484, 486 | 1.03907E-05 |
| 440, 452, 455, 460, 477, 478, 481, 484, 486 | 1.03907E-05 |
| 440, 445, 446, 450, 455, 460, 477, 478, 481, 484, 486 | 1.03907E-05 |
| 440, 445, 446, 450, 455, 477, 478, 486, 498, 501, 505 | 1.03907E-05 |
| 440, 445, 446, 460, 477, 478, 484, 486, 490, 498, 501, 505 | 1.03907E-05 |
| 450, 455, 460, 477, 478 | 1.03907E-05 |
| 440, 445, 446, 450, 455, 460, 477, 478, 481, 484, 486, 501, 505 | 1.03907E-05 |
| 440, 446, 455, 460, 477, 478, 481, 484, 486, 498, 501 | 1.03907E-05 |
| 476, 477, 478, 481, 484, 486, 498, 501, 505 | 1.03907E-05 |
| 440, 445, 446, 450, 452, 455, 476, 477, 478, 481, 484, 486, 498, 501, 505 | 1.03907E-05 |
| 440, 445, 446, 456, 460 | 1.03907E-05 |
| 440, 450, 477, 478, 481, 484, 486 | 1.03907E-05 |
| 450, 455, 477, 498, 501, 505 | 1.03907E-05 |
| 440, 445, 446, 450, 452, 455, 460, 478, 484, 486, 498, 501, 505 | 1.03907E-05 |
| 440, 445, 446, 450, 452, 455, 477, 478, 481, 498, 501, 505 | 1.03907E-05 |
| 440, 445, 446, 450, 455, 460, 498, 501, 505 | 1.03907E-05 |
| 460, 477, 478, 484, 498 | 1.03907E-05 |
| 477, 505 | 1.03907E-05 |
| 440, 445, 446, 450, 452, 455, 460, 477, 478, 481, 484, 486, 498, 501, 507 | 1.03907E-05 |
| 440, 446, 450, 452, 455, 460, 477, 478, 481 | 1.03907E-05 |
| 440, 446, 477, 478, 486, 498, 501, 505 | 1.03907E-05 |
| 440, 445, 446, 450, 452, 455, 460, 477, 478, 479, 498, 501, 505 | 1.03907E-05 |
| 455, 460, 477, 478, 481, 498 | 1.03907E-05 |
| 440, 445, 446, 450, 452, 455, 460, 507 | 1.03907E-05 |
| 440, 445, 446, 450, 452, 460, 477, 478, 481 | 1.03907E-05 |
| 484 | 1.03907E-05 |
| 477, 478, 481, 484, 486, 498, 505 | 1.03907E-05 |
| 440, 445, 446, 456, 460, 477, 478, 484, 486, 498, 501, 505 | 1.03907E-05 |
| 450, 452, 455, 460 | 1.03907E-05 |
| 440, 445, 446, 450, 452 | 1.03907E-05 |
| 507 | 1.03907E-05 |
| 446, 450, 452, 455, 460, 477, 478, 498, 501, 505 | 1.03907E-05 |
| 440, 446, 450, 452, 455, 460, 477, 478, 484, 486, 490, 498, 501, 505 | 1.03907E-05 |
| 440, 445, 446, 452, 477, 486, 498, 501, 505 | 1.03907E-05 |
| 440, 445, 446, 450, 455, 460, 477, 478, 481 | 1.03907E-05 |
| 440, 445, 446, 452, 455, 477, 478, 481, 486, 498, 501, 505 | 1.03907E-05 |
| 440, 450, 452, 455, 477, 478, 498, 501, 505 | 1.03907E-05 |
| 440, 445, 446, 452, 455, 460, 477, 478, 486, 498, 501, 505 | 1.03907E-05 |
| 477, 478, 498 | 1.03907E-05 |
| 477, 478, 481, 501 | 1.03907E-05 |
| 450, 452, 455, 460, 498, 501 | 1.03907E-05 |
| 440, 445, 446, 450, 460, 477, 478, 481, 486, 498, 501, 505 | 1.03907E-05 |
| 445, 446, 450, 452, 455, 477, 478, 498, 501, 505 | 1.03907E-05 |
| 440, 446, 460, 477, 478, 484, 486, 498, 501, 505 | 1.03907E-05 |
| 440, 445, 450, 452, 455, 498, 501, 505 | 1.03907E-05 |
| 440, 450, 452, 455, 460, 477, 478, 498, 501, 505 | 1.03907E-05 |
| 445, 446, 450, 452, 460, 477, 478, 481, 484, 486, 498, 501, 505 | 1.03907E-05 |
| 445, 446, 452, 455, 460, 477, 478, 481, 484, 486, 498, 501, 505 | 1.03907E-05 |
| 477, 478, 481, 484, 486, 501 | 1.03907E-05 |
| 440, 445, 446, 450, 460, 477, 478, 484, 486, 498, 501, 505 | 1.03907E-05 |
| 440, 446, 452, 460, 477, 478, 486, 498, 501, 505 | 1.03907E-05 |
| 440, 446, 460, 477, 478, 486, 498, 501 | 1.03907E-05 |
| 450 | 1.03907E-05 |
| 440, 445, 446, 450, 452, 455, 460, 477, 478, 482, 483, 484, 486, 498, 501 | 1.03907E-05 |
| 440, 445, 446, 452, 460, 477, 478, 484, 486, 490, 498, 501, 505 | 1.03907E-05 |
| 455, 477, 478 | 1.03907E-05 |
| 450, 498, 501, 505 | 1.03907E-05 |
| 446, 450, 452, 455, 460, 477, 478, 481, 484, 486, 498, 501 | 1.03907E-05 |
| 440, 446, 455, 460, 477, 478, 486, 498, 501, 505 | 1.03907E-05 |
| 440, 445, 450, 498, 501, 505 | 1.03907E-05 |
| 445, 446, 450, 452, 455, 460, 498, 501, 505 | 1.03907E-05 |
| 478, 481, 501, 505 | 1.03907E-05 |
| 440, 445, 452, 456, 460, 477, 478, 484, 486, 498, 501, 505 | 1.03907E-05 |
| 445, 446, 450, 452, 455, 460, 477, 478, 481, 486 | 1.03907E-05 |
| 445, 446, 450, 452, 477, 478, 481, 498, 501, 505 | 1.03907E-05 |
| 440, 445, 446, 450, 452, 455, 477, 478, 481, 486, 498, 501, 505 | 1.03907E-05 |
| 477 | 1.03907E-05 |
| 460, 477, 478, 481, 484, 486, 498 | 1.03907E-05 |
| 445, 446, 450, 452, 460, 477, 478, 481, 501, 505 | 1.03907E-05 |
| 440, 445, 446, 450, 451, 452, 455, 460, 477, 478, 498, 501, 505 | 1.03907E-05 |
| 440, 446, 450, 460, 477, 478, 486, 498, 501, 505 | 1.03907E-05 |
| 461, 477, 478, 481, 484, 486, 498, 501, 505 | 1.03907E-05 |
| 445, 446, 450, 477, 478, 481 | 1.03907E-05 |
| 440, 445, 446, 450, 452, 455, 460, 477, 478, 484, 486, 498, 505 | 1.03907E-05 |
| 440, 445, 446, 450, 455, 460, 477, 486, 498, 501, 505 | 1.03907E-05 |
| 440, 445, 446, 450, 455, 460, 477, 478, 481, 486, 498, 501, 505 | 1.03907E-05 |
| 445, 446, 455, 460, 477, 478, 481 | 1.03907E-05 |
| 440, 450, 452, 455, 460, 477, 498, 501, 505 | 1.03907E-05 |
| 440, 445, 446, 450, 452, 455, 477, 486, 498, 501, 505 | 1.03907E-05 |
| 440, 450, 452, 455, 460, 477, 478, 481 | 1.03907E-05 |
| 440, 445, 446, 450, 452, 455, 460, 477, 486, 498, 501, 505 | 1.03907E-05 |
| 440, 445, 446, 450, 455, 477, 486, 498, 501, 505 | 1.03907E-05 |
| 450, 452, 455, 460, 477, 478, 481, 484, 486, 498 | 1.03907E-05 |
| 440, 445, 446, 450, 452, 477, 478, 498, 501 | 1.03907E-05 |
| 460, 505 | 1.03907E-05 |
| 445, 450, 452, 455, 460, 477, 478 | 1.03907E-05 |
| 455, 498 | 1.03907E-05 |
| 440, 445, 446, 450, 452, 455, 460, 477, 478, 486, 498, 501 | 1.03907E-05 |
| 440, 460, 498, 501, 505 | 1.03907E-05 |
| 440, 445, 446, 450, 455, 477, 478, 498, 501, 505 | 1.03907E-05 |
| 440, 445, 446, 450, 452, 455, 460, 477, 478, 481, 484, 498, 501 | 1.03907E-05 |
| 450, 460, 498, 501 | 1.03907E-05 |
| 455, 460, 477, 478, 481, 486, 501 | 1.03907E-05 |
| 440, 450, 455, 460, 477, 478, 481, 498, 501, 505 | 1.03907E-05 |
| 440, 452, 455, 460, 477, 478, 481, 484, 486, 498, 501 | 1.03907E-05 |
| 440, 445, 446, 452, 455, 460, 477, 478, 481, 498, 501, 505 | 1.03907E-05 |
| 440, 445, 446, 450, 455, 477, 478, 481, 484, 486, 498, 501, 505 | 1.03907E-05 |
| 445, 446, 460, 477, 478, 481, 484, 486, 498, 501 | 1.03907E-05 |
| 440, 445, 446, 450, 452, 455, 460, 477, 478, 481, 484, 486, 498, 505 | 1.03907E-05 |
| 440, 441, 445, 446, 450, 452, 455, 460, 477, 478, 481 | 1.03907E-05 |
| 450, 452, 455, 460, 477, 478, 501 | 1.03907E-05 |
| 450, 452, 460, 477, 478, 498, 501, 505 | 1.03907E-05 |
| 440, 450, 452, 455, 460, 477, 478, 481, 498, 501, 505 | 1.03907E-05 |
| 440, 445, 446, 450, 452, 455, 477, 478, 498, 501 | 1.03907E-05 |
| 440, 445, 446, 450, 452, 455, 460, 475, 477, 478, 481 | 1.03907E-05 |
| 440, 445, 446, 450, 452, 455, 456, 460, 475, 477, 478, 481, 484, 498, 501, 505 | 1.03907E-05 |
| 440, 445, 446, 450, 452, 455, 460, 478, 481, 484, 486, 498, 501, 505 | 1.03907E-05 |
| 440, 445, 446, 450, 452, 455, 460, 477, 478, 481, 498, 501 | 1.03907E-05 |
| 440, 445, 446, 450, 452, 455, 460, 477, 478, 481, 484, 486, 498, 499, 501 | 1.03907E-05 |
| 440, 445, 446, 450, 452, 455, 460, 477, 478, 482, 484, 486, 498, 501 | 1.03907E-05 |
| 460, 501 | 1.03907E-05 |
| 455, 460, 505 | 1.03907E-05 |
| 440, 455, 456, 460, 477, 478, 481, 484, 486, 498, 501 | 1.03907E-05 |
| 440, 486, 498, 501, 505 | 1.03907E-05 |
| 445, 446, 450, 452, 455, 460, 477, 478, 481, 501 | 1.03907E-05 |
| 440, 452, 455, 460, 477, 478, 481, 484, 486, 498, 501, 505 | 1.03907E-05 |
| 460, 477, 478, 481, 498, 501, 505 | 1.03907E-05 |
| 440, 445, 446, 450, 452, 455, 460, 461, 477, 478, 481, 484, 486, 498, 501, 505 | 1.03907E-05 |
| 445, 446, 450, 455, 460, 477, 478, 481, 484, 486, 498, 501, 505 | 1.03907E-05 |
| 498, 505 | 1.03907E-05 |
| 440, 445, 446, 450, 452, 455, 456, 460, 486, 498, 501, 505 | 1.03907E-05 |
| 440, 445, 446, 450, 452, 455, 477, 478, 484, 486, 498, 501, 505 | 1.03907E-05 |
| 440, 445, 446, 448, 450, 452, 455, 460, 477, 478, 481, 484, 486, 498, 501, 505 | 1.03907E-05 |
| 477, 478, 481, 484 | 1.03907E-05 |
| 440, 445, 446, 455, 477, 478, 481, 484, 486, 498, 501, 505 | 1.03907E-05 |
| 440, 445, 446, 455, 460, 477, 478, 486, 498, 501, 505 | 1.03907E-05 |
| 440, 445, 446, 450, 452, 455, 460, 477, 478, 481, 484, 486, 498, 499, 501, 505 | 1.03907E-05 |
| 440, 450, 452, 475, 477, 478, 481, 484, 486, 498, 501, 505 | 1.03907E-05 |
| 440, 450, 452, 460, 498, 501, 505 | 1.03907E-05 |
| 440, 445, 446, 450, 452, 460, 477, 478, 481, 484, 486, 498, 501 | 1.03907E-05 |
| 440, 445, 446, 450, 452, 455, 477, 478, 481, 484, 486 | 1.03907E-05 |
| 450, 452, 455 | 1.03907E-05 |
| 450, 452, 455, 460, 477, 478, 498, 501 | 1.03907E-05 |
| 450, 460, 477, 478, 498, 501, 505 | 1.03907E-05 |
| 440, 445, 446, 450, 452, 455, 460, 477, 478, 479, 484, 486, 490, 498, 501, 505 | 1.03907E-05 |
| 440, 450, 452, 477, 478, 481, 484, 486, 498, 501, 505 | 1.03907E-05 |
| 440, 450, 452, 455, 456, 477, 478, 481, 484, 486, 498, 501, 505 | 1.03907E-05 |
| 450, 455, 460 | 1.03907E-05 |
| 440, 445, 446, 450, 452, 455, 460, 477, 478, 481, 483, 484, 485, 486, 498, 501, 505 | 1.03907E-05 |
| 440, 445, 446, 450, 477, 478, 481, 484, 486 | 1.03907E-05 |
| 445, 446, 450, 455, 460, 477, 478, 481, 484, 486, 498, 501 | 1.03907E-05 |
| 450, 455 | 1.03907E-05 |
| 440, 445, 446, 450, 452, 455, 460, 477, 478, 505 | 1.03907E-05 |
| 477, 478, 479, 481, 484, 486, 498, 501, 505 | 1.03907E-05 |
| 440, 445, 446, 450, 452, 455, 460, 477, 478, 481, 484, 486, 498, 501, 505, 507 | 1.03907E-05 |
| 440, 460, 477, 478 | 1.03907E-05 |
| 440, 445, 446, 450, 460 | 1.03907E-05 |
| 440, 445, 446, 450, 452, 455, 460, 477, 478, 481, 484, 493, 498, 501, 505 | 1.03907E-05 |
| 440, 446, 450, 452, 455, 460, 477, 478, 481, 484, 501, 505 | 1.03907E-05 |
| 460, 477 | 1.03907E-05 |
| 440, 445, 446, 450, 452, 477, 478, 481, 486, 498, 501, 505 | 1.03907E-05 |
| 477, 478, 501, 505 | 1.03907E-05 |
| 445, 446, 450, 452 | 1.03907E-05 |
| 440, 445, 446, 450, 452, 455, 460, 477, 478, 498 | 1.03907E-05 |
| 446, 450, 452, 455, 460, 477, 478, 481, 484, 501, 505 | 1.03907E-05 |
| 450, 452, 455, 460, 486, 498, 501, 505 | 1.03907E-05 |
| 440, 445, 446, 450, 452, 455, 456, 460, 477, 478, 481, 484, 486, 498, 501, 503, 505 | 1.03907E-05 |
| 440, 445, 446, 450, 460, 477, 478, 481, 484, 486, 498, 501 | 1.03907E-05 |
| 445, 446, 450, 452, 455, 460, 477, 478, 481, 484, 486, 501, 505 | 1.03907E-05 |
| 440, 450, 455, 460, 477, 478, 481, 484, 486, 498, 501 | 1.03907E-05 |
| 440, 445, 446, 450, 452, 455, 456, 460, 477, 478, 481, 484, 486, 501, 505 | 1.03907E-05 |
| 445, 446, 455, 498, 501, 505 | 1.03907E-05 |
| 446, 450, 452, 455, 460, 477, 478, 481, 484, 486, 498, 501, 505 | 1.03907E-05 |
| 440, 446, 450, 452, 455, 477, 478, 484, 486, 498, 501, 505 | 1.03907E-05 |
| 440, 446, 450, 452, 455, 460, 477, 478, 481, 484, 498, 501, 505 | 1.03907E-05 |
| 440, 445, 446, 450, 452, 455, 456, 460, 461, 477, 478, 481, 484, 486, 498, 501, 505 | 1.03907E-05 |
| 445, 446, 477, 478, 505 | 1.03907E-05 |
| 440, 445, 446, 450, 452, 455, 456, 460, 471, 477, 478, 481, 484, 486, 498, 501, 505 | 1.03907E-05 |
| 440, 445, 446, 452, 455, 460, 477, 478, 481, 484, 486, 498, 501 | 1.03907E-05 |
| 440, 445, 446, 450, 452, 455, 456, 460, 477, 478, 481, 485, 498, 501, 505 | 1.03907E-05 |
| 452, 455, 460, 477, 478 | 1.03907E-05 |
| 440, 445, 446, 450, 452, 455, 481, 484, 486, 498, 501, 505 | 1.03907E-05 |
| 450, 452, 455, 498, 501, 505 | 1.03907E-05 |
| 440, 445, 446, 450, 452, 455, 456, 460, 477, 478, 481, 484, 486, 498, 505 | 1.03907E-05 |
| 440, 450, 477, 478, 498, 501, 505 | 1.03907E-05 |
| 440, 455, 477, 478, 498, 501, 505 | 1.03907E-05 |
| 503, 505 | 1.03907E-05 |
| 446, 450, 452, 455, 477, 478, 481, 486, 498, 501, 505 | 1.03907E-05 |
| 445, 446, 450, 452, 455, 460, 477, 478, 498, 501, 505 | 1.03907E-05 |
| 440, 445, 446, 450, 452, 455, 460, 463, 477, 478, 481, 484, 486, 498, 501, 505 | 1.03907E-05 |
| 440, 445, 446, 450, 452, 455, 456, 460, 477, 478, 481, 486 | 1.03907E-05 |
| 440, 445, 446, 450, 452, 455, 456, 460, 477, 478, 481, 484, 486, 498, 499, 501, 505 | 1.03907E-05 |
| 452, 455, 456, 460, 477, 478, 481, 484, 486 | 1.03907E-05 |
| 455, 456, 460, 477, 478, 481, 484, 486, 498, 501 | 1.03907E-05 |
| 450, 452, 455, 456, 460, 477, 478, 481, 484, 486 | 1.03907E-05 |
| 440, 450, 452, 455, 456, 498, 501, 505 | 1.03907E-05 |
| 440, 460, 477, 478, 481, 484, 486, 498, 501 | 1.03907E-05 |
| 440, 446, 450, 455, 460, 477, 478, 481, 484, 486, 498, 501, 505 | 1.03907E-05 |
| 440, 445, 446, 450, 452, 455, 456, 477, 478, 481, 484, 486, 498, 501, 505 | 1.03907E-05 |
| 440, 450, 452, 455, 456, 460, 477, 478, 481, 484, 486, 498 | 1.03907E-05 |
| 440, 450, 452, 460, 477, 478, 498, 501, 505 | 1.03907E-05 |
| 440, 450, 455, 456, 460, 477, 478, 481, 484, 486, 498, 501, 503, 505 | 1.03907E-05 |
| 440, 445, 446, 450, 452, 455, 456, 477, 478, 486, 498, 501, 505 | 1.03907E-05 |
| 440, 441, 445, 446, 450, 452, 460, 477, 478, 481, 484, 486, 498, 501, 505 | 1.03907E-05 |
| 455, 456, 460, 477, 481, 484, 486, 498, 501, 505 | 1.03907E-05 |
| 440, 450, 455, 456, 460, 477, 478, 481, 484, 486, 498, 501, 505 | 1.03907E-05 |
| 440, 450, 456, 460, 477, 478 | 1.03907E-05 |
| 445, 446, 450, 452, 455, 456, 460, 477, 478, 498, 501, 505 | 1.03907E-05 |
| 460, 477, 481, 484, 486, 498, 501, 505 | 1.03907E-05 |
| 440, 446, 450, 452, 455, 456, 460, 477, 478, 498, 501, 505 | 1.03907E-05 |
| 460, 477, 478, 481, 498 | 1.03907E-05 |
| 440, 445, 446, 450, 455, 456, 460, 498, 501, 505 | 1.03907E-05 |
| 455, 456, 460, 477, 478, 481, 484, 486 | 1.03907E-05 |
| 440, 455, 460, 477, 478, 486, 498, 501, 505 | 1.03907E-05 |
| 440, 481, 484, 486, 498, 501, 505 | 1.03907E-05 |
| 455, 477, 478, 481, 486, 498, 501, 505 | 1.03907E-05 |
| 440, 445, 446, 450, 452, 455, 456, 460, 473, 477, 478, 481, 484, 486, 498, 501, 505 | 1.03907E-05 |
| 440, 445, 446, 450, 455, 456, 460, 477, 478, 498, 501, 505 | 1.03907E-05 |
| 445, 446, 455, 456, 460, 477, 478, 481, 484, 486, 498, 501, 505 | 1.03907E-05 |
| 440, 450, 452 | 1.03907E-05 |
| 440, 445, 446, 455, 456, 460, 477, 478, 498, 501, 505 | 1.03907E-05 |
| 440, 441, 445, 446, 450, 452, 455, 456, 460, 477, 478, 481, 484, 486 | 1.03907E-05 |
| 440, 445, 446, 450, 498, 501, 505 | 1.03907E-05 |
| 450, 456, 460, 477, 478 | 1.03907E-05 |
| 440, 445, 446, 450, 452, 455, 456, 460, 498, 501, 505 | 1.03907E-05 |
| 456, 460, 477, 478, 481, 486, 498, 501, 505 | 1.03907E-05 |
| 446, 450, 452 | 1.03907E-05 |
| 445, 446, 498, 501, 505 | 1.03907E-05 |
| 440, 452, 455, 456, 460, 498, 501, 505 | 1.03907E-05 |
| 440, 456, 460, 477, 478, 481, 484, 486, 498, 501, 505 | 1.03907E-05 |
| 440, 445, 446, 450, 452, 455, 456, 460, 477, 478 | 1.03907E-05 |
| 440, 452, 455, 456, 460, 477, 478, 498, 501, 505 | 1.03907E-05 |
| 440, 445, 446, 450, 452, 455, 456, 460, 477, 478, 486, 498, 501, 505 | 1.03907E-05 |
| 460, 477, 478, 481, 486, 498, 501, 505 | 1.03907E-05 |
| 455, 456, 460, 477, 478, 481 | 1.03907E-05 |
| 440, 450, 455, 456, 460 | 1.03907E-05 |
| 450, 477, 478, 481, 498, 501, 505 | 1.03907E-05 |
| 460, 477, 498, 501, 505 | 1.03907E-05 |
| 460, 477, 478, 498, 501 | 1.03907E-05 |
| 440, 445, 446, 450, 452, 455, 456, 460, 477, 478, 481, 484, 486, 490, 498, 501, 505 | 1.03907E-05 |
| 440, 441, 445, 446, 450, 452, 455, 456, 460 | 1.03907E-05 |
| 450, 455, 456, 460, 477, 478, 481, 484, 486 | 1.03907E-05 |
| 440, 455, 456, 460, 477, 478, 481, 484, 486, 498, 501, 505 | 1.03907E-05 |
| 460, 477, 478, 486, 498, 501 | 1.03907E-05 |
| 460, 477, 478, 481, 484, 486, 505 | 1.03907E-05 |
| 440, 450, 452, 455, 456, 460, 477, 478, 481, 484, 486, 498, 501 | 1.03907E-05 |
| 440, 446, 477, 478, 484, 498, 501, 505 | 1.03907E-05 |
| 440, 445, 446, 450, 452, 455, 456, 460, 474, 477, 478, 481, 484, 486, 498, 501, 505 | 1.03907E-05 |
| 440, 445, 446, 450, 452, 455, 456, 460, 477, 478, 481, 484, 486, 498, 501, 504, 505 | 1.03907E-05 |
| 452, 455, 456, 460, 477, 478, 481, 484, 486, 498, 501, 505 | 1.03907E-05 |
| 440, 445, 450, 452, 455, 456, 460, 477, 478, 481, 484, 486, 493, 498, 501, 505 | 1.03907E-05 |
| 440, 446, 450, 452, 455, 460, 477, 478, 481, 484, 486, 501, 505 | 1.03907E-05 |
| 455, 456, 460, 477, 478, 481, 498, 501, 505 | 1.03907E-05 |
| 440, 445, 446, 450, 452, 455, 456, 460, 462, 477, 478, 481, 484, 486 | 1.03907E-05 |
| 440, 445, 446, 450, 452, 455, 456, 460, 470, 477, 478, 481, 484, 486, 498, 501, 505 | 1.03907E-05 |
| 440, 452, 455, 456, 460, 477, 478, 481, 484, 486, 498 | 1.03907E-05 |
| 440, 445, 446, 450, 452, 455, 456, 460, 477, 478, 498, 501 | 1.03907E-05 |
| **Training set 2** | |
| **mutation combination** | **frequency** |
| 440, 445, 446, 450, 452, 455, 460, 477, 478, 481, 484, 486, 498, 501, 505 | 0.740295096 |
| 440, 445, 446, 450, 452, 455, 456, 460, 477, 478, 481, 484, 486, 498, 501, 505 | 0.182689111 |
| N/A | 0.033686617 |
| 477, 478, 481, 484, 486, 498, 501, 505 | 0.006494181 |
| 440, 445, 446, 450, 452, 455, 460, 477, 478, 481, 484, 486, 498, 501 | 0.004280964 |
| 440, 445, 446, 450, 452, 455, 460, 477, 478, 481, 486, 498, 501, 505 | 0.001818371 |
| 440, 445, 446, 450, 452, 455, 460, 477, 478, 481, 484, 486 | 0.001724855 |
| 440, 445, 446, 450, 452, 460, 477, 478, 481, 484, 486, 498, 501, 505 | 0.001704073 |
| 498, 501, 505 | 0.001381962 |
| 440, 445, 446, 450, 452, 455, 460, 477, 478, 498, 501, 505 | 0.000997506 |
| 440, 441, 445, 446, 450, 452, 455, 460, 477, 478, 481, 484, 486, 498, 501, 505 | 0.000924771 |
| 440, 444, 445, 446, 450, 452, 455, 460, 477, 478, 481, 484, 486, 498, 501, 505 | 0.000696176 |
| 440, 445, 446, 450, 452, 455, 456, 460, 477, 481, 484, 486, 498, 501, 505 | 0.000613051 |
| 440, 441, 445, 446, 450, 452, 455, 456, 460, 477, 478, 481, 484, 486, 498, 501, 505 | 0.000550707 |
| 440, 445, 446, 450, 452, 455, 456, 460, 475, 477, 478, 481, 484, 486, 498, 501, 505 | 0.000540316 |
| 440, 445, 446, 450, 452, 455, 460, 477, 478, 486, 498, 501, 505 | 0.000509144 |
| 440, 445, 446, 450, 452, 455, 460, 477, 478, 481, 483, 484, 486, 498, 501, 505 | 0.000498753 |
| 440, 445, 446, 450, 452, 455, 460, 477, 478, 484, 486, 490, 498, 501, 505 | 0.000477972 |
| 440, 445, 446, 450, 452, 455, 456, 460, 477, 478, 481, 484, 486 | 0.000477972 |
| 460, 477, 478, 481, 484, 486, 498, 501, 505 | 0.00045719 |
| 440, 445, 446, 450, 452, 455, 460, 475, 477, 478, 481, 484, 486, 498, 501, 505 | 0.000426018 |
| 440, 445, 446, 450, 452, 455, 460, 477, 478, 484, 486, 498, 501, 505 | 0.000415628 |
| 477, 478, 486, 498, 501, 505 | 0.000415628 |
| 440, 446, 450, 452, 455, 460, 477, 478, 481, 484, 486, 498, 501, 505 | 0.000405237 |
| 505 | 0.000405237 |
| 440, 445, 446, 450, 452, 455, 456, 460, 477, 478, 481, 484, 486, 498, 501 | 0.000384456 |
| 440, 445, 446, 450, 452, 455, 460, 477, 478, 481 | 0.000363674 |
| 440, 445, 446, 450, 455, 460, 477, 478, 481, 484, 486, 498, 501, 505 | 0.000353283 |
| 440, 445, 446, 450, 452, 455, 460, 477, 478, 481, 484, 498, 501, 505 | 0.000342893 |
| 440, 445, 446, 450, 452, 455, 477, 478, 481, 484, 486, 498, 501, 505 | 0.000332502 |
| 440, 445, 446, 450, 452, 455, 460, 477, 481, 484, 486, 498, 501, 505 | 0.000322111 |
| 450, 452, 455, 460, 477, 478, 481, 484, 486, 498, 501, 505 | 0.000311721 |
| 440, 450, 452, 455, 460, 477, 478, 481, 484, 486, 498, 501, 505 | 0.00030133 |
| 440, 444, 445, 446, 450, 452, 453, 455, 460, 477, 478, 481, 484, 486, 498, 501, 505 | 0.00030133 |
| 477, 478, 498, 501, 505 | 0.000290939 |
| 440, 445, 446, 450, 452, 455, 460 | 0.000280549 |
| 440, 445, 446, 450, 452, 455, 460, 498, 501, 505 | 0.000270158 |
| 445, 446, 450, 452, 455, 460, 477, 478, 481, 484, 486, 498, 501, 505 | 0.000270158 |
| 440, 445, 446, 450, 452, 455, 460, 477, 478, 482, 483, 484, 486, 498, 501, 505 | 0.000259767 |
| 440, 445, 446, 450, 452, 455, 460, 477, 478, 479, 481, 484, 486, 498, 501, 505 | 0.000238986 |
| 440, 445, 446, 450, 452, 455, 456, 460, 477, 478, 481, 484, 486, 493, 498, 501, 505 | 0.000238986 |
| 440, 445, 446, 450, 452, 455, 456, 460, 477, 478, 481 | 0.000228595 |
| 440, 445, 446, 450, 452, 455, 460, 477, 478, 481, 498, 501, 505 | 0.000218204 |
| 460, 477, 478, 481, 484, 486 | 0.000218204 |
| 440, 477, 478, 481, 484, 486, 498, 501, 505 | 0.000207814 |
| 440, 460 | 0.000207814 |
| 477, 478, 481, 484, 486 | 0.000207814 |
| 440, 445, 446, 455, 460, 477, 478, 481, 484, 486, 498, 501, 505 | 0.000166251 |
| 440, 445, 446, 450, 452, 455, 460, 477, 478, 482, 486, 498, 501, 505 | 0.00015586 |
| 440, 445, 446, 450, 452, 455, 460, 477, 478, 481, 484, 486, 498, 500, 501, 505 | 0.00015586 |
| 477, 478 | 0.00014547 |
| 440, 445, 446, 450, 452, 455, 460, 476, 477, 478, 481, 484, 486, 498, 501, 505 | 0.00014547 |
| 498, 501 | 0.00014547 |
| 455, 460, 477, 478, 481, 484, 486, 498, 501, 505 | 0.00014547 |
| 440, 445, 446, 450, 452, 455, 456, 460, 477, 478, 498, 501, 505 | 0.000135079 |
| 440, 460, 498 | 0.000135079 |
| 440, 445, 446, 450, 452, 455, 460, 477, 478, 481, 484, 486, 498 | 0.000114298 |
| 440, 445, 446, 477, 478, 481, 484, 486, 498, 501, 505 | 0.000114298 |
| 501, 505 | 0.000114298 |
| 440, 445, 446, 450, 452, 455, 460, 477, 478, 482, 484, 486, 498, 501, 505 | 0.000114298 |
| 440, 445, 446, 450, 455, 456, 460, 477, 478, 481, 498, 501, 505 | 0.000114298 |
| 440, 444, 445, 446, 450, 452, 455, 456, 460, 477, 478, 481, 484, 486, 498, 501, 505 | 0.000114298 |
| 440, 445, 446, 450, 452, 455, 460, 477, 478 | 0.000103907 |
| 450, 452, 455, 456, 460, 477, 478, 481, 484, 486, 498, 501, 505 | 0.000103907 |
| 440, 445, 446, 450, 452, 455, 460, 477, 478, 481, 484, 486, 498, 501, 503, 505 | 9.35162E-05 |
| 460 | 9.35162E-05 |
| 440, 445, 446, 450, 460, 477, 478, 481, 484, 486, 498, 501, 505 | 9.35162E-05 |
| 501 | 9.35162E-05 |
| 440, 445, 446, 450, 452, 455, 456, 460, 477, 478, 481, 484, 498, 501, 505 | 9.35162E-05 |
| 440, 445, 446, 450, 452, 455, 456, 460, 477, 478, 481, 486, 498, 501, 505 | 9.35162E-05 |
| 440, 445, 446, 477, 478, 486, 498, 501, 505 | 8.31255E-05 |
| 440, 445, 446, 450, 452, 455, 460, 470, 477, 478, 481, 484, 486, 498, 501, 505 | 8.31255E-05 |
| 440, 460, 477, 478, 481, 484, 486, 498, 501, 505 | 8.31255E-05 |
| 440, 445, 446, 450, 452, 455, 460, 477, 478, 481, 484, 485, 486, 498, 501, 505 | 8.31255E-05 |
| 440, 445, 446, 450, 452, 455, 456, 460, 477, 478, 481, 484, 486, 498, 500, 501, 505 | 8.31255E-05 |
| 450, 477, 478, 481, 484, 486, 498, 501, 505 | 7.27348E-05 |
| 440, 455, 460, 477, 478, 481, 484, 486, 498, 501, 505 | 7.27348E-05 |
| 498 | 7.27348E-05 |
| 440, 445, 446, 450, 452, 455, 477, 478, 498, 501, 505 | 7.27348E-05 |
| 440 | 7.27348E-05 |
| 440, 445, 446, 450, 452, 455, 458, 460, 477, 478, 481, 484, 486, 498, 501, 505 | 7.27348E-05 |
| 440, 460, 498, 505 | 7.27348E-05 |
| 455, 460, 477, 478, 498, 501, 505 | 6.23441E-05 |
| 440, 445, 446, 450, 452, 455, 498, 501, 505 | 6.23441E-05 |
| 455, 460, 477, 478, 481, 484, 486, 498, 501 | 6.23441E-05 |
| 455, 460, 477, 478, 481, 484, 486 | 6.23441E-05 |
| 440, 445, 446, 450, 452, 455, 460, 462, 477, 478, 481, 484, 486, 498, 501, 505 | 6.23441E-05 |
| 460, 477, 478 | 6.23441E-05 |
| 440, 445, 446, 450, 452, 455, 460, 477, 478, 481, 484, 486, 501, 505 | 6.23441E-05 |
| 455, 498, 501, 505 | 6.23441E-05 |
| 440, 446, 450, 452, 455, 456, 460, 477, 478, 481, 484, 486, 498, 501, 505 | 6.23441E-05 |
| 440, 445, 446, 450, 452, 455, 456, 460, 477, 478, 482, 483, 484, 486, 498, 501, 505 | 6.23441E-05 |
| 440, 445, 446, 450, 455, 460, 477, 478, 486, 498, 501, 505 | 5.19534E-05 |
| 460, 477, 478, 481 | 5.19534E-05 |
| 440, 445, 477, 478, 481, 484, 486, 498, 501, 505 | 5.19534E-05 |
| 440, 445, 446, 450, 452, 455, 460, 477, 478, 483, 484, 486, 498, 501, 505 | 5.19534E-05 |
| 440, 445, 446, 450, 452, 477, 478, 481, 484, 486, 498, 501, 505 | 5.19534E-05 |
| 440, 445, 446, 452, 455, 460, 477, 478, 481, 484, 486, 498, 501, 505 | 5.19534E-05 |
| 450, 455, 460, 477, 478, 481, 484, 486, 498, 501, 505 | 5.19534E-05 |
| 440, 445, 446, 460, 477, 478, 481, 484, 486, 498, 501, 505 | 5.19534E-05 |
| 440, 450, 460, 477, 478, 481, 484, 486, 498, 501, 505 | 5.19534E-05 |
| 477, 478, 481 | 5.19534E-05 |
| 440, 460, 505 | 5.19534E-05 |
| 445, 446, 450, 452, 455, 456, 460, 477, 478, 481, 484, 486, 498, 501, 505 | 5.19534E-05 |
| 440, 445, 446, 450, 452, 455, 456, 459, 460, 477, 478, 481, 484, 486, 498, 501, 505 | 5.19534E-05 |
| 440, 445, 446, 460, 477, 478, 486, 498, 501, 505 | 4.15628E-05 |
| 445, 446, 450, 452, 455, 460, 477, 478, 481, 484, 486 | 4.15628E-05 |
| 450, 455, 498, 501, 505 | 4.15628E-05 |
| 440, 445, 446, 450, 452, 455 | 4.15628E-05 |
| 477, 478, 481, 498, 501, 505 | 4.15628E-05 |
| 440, 445, 446, 450, 452, 455, 460, 477, 478, 481, 484, 486, 487, 498, 501, 505 | 4.15628E-05 |
| 460, 477, 478, 498, 501, 505 | 4.15628E-05 |
| 477, 478, 481, 484, 486, 498, 501 | 4.15628E-05 |
| 440, 450, 455, 460, 477, 478, 481, 484, 486, 498, 501, 505 | 4.15628E-05 |
| 440, 445, 446, 450, 452, 498, 501, 505 | 4.15628E-05 |
| 440, 445, 446, 450, 455, 460, 477, 478, 484, 486, 498, 501, 505 | 4.15628E-05 |
| 440, 445, 446, 450, 452, 455, 460, 477, 478, 481, 484, 486, 494, 498, 501, 505 | 4.15628E-05 |
| 440, 450, 455, 460, 477, 478, 498, 501, 505 | 4.15628E-05 |
| 450, 452, 455, 460, 477, 478, 498, 501, 505 | 4.15628E-05 |
| 440, 445, 446, 450, 452, 455, 460, 477, 498, 501, 505 | 4.15628E-05 |
| 440, 445, 446, 450, 452, 456, 460, 477, 478, 481, 484, 486, 498, 501, 505 | 4.15628E-05 |
| 440, 445, 446, 450, 452, 455, 456, 460, 477, 478, 481, 498, 501, 505 | 4.15628E-05 |
| 440, 445, 446, 450, 452, 455, 460, 477, 478, 484, 498, 501, 505 | 4.15628E-05 |
| 440, 450, 452, 455, 456, 460, 477, 478, 481, 484, 486, 498, 501, 505 | 4.15628E-05 |
| 440, 445, 446, 450, 452, 455, 456, 460, 477, 478, 484, 486, 498, 501, 505 | 4.15628E-05 |
| 440, 445, 446, 450, 460, 477, 478, 486, 498, 501, 505 | 3.11721E-05 |
| 440, 445, 460, 477, 478, 481, 484, 486, 498, 501, 505 | 3.11721E-05 |
| 440, 445, 446, 456, 460, 477, 478, 484, 486, 490, 498, 501, 505 | 3.11721E-05 |
| 440, 445, 446, 452, 460, 477, 478, 484, 486, 498, 501, 505 | 3.11721E-05 |
| 440, 445, 446, 452, 456, 460, 477, 478, 484, 486, 490, 498, 501, 505 | 3.11721E-05 |
| 440, 460, 477, 478, 486, 498, 501, 505 | 3.11721E-05 |
| 455, 460, 498, 501, 505 | 3.11721E-05 |
| 440, 445, 446, 450, 452, 455, 459, 460, 477, 478, 481, 484, 486, 498, 501, 505 | 3.11721E-05 |
| 440, 445, 446, 450, 452, 498, 501 | 3.11721E-05 |
| 440, 445, 446, 450, 452, 455, 456, 460, 477, 478, 484, 486, 490, 498, 501, 505 | 3.11721E-05 |
| 455 | 3.11721E-05 |
| 440, 450, 452, 455, 460, 477, 478 | 3.11721E-05 |
| 450, 452, 455, 460, 477, 478, 481, 484, 486 | 3.11721E-05 |
| 440, 445, 446, 450, 477, 478, 481, 484, 486, 498, 501, 505 | 3.11721E-05 |
| 477, 498, 501, 505 | 3.11721E-05 |
| 440, 445, 446, 450, 452, 455, 460, 474, 477, 478, 481, 484, 486, 498, 501, 505 | 3.11721E-05 |
| 440, 450, 477, 478, 481, 484, 486, 498, 501, 505 | 3.11721E-05 |
| 450, 452, 455, 460, 477, 478, 481, 484, 486, 498, 501 | 3.11721E-05 |
| 440, 443, 445, 446, 450, 452, 455, 460, 477, 478, 481, 484, 486, 498, 501, 505 | 3.11721E-05 |
| 440, 450, 452, 455, 460, 477, 478, 481, 484, 486, 498, 501 | 3.11721E-05 |
| 450, 452, 455, 460, 477, 478 | 3.11721E-05 |
| 440, 445, 446, 450, 452, 455, 456, 460, 477, 478, 481, 483, 484, 486, 498, 501, 505 | 3.11721E-05 |
| 450, 455, 460, 477, 478, 498, 501, 505 | 3.11721E-05 |
| 440, 445, 446, 450, 452, 477, 478, 498, 501, 505 | 3.11721E-05 |
| 440, 445, 446, 450, 452, 477, 478, 486, 498, 501, 505 | 3.11721E-05 |
| 440, 445, 446, 450, 452, 453, 455, 460, 477, 478, 481, 484, 486, 498, 501, 505 | 3.11721E-05 |
| 440, 445, 446, 450, 452, 455, 456, 460, 476, 477, 478, 481, 484, 486, 498, 501, 505 | 3.11721E-05 |
| 440, 445, 446, 450, 452, 455, 456, 460, 477, 478, 481, 484, 485, 486, 498, 501, 505 | 3.11721E-05 |
| 440, 445, 446, 455, 456, 460, 477, 478, 484, 486, 490, 498, 501, 505 | 2.07814E-05 |
| 455, 460, 477, 478 | 2.07814E-05 |
| 440, 445, 446, 450, 452, 455, 460, 505 | 2.07814E-05 |
| 440, 455, 460, 477, 478, 498, 501, 505 | 2.07814E-05 |
| 440, 445, 446, 455, 460, 477, 478, 484, 486, 490, 498, 501, 505 | 2.07814E-05 |
| 440, 445, 446, 455, 456, 460, 477, 478, 481, 484, 486, 498, 501, 505 | 2.07814E-05 |
| 450, 452, 455, 477, 478, 481, 484, 486, 498, 501, 505 | 2.07814E-05 |
| 440, 445, 446, 450, 455, 460, 477, 478, 481, 484, 486, 498, 501 | 2.07814E-05 |
| 477, 478, 484, 486, 498, 501, 505 | 2.07814E-05 |
| 440, 445, 446, 450, 477, 478, 486, 498, 501, 505 | 2.07814E-05 |
| 481, 484, 486, 498, 501, 505 | 2.07814E-05 |
| 440, 445, 446, 460, 477, 478, 484, 486, 498, 501, 505 | 2.07814E-05 |
| 440, 445, 446, 450, 452, 460, 477, 478, 486, 498, 501, 505 | 2.07814E-05 |
| 486, 498, 501, 505 | 2.07814E-05 |
| 440, 445, 446, 450, 452, 455, 460, 477, 478, 498, 501 | 2.07814E-05 |
| 450, 452 | 2.07814E-05 |
| 440, 445, 446, 450, 452, 455, 460, 477, 478, 481, 484, 486, 502, 503 | 2.07814E-05 |
| 460, 498, 501, 505 | 2.07814E-05 |
| 460, 477, 478, 481, 484, 486, 498, 501 | 2.07814E-05 |
| 440, 445, 446, 452, 460, 477, 478, 486, 498, 501, 505 | 2.07814E-05 |
| 440, 450, 455, 460, 477, 478, 481, 484, 486, 498 | 2.07814E-05 |
| 440, 445, 446, 450, 452, 455, 460, 477, 478, 501, 505 | 2.07814E-05 |
| 450, 452, 455, 460, 477, 478, 481 | 2.07814E-05 |
| 455, 477, 478, 481, 484, 486, 498, 501, 505 | 2.07814E-05 |
| 440, 450, 452, 460, 477, 478 | 2.07814E-05 |
| 450, 460, 477, 478 | 2.07814E-05 |
| 445, 446, 450, 452, 455, 477, 478, 481, 484, 486, 498, 501, 505 | 2.07814E-05 |
| 440, 445, 446, 450, 452, 455, 460, 477, 478, 481, 484, 486, 505 | 2.07814E-05 |
| 450, 460, 477, 478, 481, 484, 486, 498, 501, 505 | 2.07814E-05 |
| 440, 445, 450, 452, 455, 460, 477, 478, 481, 484, 486, 498, 501, 505 | 2.07814E-05 |
| 450, 452, 477, 478, 481, 484, 486, 498, 501, 505 | 2.07814E-05 |
| 440, 446, 450, 452, 455, 460, 477, 478, 498, 501, 505 | 2.07814E-05 |
| 445, 446, 450, 452, 455, 460, 477, 478, 481, 498, 501, 505 | 2.07814E-05 |
| 477, 478, 498, 501 | 2.07814E-05 |
| 440, 445, 446, 450, 452, 455, 460, 477, 478, 481, 484, 486, 501 | 2.07814E-05 |
| 440, 498, 501, 505 | 2.07814E-05 |
| 440, 445, 446, 450, 452, 455, 460, 486, 498, 501, 505 | 2.07814E-05 |
| 455, 460 | 2.07814E-05 |
| 455, 460, 477, 478, 481, 498, 501, 505 | 2.07814E-05 |
| 440, 445, 446, 450, 452, 455, 460, 477, 478, 481, 485, 498, 501, 505 | 2.07814E-05 |
| 460, 498 | 2.07814E-05 |
| 440, 450, 455, 477, 478, 481, 484, 486, 498, 501, 505 | 2.07814E-05 |
| 477, 478, 481, 486, 498, 501, 505 | 2.07814E-05 |
| 477, 478, 501 | 2.07814E-05 |
| 440, 445, 446, 450, 452, 455, 460, 477, 478, 481, 484, 486, 498, 501, 505, 508 | 2.07814E-05 |
| 440, 450, 452, 455, 477, 478, 481, 484, 486, 498, 501, 505 | 2.07814E-05 |
| 440, 445, 446, 450, 452, 460, 477, 478, 481, 484, 498, 501, 505 | 2.07814E-05 |
| 440, 445, 446, 450, 452, 455, 477, 478, 486, 498, 501, 505 | 2.07814E-05 |
| 445, 446, 450, 452, 498, 501, 505 | 2.07814E-05 |
| 440, 445, 446, 450, 452, 477, 478, 484, 486, 498, 501, 505 | 2.07814E-05 |
| 450, 452, 455, 460, 498, 501, 505 | 2.07814E-05 |
| 440, 445, 446, 450, 452, 455, 460, 477, 478, 481, 484, 486, 498, 501, 504, 505 | 2.07814E-05 |
| 440, 477, 478, 498, 501, 505 | 2.07814E-05 |
| 477, 478, 481, 484, 498, 501, 505 | 2.07814E-05 |
| 440, 445, 446, 450, 452, 455, 460, 477, 478, 481, 484, 486, 493, 498, 501, 505 | 2.07814E-05 |
| 450, 452, 455, 456, 460, 477, 478, 498, 501, 505 | 2.07814E-05 |
| 440, 445, 446, 450, 452, 455, 456, 460, 477, 478, 482, 484, 486, 498, 501, 505 | 2.07814E-05 |
| 455, 456, 460 | 2.07814E-05 |
| 455, 456, 460, 477, 478, 481, 484, 486, 498, 501, 505 | 2.07814E-05 |
| 440, 445, 446, 455, 456, 460, 477, 478, 481, 498, 501, 505 | 2.07814E-05 |
| 440, 445, 446, 450, 452, 453, 455, 456, 460, 477, 478, 481, 484, 486, 498, 501, 505 | 2.07814E-05 |
| 440, 445, 446, 450, 452, 455, 456, 498, 501, 505 | 2.07814E-05 |
| 440, 445, 446, 450, 452, 455, 456, 460, 468, 477, 478, 481, 484, 486, 498, 501, 505 | 2.07814E-05 |
| 450, 452, 455, 456, 498, 501, 505 | 2.07814E-05 |
| 440, 445, 446, 450, 452, 455, 456, 460, 477, 498, 501, 505 | 2.07814E-05 |
| 440, 445, 446, 450, 455, 456, 460, 477, 478, 481, 484, 486, 498, 501, 505 | 2.07814E-05 |
| 456, 460, 477, 478, 481, 484, 486, 498, 501, 505 | 2.07814E-05 |
| 440, 445, 446, 450, 452, 455, 456, 460, 477, 478, 479, 481, 484, 486, 498, 501, 505 | 2.07814E-05 |
| 440, 445, 446, 450, 452, 460, 477, 478, 498, 501, 505 | 1.03907E-05 |
| 440, 445, 446, 456, 460, 484, 486, 490, 498, 501, 505 | 1.03907E-05 |
| 440, 452, 460, 477, 478, 481, 484, 486 | 1.03907E-05 |
| 440, 445, 446, 450, 452, 455, 460, 477, 478, 481, 484, 501, 505 | 1.03907E-05 |
| 440, 445, 446, 477, 486, 498, 501, 505 | 1.03907E-05 |
| 440, 445, 446, 450, 452, 455, 460, 498, 501 | 1.03907E-05 |
| 440, 445, 477 | 1.03907E-05 |
| 440, 445, 446, 460, 477, 478, 501, 505 | 1.03907E-05 |
| 440, 450, 460, 477, 478, 486, 498, 501, 505 | 1.03907E-05 |
| 445, 446, 450, 452, 455, 460, 477, 478, 481, 484, 486, 498 | 1.03907E-05 |
| 440, 445, 446, 477, 478, 498, 501, 505 | 1.03907E-05 |
| 440, 450, 452, 455, 460, 477, 478, 481, 484, 486 | 1.03907E-05 |
| 440, 445, 446, 477, 498, 501, 505 | 1.03907E-05 |
| 455, 456, 475, 477, 478, 484, 486, 490 | 1.03907E-05 |
| 440, 441, 445, 446, 450, 455, 460, 477, 478, 481, 484, 486, 498, 501, 505 | 1.03907E-05 |
| 440, 445, 446, 450, 452, 455, 460, 475, 477, 478, 498, 501, 505 | 1.03907E-05 |
| 440, 445, 446, 450, 452, 455, 477, 498, 501, 505 | 1.03907E-05 |
| 450, 455, 477, 478, 481, 484, 486, 498, 501, 505 | 1.03907E-05 |
| 440, 445, 446, 452, 477, 478, 481, 484, 486, 498, 501, 505 | 1.03907E-05 |
| 440, 450, 455, 498, 501, 505 | 1.03907E-05 |
| 440, 445, 446, 452, 455, 460, 477, 478, 484, 486, 498, 501, 505 | 1.03907E-05 |
| 440, 445, 446, 450, 477, 486, 498, 501, 505 | 1.03907E-05 |
| 452, 455, 460, 477, 478, 481, 484, 486 | 1.03907E-05 |
| 440, 445, 446, 452, 455, 477, 478, 481, 484, 486, 498, 501, 505 | 1.03907E-05 |
| 440, 455, 460, 477, 478, 481, 486, 498, 501, 505 | 1.03907E-05 |
| 450, 452, 455, 498, 501 | 1.03907E-05 |
| 440, 445, 446, 450, 452, 455, 475, 477, 478, 481, 484, 486 | 1.03907E-05 |
| 440, 452, 455, 460, 477, 478, 481, 484, 486 | 1.03907E-05 |
| 440, 445, 446, 450, 455, 460, 477, 478, 481, 484, 486 | 1.03907E-05 |
| 440, 445, 446, 450, 455, 477, 478, 486, 498, 501, 505 | 1.03907E-05 |
| 440, 445, 446, 460, 477, 478, 484, 486, 490, 498, 501, 505 | 1.03907E-05 |
| 450, 455, 460, 477, 478 | 1.03907E-05 |
| 440, 445, 446, 450, 455, 460, 477, 478, 481, 484, 486, 501, 505 | 1.03907E-05 |
| 440, 446, 455, 460, 477, 478, 481, 484, 486, 498, 501 | 1.03907E-05 |
| 476, 477, 478, 481, 484, 486, 498, 501, 505 | 1.03907E-05 |
| 440, 445, 446, 450, 452, 455, 476, 477, 478, 481, 484, 486, 498, 501, 505 | 1.03907E-05 |
| 440, 445, 446, 456, 460 | 1.03907E-05 |
| 440, 450, 477, 478, 481, 484, 486 | 1.03907E-05 |
| 450, 455, 477, 498, 501, 505 | 1.03907E-05 |
| 440, 445, 446, 450, 452, 455, 460, 478, 484, 486, 498, 501, 505 | 1.03907E-05 |
| 440, 445, 446, 450, 452, 455, 477, 478, 481, 498, 501, 505 | 1.03907E-05 |
| 440, 445, 446, 450, 455, 460, 498, 501, 505 | 1.03907E-05 |
| 460, 477, 478, 484, 498 | 1.03907E-05 |
| 477, 505 | 1.03907E-05 |
| 440, 445, 446, 450, 452, 455, 460, 477, 478, 481, 484, 486, 498, 501, 507 | 1.03907E-05 |
| 440, 446, 450, 452, 455, 460, 477, 478, 481 | 1.03907E-05 |
| 440, 446, 477, 478, 486, 498, 501, 505 | 1.03907E-05 |
| 440, 445, 446, 450, 452, 455, 460, 477, 478, 479, 498, 501, 505 | 1.03907E-05 |
| 455, 460, 477, 478, 481, 498 | 1.03907E-05 |
| 440, 445, 446, 450, 452, 455, 460, 507 | 1.03907E-05 |
| 440, 445, 446, 450, 452, 460, 477, 478, 481 | 1.03907E-05 |
| 484 | 1.03907E-05 |
| 477, 478, 481, 484, 486, 498, 505 | 1.03907E-05 |
| 440, 445, 446, 456, 460, 477, 478, 484, 486, 498, 501, 505 | 1.03907E-05 |
| 450, 452, 455, 460 | 1.03907E-05 |
| 440, 445, 446, 450, 452 | 1.03907E-05 |
| 507 | 1.03907E-05 |
| 446, 450, 452, 455, 460, 477, 478, 498, 501, 505 | 1.03907E-05 |
| 440, 446, 450, 452, 455, 460, 477, 478, 484, 486, 490, 498, 501, 505 | 1.03907E-05 |
| 440, 445, 446, 452, 477, 486, 498, 501, 505 | 1.03907E-05 |
| 440, 445, 446, 450, 455, 460, 477, 478, 481 | 1.03907E-05 |
| 440, 445, 446, 452, 455, 477, 478, 481, 486, 498, 501, 505 | 1.03907E-05 |
| 440, 450, 452, 455, 477, 478, 498, 501, 505 | 1.03907E-05 |
| 440, 445, 446, 452, 455, 460, 477, 478, 486, 498, 501, 505 | 1.03907E-05 |
| 477, 478, 498 | 1.03907E-05 |
| 477, 478, 481, 501 | 1.03907E-05 |
| 450, 452, 455, 460, 498, 501 | 1.03907E-05 |
| 440, 445, 446, 450, 460, 477, 478, 481, 486, 498, 501, 505 | 1.03907E-05 |
| 445, 446, 450, 452, 455, 477, 478, 498, 501, 505 | 1.03907E-05 |
| 440, 446, 460, 477, 478, 484, 486, 498, 501, 505 | 1.03907E-05 |
| 440, 445, 450, 452, 455, 498, 501, 505 | 1.03907E-05 |
| 440, 450, 452, 455, 460, 477, 478, 498, 501, 505 | 1.03907E-05 |
| 445, 446, 450, 452, 460, 477, 478, 481, 484, 486, 498, 501, 505 | 1.03907E-05 |
| 445, 446, 452, 455, 460, 477, 478, 481, 484, 486, 498, 501, 505 | 1.03907E-05 |
| 477, 478, 481, 484, 486, 501 | 1.03907E-05 |
| 440, 445, 446, 450, 460, 477, 478, 484, 486, 498, 501, 505 | 1.03907E-05 |
| 440, 446, 452, 460, 477, 478, 486, 498, 501, 505 | 1.03907E-05 |
| 440, 446, 460, 477, 478, 486, 498, 501 | 1.03907E-05 |
| 450 | 1.03907E-05 |
| 440, 445, 446, 450, 452, 455, 460, 477, 478, 482, 483, 484, 486, 498, 501 | 1.03907E-05 |
| 440, 445, 446, 452, 460, 477, 478, 484, 486, 490, 498, 501, 505 | 1.03907E-05 |
| 455, 477, 478 | 1.03907E-05 |
| 450, 498, 501, 505 | 1.03907E-05 |
| 446, 450, 452, 455, 460, 477, 478, 481, 484, 486, 498, 501 | 1.03907E-05 |
| 440, 446, 455, 460, 477, 478, 486, 498, 501, 505 | 1.03907E-05 |
| 440, 445, 450, 498, 501, 505 | 1.03907E-05 |
| 445, 446, 450, 452, 455, 460, 498, 501, 505 | 1.03907E-05 |
| 478, 481, 501, 505 | 1.03907E-05 |
| 440, 445, 452, 456, 460, 477, 478, 484, 486, 498, 501, 505 | 1.03907E-05 |
| 445, 446, 450, 452, 455, 460, 477, 478, 481, 486 | 1.03907E-05 |
| 445, 446, 450, 452, 477, 478, 481, 498, 501, 505 | 1.03907E-05 |
| 440, 445, 446, 450, 452, 455, 477, 478, 481, 486, 498, 501, 505 | 1.03907E-05 |
| 477 | 1.03907E-05 |
| 460, 477, 478, 481, 484, 486, 498 | 1.03907E-05 |
| 445, 446, 450, 452, 460, 477, 478, 481, 501, 505 | 1.03907E-05 |
| 440, 445, 446, 450, 451, 452, 455, 460, 477, 478, 498, 501, 505 | 1.03907E-05 |
| 440, 446, 450, 460, 477, 478, 486, 498, 501, 505 | 1.03907E-05 |
| 461, 477, 478, 481, 484, 486, 498, 501, 505 | 1.03907E-05 |
| 445, 446, 450, 477, 478, 481 | 1.03907E-05 |
| 440, 445, 446, 450, 452, 455, 460, 477, 478, 484, 486, 498, 505 | 1.03907E-05 |
| 440, 445, 446, 450, 455, 460, 477, 486, 498, 501, 505 | 1.03907E-05 |
| 440, 445, 446, 450, 455, 460, 477, 478, 481, 486, 498, 501, 505 | 1.03907E-05 |
| 445, 446, 455, 460, 477, 478, 481 | 1.03907E-05 |
| 440, 450, 452, 455, 460, 477, 498, 501, 505 | 1.03907E-05 |
| 440, 445, 446, 450, 452, 455, 477, 486, 498, 501, 505 | 1.03907E-05 |
| 440, 450, 452, 455, 460, 477, 478, 481 | 1.03907E-05 |
| 440, 445, 446, 450, 452, 455, 460, 477, 486, 498, 501, 505 | 1.03907E-05 |
| 440, 445, 446, 450, 455, 477, 486, 498, 501, 505 | 1.03907E-05 |
| 450, 452, 455, 460, 477, 478, 481, 484, 486, 498 | 1.03907E-05 |
| 440, 445, 446, 450, 452, 477, 478, 498, 501 | 1.03907E-05 |
| 460, 505 | 1.03907E-05 |
| 445, 450, 452, 455, 460, 477, 478 | 1.03907E-05 |
| 455, 498 | 1.03907E-05 |
| 440, 445, 446, 450, 452, 455, 460, 477, 478, 486, 498, 501 | 1.03907E-05 |
| 440, 460, 498, 501, 505 | 1.03907E-05 |
| 440, 445, 446, 450, 455, 477, 478, 498, 501, 505 | 1.03907E-05 |
| 440, 445, 446, 450, 452, 455, 460, 477, 478, 481, 484, 498, 501 | 1.03907E-05 |
| 450, 460, 498, 501 | 1.03907E-05 |
| 455, 460, 477, 478, 481, 486, 501 | 1.03907E-05 |
| 440, 450, 455, 460, 477, 478, 481, 498, 501, 505 | 1.03907E-05 |
| 440, 452, 455, 460, 477, 478, 481, 484, 486, 498, 501 | 1.03907E-05 |
| 440, 445, 446, 452, 455, 460, 477, 478, 481, 498, 501, 505 | 1.03907E-05 |
| 440, 445, 446, 450, 455, 477, 478, 481, 484, 486, 498, 501, 505 | 1.03907E-05 |
| 445, 446, 460, 477, 478, 481, 484, 486, 498, 501 | 1.03907E-05 |
| 440, 445, 446, 450, 452, 455, 460, 477, 478, 481, 484, 486, 498, 505 | 1.03907E-05 |
| 440, 441, 445, 446, 450, 452, 455, 460, 477, 478, 481 | 1.03907E-05 |
| 450, 452, 455, 460, 477, 478, 501 | 1.03907E-05 |
| 450, 452, 460, 477, 478, 498, 501, 505 | 1.03907E-05 |
| 440, 450, 452, 455, 460, 477, 478, 481, 498, 501, 505 | 1.03907E-05 |
| 440, 445, 446, 450, 452, 455, 477, 478, 498, 501 | 1.03907E-05 |
| 440, 445, 446, 450, 452, 455, 460, 475, 477, 478, 481 | 1.03907E-05 |
| 440, 445, 446, 450, 452, 455, 456, 460, 475, 477, 478, 481, 484, 498, 501, 505 | 1.03907E-05 |
| 440, 445, 446, 450, 452, 455, 460, 478, 481, 484, 486, 498, 501, 505 | 1.03907E-05 |
| 440, 445, 446, 450, 452, 455, 460, 477, 478, 481, 498, 501 | 1.03907E-05 |
| 440, 445, 446, 450, 452, 455, 460, 477, 478, 481, 484, 486, 498, 499, 501 | 1.03907E-05 |
| 440, 445, 446, 450, 452, 455, 460, 477, 478, 482, 484, 486, 498, 501 | 1.03907E-05 |
| 460, 501 | 1.03907E-05 |
| 455, 460, 505 | 1.03907E-05 |
| 440, 455, 456, 460, 477, 478, 481, 484, 486, 498, 501 | 1.03907E-05 |
| 440, 486, 498, 501, 505 | 1.03907E-05 |
| 445, 446, 450, 452, 455, 460, 477, 478, 481, 501 | 1.03907E-05 |
| 440, 452, 455, 460, 477, 478, 481, 484, 486, 498, 501, 505 | 1.03907E-05 |
| 460, 477, 478, 481, 498, 501, 505 | 1.03907E-05 |
| 440, 445, 446, 450, 452, 455, 460, 461, 477, 478, 481, 484, 486, 498, 501, 505 | 1.03907E-05 |
| 445, 446, 450, 455, 460, 477, 478, 481, 484, 486, 498, 501, 505 | 1.03907E-05 |
| 498, 505 | 1.03907E-05 |
| 440, 445, 446, 450, 452, 455, 456, 460, 486, 498, 501, 505 | 1.03907E-05 |
| 440, 445, 446, 450, 452, 455, 477, 478, 484, 486, 498, 501, 505 | 1.03907E-05 |
| 440, 445, 446, 448, 450, 452, 455, 460, 477, 478, 481, 484, 486, 498, 501, 505 | 1.03907E-05 |
| 477, 478, 481, 484 | 1.03907E-05 |
| 440, 445, 446, 455, 477, 478, 481, 484, 486, 498, 501, 505 | 1.03907E-05 |
| 440, 445, 446, 455, 460, 477, 478, 486, 498, 501, 505 | 1.03907E-05 |
| 440, 445, 446, 450, 452, 455, 460, 477, 478, 481, 484, 486, 498, 499, 501, 505 | 1.03907E-05 |
| 440, 450, 452, 475, 477, 478, 481, 484, 486, 498, 501, 505 | 1.03907E-05 |
| 440, 450, 452, 460, 498, 501, 505 | 1.03907E-05 |
| 440, 445, 446, 450, 452, 460, 477, 478, 481, 484, 486, 498, 501 | 1.03907E-05 |
| 440, 445, 446, 450, 452, 455, 477, 478, 481, 484, 486 | 1.03907E-05 |
| 450, 452, 455 | 1.03907E-05 |
| 450, 452, 455, 460, 477, 478, 498, 501 | 1.03907E-05 |
| 450, 460, 477, 478, 498, 501, 505 | 1.03907E-05 |
| 440, 445, 446, 450, 452, 455, 460, 477, 478, 479, 484, 486, 490, 498, 501, 505 | 1.03907E-05 |
| 440, 450, 452, 477, 478, 481, 484, 486, 498, 501, 505 | 1.03907E-05 |
| 440, 450, 452, 455, 456, 477, 478, 481, 484, 486, 498, 501, 505 | 1.03907E-05 |
| 450, 455, 460 | 1.03907E-05 |
| 440, 445, 446, 450, 452, 455, 460, 477, 478, 481, 483, 484, 485, 486, 498, 501, 505 | 1.03907E-05 |
| 440, 445, 446, 450, 477, 478, 481, 484, 486 | 1.03907E-05 |
| 445, 446, 450, 455, 460, 477, 478, 481, 484, 486, 498, 501 | 1.03907E-05 |
| 450, 455 | 1.03907E-05 |
| 440, 445, 446, 450, 452, 455, 460, 477, 478, 505 | 1.03907E-05 |
| 477, 478, 479, 481, 484, 486, 498, 501, 505 | 1.03907E-05 |
| 440, 445, 446, 450, 452, 455, 460, 477, 478, 481, 484, 486, 498, 501, 505, 507 | 1.03907E-05 |
| 440, 460, 477, 478 | 1.03907E-05 |
| 440, 445, 446, 450, 460 | 1.03907E-05 |
| 440, 445, 446, 450, 452, 455, 460, 477, 478, 481, 484, 493, 498, 501, 505 | 1.03907E-05 |
| 440, 446, 450, 452, 455, 460, 477, 478, 481, 484, 501, 505 | 1.03907E-05 |
| 460, 477 | 1.03907E-05 |
| 440, 445, 446, 450, 452, 477, 478, 481, 486, 498, 501, 505 | 1.03907E-05 |
| 477, 478, 501, 505 | 1.03907E-05 |
| 445, 446, 450, 452 | 1.03907E-05 |
| 440, 445, 446, 450, 452, 455, 460, 477, 478, 498 | 1.03907E-05 |
| 446, 450, 452, 455, 460, 477, 478, 481, 484, 501, 505 | 1.03907E-05 |
| 450, 452, 455, 460, 486, 498, 501, 505 | 1.03907E-05 |
| 440, 445, 446, 450, 452, 455, 456, 460, 477, 478, 481, 484, 486, 498, 501, 503, 505 | 1.03907E-05 |
| 440, 445, 446, 450, 460, 477, 478, 481, 484, 486, 498, 501 | 1.03907E-05 |
| 445, 446, 450, 452, 455, 460, 477, 478, 481, 484, 486, 501, 505 | 1.03907E-05 |
| 440, 450, 455, 460, 477, 478, 481, 484, 486, 498, 501 | 1.03907E-05 |
| 440, 445, 446, 450, 452, 455, 456, 460, 477, 478, 481, 484, 486, 501, 505 | 1.03907E-05 |
| 445, 446, 455, 498, 501, 505 | 1.03907E-05 |
| 446, 450, 452, 455, 460, 477, 478, 481, 484, 486, 498, 501, 505 | 1.03907E-05 |
| 440, 446, 450, 452, 455, 477, 478, 484, 486, 498, 501, 505 | 1.03907E-05 |
| 440, 446, 450, 452, 455, 460, 477, 478, 481, 484, 498, 501, 505 | 1.03907E-05 |
| 440, 445, 446, 450, 452, 455, 456, 460, 461, 477, 478, 481, 484, 486, 498, 501, 505 | 1.03907E-05 |
| 445, 446, 477, 478, 505 | 1.03907E-05 |
| 440, 445, 446, 450, 452, 455, 456, 460, 471, 477, 478, 481, 484, 486, 498, 501, 505 | 1.03907E-05 |
| 440, 445, 446, 452, 455, 460, 477, 478, 481, 484, 486, 498, 501 | 1.03907E-05 |
| 440, 445, 446, 450, 452, 455, 456, 460, 477, 478, 481, 485, 498, 501, 505 | 1.03907E-05 |
| 452, 455, 460, 477, 478 | 1.03907E-05 |
| 440, 445, 446, 450, 452, 455, 481, 484, 486, 498, 501, 505 | 1.03907E-05 |
| 450, 452, 455, 498, 501, 505 | 1.03907E-05 |
| 440, 445, 446, 450, 452, 455, 456, 460, 477, 478, 481, 484, 486, 498, 505 | 1.03907E-05 |
| 440, 450, 477, 478, 498, 501, 505 | 1.03907E-05 |
| 440, 455, 477, 478, 498, 501, 505 | 1.03907E-05 |
| 503, 505 | 1.03907E-05 |
| 446, 450, 452, 455, 477, 478, 481, 486, 498, 501, 505 | 1.03907E-05 |
| 445, 446, 450, 452, 455, 460, 477, 478, 498, 501, 505 | 1.03907E-05 |
| 440, 445, 446, 450, 452, 455, 460, 463, 477, 478, 481, 484, 486, 498, 501, 505 | 1.03907E-05 |
| 440, 445, 446, 450, 452, 455, 456, 460, 477, 478, 481, 486 | 1.03907E-05 |
| 440, 445, 446, 450, 452, 455, 456, 460, 477, 478, 481, 484, 486, 498, 499, 501, 505 | 1.03907E-05 |
| 452, 455, 456, 460, 477, 478, 481, 484, 486 | 1.03907E-05 |
| 455, 456, 460, 477, 478, 481, 484, 486, 498, 501 | 1.03907E-05 |
| 450, 452, 455, 456, 460, 477, 478, 481, 484, 486 | 1.03907E-05 |
| 440, 450, 452, 455, 456, 498, 501, 505 | 1.03907E-05 |
| 440, 460, 477, 478, 481, 484, 486, 498, 501 | 1.03907E-05 |
| 440, 446, 450, 455, 460, 477, 478, 481, 484, 486, 498, 501, 505 | 1.03907E-05 |
| 440, 445, 446, 450, 452, 455, 456, 477, 478, 481, 484, 486, 498, 501, 505 | 1.03907E-05 |
| 440, 450, 452, 455, 456, 460, 477, 478, 481, 484, 486, 498 | 1.03907E-05 |
| 440, 450, 452, 460, 477, 478, 498, 501, 505 | 1.03907E-05 |
| 440, 450, 455, 456, 460, 477, 478, 481, 484, 486, 498, 501, 503, 505 | 1.03907E-05 |
| 440, 445, 446, 450, 452, 455, 456, 477, 478, 486, 498, 501, 505 | 1.03907E-05 |
| 440, 441, 445, 446, 450, 452, 460, 477, 478, 481, 484, 486, 498, 501, 505 | 1.03907E-05 |
| 455, 456, 460, 477, 481, 484, 486, 498, 501, 505 | 1.03907E-05 |
| 440, 450, 455, 456, 460, 477, 478, 481, 484, 486, 498, 501, 505 | 1.03907E-05 |
| 440, 450, 456, 460, 477, 478 | 1.03907E-05 |
| 445, 446, 450, 452, 455, 456, 460, 477, 478, 498, 501, 505 | 1.03907E-05 |
| 460, 477, 481, 484, 486, 498, 501, 505 | 1.03907E-05 |
| 440, 446, 450, 452, 455, 456, 460, 477, 478, 498, 501, 505 | 1.03907E-05 |
| 460, 477, 478, 481, 498 | 1.03907E-05 |
| 440, 445, 446, 450, 455, 456, 460, 498, 501, 505 | 1.03907E-05 |
| 455, 456, 460, 477, 478, 481, 484, 486 | 1.03907E-05 |
| 440, 455, 460, 477, 478, 486, 498, 501, 505 | 1.03907E-05 |
| 440, 481, 484, 486, 498, 501, 505 | 1.03907E-05 |
| 455, 477, 478, 481, 486, 498, 501, 505 | 1.03907E-05 |
| 440, 445, 446, 450, 452, 455, 456, 460, 473, 477, 478, 481, 484, 486, 498, 501, 505 | 1.03907E-05 |
| 440, 445, 446, 450, 455, 456, 460, 477, 478, 498, 501, 505 | 1.03907E-05 |
| 445, 446, 455, 456, 460, 477, 478, 481, 484, 486, 498, 501, 505 | 1.03907E-05 |
| 440, 450, 452 | 1.03907E-05 |
| 440, 445, 446, 455, 456, 460, 477, 478, 498, 501, 505 | 1.03907E-05 |
| 440, 441, 445, 446, 450, 452, 455, 456, 460, 477, 478, 481, 484, 486 | 1.03907E-05 |
| 440, 445, 446, 450, 498, 501, 505 | 1.03907E-05 |
| 450, 456, 460, 477, 478 | 1.03907E-05 |
| 440, 445, 446, 450, 452, 455, 456, 460, 498, 501, 505 | 1.03907E-05 |
| 456, 460, 477, 478, 481, 486, 498, 501, 505 | 1.03907E-05 |
| 446, 450, 452 | 1.03907E-05 |
| 445, 446, 498, 501, 505 | 1.03907E-05 |
| 440, 452, 455, 456, 460, 498, 501, 505 | 1.03907E-05 |
| 440, 456, 460, 477, 478, 481, 484, 486, 498, 501, 505 | 1.03907E-05 |
| 440, 445, 446, 450, 452, 455, 456, 460, 477, 478 | 1.03907E-05 |
| 440, 452, 455, 456, 460, 477, 478, 498, 501, 505 | 1.03907E-05 |
| 440, 445, 446, 450, 452, 455, 456, 460, 477, 478, 486, 498, 501, 505 | 1.03907E-05 |
| 460, 477, 478, 481, 486, 498, 501, 505 | 1.03907E-05 |
| 455, 456, 460, 477, 478, 481 | 1.03907E-05 |
| 440, 450, 455, 456, 460 | 1.03907E-05 |
| 450, 477, 478, 481, 498, 501, 505 | 1.03907E-05 |
| 460, 477, 498, 501, 505 | 1.03907E-05 |
| 460, 477, 478, 498, 501 | 1.03907E-05 |
| 440, 445, 446, 450, 452, 455, 456, 460, 477, 478, 481, 484, 486, 490, 498, 501, 505 | 1.03907E-05 |
| 440, 441, 445, 446, 450, 452, 455, 456, 460 | 1.03907E-05 |
| 450, 455, 456, 460, 477, 478, 481, 484, 486 | 1.03907E-05 |
| 440, 455, 456, 460, 477, 478, 481, 484, 486, 498, 501, 505 | 1.03907E-05 |
| 460, 477, 478, 486, 498, 501 | 1.03907E-05 |
| 460, 477, 478, 481, 484, 486, 505 | 1.03907E-05 |
| 440, 450, 452, 455, 456, 460, 477, 478, 481, 484, 486, 498, 501 | 1.03907E-05 |
| 440, 446, 477, 478, 484, 498, 501, 505 | 1.03907E-05 |
| 440, 445, 446, 450, 452, 455, 456, 460, 474, 477, 478, 481, 484, 486, 498, 501, 505 | 1.03907E-05 |
| 440, 445, 446, 450, 452, 455, 456, 460, 477, 478, 481, 484, 486, 498, 501, 504, 505 | 1.03907E-05 |
| 452, 455, 456, 460, 477, 478, 481, 484, 486, 498, 501, 505 | 1.03907E-05 |
| 440, 445, 450, 452, 455, 456, 460, 477, 478, 481, 484, 486, 493, 498, 501, 505 | 1.03907E-05 |
| 440, 446, 450, 452, 455, 460, 477, 478, 481, 484, 486, 501, 505 | 1.03907E-05 |
| 455, 456, 460, 477, 478, 481, 498, 501, 505 | 1.03907E-05 |
| 440, 445, 446, 450, 452, 455, 456, 460, 462, 477, 478, 481, 484, 486 | 1.03907E-05 |
| 440, 445, 446, 450, 452, 455, 456, 460, 470, 477, 478, 481, 484, 486, 498, 501, 505 | 1.03907E-05 |
| 440, 452, 455, 456, 460, 477, 478, 481, 484, 486, 498 | 1.03907E-05 |
| 440, 445, 446, 450, 452, 455, 456, 460, 477, 478, 498, 501 | 1.03907E-05 |
| **Training set 3** | |
| **mutation combination** | **frequency** |
| 440, 445, 446, 450, 452, 455, 460, 477, 478, 481, 484, 486, 498, 501, 505 | 0.740295096 |
| 440, 445, 446, 450, 452, 455, 456, 460, 477, 478, 481, 484, 486, 498, 501, 505 | 0.182689111 |
| N/A | 0.033686617 |
| 477, 478, 481, 484, 486, 498, 501, 505 | 0.006494181 |
| 440, 445, 446, 450, 452, 455, 460, 477, 478, 481, 484, 486, 498, 501 | 0.004280964 |
| 440, 445, 446, 450, 452, 455, 460, 477, 478, 481, 486, 498, 501, 505 | 0.001818371 |
| 440, 445, 446, 450, 452, 455, 460, 477, 478, 481, 484, 486 | 0.001724855 |
| 440, 445, 446, 450, 452, 460, 477, 478, 481, 484, 486, 498, 501, 505 | 0.001704073 |
| 498, 501, 505 | 0.001381962 |
| 440, 445, 446, 450, 452, 455, 460, 477, 478, 498, 501, 505 | 0.000997506 |
| 440, 441, 445, 446, 450, 452, 455, 460, 477, 478, 481, 484, 486, 498, 501, 505 | 0.000924771 |
| 440, 444, 445, 446, 450, 452, 455, 460, 477, 478, 481, 484, 486, 498, 501, 505 | 0.000696176 |
| 440, 445, 446, 450, 452, 455, 456, 460, 477, 481, 484, 486, 498, 501, 505 | 0.000613051 |
| 440, 441, 445, 446, 450, 452, 455, 456, 460, 477, 478, 481, 484, 486, 498, 501, 505 | 0.000550707 |
| 440, 445, 446, 450, 452, 455, 456, 460, 475, 477, 478, 481, 484, 486, 498, 501, 505 | 0.000540316 |
| 440, 445, 446, 450, 452, 455, 460, 477, 478, 486, 498, 501, 505 | 0.000509144 |
| 440, 445, 446, 450, 452, 455, 460, 477, 478, 481, 483, 484, 486, 498, 501, 505 | 0.000498753 |
| 440, 445, 446, 450, 452, 455, 460, 477, 478, 484, 486, 490, 498, 501, 505 | 0.000477972 |
| 440, 445, 446, 450, 452, 455, 456, 460, 477, 478, 481, 484, 486 | 0.000477972 |
| 460, 477, 478, 481, 484, 486, 498, 501, 505 | 0.00045719 |
| 440, 445, 446, 450, 452, 455, 460, 475, 477, 478, 481, 484, 486, 498, 501, 505 | 0.000426018 |
| 440, 445, 446, 450, 452, 455, 460, 477, 478, 484, 486, 498, 501, 505 | 0.000415628 |
| 477, 478, 486, 498, 501, 505 | 0.000415628 |
| 440, 446, 450, 452, 455, 460, 477, 478, 481, 484, 486, 498, 501, 505 | 0.000405237 |
| 505 | 0.000405237 |
| 440, 445, 446, 450, 452, 455, 456, 460, 477, 478, 481, 484, 486, 498, 501 | 0.000384456 |
| 440, 445, 446, 450, 452, 455, 460, 477, 478, 481 | 0.000363674 |
| 440, 445, 446, 450, 455, 460, 477, 478, 481, 484, 486, 498, 501, 505 | 0.000353283 |
| 440, 445, 446, 450, 452, 455, 460, 477, 478, 481, 484, 498, 501, 505 | 0.000342893 |
| 440, 445, 446, 450, 452, 455, 477, 478, 481, 484, 486, 498, 501, 505 | 0.000332502 |
| 440, 445, 446, 450, 452, 455, 460, 477, 481, 484, 486, 498, 501, 505 | 0.000322111 |
| 450, 452, 455, 460, 477, 478, 481, 484, 486, 498, 501, 505 | 0.000311721 |
| 440, 450, 452, 455, 460, 477, 478, 481, 484, 486, 498, 501, 505 | 0.00030133 |
| 440, 444, 445, 446, 450, 452, 453, 455, 460, 477, 478, 481, 484, 486, 498, 501, 505 | 0.00030133 |
| 477, 478, 498, 501, 505 | 0.000290939 |
| 440, 445, 446, 450, 452, 455, 460 | 0.000280549 |
| 440, 445, 446, 450, 452, 455, 460, 498, 501, 505 | 0.000270158 |
| 445, 446, 450, 452, 455, 460, 477, 478, 481, 484, 486, 498, 501, 505 | 0.000270158 |
| 440, 445, 446, 450, 452, 455, 460, 477, 478, 482, 483, 484, 486, 498, 501, 505 | 0.000259767 |
| 440, 445, 446, 450, 452, 455, 460, 477, 478, 479, 481, 484, 486, 498, 501, 505 | 0.000238986 |
| 440, 445, 446, 450, 452, 455, 456, 460, 477, 478, 481, 484, 486, 493, 498, 501, 505 | 0.000238986 |
| 440, 445, 446, 450, 452, 455, 456, 460, 477, 478, 481 | 0.000228595 |
| 440, 445, 446, 450, 452, 455, 460, 477, 478, 481, 498, 501, 505 | 0.000218204 |
| 460, 477, 478, 481, 484, 486 | 0.000218204 |
| 440, 477, 478, 481, 484, 486, 498, 501, 505 | 0.000207814 |
| 440, 460 | 0.000207814 |
| 477, 478, 481, 484, 486 | 0.000207814 |
| 440, 445, 446, 455, 460, 477, 478, 481, 484, 486, 498, 501, 505 | 0.000166251 |
| 440, 445, 446, 450, 452, 455, 460, 477, 478, 482, 486, 498, 501, 505 | 0.00015586 |
| 440, 445, 446, 450, 452, 455, 460, 477, 478, 481, 484, 486, 498, 500, 501, 505 | 0.00015586 |
| 477, 478 | 0.00014547 |
| 440, 445, 446, 450, 452, 455, 460, 476, 477, 478, 481, 484, 486, 498, 501, 505 | 0.00014547 |
| 498, 501 | 0.00014547 |
| 455, 460, 477, 478, 481, 484, 486, 498, 501, 505 | 0.00014547 |
| 440, 445, 446, 450, 452, 455, 456, 460, 477, 478, 498, 501, 505 | 0.000135079 |
| 440, 460, 498 | 0.000135079 |
| 440, 445, 446, 450, 452, 455, 460, 477, 478, 481, 484, 486, 498 | 0.000114298 |
| 440, 445, 446, 477, 478, 481, 484, 486, 498, 501, 505 | 0.000114298 |
| 501, 505 | 0.000114298 |
| 440, 445, 446, 450, 452, 455, 460, 477, 478, 482, 484, 486, 498, 501, 505 | 0.000114298 |
| 440, 445, 446, 450, 455, 456, 460, 477, 478, 481, 498, 501, 505 | 0.000114298 |
| 440, 444, 445, 446, 450, 452, 455, 456, 460, 477, 478, 481, 484, 486, 498, 501, 505 | 0.000114298 |
| 440, 445, 446, 450, 452, 455, 460, 477, 478 | 0.000103907 |
| 450, 452, 455, 456, 460, 477, 478, 481, 484, 486, 498, 501, 505 | 0.000103907 |
| 440, 445, 446, 450, 452, 455, 460, 477, 478, 481, 484, 486, 498, 501, 503, 505 | 9.35162E-05 |
| 460 | 9.35162E-05 |
| 440, 445, 446, 450, 460, 477, 478, 481, 484, 486, 498, 501, 505 | 9.35162E-05 |
| 501 | 9.35162E-05 |
| 440, 445, 446, 450, 452, 455, 456, 460, 477, 478, 481, 484, 498, 501, 505 | 9.35162E-05 |
| 440, 445, 446, 450, 452, 455, 456, 460, 477, 478, 481, 486, 498, 501, 505 | 9.35162E-05 |
| 440, 445, 446, 477, 478, 486, 498, 501, 505 | 8.31255E-05 |
| 440, 445, 446, 450, 452, 455, 460, 470, 477, 478, 481, 484, 486, 498, 501, 505 | 8.31255E-05 |
| 440, 460, 477, 478, 481, 484, 486, 498, 501, 505 | 8.31255E-05 |
| 440, 445, 446, 450, 452, 455, 460, 477, 478, 481, 484, 485, 486, 498, 501, 505 | 8.31255E-05 |
| 440, 445, 446, 450, 452, 455, 456, 460, 477, 478, 481, 484, 486, 498, 500, 501, 505 | 8.31255E-05 |
| 450, 477, 478, 481, 484, 486, 498, 501, 505 | 7.27348E-05 |
| 440, 455, 460, 477, 478, 481, 484, 486, 498, 501, 505 | 7.27348E-05 |
| 498 | 7.27348E-05 |
| 440, 445, 446, 450, 452, 455, 477, 478, 498, 501, 505 | 7.27348E-05 |
| 440 | 7.27348E-05 |
| 440, 445, 446, 450, 452, 455, 458, 460, 477, 478, 481, 484, 486, 498, 501, 505 | 7.27348E-05 |
| 440, 460, 498, 505 | 7.27348E-05 |
| 455, 460, 477, 478, 498, 501, 505 | 6.23441E-05 |
| 440, 445, 446, 450, 452, 455, 498, 501, 505 | 6.23441E-05 |
| 455, 460, 477, 478, 481, 484, 486, 498, 501 | 6.23441E-05 |
| 455, 460, 477, 478, 481, 484, 486 | 6.23441E-05 |
| 440, 445, 446, 450, 452, 455, 460, 462, 477, 478, 481, 484, 486, 498, 501, 505 | 6.23441E-05 |
| 460, 477, 478 | 6.23441E-05 |
| 440, 445, 446, 450, 452, 455, 460, 477, 478, 481, 484, 486, 501, 505 | 6.23441E-05 |
| 455, 498, 501, 505 | 6.23441E-05 |
| 440, 446, 450, 452, 455, 456, 460, 477, 478, 481, 484, 486, 498, 501, 505 | 6.23441E-05 |
| 440, 445, 446, 450, 452, 455, 456, 460, 477, 478, 482, 483, 484, 486, 498, 501, 505 | 6.23441E-05 |
| 440, 445, 446, 450, 455, 460, 477, 478, 486, 498, 501, 505 | 5.19534E-05 |
| 460, 477, 478, 481 | 5.19534E-05 |
| 440, 445, 477, 478, 481, 484, 486, 498, 501, 505 | 5.19534E-05 |
| 440, 445, 446, 450, 452, 455, 460, 477, 478, 483, 484, 486, 498, 501, 505 | 5.19534E-05 |
| 440, 445, 446, 450, 452, 477, 478, 481, 484, 486, 498, 501, 505 | 5.19534E-05 |
| 440, 445, 446, 452, 455, 460, 477, 478, 481, 484, 486, 498, 501, 505 | 5.19534E-05 |
| 450, 455, 460, 477, 478, 481, 484, 486, 498, 501, 505 | 5.19534E-05 |
| 440, 445, 446, 460, 477, 478, 481, 484, 486, 498, 501, 505 | 5.19534E-05 |
| 440, 450, 460, 477, 478, 481, 484, 486, 498, 501, 505 | 5.19534E-05 |
| 477, 478, 481 | 5.19534E-05 |
| 440, 460, 505 | 5.19534E-05 |
| 445, 446, 450, 452, 455, 456, 460, 477, 478, 481, 484, 486, 498, 501, 505 | 5.19534E-05 |
| 440, 445, 446, 450, 452, 455, 456, 459, 460, 477, 478, 481, 484, 486, 498, 501, 505 | 5.19534E-05 |
| 440, 445, 446, 460, 477, 478, 486, 498, 501, 505 | 4.15628E-05 |
| 445, 446, 450, 452, 455, 460, 477, 478, 481, 484, 486 | 4.15628E-05 |
| 450, 455, 498, 501, 505 | 4.15628E-05 |
| 440, 445, 446, 450, 452, 455 | 4.15628E-05 |
| 477, 478, 481, 498, 501, 505 | 4.15628E-05 |
| 440, 445, 446, 450, 452, 455, 460, 477, 478, 481, 484, 486, 487, 498, 501, 505 | 4.15628E-05 |
| 460, 477, 478, 498, 501, 505 | 4.15628E-05 |
| 477, 478, 481, 484, 486, 498, 501 | 4.15628E-05 |
| 440, 450, 455, 460, 477, 478, 481, 484, 486, 498, 501, 505 | 4.15628E-05 |
| 440, 445, 446, 450, 452, 498, 501, 505 | 4.15628E-05 |
| 440, 445, 446, 450, 455, 460, 477, 478, 484, 486, 498, 501, 505 | 4.15628E-05 |
| 440, 445, 446, 450, 452, 455, 460, 477, 478, 481, 484, 486, 494, 498, 501, 505 | 4.15628E-05 |
| 440, 450, 455, 460, 477, 478, 498, 501, 505 | 4.15628E-05 |
| 450, 452, 455, 460, 477, 478, 498, 501, 505 | 4.15628E-05 |
| 440, 445, 446, 450, 452, 455, 460, 477, 498, 501, 505 | 4.15628E-05 |
| 440, 445, 446, 450, 452, 456, 460, 477, 478, 481, 484, 486, 498, 501, 505 | 4.15628E-05 |
| 440, 445, 446, 450, 452, 455, 456, 460, 477, 478, 481, 498, 501, 505 | 4.15628E-05 |
| 440, 445, 446, 450, 452, 455, 460, 477, 478, 484, 498, 501, 505 | 4.15628E-05 |
| 440, 450, 452, 455, 456, 460, 477, 478, 481, 484, 486, 498, 501, 505 | 4.15628E-05 |
| 440, 445, 446, 450, 452, 455, 456, 460, 477, 478, 484, 486, 498, 501, 505 | 4.15628E-05 |
| 440, 445, 446, 450, 460, 477, 478, 486, 498, 501, 505 | 3.11721E-05 |
| 440, 445, 460, 477, 478, 481, 484, 486, 498, 501, 505 | 3.11721E-05 |
| 440, 445, 446, 456, 460, 477, 478, 484, 486, 490, 498, 501, 505 | 3.11721E-05 |
| 440, 445, 446, 452, 460, 477, 478, 484, 486, 498, 501, 505 | 3.11721E-05 |
| 440, 445, 446, 452, 456, 460, 477, 478, 484, 486, 490, 498, 501, 505 | 3.11721E-05 |
| 440, 460, 477, 478, 486, 498, 501, 505 | 3.11721E-05 |
| 455, 460, 498, 501, 505 | 3.11721E-05 |
| 440, 445, 446, 450, 452, 455, 459, 460, 477, 478, 481, 484, 486, 498, 501, 505 | 3.11721E-05 |
| 440, 445, 446, 450, 452, 498, 501 | 3.11721E-05 |
| 440, 445, 446, 450, 452, 455, 456, 460, 477, 478, 484, 486, 490, 498, 501, 505 | 3.11721E-05 |
| 455 | 3.11721E-05 |
| 440, 450, 452, 455, 460, 477, 478 | 3.11721E-05 |
| 450, 452, 455, 460, 477, 478, 481, 484, 486 | 3.11721E-05 |
| 440, 445, 446, 450, 477, 478, 481, 484, 486, 498, 501, 505 | 3.11721E-05 |
| 477, 498, 501, 505 | 3.11721E-05 |
| 440, 445, 446, 450, 452, 455, 460, 474, 477, 478, 481, 484, 486, 498, 501, 505 | 3.11721E-05 |
| 440, 450, 477, 478, 481, 484, 486, 498, 501, 505 | 3.11721E-05 |
| 450, 452, 455, 460, 477, 478, 481, 484, 486, 498, 501 | 3.11721E-05 |
| 440, 443, 445, 446, 450, 452, 455, 460, 477, 478, 481, 484, 486, 498, 501, 505 | 3.11721E-05 |
| 440, 450, 452, 455, 460, 477, 478, 481, 484, 486, 498, 501 | 3.11721E-05 |
| 450, 452, 455, 460, 477, 478 | 3.11721E-05 |
| 440, 445, 446, 450, 452, 455, 456, 460, 477, 478, 481, 483, 484, 486, 498, 501, 505 | 3.11721E-05 |
| 450, 455, 460, 477, 478, 498, 501, 505 | 3.11721E-05 |
| 440, 445, 446, 450, 452, 477, 478, 498, 501, 505 | 3.11721E-05 |
| 440, 445, 446, 450, 452, 477, 478, 486, 498, 501, 505 | 3.11721E-05 |
| 440, 445, 446, 450, 452, 453, 455, 460, 477, 478, 481, 484, 486, 498, 501, 505 | 3.11721E-05 |
| 440, 445, 446, 450, 452, 455, 456, 460, 476, 477, 478, 481, 484, 486, 498, 501, 505 | 3.11721E-05 |
| 440, 445, 446, 450, 452, 455, 456, 460, 477, 478, 481, 484, 485, 486, 498, 501, 505 | 3.11721E-05 |
| 440, 445, 446, 455, 456, 460, 477, 478, 484, 486, 490, 498, 501, 505 | 2.07814E-05 |
| 455, 460, 477, 478 | 2.07814E-05 |
| 440, 445, 446, 450, 452, 455, 460, 505 | 2.07814E-05 |
| 440, 455, 460, 477, 478, 498, 501, 505 | 2.07814E-05 |
| 440, 445, 446, 455, 460, 477, 478, 484, 486, 490, 498, 501, 505 | 2.07814E-05 |
| 440, 445, 446, 455, 456, 460, 477, 478, 481, 484, 486, 498, 501, 505 | 2.07814E-05 |
| 450, 452, 455, 477, 478, 481, 484, 486, 498, 501, 505 | 2.07814E-05 |
| 440, 445, 446, 450, 455, 460, 477, 478, 481, 484, 486, 498, 501 | 2.07814E-05 |
| 477, 478, 484, 486, 498, 501, 505 | 2.07814E-05 |
| 440, 445, 446, 450, 477, 478, 486, 498, 501, 505 | 2.07814E-05 |
| 481, 484, 486, 498, 501, 505 | 2.07814E-05 |
| 440, 445, 446, 460, 477, 478, 484, 486, 498, 501, 505 | 2.07814E-05 |
| 440, 445, 446, 450, 452, 460, 477, 478, 486, 498, 501, 505 | 2.07814E-05 |
| 486, 498, 501, 505 | 2.07814E-05 |
| 440, 445, 446, 450, 452, 455, 460, 477, 478, 498, 501 | 2.07814E-05 |
| 450, 452 | 2.07814E-05 |
| 440, 445, 446, 450, 452, 455, 460, 477, 478, 481, 484, 486, 502, 503 | 2.07814E-05 |
| 460, 498, 501, 505 | 2.07814E-05 |
| 460, 477, 478, 481, 484, 486, 498, 501 | 2.07814E-05 |
| 440, 445, 446, 452, 460, 477, 478, 486, 498, 501, 505 | 2.07814E-05 |
| 440, 450, 455, 460, 477, 478, 481, 484, 486, 498 | 2.07814E-05 |
| 440, 445, 446, 450, 452, 455, 460, 477, 478, 501, 505 | 2.07814E-05 |
| 450, 452, 455, 460, 477, 478, 481 | 2.07814E-05 |
| 455, 477, 478, 481, 484, 486, 498, 501, 505 | 2.07814E-05 |
| 440, 450, 452, 460, 477, 478 | 2.07814E-05 |
| 450, 460, 477, 478 | 2.07814E-05 |
| 445, 446, 450, 452, 455, 477, 478, 481, 484, 486, 498, 501, 505 | 2.07814E-05 |
| 440, 445, 446, 450, 452, 455, 460, 477, 478, 481, 484, 486, 505 | 2.07814E-05 |
| 450, 460, 477, 478, 481, 484, 486, 498, 501, 505 | 2.07814E-05 |
| 440, 445, 450, 452, 455, 460, 477, 478, 481, 484, 486, 498, 501, 505 | 2.07814E-05 |
| 450, 452, 477, 478, 481, 484, 486, 498, 501, 505 | 2.07814E-05 |
| 440, 446, 450, 452, 455, 460, 477, 478, 498, 501, 505 | 2.07814E-05 |
| 445, 446, 450, 452, 455, 460, 477, 478, 481, 498, 501, 505 | 2.07814E-05 |
| 477, 478, 498, 501 | 2.07814E-05 |
| 440, 445, 446, 450, 452, 455, 460, 477, 478, 481, 484, 486, 501 | 2.07814E-05 |
| 440, 498, 501, 505 | 2.07814E-05 |
| 440, 445, 446, 450, 452, 455, 460, 486, 498, 501, 505 | 2.07814E-05 |
| 455, 460 | 2.07814E-05 |
| 455, 460, 477, 478, 481, 498, 501, 505 | 2.07814E-05 |
| 440, 445, 446, 450, 452, 455, 460, 477, 478, 481, 485, 498, 501, 505 | 2.07814E-05 |
| 460, 498 | 2.07814E-05 |
| 440, 450, 455, 477, 478, 481, 484, 486, 498, 501, 505 | 2.07814E-05 |
| 477, 478, 481, 486, 498, 501, 505 | 2.07814E-05 |
| 477, 478, 501 | 2.07814E-05 |
| 440, 445, 446, 450, 452, 455, 460, 477, 478, 481, 484, 486, 498, 501, 505, 508 | 2.07814E-05 |
| 440, 450, 452, 455, 477, 478, 481, 484, 486, 498, 501, 505 | 2.07814E-05 |
| 440, 445, 446, 450, 452, 460, 477, 478, 481, 484, 498, 501, 505 | 2.07814E-05 |
| 440, 445, 446, 450, 452, 455, 477, 478, 486, 498, 501, 505 | 2.07814E-05 |
| 445, 446, 450, 452, 498, 501, 505 | 2.07814E-05 |
| 440, 445, 446, 450, 452, 477, 478, 484, 486, 498, 501, 505 | 2.07814E-05 |
| 450, 452, 455, 460, 498, 501, 505 | 2.07814E-05 |
| 440, 445, 446, 450, 452, 455, 460, 477, 478, 481, 484, 486, 498, 501, 504, 505 | 2.07814E-05 |
| 440, 477, 478, 498, 501, 505 | 2.07814E-05 |
| 477, 478, 481, 484, 498, 501, 505 | 2.07814E-05 |
| 440, 445, 446, 450, 452, 455, 460, 477, 478, 481, 484, 486, 493, 498, 501, 505 | 2.07814E-05 |
| 450, 452, 455, 456, 460, 477, 478, 498, 501, 505 | 2.07814E-05 |
| 440, 445, 446, 450, 452, 455, 456, 460, 477, 478, 482, 484, 486, 498, 501, 505 | 2.07814E-05 |
| 455, 456, 460 | 2.07814E-05 |
| 455, 456, 460, 477, 478, 481, 484, 486, 498, 501, 505 | 2.07814E-05 |
| 440, 445, 446, 455, 456, 460, 477, 478, 481, 498, 501, 505 | 2.07814E-05 |
| 440, 445, 446, 450, 452, 453, 455, 456, 460, 477, 478, 481, 484, 486, 498, 501, 505 | 2.07814E-05 |
| 440, 445, 446, 450, 452, 455, 456, 498, 501, 505 | 2.07814E-05 |
| 440, 445, 446, 450, 452, 455, 456, 460, 468, 477, 478, 481, 484, 486, 498, 501, 505 | 2.07814E-05 |
| 450, 452, 455, 456, 498, 501, 505 | 2.07814E-05 |
| 440, 445, 446, 450, 452, 455, 456, 460, 477, 498, 501, 505 | 2.07814E-05 |
| 440, 445, 446, 450, 455, 456, 460, 477, 478, 481, 484, 486, 498, 501, 505 | 2.07814E-05 |
| 456, 460, 477, 478, 481, 484, 486, 498, 501, 505 | 2.07814E-05 |
| 440, 445, 446, 450, 452, 455, 456, 460, 477, 478, 479, 481, 484, 486, 498, 501, 505 | 2.07814E-05 |
| 440, 445, 446, 450, 452, 460, 477, 478, 498, 501, 505 | 1.03907E-05 |
| 440, 445, 446, 456, 460, 484, 486, 490, 498, 501, 505 | 1.03907E-05 |
| 440, 452, 460, 477, 478, 481, 484, 486 | 1.03907E-05 |
| 440, 445, 446, 450, 452, 455, 460, 477, 478, 481, 484, 501, 505 | 1.03907E-05 |
| 440, 445, 446, 477, 486, 498, 501, 505 | 1.03907E-05 |
| 440, 445, 446, 450, 452, 455, 460, 498, 501 | 1.03907E-05 |
| 440, 445, 477 | 1.03907E-05 |
| 440, 445, 446, 460, 477, 478, 501, 505 | 1.03907E-05 |
| 440, 450, 460, 477, 478, 486, 498, 501, 505 | 1.03907E-05 |
| 445, 446, 450, 452, 455, 460, 477, 478, 481, 484, 486, 498 | 1.03907E-05 |
| 440, 445, 446, 477, 478, 498, 501, 505 | 1.03907E-05 |
| 440, 450, 452, 455, 460, 477, 478, 481, 484, 486 | 1.03907E-05 |
| 440, 445, 446, 477, 498, 501, 505 | 1.03907E-05 |
| 455, 456, 475, 477, 478, 484, 486, 490 | 1.03907E-05 |
| 440, 441, 445, 446, 450, 455, 460, 477, 478, 481, 484, 486, 498, 501, 505 | 1.03907E-05 |
| 440, 445, 446, 450, 452, 455, 460, 475, 477, 478, 498, 501, 505 | 1.03907E-05 |
| 440, 445, 446, 450, 452, 455, 477, 498, 501, 505 | 1.03907E-05 |
| 450, 455, 477, 478, 481, 484, 486, 498, 501, 505 | 1.03907E-05 |
| 440, 445, 446, 452, 477, 478, 481, 484, 486, 498, 501, 505 | 1.03907E-05 |
| 440, 450, 455, 498, 501, 505 | 1.03907E-05 |
| 440, 445, 446, 452, 455, 460, 477, 478, 484, 486, 498, 501, 505 | 1.03907E-05 |
| 440, 445, 446, 450, 477, 486, 498, 501, 505 | 1.03907E-05 |
| 452, 455, 460, 477, 478, 481, 484, 486 | 1.03907E-05 |
| 440, 445, 446, 452, 455, 477, 478, 481, 484, 486, 498, 501, 505 | 1.03907E-05 |
| 440, 455, 460, 477, 478, 481, 486, 498, 501, 505 | 1.03907E-05 |
| 450, 452, 455, 498, 501 | 1.03907E-05 |
| 440, 445, 446, 450, 452, 455, 475, 477, 478, 481, 484, 486 | 1.03907E-05 |
| 440, 452, 455, 460, 477, 478, 481, 484, 486 | 1.03907E-05 |
| 440, 445, 446, 450, 455, 460, 477, 478, 481, 484, 486 | 1.03907E-05 |
| 440, 445, 446, 450, 455, 477, 478, 486, 498, 501, 505 | 1.03907E-05 |
| 440, 445, 446, 460, 477, 478, 484, 486, 490, 498, 501, 505 | 1.03907E-05 |
| 450, 455, 460, 477, 478 | 1.03907E-05 |
| 440, 445, 446, 450, 455, 460, 477, 478, 481, 484, 486, 501, 505 | 1.03907E-05 |
| 440, 446, 455, 460, 477, 478, 481, 484, 486, 498, 501 | 1.03907E-05 |
| 476, 477, 478, 481, 484, 486, 498, 501, 505 | 1.03907E-05 |
| 440, 445, 446, 450, 452, 455, 476, 477, 478, 481, 484, 486, 498, 501, 505 | 1.03907E-05 |
| 440, 445, 446, 456, 460 | 1.03907E-05 |
| 440, 450, 477, 478, 481, 484, 486 | 1.03907E-05 |
| 450, 455, 477, 498, 501, 505 | 1.03907E-05 |
| 440, 445, 446, 450, 452, 455, 460, 478, 484, 486, 498, 501, 505 | 1.03907E-05 |
| 440, 445, 446, 450, 452, 455, 477, 478, 481, 498, 501, 505 | 1.03907E-05 |
| 440, 445, 446, 450, 455, 460, 498, 501, 505 | 1.03907E-05 |
| 460, 477, 478, 484, 498 | 1.03907E-05 |
| 477, 505 | 1.03907E-05 |
| 440, 445, 446, 450, 452, 455, 460, 477, 478, 481, 484, 486, 498, 501, 507 | 1.03907E-05 |
| 440, 446, 450, 452, 455, 460, 477, 478, 481 | 1.03907E-05 |
| 440, 446, 477, 478, 486, 498, 501, 505 | 1.03907E-05 |
| 440, 445, 446, 450, 452, 455, 460, 477, 478, 479, 498, 501, 505 | 1.03907E-05 |
| 455, 460, 477, 478, 481, 498 | 1.03907E-05 |
| 440, 445, 446, 450, 452, 455, 460, 507 | 1.03907E-05 |
| 440, 445, 446, 450, 452, 460, 477, 478, 481 | 1.03907E-05 |
| 484 | 1.03907E-05 |
| 477, 478, 481, 484, 486, 498, 505 | 1.03907E-05 |
| 440, 445, 446, 456, 460, 477, 478, 484, 486, 498, 501, 505 | 1.03907E-05 |
| 450, 452, 455, 460 | 1.03907E-05 |
| 440, 445, 446, 450, 452 | 1.03907E-05 |
| 507 | 1.03907E-05 |
| 446, 450, 452, 455, 460, 477, 478, 498, 501, 505 | 1.03907E-05 |
| 440, 446, 450, 452, 455, 460, 477, 478, 484, 486, 490, 498, 501, 505 | 1.03907E-05 |
| 440, 445, 446, 452, 477, 486, 498, 501, 505 | 1.03907E-05 |
| 440, 445, 446, 450, 455, 460, 477, 478, 481 | 1.03907E-05 |
| 440, 445, 446, 452, 455, 477, 478, 481, 486, 498, 501, 505 | 1.03907E-05 |
| 440, 450, 452, 455, 477, 478, 498, 501, 505 | 1.03907E-05 |
| 440, 445, 446, 452, 455, 460, 477, 478, 486, 498, 501, 505 | 1.03907E-05 |
| 477, 478, 498 | 1.03907E-05 |
| 477, 478, 481, 501 | 1.03907E-05 |
| 450, 452, 455, 460, 498, 501 | 1.03907E-05 |
| 440, 445, 446, 450, 460, 477, 478, 481, 486, 498, 501, 505 | 1.03907E-05 |
| 445, 446, 450, 452, 455, 477, 478, 498, 501, 505 | 1.03907E-05 |
| 440, 446, 460, 477, 478, 484, 486, 498, 501, 505 | 1.03907E-05 |
| 440, 445, 450, 452, 455, 498, 501, 505 | 1.03907E-05 |
| 440, 450, 452, 455, 460, 477, 478, 498, 501, 505 | 1.03907E-05 |
| 445, 446, 450, 452, 460, 477, 478, 481, 484, 486, 498, 501, 505 | 1.03907E-05 |
| 445, 446, 452, 455, 460, 477, 478, 481, 484, 486, 498, 501, 505 | 1.03907E-05 |
| 477, 478, 481, 484, 486, 501 | 1.03907E-05 |
| 440, 445, 446, 450, 460, 477, 478, 484, 486, 498, 501, 505 | 1.03907E-05 |
| 440, 446, 452, 460, 477, 478, 486, 498, 501, 505 | 1.03907E-05 |
| 440, 446, 460, 477, 478, 486, 498, 501 | 1.03907E-05 |
| 450 | 1.03907E-05 |
| 440, 445, 446, 450, 452, 455, 460, 477, 478, 482, 483, 484, 486, 498, 501 | 1.03907E-05 |
| 440, 445, 446, 452, 460, 477, 478, 484, 486, 490, 498, 501, 505 | 1.03907E-05 |
| 455, 477, 478 | 1.03907E-05 |
| 450, 498, 501, 505 | 1.03907E-05 |
| 446, 450, 452, 455, 460, 477, 478, 481, 484, 486, 498, 501 | 1.03907E-05 |
| 440, 446, 455, 460, 477, 478, 486, 498, 501, 505 | 1.03907E-05 |
| 440, 445, 450, 498, 501, 505 | 1.03907E-05 |
| 445, 446, 450, 452, 455, 460, 498, 501, 505 | 1.03907E-05 |
| 478, 481, 501, 505 | 1.03907E-05 |
| 440, 445, 452, 456, 460, 477, 478, 484, 486, 498, 501, 505 | 1.03907E-05 |
| 445, 446, 450, 452, 455, 460, 477, 478, 481, 486 | 1.03907E-05 |
| 445, 446, 450, 452, 477, 478, 481, 498, 501, 505 | 1.03907E-05 |
| 440, 445, 446, 450, 452, 455, 477, 478, 481, 486, 498, 501, 505 | 1.03907E-05 |
| 477 | 1.03907E-05 |
| 460, 477, 478, 481, 484, 486, 498 | 1.03907E-05 |
| 445, 446, 450, 452, 460, 477, 478, 481, 501, 505 | 1.03907E-05 |
| 440, 445, 446, 450, 451, 452, 455, 460, 477, 478, 498, 501, 505 | 1.03907E-05 |
| 440, 446, 450, 460, 477, 478, 486, 498, 501, 505 | 1.03907E-05 |
| 461, 477, 478, 481, 484, 486, 498, 501, 505 | 1.03907E-05 |
| 445, 446, 450, 477, 478, 481 | 1.03907E-05 |
| 440, 445, 446, 450, 452, 455, 460, 477, 478, 484, 486, 498, 505 | 1.03907E-05 |
| 440, 445, 446, 450, 455, 460, 477, 486, 498, 501, 505 | 1.03907E-05 |
| 440, 445, 446, 450, 455, 460, 477, 478, 481, 486, 498, 501, 505 | 1.03907E-05 |
| 445, 446, 455, 460, 477, 478, 481 | 1.03907E-05 |
| 440, 450, 452, 455, 460, 477, 498, 501, 505 | 1.03907E-05 |
| 440, 445, 446, 450, 452, 455, 477, 486, 498, 501, 505 | 1.03907E-05 |
| 440, 450, 452, 455, 460, 477, 478, 481 | 1.03907E-05 |
| 440, 445, 446, 450, 452, 455, 460, 477, 486, 498, 501, 505 | 1.03907E-05 |
| 440, 445, 446, 450, 455, 477, 486, 498, 501, 505 | 1.03907E-05 |
| 450, 452, 455, 460, 477, 478, 481, 484, 486, 498 | 1.03907E-05 |
| 440, 445, 446, 450, 452, 477, 478, 498, 501 | 1.03907E-05 |
| 460, 505 | 1.03907E-05 |
| 445, 450, 452, 455, 460, 477, 478 | 1.03907E-05 |
| 455, 498 | 1.03907E-05 |
| 440, 445, 446, 450, 452, 455, 460, 477, 478, 486, 498, 501 | 1.03907E-05 |
| 440, 460, 498, 501, 505 | 1.03907E-05 |
| 440, 445, 446, 450, 455, 477, 478, 498, 501, 505 | 1.03907E-05 |
| 440, 445, 446, 450, 452, 455, 460, 477, 478, 481, 484, 498, 501 | 1.03907E-05 |
| 450, 460, 498, 501 | 1.03907E-05 |
| 455, 460, 477, 478, 481, 486, 501 | 1.03907E-05 |
| 440, 450, 455, 460, 477, 478, 481, 498, 501, 505 | 1.03907E-05 |
| 440, 452, 455, 460, 477, 478, 481, 484, 486, 498, 501 | 1.03907E-05 |
| 440, 445, 446, 452, 455, 460, 477, 478, 481, 498, 501, 505 | 1.03907E-05 |
| 440, 445, 446, 450, 455, 477, 478, 481, 484, 486, 498, 501, 505 | 1.03907E-05 |
| 445, 446, 460, 477, 478, 481, 484, 486, 498, 501 | 1.03907E-05 |
| 440, 445, 446, 450, 452, 455, 460, 477, 478, 481, 484, 486, 498, 505 | 1.03907E-05 |
| 440, 441, 445, 446, 450, 452, 455, 460, 477, 478, 481 | 1.03907E-05 |
| 450, 452, 455, 460, 477, 478, 501 | 1.03907E-05 |
| 450, 452, 460, 477, 478, 498, 501, 505 | 1.03907E-05 |
| 440, 450, 452, 455, 460, 477, 478, 481, 498, 501, 505 | 1.03907E-05 |
| 440, 445, 446, 450, 452, 455, 477, 478, 498, 501 | 1.03907E-05 |
| 440, 445, 446, 450, 452, 455, 460, 475, 477, 478, 481 | 1.03907E-05 |
| 440, 445, 446, 450, 452, 455, 456, 460, 475, 477, 478, 481, 484, 498, 501, 505 | 1.03907E-05 |
| 440, 445, 446, 450, 452, 455, 460, 478, 481, 484, 486, 498, 501, 505 | 1.03907E-05 |
| 440, 445, 446, 450, 452, 455, 460, 477, 478, 481, 498, 501 | 1.03907E-05 |
| 440, 445, 446, 450, 452, 455, 460, 477, 478, 481, 484, 486, 498, 499, 501 | 1.03907E-05 |
| 440, 445, 446, 450, 452, 455, 460, 477, 478, 482, 484, 486, 498, 501 | 1.03907E-05 |
| 460, 501 | 1.03907E-05 |
| 455, 460, 505 | 1.03907E-05 |
| 440, 455, 456, 460, 477, 478, 481, 484, 486, 498, 501 | 1.03907E-05 |
| 440, 486, 498, 501, 505 | 1.03907E-05 |
| 445, 446, 450, 452, 455, 460, 477, 478, 481, 501 | 1.03907E-05 |
| 440, 452, 455, 460, 477, 478, 481, 484, 486, 498, 501, 505 | 1.03907E-05 |
| 460, 477, 478, 481, 498, 501, 505 | 1.03907E-05 |
| 440, 445, 446, 450, 452, 455, 460, 461, 477, 478, 481, 484, 486, 498, 501, 505 | 1.03907E-05 |
| 445, 446, 450, 455, 460, 477, 478, 481, 484, 486, 498, 501, 505 | 1.03907E-05 |
| 498, 505 | 1.03907E-05 |
| 440, 445, 446, 450, 452, 455, 456, 460, 486, 498, 501, 505 | 1.03907E-05 |
| 440, 445, 446, 450, 452, 455, 477, 478, 484, 486, 498, 501, 505 | 1.03907E-05 |
| 440, 445, 446, 448, 450, 452, 455, 460, 477, 478, 481, 484, 486, 498, 501, 505 | 1.03907E-05 |
| 477, 478, 481, 484 | 1.03907E-05 |
| 440, 445, 446, 455, 477, 478, 481, 484, 486, 498, 501, 505 | 1.03907E-05 |
| 440, 445, 446, 455, 460, 477, 478, 486, 498, 501, 505 | 1.03907E-05 |
| 440, 445, 446, 450, 452, 455, 460, 477, 478, 481, 484, 486, 498, 499, 501, 505 | 1.03907E-05 |
| 440, 450, 452, 475, 477, 478, 481, 484, 486, 498, 501, 505 | 1.03907E-05 |
| 440, 450, 452, 460, 498, 501, 505 | 1.03907E-05 |
| 440, 445, 446, 450, 452, 460, 477, 478, 481, 484, 486, 498, 501 | 1.03907E-05 |
| 440, 445, 446, 450, 452, 455, 477, 478, 481, 484, 486 | 1.03907E-05 |
| 450, 452, 455 | 1.03907E-05 |
| 450, 452, 455, 460, 477, 478, 498, 501 | 1.03907E-05 |
| 450, 460, 477, 478, 498, 501, 505 | 1.03907E-05 |
| 440, 445, 446, 450, 452, 455, 460, 477, 478, 479, 484, 486, 490, 498, 501, 505 | 1.03907E-05 |
| 440, 450, 452, 477, 478, 481, 484, 486, 498, 501, 505 | 1.03907E-05 |
| 440, 450, 452, 455, 456, 477, 478, 481, 484, 486, 498, 501, 505 | 1.03907E-05 |
| 450, 455, 460 | 1.03907E-05 |
| 440, 445, 446, 450, 452, 455, 460, 477, 478, 481, 483, 484, 485, 486, 498, 501, 505 | 1.03907E-05 |
| 440, 445, 446, 450, 477, 478, 481, 484, 486 | 1.03907E-05 |
| 445, 446, 450, 455, 460, 477, 478, 481, 484, 486, 498, 501 | 1.03907E-05 |
| 450, 455 | 1.03907E-05 |
| 440, 445, 446, 450, 452, 455, 460, 477, 478, 505 | 1.03907E-05 |
| 477, 478, 479, 481, 484, 486, 498, 501, 505 | 1.03907E-05 |
| 440, 445, 446, 450, 452, 455, 460, 477, 478, 481, 484, 486, 498, 501, 505, 507 | 1.03907E-05 |
| 440, 460, 477, 478 | 1.03907E-05 |
| 440, 445, 446, 450, 460 | 1.03907E-05 |
| 440, 445, 446, 450, 452, 455, 460, 477, 478, 481, 484, 493, 498, 501, 505 | 1.03907E-05 |
| 440, 446, 450, 452, 455, 460, 477, 478, 481, 484, 501, 505 | 1.03907E-05 |
| 460, 477 | 1.03907E-05 |
| 440, 445, 446, 450, 452, 477, 478, 481, 486, 498, 501, 505 | 1.03907E-05 |
| 477, 478, 501, 505 | 1.03907E-05 |
| 445, 446, 450, 452 | 1.03907E-05 |
| 440, 445, 446, 450, 452, 455, 460, 477, 478, 498 | 1.03907E-05 |
| 446, 450, 452, 455, 460, 477, 478, 481, 484, 501, 505 | 1.03907E-05 |
| 450, 452, 455, 460, 486, 498, 501, 505 | 1.03907E-05 |
| 440, 445, 446, 450, 452, 455, 456, 460, 477, 478, 481, 484, 486, 498, 501, 503, 505 | 1.03907E-05 |
| 440, 445, 446, 450, 460, 477, 478, 481, 484, 486, 498, 501 | 1.03907E-05 |
| 445, 446, 450, 452, 455, 460, 477, 478, 481, 484, 486, 501, 505 | 1.03907E-05 |
| 440, 450, 455, 460, 477, 478, 481, 484, 486, 498, 501 | 1.03907E-05 |
| 440, 445, 446, 450, 452, 455, 456, 460, 477, 478, 481, 484, 486, 501, 505 | 1.03907E-05 |
| 445, 446, 455, 498, 501, 505 | 1.03907E-05 |
| 446, 450, 452, 455, 460, 477, 478, 481, 484, 486, 498, 501, 505 | 1.03907E-05 |
| 440, 446, 450, 452, 455, 477, 478, 484, 486, 498, 501, 505 | 1.03907E-05 |
| 440, 446, 450, 452, 455, 460, 477, 478, 481, 484, 498, 501, 505 | 1.03907E-05 |
| 440, 445, 446, 450, 452, 455, 456, 460, 461, 477, 478, 481, 484, 486, 498, 501, 505 | 1.03907E-05 |
| 445, 446, 477, 478, 505 | 1.03907E-05 |
| 440, 445, 446, 450, 452, 455, 456, 460, 471, 477, 478, 481, 484, 486, 498, 501, 505 | 1.03907E-05 |
| 440, 445, 446, 452, 455, 460, 477, 478, 481, 484, 486, 498, 501 | 1.03907E-05 |
| 440, 445, 446, 450, 452, 455, 456, 460, 477, 478, 481, 485, 498, 501, 505 | 1.03907E-05 |
| 452, 455, 460, 477, 478 | 1.03907E-05 |
| 440, 445, 446, 450, 452, 455, 481, 484, 486, 498, 501, 505 | 1.03907E-05 |
| 450, 452, 455, 498, 501, 505 | 1.03907E-05 |
| 440, 445, 446, 450, 452, 455, 456, 460, 477, 478, 481, 484, 486, 498, 505 | 1.03907E-05 |
| 440, 450, 477, 478, 498, 501, 505 | 1.03907E-05 |
| 440, 455, 477, 478, 498, 501, 505 | 1.03907E-05 |
| 503, 505 | 1.03907E-05 |
| 446, 450, 452, 455, 477, 478, 481, 486, 498, 501, 505 | 1.03907E-05 |
| 445, 446, 450, 452, 455, 460, 477, 478, 498, 501, 505 | 1.03907E-05 |
| 440, 445, 446, 450, 452, 455, 460, 463, 477, 478, 481, 484, 486, 498, 501, 505 | 1.03907E-05 |
| 440, 445, 446, 450, 452, 455, 456, 460, 477, 478, 481, 486 | 1.03907E-05 |
| 440, 445, 446, 450, 452, 455, 456, 460, 477, 478, 481, 484, 486, 498, 499, 501, 505 | 1.03907E-05 |
| 452, 455, 456, 460, 477, 478, 481, 484, 486 | 1.03907E-05 |
| 455, 456, 460, 477, 478, 481, 484, 486, 498, 501 | 1.03907E-05 |
| 450, 452, 455, 456, 460, 477, 478, 481, 484, 486 | 1.03907E-05 |
| 440, 450, 452, 455, 456, 498, 501, 505 | 1.03907E-05 |
| 440, 460, 477, 478, 481, 484, 486, 498, 501 | 1.03907E-05 |
| 440, 446, 450, 455, 460, 477, 478, 481, 484, 486, 498, 501, 505 | 1.03907E-05 |
| 440, 445, 446, 450, 452, 455, 456, 477, 478, 481, 484, 486, 498, 501, 505 | 1.03907E-05 |
| 440, 450, 452, 455, 456, 460, 477, 478, 481, 484, 486, 498 | 1.03907E-05 |
| 440, 450, 452, 460, 477, 478, 498, 501, 505 | 1.03907E-05 |
| 440, 450, 455, 456, 460, 477, 478, 481, 484, 486, 498, 501, 503, 505 | 1.03907E-05 |
| 440, 445, 446, 450, 452, 455, 456, 477, 478, 486, 498, 501, 505 | 1.03907E-05 |
| 440, 441, 445, 446, 450, 452, 460, 477, 478, 481, 484, 486, 498, 501, 505 | 1.03907E-05 |
| 455, 456, 460, 477, 481, 484, 486, 498, 501, 505 | 1.03907E-05 |
| 440, 450, 455, 456, 460, 477, 478, 481, 484, 486, 498, 501, 505 | 1.03907E-05 |
| 440, 450, 456, 460, 477, 478 | 1.03907E-05 |
| 445, 446, 450, 452, 455, 456, 460, 477, 478, 498, 501, 505 | 1.03907E-05 |
| 460, 477, 481, 484, 486, 498, 501, 505 | 1.03907E-05 |
| 440, 446, 450, 452, 455, 456, 460, 477, 478, 498, 501, 505 | 1.03907E-05 |
| 460, 477, 478, 481, 498 | 1.03907E-05 |
| 440, 445, 446, 450, 455, 456, 460, 498, 501, 505 | 1.03907E-05 |
| 455, 456, 460, 477, 478, 481, 484, 486 | 1.03907E-05 |
| 440, 455, 460, 477, 478, 486, 498, 501, 505 | 1.03907E-05 |
| 440, 481, 484, 486, 498, 501, 505 | 1.03907E-05 |
| 455, 477, 478, 481, 486, 498, 501, 505 | 1.03907E-05 |
| 440, 445, 446, 450, 452, 455, 456, 460, 473, 477, 478, 481, 484, 486, 498, 501, 505 | 1.03907E-05 |
| 440, 445, 446, 450, 455, 456, 460, 477, 478, 498, 501, 505 | 1.03907E-05 |
| 445, 446, 455, 456, 460, 477, 478, 481, 484, 486, 498, 501, 505 | 1.03907E-05 |
| 440, 450, 452 | 1.03907E-05 |
| 440, 445, 446, 455, 456, 460, 477, 478, 498, 501, 505 | 1.03907E-05 |
| 440, 441, 445, 446, 450, 452, 455, 456, 460, 477, 478, 481, 484, 486 | 1.03907E-05 |
| 440, 445, 446, 450, 498, 501, 505 | 1.03907E-05 |
| 450, 456, 460, 477, 478 | 1.03907E-05 |
| 440, 445, 446, 450, 452, 455, 456, 460, 498, 501, 505 | 1.03907E-05 |
| 456, 460, 477, 478, 481, 486, 498, 501, 505 | 1.03907E-05 |
| 446, 450, 452 | 1.03907E-05 |
| 445, 446, 498, 501, 505 | 1.03907E-05 |
| 440, 452, 455, 456, 460, 498, 501, 505 | 1.03907E-05 |
| 440, 456, 460, 477, 478, 481, 484, 486, 498, 501, 505 | 1.03907E-05 |
| 440, 445, 446, 450, 452, 455, 456, 460, 477, 478 | 1.03907E-05 |
| 440, 452, 455, 456, 460, 477, 478, 498, 501, 505 | 1.03907E-05 |
| 440, 445, 446, 450, 452, 455, 456, 460, 477, 478, 486, 498, 501, 505 | 1.03907E-05 |
| 460, 477, 478, 481, 486, 498, 501, 505 | 1.03907E-05 |
| 455, 456, 460, 477, 478, 481 | 1.03907E-05 |
| 440, 450, 455, 456, 460 | 1.03907E-05 |
| 450, 477, 478, 481, 498, 501, 505 | 1.03907E-05 |
| 460, 477, 498, 501, 505 | 1.03907E-05 |
| 460, 477, 478, 498, 501 | 1.03907E-05 |
| 440, 445, 446, 450, 452, 455, 456, 460, 477, 478, 481, 484, 486, 490, 498, 501, 505 | 1.03907E-05 |
| 440, 441, 445, 446, 450, 452, 455, 456, 460 | 1.03907E-05 |
| 450, 455, 456, 460, 477, 478, 481, 484, 486 | 1.03907E-05 |
| 440, 455, 456, 460, 477, 478, 481, 484, 486, 498, 501, 505 | 1.03907E-05 |
| 460, 477, 478, 486, 498, 501 | 1.03907E-05 |
| 460, 477, 478, 481, 484, 486, 505 | 1.03907E-05 |
| 440, 450, 452, 455, 456, 460, 477, 478, 481, 484, 486, 498, 501 | 1.03907E-05 |
| 440, 446, 477, 478, 484, 498, 501, 505 | 1.03907E-05 |
| 440, 445, 446, 450, 452, 455, 456, 460, 474, 477, 478, 481, 484, 486, 498, 501, 505 | 1.03907E-05 |
| 440, 445, 446, 450, 452, 455, 456, 460, 477, 478, 481, 484, 486, 498, 501, 504, 505 | 1.03907E-05 |
| 452, 455, 456, 460, 477, 478, 481, 484, 486, 498, 501, 505 | 1.03907E-05 |
| 440, 445, 450, 452, 455, 456, 460, 477, 478, 481, 484, 486, 493, 498, 501, 505 | 1.03907E-05 |
| 440, 446, 450, 452, 455, 460, 477, 478, 481, 484, 486, 501, 505 | 1.03907E-05 |
| 455, 456, 460, 477, 478, 481, 498, 501, 505 | 1.03907E-05 |
| 440, 445, 446, 450, 452, 455, 456, 460, 462, 477, 478, 481, 484, 486 | 1.03907E-05 |
| 440, 445, 446, 450, 452, 455, 456, 460, 470, 477, 478, 481, 484, 486, 498, 501, 505 | 1.03907E-05 |
| 440, 452, 455, 456, 460, 477, 478, 481, 484, 486, 498 | 1.03907E-05 |
| 440, 445, 446, 450, 452, 455, 456, 460, 477, 478, 498, 501 | 1.03907E-05 |
| **Training set 4** | |
| **mutation combination** | **frequency** |
| 440, 445, 446, 450, 452, 455, 460, 477, 478, 481, 484, 486, 498, 501, 505 | 0.740295096 |
| 440, 445, 446, 450, 452, 455, 456, 460, 477, 478, 481, 484, 486, 498, 501, 505 | 0.182689111 |
| N/A | 0.033686617 |
| 477, 478, 481, 484, 486, 498, 501, 505 | 0.006494181 |
| 440, 445, 446, 450, 452, 455, 460, 477, 478, 481, 484, 486, 498, 501 | 0.004280964 |
| 440, 445, 446, 450, 452, 455, 460, 477, 478, 481, 486, 498, 501, 505 | 0.001818371 |
| 440, 445, 446, 450, 452, 455, 460, 477, 478, 481, 484, 486 | 0.001724855 |
| 440, 445, 446, 450, 452, 460, 477, 478, 481, 484, 486, 498, 501, 505 | 0.001704073 |
| 498, 501, 505 | 0.001381962 |
| 440, 445, 446, 450, 452, 455, 460, 477, 478, 498, 501, 505 | 0.000997506 |
| 440, 441, 445, 446, 450, 452, 455, 460, 477, 478, 481, 484, 486, 498, 501, 505 | 0.000924771 |
| 440, 444, 445, 446, 450, 452, 455, 460, 477, 478, 481, 484, 486, 498, 501, 505 | 0.000696176 |
| 440, 445, 446, 450, 452, 455, 456, 460, 477, 481, 484, 486, 498, 501, 505 | 0.000613051 |
| 440, 441, 445, 446, 450, 452, 455, 456, 460, 477, 478, 481, 484, 486, 498, 501, 505 | 0.000550707 |
| 440, 445, 446, 450, 452, 455, 456, 460, 475, 477, 478, 481, 484, 486, 498, 501, 505 | 0.000540316 |
| 440, 445, 446, 450, 452, 455, 460, 477, 478, 486, 498, 501, 505 | 0.000509144 |
| 440, 445, 446, 450, 452, 455, 460, 477, 478, 481, 483, 484, 486, 498, 501, 505 | 0.000498753 |
| 440, 445, 446, 450, 452, 455, 460, 477, 478, 484, 486, 490, 498, 501, 505 | 0.000477972 |
| 440, 445, 446, 450, 452, 455, 456, 460, 477, 478, 481, 484, 486 | 0.000477972 |
| 460, 477, 478, 481, 484, 486, 498, 501, 505 | 0.00045719 |
| 440, 445, 446, 450, 452, 455, 460, 475, 477, 478, 481, 484, 486, 498, 501, 505 | 0.000426018 |
| 440, 445, 446, 450, 452, 455, 460, 477, 478, 484, 486, 498, 501, 505 | 0.000415628 |
| 477, 478, 486, 498, 501, 505 | 0.000415628 |
| 440, 446, 450, 452, 455, 460, 477, 478, 481, 484, 486, 498, 501, 505 | 0.000405237 |
| 505 | 0.000405237 |
| 440, 445, 446, 450, 452, 455, 456, 460, 477, 478, 481, 484, 486, 498, 501 | 0.000384456 |
| 440, 445, 446, 450, 452, 455, 460, 477, 478, 481 | 0.000363674 |
| 440, 445, 446, 450, 455, 460, 477, 478, 481, 484, 486, 498, 501, 505 | 0.000353283 |
| 440, 445, 446, 450, 452, 455, 460, 477, 478, 481, 484, 498, 501, 505 | 0.000342893 |
| 440, 445, 446, 450, 452, 455, 477, 478, 481, 484, 486, 498, 501, 505 | 0.000332502 |
| 440, 445, 446, 450, 452, 455, 460, 477, 481, 484, 486, 498, 501, 505 | 0.000322111 |
| 450, 452, 455, 460, 477, 478, 481, 484, 486, 498, 501, 505 | 0.000311721 |
| 440, 450, 452, 455, 460, 477, 478, 481, 484, 486, 498, 501, 505 | 0.00030133 |
| 440, 444, 445, 446, 450, 452, 453, 455, 460, 477, 478, 481, 484, 486, 498, 501, 505 | 0.00030133 |
| 477, 478, 498, 501, 505 | 0.000290939 |
| 440, 445, 446, 450, 452, 455, 460 | 0.000280549 |
| 440, 445, 446, 450, 452, 455, 460, 498, 501, 505 | 0.000270158 |
| 445, 446, 450, 452, 455, 460, 477, 478, 481, 484, 486, 498, 501, 505 | 0.000270158 |
| 440, 445, 446, 450, 452, 455, 460, 477, 478, 482, 483, 484, 486, 498, 501, 505 | 0.000259767 |
| 440, 445, 446, 450, 452, 455, 460, 477, 478, 479, 481, 484, 486, 498, 501, 505 | 0.000238986 |
| 440, 445, 446, 450, 452, 455, 456, 460, 477, 478, 481, 484, 486, 493, 498, 501, 505 | 0.000238986 |
| 440, 445, 446, 450, 452, 455, 456, 460, 477, 478, 481 | 0.000228595 |
| 440, 445, 446, 450, 452, 455, 460, 477, 478, 481, 498, 501, 505 | 0.000218204 |
| 460, 477, 478, 481, 484, 486 | 0.000218204 |
| 440, 477, 478, 481, 484, 486, 498, 501, 505 | 0.000207814 |
| 440, 460 | 0.000207814 |
| 477, 478, 481, 484, 486 | 0.000207814 |
| 440, 445, 446, 455, 460, 477, 478, 481, 484, 486, 498, 501, 505 | 0.000166251 |
| 440, 445, 446, 450, 452, 455, 460, 477, 478, 482, 486, 498, 501, 505 | 0.00015586 |
| 440, 445, 446, 450, 452, 455, 460, 477, 478, 481, 484, 486, 498, 500, 501, 505 | 0.00015586 |
| 477, 478 | 0.00014547 |
| 440, 445, 446, 450, 452, 455, 460, 476, 477, 478, 481, 484, 486, 498, 501, 505 | 0.00014547 |
| 498, 501 | 0.00014547 |
| 455, 460, 477, 478, 481, 484, 486, 498, 501, 505 | 0.00014547 |
| 440, 445, 446, 450, 452, 455, 456, 460, 477, 478, 498, 501, 505 | 0.000135079 |
| 440, 460, 498 | 0.000135079 |
| 440, 445, 446, 450, 452, 455, 460, 477, 478, 481, 484, 486, 498 | 0.000114298 |
| 440, 445, 446, 477, 478, 481, 484, 486, 498, 501, 505 | 0.000114298 |
| 501, 505 | 0.000114298 |
| 440, 445, 446, 450, 452, 455, 460, 477, 478, 482, 484, 486, 498, 501, 505 | 0.000114298 |
| 440, 445, 446, 450, 455, 456, 460, 477, 478, 481, 498, 501, 505 | 0.000114298 |
| 440, 444, 445, 446, 450, 452, 455, 456, 460, 477, 478, 481, 484, 486, 498, 501, 505 | 0.000114298 |
| 440, 445, 446, 450, 452, 455, 460, 477, 478 | 0.000103907 |
| 450, 452, 455, 456, 460, 477, 478, 481, 484, 486, 498, 501, 505 | 0.000103907 |
| 440, 445, 446, 450, 452, 455, 460, 477, 478, 481, 484, 486, 498, 501, 503, 505 | 9.35162E-05 |
| 460 | 9.35162E-05 |
| 440, 445, 446, 450, 460, 477, 478, 481, 484, 486, 498, 501, 505 | 9.35162E-05 |
| 501 | 9.35162E-05 |
| 440, 445, 446, 450, 452, 455, 456, 460, 477, 478, 481, 484, 498, 501, 505 | 9.35162E-05 |
| 440, 445, 446, 450, 452, 455, 456, 460, 477, 478, 481, 486, 498, 501, 505 | 9.35162E-05 |
| 440, 445, 446, 477, 478, 486, 498, 501, 505 | 8.31255E-05 |
| 440, 445, 446, 450, 452, 455, 460, 470, 477, 478, 481, 484, 486, 498, 501, 505 | 8.31255E-05 |
| 440, 460, 477, 478, 481, 484, 486, 498, 501, 505 | 8.31255E-05 |
| 440, 445, 446, 450, 452, 455, 460, 477, 478, 481, 484, 485, 486, 498, 501, 505 | 8.31255E-05 |
| 440, 445, 446, 450, 452, 455, 456, 460, 477, 478, 481, 484, 486, 498, 500, 501, 505 | 8.31255E-05 |
| 450, 477, 478, 481, 484, 486, 498, 501, 505 | 7.27348E-05 |
| 440, 455, 460, 477, 478, 481, 484, 486, 498, 501, 505 | 7.27348E-05 |
| 498 | 7.27348E-05 |
| 440, 445, 446, 450, 452, 455, 477, 478, 498, 501, 505 | 7.27348E-05 |
| 440 | 7.27348E-05 |
| 440, 445, 446, 450, 452, 455, 458, 460, 477, 478, 481, 484, 486, 498, 501, 505 | 7.27348E-05 |
| 440, 460, 498, 505 | 7.27348E-05 |
| 455, 460, 477, 478, 498, 501, 505 | 6.23441E-05 |
| 440, 445, 446, 450, 452, 455, 498, 501, 505 | 6.23441E-05 |
| 455, 460, 477, 478, 481, 484, 486, 498, 501 | 6.23441E-05 |
| 455, 460, 477, 478, 481, 484, 486 | 6.23441E-05 |
| 440, 445, 446, 450, 452, 455, 460, 462, 477, 478, 481, 484, 486, 498, 501, 505 | 6.23441E-05 |
| 460, 477, 478 | 6.23441E-05 |
| 440, 445, 446, 450, 452, 455, 460, 477, 478, 481, 484, 486, 501, 505 | 6.23441E-05 |
| 455, 498, 501, 505 | 6.23441E-05 |
| 440, 446, 450, 452, 455, 456, 460, 477, 478, 481, 484, 486, 498, 501, 505 | 6.23441E-05 |
| 440, 445, 446, 450, 452, 455, 456, 460, 477, 478, 482, 483, 484, 486, 498, 501, 505 | 6.23441E-05 |
| 440, 445, 446, 450, 455, 460, 477, 478, 486, 498, 501, 505 | 5.19534E-05 |
| 460, 477, 478, 481 | 5.19534E-05 |
| 440, 445, 477, 478, 481, 484, 486, 498, 501, 505 | 5.19534E-05 |
| 440, 445, 446, 450, 452, 455, 460, 477, 478, 483, 484, 486, 498, 501, 505 | 5.19534E-05 |
| 440, 445, 446, 450, 452, 477, 478, 481, 484, 486, 498, 501, 505 | 5.19534E-05 |
| 440, 445, 446, 452, 455, 460, 477, 478, 481, 484, 486, 498, 501, 505 | 5.19534E-05 |
| 450, 455, 460, 477, 478, 481, 484, 486, 498, 501, 505 | 5.19534E-05 |
| 440, 445, 446, 460, 477, 478, 481, 484, 486, 498, 501, 505 | 5.19534E-05 |
| 440, 450, 460, 477, 478, 481, 484, 486, 498, 501, 505 | 5.19534E-05 |
| 477, 478, 481 | 5.19534E-05 |
| 440, 460, 505 | 5.19534E-05 |
| 445, 446, 450, 452, 455, 456, 460, 477, 478, 481, 484, 486, 498, 501, 505 | 5.19534E-05 |
| 440, 445, 446, 450, 452, 455, 456, 459, 460, 477, 478, 481, 484, 486, 498, 501, 505 | 5.19534E-05 |
| 440, 445, 446, 460, 477, 478, 486, 498, 501, 505 | 4.15628E-05 |
| 445, 446, 450, 452, 455, 460, 477, 478, 481, 484, 486 | 4.15628E-05 |
| 450, 455, 498, 501, 505 | 4.15628E-05 |
| 440, 445, 446, 450, 452, 455 | 4.15628E-05 |
| 477, 478, 481, 498, 501, 505 | 4.15628E-05 |
| 440, 445, 446, 450, 452, 455, 460, 477, 478, 481, 484, 486, 487, 498, 501, 505 | 4.15628E-05 |
| 460, 477, 478, 498, 501, 505 | 4.15628E-05 |
| 477, 478, 481, 484, 486, 498, 501 | 4.15628E-05 |
| 440, 450, 455, 460, 477, 478, 481, 484, 486, 498, 501, 505 | 4.15628E-05 |
| 440, 445, 446, 450, 452, 498, 501, 505 | 4.15628E-05 |
| 440, 445, 446, 450, 455, 460, 477, 478, 484, 486, 498, 501, 505 | 4.15628E-05 |
| 440, 445, 446, 450, 452, 455, 460, 477, 478, 481, 484, 486, 494, 498, 501, 505 | 4.15628E-05 |
| 440, 450, 455, 460, 477, 478, 498, 501, 505 | 4.15628E-05 |
| 450, 452, 455, 460, 477, 478, 498, 501, 505 | 4.15628E-05 |
| 440, 445, 446, 450, 452, 455, 460, 477, 498, 501, 505 | 4.15628E-05 |
| 440, 445, 446, 450, 452, 456, 460, 477, 478, 481, 484, 486, 498, 501, 505 | 4.15628E-05 |
| 440, 445, 446, 450, 452, 455, 456, 460, 477, 478, 481, 498, 501, 505 | 4.15628E-05 |
| 440, 445, 446, 450, 452, 455, 460, 477, 478, 484, 498, 501, 505 | 4.15628E-05 |
| 440, 450, 452, 455, 456, 460, 477, 478, 481, 484, 486, 498, 501, 505 | 4.15628E-05 |
| 440, 445, 446, 450, 452, 455, 456, 460, 477, 478, 484, 486, 498, 501, 505 | 4.15628E-05 |
| 440, 445, 446, 450, 460, 477, 478, 486, 498, 501, 505 | 3.11721E-05 |
| 440, 445, 460, 477, 478, 481, 484, 486, 498, 501, 505 | 3.11721E-05 |
| 440, 445, 446, 456, 460, 477, 478, 484, 486, 490, 498, 501, 505 | 3.11721E-05 |
| 440, 445, 446, 452, 460, 477, 478, 484, 486, 498, 501, 505 | 3.11721E-05 |
| 440, 445, 446, 452, 456, 460, 477, 478, 484, 486, 490, 498, 501, 505 | 3.11721E-05 |
| 440, 460, 477, 478, 486, 498, 501, 505 | 3.11721E-05 |
| 455, 460, 498, 501, 505 | 3.11721E-05 |
| 440, 445, 446, 450, 452, 455, 459, 460, 477, 478, 481, 484, 486, 498, 501, 505 | 3.11721E-05 |
| 440, 445, 446, 450, 452, 498, 501 | 3.11721E-05 |
| 440, 445, 446, 450, 452, 455, 456, 460, 477, 478, 484, 486, 490, 498, 501, 505 | 3.11721E-05 |
| 455 | 3.11721E-05 |
| 440, 450, 452, 455, 460, 477, 478 | 3.11721E-05 |
| 450, 452, 455, 460, 477, 478, 481, 484, 486 | 3.11721E-05 |
| 440, 445, 446, 450, 477, 478, 481, 484, 486, 498, 501, 505 | 3.11721E-05 |
| 477, 498, 501, 505 | 3.11721E-05 |
| 440, 445, 446, 450, 452, 455, 460, 474, 477, 478, 481, 484, 486, 498, 501, 505 | 3.11721E-05 |
| 440, 450, 477, 478, 481, 484, 486, 498, 501, 505 | 3.11721E-05 |
| 450, 452, 455, 460, 477, 478, 481, 484, 486, 498, 501 | 3.11721E-05 |
| 440, 443, 445, 446, 450, 452, 455, 460, 477, 478, 481, 484, 486, 498, 501, 505 | 3.11721E-05 |
| 440, 450, 452, 455, 460, 477, 478, 481, 484, 486, 498, 501 | 3.11721E-05 |
| 450, 452, 455, 460, 477, 478 | 3.11721E-05 |
| 440, 445, 446, 450, 452, 455, 456, 460, 477, 478, 481, 483, 484, 486, 498, 501, 505 | 3.11721E-05 |
| 450, 455, 460, 477, 478, 498, 501, 505 | 3.11721E-05 |
| 440, 445, 446, 450, 452, 477, 478, 498, 501, 505 | 3.11721E-05 |
| 440, 445, 446, 450, 452, 477, 478, 486, 498, 501, 505 | 3.11721E-05 |
| 440, 445, 446, 450, 452, 453, 455, 460, 477, 478, 481, 484, 486, 498, 501, 505 | 3.11721E-05 |
| 440, 445, 446, 450, 452, 455, 456, 460, 476, 477, 478, 481, 484, 486, 498, 501, 505 | 3.11721E-05 |
| 440, 445, 446, 450, 452, 455, 456, 460, 477, 478, 481, 484, 485, 486, 498, 501, 505 | 3.11721E-05 |
| 440, 445, 446, 455, 456, 460, 477, 478, 484, 486, 490, 498, 501, 505 | 2.07814E-05 |
| 455, 460, 477, 478 | 2.07814E-05 |
| 440, 445, 446, 450, 452, 455, 460, 505 | 2.07814E-05 |
| 440, 455, 460, 477, 478, 498, 501, 505 | 2.07814E-05 |
| 440, 445, 446, 455, 460, 477, 478, 484, 486, 490, 498, 501, 505 | 2.07814E-05 |
| 440, 445, 446, 455, 456, 460, 477, 478, 481, 484, 486, 498, 501, 505 | 2.07814E-05 |
| 450, 452, 455, 477, 478, 481, 484, 486, 498, 501, 505 | 2.07814E-05 |
| 440, 445, 446, 450, 455, 460, 477, 478, 481, 484, 486, 498, 501 | 2.07814E-05 |
| 477, 478, 484, 486, 498, 501, 505 | 2.07814E-05 |
| 440, 445, 446, 450, 477, 478, 486, 498, 501, 505 | 2.07814E-05 |
| 481, 484, 486, 498, 501, 505 | 2.07814E-05 |
| 440, 445, 446, 460, 477, 478, 484, 486, 498, 501, 505 | 2.07814E-05 |
| 440, 445, 446, 450, 452, 460, 477, 478, 486, 498, 501, 505 | 2.07814E-05 |
| 486, 498, 501, 505 | 2.07814E-05 |
| 440, 445, 446, 450, 452, 455, 460, 477, 478, 498, 501 | 2.07814E-05 |
| 450, 452 | 2.07814E-05 |
| 440, 445, 446, 450, 452, 455, 460, 477, 478, 481, 484, 486, 502, 503 | 2.07814E-05 |
| 460, 498, 501, 505 | 2.07814E-05 |
| 460, 477, 478, 481, 484, 486, 498, 501 | 2.07814E-05 |
| 440, 445, 446, 452, 460, 477, 478, 486, 498, 501, 505 | 2.07814E-05 |
| 440, 450, 455, 460, 477, 478, 481, 484, 486, 498 | 2.07814E-05 |
| 440, 445, 446, 450, 452, 455, 460, 477, 478, 501, 505 | 2.07814E-05 |
| 450, 452, 455, 460, 477, 478, 481 | 2.07814E-05 |
| 455, 477, 478, 481, 484, 486, 498, 501, 505 | 2.07814E-05 |
| 440, 450, 452, 460, 477, 478 | 2.07814E-05 |
| 450, 460, 477, 478 | 2.07814E-05 |
| 445, 446, 450, 452, 455, 477, 478, 481, 484, 486, 498, 501, 505 | 2.07814E-05 |
| 440, 445, 446, 450, 452, 455, 460, 477, 478, 481, 484, 486, 505 | 2.07814E-05 |
| 450, 460, 477, 478, 481, 484, 486, 498, 501, 505 | 2.07814E-05 |
| 440, 445, 450, 452, 455, 460, 477, 478, 481, 484, 486, 498, 501, 505 | 2.07814E-05 |
| 450, 452, 477, 478, 481, 484, 486, 498, 501, 505 | 2.07814E-05 |
| 440, 446, 450, 452, 455, 460, 477, 478, 498, 501, 505 | 2.07814E-05 |
| 445, 446, 450, 452, 455, 460, 477, 478, 481, 498, 501, 505 | 2.07814E-05 |
| 477, 478, 498, 501 | 2.07814E-05 |
| 440, 445, 446, 450, 452, 455, 460, 477, 478, 481, 484, 486, 501 | 2.07814E-05 |
| 440, 498, 501, 505 | 2.07814E-05 |
| 440, 445, 446, 450, 452, 455, 460, 486, 498, 501, 505 | 2.07814E-05 |
| 455, 460 | 2.07814E-05 |
| 455, 460, 477, 478, 481, 498, 501, 505 | 2.07814E-05 |
| 440, 445, 446, 450, 452, 455, 460, 477, 478, 481, 485, 498, 501, 505 | 2.07814E-05 |
| 460, 498 | 2.07814E-05 |
| 440, 450, 455, 477, 478, 481, 484, 486, 498, 501, 505 | 2.07814E-05 |
| 477, 478, 481, 486, 498, 501, 505 | 2.07814E-05 |
| 477, 478, 501 | 2.07814E-05 |
| 440, 445, 446, 450, 452, 455, 460, 477, 478, 481, 484, 486, 498, 501, 505, 508 | 2.07814E-05 |
| 440, 450, 452, 455, 477, 478, 481, 484, 486, 498, 501, 505 | 2.07814E-05 |
| 440, 445, 446, 450, 452, 460, 477, 478, 481, 484, 498, 501, 505 | 2.07814E-05 |
| 440, 445, 446, 450, 452, 455, 477, 478, 486, 498, 501, 505 | 2.07814E-05 |
| 445, 446, 450, 452, 498, 501, 505 | 2.07814E-05 |
| 440, 445, 446, 450, 452, 477, 478, 484, 486, 498, 501, 505 | 2.07814E-05 |
| 450, 452, 455, 460, 498, 501, 505 | 2.07814E-05 |
| 440, 445, 446, 450, 452, 455, 460, 477, 478, 481, 484, 486, 498, 501, 504, 505 | 2.07814E-05 |
| 440, 477, 478, 498, 501, 505 | 2.07814E-05 |
| 477, 478, 481, 484, 498, 501, 505 | 2.07814E-05 |
| 440, 445, 446, 450, 452, 455, 460, 477, 478, 481, 484, 486, 493, 498, 501, 505 | 2.07814E-05 |
| 450, 452, 455, 456, 460, 477, 478, 498, 501, 505 | 2.07814E-05 |
| 440, 445, 446, 450, 452, 455, 456, 460, 477, 478, 482, 484, 486, 498, 501, 505 | 2.07814E-05 |
| 455, 456, 460 | 2.07814E-05 |
| 455, 456, 460, 477, 478, 481, 484, 486, 498, 501, 505 | 2.07814E-05 |
| 440, 445, 446, 455, 456, 460, 477, 478, 481, 498, 501, 505 | 2.07814E-05 |
| 440, 445, 446, 450, 452, 453, 455, 456, 460, 477, 478, 481, 484, 486, 498, 501, 505 | 2.07814E-05 |
| 440, 445, 446, 450, 452, 455, 456, 498, 501, 505 | 2.07814E-05 |
| 440, 445, 446, 450, 452, 455, 456, 460, 468, 477, 478, 481, 484, 486, 498, 501, 505 | 2.07814E-05 |
| 450, 452, 455, 456, 498, 501, 505 | 2.07814E-05 |
| 440, 445, 446, 450, 452, 455, 456, 460, 477, 498, 501, 505 | 2.07814E-05 |
| 440, 445, 446, 450, 455, 456, 460, 477, 478, 481, 484, 486, 498, 501, 505 | 2.07814E-05 |
| 456, 460, 477, 478, 481, 484, 486, 498, 501, 505 | 2.07814E-05 |
| 440, 445, 446, 450, 452, 455, 456, 460, 477, 478, 479, 481, 484, 486, 498, 501, 505 | 2.07814E-05 |
| 440, 445, 446, 450, 452, 460, 477, 478, 498, 501, 505 | 1.03907E-05 |
| 440, 445, 446, 456, 460, 484, 486, 490, 498, 501, 505 | 1.03907E-05 |
| 440, 452, 460, 477, 478, 481, 484, 486 | 1.03907E-05 |
| 440, 445, 446, 450, 452, 455, 460, 477, 478, 481, 484, 501, 505 | 1.03907E-05 |
| 440, 445, 446, 477, 486, 498, 501, 505 | 1.03907E-05 |
| 440, 445, 446, 450, 452, 455, 460, 498, 501 | 1.03907E-05 |
| 440, 445, 477 | 1.03907E-05 |
| 440, 445, 446, 460, 477, 478, 501, 505 | 1.03907E-05 |
| 440, 450, 460, 477, 478, 486, 498, 501, 505 | 1.03907E-05 |
| 445, 446, 450, 452, 455, 460, 477, 478, 481, 484, 486, 498 | 1.03907E-05 |
| 440, 445, 446, 477, 478, 498, 501, 505 | 1.03907E-05 |
| 440, 450, 452, 455, 460, 477, 478, 481, 484, 486 | 1.03907E-05 |
| 440, 445, 446, 477, 498, 501, 505 | 1.03907E-05 |
| 455, 456, 475, 477, 478, 484, 486, 490 | 1.03907E-05 |
| 440, 441, 445, 446, 450, 455, 460, 477, 478, 481, 484, 486, 498, 501, 505 | 1.03907E-05 |
| 440, 445, 446, 450, 452, 455, 460, 475, 477, 478, 498, 501, 505 | 1.03907E-05 |
| 440, 445, 446, 450, 452, 455, 477, 498, 501, 505 | 1.03907E-05 |
| 450, 455, 477, 478, 481, 484, 486, 498, 501, 505 | 1.03907E-05 |
| 440, 445, 446, 452, 477, 478, 481, 484, 486, 498, 501, 505 | 1.03907E-05 |
| 440, 450, 455, 498, 501, 505 | 1.03907E-05 |
| 440, 445, 446, 452, 455, 460, 477, 478, 484, 486, 498, 501, 505 | 1.03907E-05 |
| 440, 445, 446, 450, 477, 486, 498, 501, 505 | 1.03907E-05 |
| 452, 455, 460, 477, 478, 481, 484, 486 | 1.03907E-05 |
| 440, 445, 446, 452, 455, 477, 478, 481, 484, 486, 498, 501, 505 | 1.03907E-05 |
| 440, 455, 460, 477, 478, 481, 486, 498, 501, 505 | 1.03907E-05 |
| 450, 452, 455, 498, 501 | 1.03907E-05 |
| 440, 445, 446, 450, 452, 455, 475, 477, 478, 481, 484, 486 | 1.03907E-05 |
| 440, 452, 455, 460, 477, 478, 481, 484, 486 | 1.03907E-05 |
| 440, 445, 446, 450, 455, 460, 477, 478, 481, 484, 486 | 1.03907E-05 |
| 440, 445, 446, 450, 455, 477, 478, 486, 498, 501, 505 | 1.03907E-05 |
| 440, 445, 446, 460, 477, 478, 484, 486, 490, 498, 501, 505 | 1.03907E-05 |
| 450, 455, 460, 477, 478 | 1.03907E-05 |
| 440, 445, 446, 450, 455, 460, 477, 478, 481, 484, 486, 501, 505 | 1.03907E-05 |
| 440, 446, 455, 460, 477, 478, 481, 484, 486, 498, 501 | 1.03907E-05 |
| 476, 477, 478, 481, 484, 486, 498, 501, 505 | 1.03907E-05 |
| 440, 445, 446, 450, 452, 455, 476, 477, 478, 481, 484, 486, 498, 501, 505 | 1.03907E-05 |
| 440, 445, 446, 456, 460 | 1.03907E-05 |
| 440, 450, 477, 478, 481, 484, 486 | 1.03907E-05 |
| 450, 455, 477, 498, 501, 505 | 1.03907E-05 |
| 440, 445, 446, 450, 452, 455, 460, 478, 484, 486, 498, 501, 505 | 1.03907E-05 |
| 440, 445, 446, 450, 452, 455, 477, 478, 481, 498, 501, 505 | 1.03907E-05 |
| 440, 445, 446, 450, 455, 460, 498, 501, 505 | 1.03907E-05 |
| 460, 477, 478, 484, 498 | 1.03907E-05 |
| 477, 505 | 1.03907E-05 |
| 440, 445, 446, 450, 452, 455, 460, 477, 478, 481, 484, 486, 498, 501, 507 | 1.03907E-05 |
| 440, 446, 450, 452, 455, 460, 477, 478, 481 | 1.03907E-05 |
| 440, 446, 477, 478, 486, 498, 501, 505 | 1.03907E-05 |
| 440, 445, 446, 450, 452, 455, 460, 477, 478, 479, 498, 501, 505 | 1.03907E-05 |
| 455, 460, 477, 478, 481, 498 | 1.03907E-05 |
| 440, 445, 446, 450, 452, 455, 460, 507 | 1.03907E-05 |
| 440, 445, 446, 450, 452, 460, 477, 478, 481 | 1.03907E-05 |
| 484 | 1.03907E-05 |
| 477, 478, 481, 484, 486, 498, 505 | 1.03907E-05 |
| 440, 445, 446, 456, 460, 477, 478, 484, 486, 498, 501, 505 | 1.03907E-05 |
| 450, 452, 455, 460 | 1.03907E-05 |
| 440, 445, 446, 450, 452 | 1.03907E-05 |
| 507 | 1.03907E-05 |
| 446, 450, 452, 455, 460, 477, 478, 498, 501, 505 | 1.03907E-05 |
| 440, 446, 450, 452, 455, 460, 477, 478, 484, 486, 490, 498, 501, 505 | 1.03907E-05 |
| 440, 445, 446, 452, 477, 486, 498, 501, 505 | 1.03907E-05 |
| 440, 445, 446, 450, 455, 460, 477, 478, 481 | 1.03907E-05 |
| 440, 445, 446, 452, 455, 477, 478, 481, 486, 498, 501, 505 | 1.03907E-05 |
| 440, 450, 452, 455, 477, 478, 498, 501, 505 | 1.03907E-05 |
| 440, 445, 446, 452, 455, 460, 477, 478, 486, 498, 501, 505 | 1.03907E-05 |
| 477, 478, 498 | 1.03907E-05 |
| 477, 478, 481, 501 | 1.03907E-05 |
| 450, 452, 455, 460, 498, 501 | 1.03907E-05 |
| 440, 445, 446, 450, 460, 477, 478, 481, 486, 498, 501, 505 | 1.03907E-05 |
| 445, 446, 450, 452, 455, 477, 478, 498, 501, 505 | 1.03907E-05 |
| 440, 446, 460, 477, 478, 484, 486, 498, 501, 505 | 1.03907E-05 |
| 440, 445, 450, 452, 455, 498, 501, 505 | 1.03907E-05 |
| 440, 450, 452, 455, 460, 477, 478, 498, 501, 505 | 1.03907E-05 |
| 445, 446, 450, 452, 460, 477, 478, 481, 484, 486, 498, 501, 505 | 1.03907E-05 |
| 445, 446, 452, 455, 460, 477, 478, 481, 484, 486, 498, 501, 505 | 1.03907E-05 |
| 477, 478, 481, 484, 486, 501 | 1.03907E-05 |
| 440, 445, 446, 450, 460, 477, 478, 484, 486, 498, 501, 505 | 1.03907E-05 |
| 440, 446, 452, 460, 477, 478, 486, 498, 501, 505 | 1.03907E-05 |
| 440, 446, 460, 477, 478, 486, 498, 501 | 1.03907E-05 |
| 450 | 1.03907E-05 |
| 440, 445, 446, 450, 452, 455, 460, 477, 478, 482, 483, 484, 486, 498, 501 | 1.03907E-05 |
| 440, 445, 446, 452, 460, 477, 478, 484, 486, 490, 498, 501, 505 | 1.03907E-05 |
| 455, 477, 478 | 1.03907E-05 |
| 450, 498, 501, 505 | 1.03907E-05 |
| 446, 450, 452, 455, 460, 477, 478, 481, 484, 486, 498, 501 | 1.03907E-05 |
| 440, 446, 455, 460, 477, 478, 486, 498, 501, 505 | 1.03907E-05 |
| 440, 445, 450, 498, 501, 505 | 1.03907E-05 |
| 445, 446, 450, 452, 455, 460, 498, 501, 505 | 1.03907E-05 |
| 478, 481, 501, 505 | 1.03907E-05 |
| 440, 445, 452, 456, 460, 477, 478, 484, 486, 498, 501, 505 | 1.03907E-05 |
| 445, 446, 450, 452, 455, 460, 477, 478, 481, 486 | 1.03907E-05 |
| 445, 446, 450, 452, 477, 478, 481, 498, 501, 505 | 1.03907E-05 |
| 440, 445, 446, 450, 452, 455, 477, 478, 481, 486, 498, 501, 505 | 1.03907E-05 |
| 477 | 1.03907E-05 |
| 460, 477, 478, 481, 484, 486, 498 | 1.03907E-05 |
| 445, 446, 450, 452, 460, 477, 478, 481, 501, 505 | 1.03907E-05 |
| 440, 445, 446, 450, 451, 452, 455, 460, 477, 478, 498, 501, 505 | 1.03907E-05 |
| 440, 446, 450, 460, 477, 478, 486, 498, 501, 505 | 1.03907E-05 |
| 461, 477, 478, 481, 484, 486, 498, 501, 505 | 1.03907E-05 |
| 445, 446, 450, 477, 478, 481 | 1.03907E-05 |
| 440, 445, 446, 450, 452, 455, 460, 477, 478, 484, 486, 498, 505 | 1.03907E-05 |
| 440, 445, 446, 450, 455, 460, 477, 486, 498, 501, 505 | 1.03907E-05 |
| 440, 445, 446, 450, 455, 460, 477, 478, 481, 486, 498, 501, 505 | 1.03907E-05 |
| 445, 446, 455, 460, 477, 478, 481 | 1.03907E-05 |
| 440, 450, 452, 455, 460, 477, 498, 501, 505 | 1.03907E-05 |
| 440, 445, 446, 450, 452, 455, 477, 486, 498, 501, 505 | 1.03907E-05 |
| 440, 450, 452, 455, 460, 477, 478, 481 | 1.03907E-05 |
| 440, 445, 446, 450, 452, 455, 460, 477, 486, 498, 501, 505 | 1.03907E-05 |
| 440, 445, 446, 450, 455, 477, 486, 498, 501, 505 | 1.03907E-05 |
| 450, 452, 455, 460, 477, 478, 481, 484, 486, 498 | 1.03907E-05 |
| 440, 445, 446, 450, 452, 477, 478, 498, 501 | 1.03907E-05 |
| 460, 505 | 1.03907E-05 |
| 445, 450, 452, 455, 460, 477, 478 | 1.03907E-05 |
| 455, 498 | 1.03907E-05 |
| 440, 445, 446, 450, 452, 455, 460, 477, 478, 486, 498, 501 | 1.03907E-05 |
| 440, 460, 498, 501, 505 | 1.03907E-05 |
| 440, 445, 446, 450, 455, 477, 478, 498, 501, 505 | 1.03907E-05 |
| 440, 445, 446, 450, 452, 455, 460, 477, 478, 481, 484, 498, 501 | 1.03907E-05 |
| 450, 460, 498, 501 | 1.03907E-05 |
| 455, 460, 477, 478, 481, 486, 501 | 1.03907E-05 |
| 440, 450, 455, 460, 477, 478, 481, 498, 501, 505 | 1.03907E-05 |
| 440, 452, 455, 460, 477, 478, 481, 484, 486, 498, 501 | 1.03907E-05 |
| 440, 445, 446, 452, 455, 460, 477, 478, 481, 498, 501, 505 | 1.03907E-05 |
| 440, 445, 446, 450, 455, 477, 478, 481, 484, 486, 498, 501, 505 | 1.03907E-05 |
| 445, 446, 460, 477, 478, 481, 484, 486, 498, 501 | 1.03907E-05 |
| 440, 445, 446, 450, 452, 455, 460, 477, 478, 481, 484, 486, 498, 505 | 1.03907E-05 |
| 440, 441, 445, 446, 450, 452, 455, 460, 477, 478, 481 | 1.03907E-05 |
| 450, 452, 455, 460, 477, 478, 501 | 1.03907E-05 |
| 450, 452, 460, 477, 478, 498, 501, 505 | 1.03907E-05 |
| 440, 450, 452, 455, 460, 477, 478, 481, 498, 501, 505 | 1.03907E-05 |
| 440, 445, 446, 450, 452, 455, 477, 478, 498, 501 | 1.03907E-05 |
| 440, 445, 446, 450, 452, 455, 460, 475, 477, 478, 481 | 1.03907E-05 |
| 440, 445, 446, 450, 452, 455, 456, 460, 475, 477, 478, 481, 484, 498, 501, 505 | 1.03907E-05 |
| 440, 445, 446, 450, 452, 455, 460, 478, 481, 484, 486, 498, 501, 505 | 1.03907E-05 |
| 440, 445, 446, 450, 452, 455, 460, 477, 478, 481, 498, 501 | 1.03907E-05 |
| 440, 445, 446, 450, 452, 455, 460, 477, 478, 481, 484, 486, 498, 499, 501 | 1.03907E-05 |
| 440, 445, 446, 450, 452, 455, 460, 477, 478, 482, 484, 486, 498, 501 | 1.03907E-05 |
| 460, 501 | 1.03907E-05 |
| 455, 460, 505 | 1.03907E-05 |
| 440, 455, 456, 460, 477, 478, 481, 484, 486, 498, 501 | 1.03907E-05 |
| 440, 486, 498, 501, 505 | 1.03907E-05 |
| 445, 446, 450, 452, 455, 460, 477, 478, 481, 501 | 1.03907E-05 |
| 440, 452, 455, 460, 477, 478, 481, 484, 486, 498, 501, 505 | 1.03907E-05 |
| 460, 477, 478, 481, 498, 501, 505 | 1.03907E-05 |
| 440, 445, 446, 450, 452, 455, 460, 461, 477, 478, 481, 484, 486, 498, 501, 505 | 1.03907E-05 |
| 445, 446, 450, 455, 460, 477, 478, 481, 484, 486, 498, 501, 505 | 1.03907E-05 |
| 498, 505 | 1.03907E-05 |
| 440, 445, 446, 450, 452, 455, 456, 460, 486, 498, 501, 505 | 1.03907E-05 |
| 440, 445, 446, 450, 452, 455, 477, 478, 484, 486, 498, 501, 505 | 1.03907E-05 |
| 440, 445, 446, 448, 450, 452, 455, 460, 477, 478, 481, 484, 486, 498, 501, 505 | 1.03907E-05 |
| 477, 478, 481, 484 | 1.03907E-05 |
| 440, 445, 446, 455, 477, 478, 481, 484, 486, 498, 501, 505 | 1.03907E-05 |
| 440, 445, 446, 455, 460, 477, 478, 486, 498, 501, 505 | 1.03907E-05 |
| 440, 445, 446, 450, 452, 455, 460, 477, 478, 481, 484, 486, 498, 499, 501, 505 | 1.03907E-05 |
| 440, 450, 452, 475, 477, 478, 481, 484, 486, 498, 501, 505 | 1.03907E-05 |
| 440, 450, 452, 460, 498, 501, 505 | 1.03907E-05 |
| 440, 445, 446, 450, 452, 460, 477, 478, 481, 484, 486, 498, 501 | 1.03907E-05 |
| 440, 445, 446, 450, 452, 455, 477, 478, 481, 484, 486 | 1.03907E-05 |
| 450, 452, 455 | 1.03907E-05 |
| 450, 452, 455, 460, 477, 478, 498, 501 | 1.03907E-05 |
| 450, 460, 477, 478, 498, 501, 505 | 1.03907E-05 |
| 440, 445, 446, 450, 452, 455, 460, 477, 478, 479, 484, 486, 490, 498, 501, 505 | 1.03907E-05 |
| 440, 450, 452, 477, 478, 481, 484, 486, 498, 501, 505 | 1.03907E-05 |
| 440, 450, 452, 455, 456, 477, 478, 481, 484, 486, 498, 501, 505 | 1.03907E-05 |
| 450, 455, 460 | 1.03907E-05 |
| 440, 445, 446, 450, 452, 455, 460, 477, 478, 481, 483, 484, 485, 486, 498, 501, 505 | 1.03907E-05 |
| 440, 445, 446, 450, 477, 478, 481, 484, 486 | 1.03907E-05 |
| 445, 446, 450, 455, 460, 477, 478, 481, 484, 486, 498, 501 | 1.03907E-05 |
| 450, 455 | 1.03907E-05 |
| 440, 445, 446, 450, 452, 455, 460, 477, 478, 505 | 1.03907E-05 |
| 477, 478, 479, 481, 484, 486, 498, 501, 505 | 1.03907E-05 |
| 440, 445, 446, 450, 452, 455, 460, 477, 478, 481, 484, 486, 498, 501, 505, 507 | 1.03907E-05 |
| 440, 460, 477, 478 | 1.03907E-05 |
| 440, 445, 446, 450, 460 | 1.03907E-05 |
| 440, 445, 446, 450, 452, 455, 460, 477, 478, 481, 484, 493, 498, 501, 505 | 1.03907E-05 |
| 440, 446, 450, 452, 455, 460, 477, 478, 481, 484, 501, 505 | 1.03907E-05 |
| 460, 477 | 1.03907E-05 |
| 440, 445, 446, 450, 452, 477, 478, 481, 486, 498, 501, 505 | 1.03907E-05 |
| 477, 478, 501, 505 | 1.03907E-05 |
| 445, 446, 450, 452 | 1.03907E-05 |
| 440, 445, 446, 450, 452, 455, 460, 477, 478, 498 | 1.03907E-05 |
| 446, 450, 452, 455, 460, 477, 478, 481, 484, 501, 505 | 1.03907E-05 |
| 450, 452, 455, 460, 486, 498, 501, 505 | 1.03907E-05 |
| 440, 445, 446, 450, 452, 455, 456, 460, 477, 478, 481, 484, 486, 498, 501, 503, 505 | 1.03907E-05 |
| 440, 445, 446, 450, 460, 477, 478, 481, 484, 486, 498, 501 | 1.03907E-05 |
| 445, 446, 450, 452, 455, 460, 477, 478, 481, 484, 486, 501, 505 | 1.03907E-05 |
| 440, 450, 455, 460, 477, 478, 481, 484, 486, 498, 501 | 1.03907E-05 |
| 440, 445, 446, 450, 452, 455, 456, 460, 477, 478, 481, 484, 486, 501, 505 | 1.03907E-05 |
| 445, 446, 455, 498, 501, 505 | 1.03907E-05 |
| 446, 450, 452, 455, 460, 477, 478, 481, 484, 486, 498, 501, 505 | 1.03907E-05 |
| 440, 446, 450, 452, 455, 477, 478, 484, 486, 498, 501, 505 | 1.03907E-05 |
| 440, 446, 450, 452, 455, 460, 477, 478, 481, 484, 498, 501, 505 | 1.03907E-05 |
| 440, 445, 446, 450, 452, 455, 456, 460, 461, 477, 478, 481, 484, 486, 498, 501, 505 | 1.03907E-05 |
| 445, 446, 477, 478, 505 | 1.03907E-05 |
| 440, 445, 446, 450, 452, 455, 456, 460, 471, 477, 478, 481, 484, 486, 498, 501, 505 | 1.03907E-05 |
| 440, 445, 446, 452, 455, 460, 477, 478, 481, 484, 486, 498, 501 | 1.03907E-05 |
| 440, 445, 446, 450, 452, 455, 456, 460, 477, 478, 481, 485, 498, 501, 505 | 1.03907E-05 |
| 452, 455, 460, 477, 478 | 1.03907E-05 |
| 440, 445, 446, 450, 452, 455, 481, 484, 486, 498, 501, 505 | 1.03907E-05 |
| 450, 452, 455, 498, 501, 505 | 1.03907E-05 |
| 440, 445, 446, 450, 452, 455, 456, 460, 477, 478, 481, 484, 486, 498, 505 | 1.03907E-05 |
| 440, 450, 477, 478, 498, 501, 505 | 1.03907E-05 |
| 440, 455, 477, 478, 498, 501, 505 | 1.03907E-05 |
| 503, 505 | 1.03907E-05 |
| 446, 450, 452, 455, 477, 478, 481, 486, 498, 501, 505 | 1.03907E-05 |
| 445, 446, 450, 452, 455, 460, 477, 478, 498, 501, 505 | 1.03907E-05 |
| 440, 445, 446, 450, 452, 455, 460, 463, 477, 478, 481, 484, 486, 498, 501, 505 | 1.03907E-05 |
| 440, 445, 446, 450, 452, 455, 456, 460, 477, 478, 481, 486 | 1.03907E-05 |
| 440, 445, 446, 450, 452, 455, 456, 460, 477, 478, 481, 484, 486, 498, 499, 501, 505 | 1.03907E-05 |
| 452, 455, 456, 460, 477, 478, 481, 484, 486 | 1.03907E-05 |
| 455, 456, 460, 477, 478, 481, 484, 486, 498, 501 | 1.03907E-05 |
| 450, 452, 455, 456, 460, 477, 478, 481, 484, 486 | 1.03907E-05 |
| 440, 450, 452, 455, 456, 498, 501, 505 | 1.03907E-05 |
| 440, 460, 477, 478, 481, 484, 486, 498, 501 | 1.03907E-05 |
| 440, 446, 450, 455, 460, 477, 478, 481, 484, 486, 498, 501, 505 | 1.03907E-05 |
| 440, 445, 446, 450, 452, 455, 456, 477, 478, 481, 484, 486, 498, 501, 505 | 1.03907E-05 |
| 440, 450, 452, 455, 456, 460, 477, 478, 481, 484, 486, 498 | 1.03907E-05 |
| 440, 450, 452, 460, 477, 478, 498, 501, 505 | 1.03907E-05 |
| 440, 450, 455, 456, 460, 477, 478, 481, 484, 486, 498, 501, 503, 505 | 1.03907E-05 |
| 440, 445, 446, 450, 452, 455, 456, 477, 478, 486, 498, 501, 505 | 1.03907E-05 |
| 440, 441, 445, 446, 450, 452, 460, 477, 478, 481, 484, 486, 498, 501, 505 | 1.03907E-05 |
| 455, 456, 460, 477, 481, 484, 486, 498, 501, 505 | 1.03907E-05 |
| 440, 450, 455, 456, 460, 477, 478, 481, 484, 486, 498, 501, 505 | 1.03907E-05 |
| 440, 450, 456, 460, 477, 478 | 1.03907E-05 |
| 445, 446, 450, 452, 455, 456, 460, 477, 478, 498, 501, 505 | 1.03907E-05 |
| 460, 477, 481, 484, 486, 498, 501, 505 | 1.03907E-05 |
| 440, 446, 450, 452, 455, 456, 460, 477, 478, 498, 501, 505 | 1.03907E-05 |
| 460, 477, 478, 481, 498 | 1.03907E-05 |
| 440, 445, 446, 450, 455, 456, 460, 498, 501, 505 | 1.03907E-05 |
| 455, 456, 460, 477, 478, 481, 484, 486 | 1.03907E-05 |
| 440, 455, 460, 477, 478, 486, 498, 501, 505 | 1.03907E-05 |
| 440, 481, 484, 486, 498, 501, 505 | 1.03907E-05 |
| 455, 477, 478, 481, 486, 498, 501, 505 | 1.03907E-05 |
| 440, 445, 446, 450, 452, 455, 456, 460, 473, 477, 478, 481, 484, 486, 498, 501, 505 | 1.03907E-05 |
| 440, 445, 446, 450, 455, 456, 460, 477, 478, 498, 501, 505 | 1.03907E-05 |
| 445, 446, 455, 456, 460, 477, 478, 481, 484, 486, 498, 501, 505 | 1.03907E-05 |
| 440, 450, 452 | 1.03907E-05 |
| 440, 445, 446, 455, 456, 460, 477, 478, 498, 501, 505 | 1.03907E-05 |
| 440, 441, 445, 446, 450, 452, 455, 456, 460, 477, 478, 481, 484, 486 | 1.03907E-05 |
| 440, 445, 446, 450, 498, 501, 505 | 1.03907E-05 |
| 450, 456, 460, 477, 478 | 1.03907E-05 |
| 440, 445, 446, 450, 452, 455, 456, 460, 498, 501, 505 | 1.03907E-05 |
| 456, 460, 477, 478, 481, 486, 498, 501, 505 | 1.03907E-05 |
| 446, 450, 452 | 1.03907E-05 |
| 445, 446, 498, 501, 505 | 1.03907E-05 |
| 440, 452, 455, 456, 460, 498, 501, 505 | 1.03907E-05 |
| 440, 456, 460, 477, 478, 481, 484, 486, 498, 501, 505 | 1.03907E-05 |
| 440, 445, 446, 450, 452, 455, 456, 460, 477, 478 | 1.03907E-05 |
| 440, 452, 455, 456, 460, 477, 478, 498, 501, 505 | 1.03907E-05 |
| 440, 445, 446, 450, 452, 455, 456, 460, 477, 478, 486, 498, 501, 505 | 1.03907E-05 |
| 460, 477, 478, 481, 486, 498, 501, 505 | 1.03907E-05 |
| 455, 456, 460, 477, 478, 481 | 1.03907E-05 |
| 440, 450, 455, 456, 460 | 1.03907E-05 |
| 450, 477, 478, 481, 498, 501, 505 | 1.03907E-05 |
| 460, 477, 498, 501, 505 | 1.03907E-05 |
| 460, 477, 478, 498, 501 | 1.03907E-05 |
| 440, 445, 446, 450, 452, 455, 456, 460, 477, 478, 481, 484, 486, 490, 498, 501, 505 | 1.03907E-05 |
| 440, 441, 445, 446, 450, 452, 455, 456, 460 | 1.03907E-05 |
| 450, 455, 456, 460, 477, 478, 481, 484, 486 | 1.03907E-05 |
| 440, 455, 456, 460, 477, 478, 481, 484, 486, 498, 501, 505 | 1.03907E-05 |
| 460, 477, 478, 486, 498, 501 | 1.03907E-05 |
| 460, 477, 478, 481, 484, 486, 505 | 1.03907E-05 |
| 440, 450, 452, 455, 456, 460, 477, 478, 481, 484, 486, 498, 501 | 1.03907E-05 |
| 440, 446, 477, 478, 484, 498, 501, 505 | 1.03907E-05 |
| 440, 445, 446, 450, 452, 455, 456, 460, 474, 477, 478, 481, 484, 486, 498, 501, 505 | 1.03907E-05 |
| 440, 445, 446, 450, 452, 455, 456, 460, 477, 478, 481, 484, 486, 498, 501, 504, 505 | 1.03907E-05 |
| 452, 455, 456, 460, 477, 478, 481, 484, 486, 498, 501, 505 | 1.03907E-05 |
| 440, 445, 450, 452, 455, 456, 460, 477, 478, 481, 484, 486, 493, 498, 501, 505 | 1.03907E-05 |
| 440, 446, 450, 452, 455, 460, 477, 478, 481, 484, 486, 501, 505 | 1.03907E-05 |
| 455, 456, 460, 477, 478, 481, 498, 501, 505 | 1.03907E-05 |
| 440, 445, 446, 450, 452, 455, 456, 460, 462, 477, 478, 481, 484, 486 | 1.03907E-05 |
| 440, 445, 446, 450, 452, 455, 456, 460, 470, 477, 478, 481, 484, 486, 498, 501, 505 | 1.03907E-05 |
| 440, 452, 455, 456, 460, 477, 478, 481, 484, 486, 498 | 1.03907E-05 |
| 440, 445, 446, 450, 452, 455, 456, 460, 477, 478, 498, 501 | 1.03907E-05 |
| **Training set 5** | |
| **mutation combination** | **frequency** |
| 440, 445, 446, 450, 452, 455, 460, 477, 478, 481, 484, 486, 498, 501, 505 | 0.740409393 |
| 440, 445, 446, 450, 452, 455, 456, 460, 477, 478, 481, 484, 486, 498, 501, 505 | 0.182741064 |
| N/A | 0.033697007 |
| 477, 478, 481, 484, 486, 498, 501, 505 | 0.006494181 |
| 440, 445, 446, 450, 452, 455, 460, 477, 478, 481, 484, 486, 498, 501 | 0.004291355 |
| 440, 445, 446, 450, 452, 455, 460, 477, 478, 481, 486, 498, 501, 505 | 0.001818371 |
| 440, 445, 446, 450, 452, 455, 460, 477, 478, 481, 484, 486 | 0.001724855 |
| 440, 445, 446, 450, 452, 460, 477, 478, 481, 484, 486, 498, 501, 505 | 0.001704073 |
| 498, 501, 505 | 0.001381962 |
| 440, 445, 446, 450, 452, 455, 460, 477, 478, 498, 501, 505 | 0.001007897 |
| 440, 441, 445, 446, 450, 452, 455, 460, 477, 478, 481, 484, 486, 498, 501, 505 | 0.000924771 |
| 440, 444, 445, 446, 450, 452, 455, 460, 477, 478, 481, 484, 486, 498, 501, 505 | 0.000696176 |
| 440, 445, 446, 450, 452, 455, 456, 460, 477, 481, 484, 486, 498, 501, 505 | 0.000613051 |
| 440, 441, 445, 446, 450, 452, 455, 456, 460, 477, 478, 481, 484, 486, 498, 501, 505 | 0.000550707 |
| 440, 445, 446, 450, 452, 455, 456, 460, 475, 477, 478, 481, 484, 486, 498, 501, 505 | 0.000540316 |
| 440, 445, 446, 450, 452, 455, 460, 477, 478, 486, 498, 501, 505 | 0.000509144 |
| 440, 445, 446, 450, 452, 455, 460, 477, 478, 481, 483, 484, 486, 498, 501, 505 | 0.000498753 |
| 440, 445, 446, 450, 452, 455, 460, 477, 478, 484, 486, 490, 498, 501, 505 | 0.000477972 |
| 440, 445, 446, 450, 452, 455, 456, 460, 477, 478, 481, 484, 486 | 0.000477972 |
| 460, 477, 478, 481, 484, 486, 498, 501, 505 | 0.00045719 |
| 440, 445, 446, 450, 452, 455, 460, 475, 477, 478, 481, 484, 486, 498, 501, 505 | 0.000426018 |
| 440, 445, 446, 450, 452, 455, 460, 477, 478, 484, 486, 498, 501, 505 | 0.000415628 |
| 477, 478, 486, 498, 501, 505 | 0.000415628 |
| 440, 446, 450, 452, 455, 460, 477, 478, 481, 484, 486, 498, 501, 505 | 0.000405237 |
| 505 | 0.000405237 |
| 440, 445, 446, 450, 452, 455, 456, 460, 477, 478, 481, 484, 486, 498, 501 | 0.000384456 |
| 440, 445, 446, 450, 452, 455, 460, 477, 478, 481 | 0.000363674 |
| 440, 445, 446, 450, 455, 460, 477, 478, 481, 484, 486, 498, 501, 505 | 0.000353283 |
| 440, 445, 446, 450, 452, 455, 460, 477, 478, 481, 484, 498, 501, 505 | 0.000342893 |
| 440, 445, 446, 450, 452, 455, 477, 478, 481, 484, 486, 498, 501, 505 | 0.000332502 |
| 440, 445, 446, 450, 452, 455, 460, 477, 481, 484, 486, 498, 501, 505 | 0.000322111 |
| 450, 452, 455, 460, 477, 478, 481, 484, 486, 498, 501, 505 | 0.000311721 |
| 440, 450, 452, 455, 460, 477, 478, 481, 484, 486, 498, 501, 505 | 0.00030133 |
| 440, 444, 445, 446, 450, 452, 453, 455, 460, 477, 478, 481, 484, 486, 498, 501, 505 | 0.00030133 |
| 440, 445, 446, 450, 452, 455, 460 | 0.000290939 |
| 477, 478, 498, 501, 505 | 0.000290939 |
| 440, 445, 446, 450, 452, 455, 460, 498, 501, 505 | 0.000270158 |
| 445, 446, 450, 452, 455, 460, 477, 478, 481, 484, 486, 498, 501, 505 | 0.000270158 |
| 440, 445, 446, 450, 452, 455, 460, 477, 478, 482, 483, 484, 486, 498, 501, 505 | 0.000259767 |
| 440, 445, 446, 450, 452, 455, 460, 477, 478, 479, 481, 484, 486, 498, 501, 505 | 0.000238986 |
| 440, 445, 446, 450, 452, 455, 456, 460, 477, 478, 481, 484, 486, 493, 498, 501, 505 | 0.000238986 |
| 440, 445, 446, 450, 452, 455, 456, 460, 477, 478, 481 | 0.000228595 |
| 440, 445, 446, 450, 452, 455, 460, 477, 478, 481, 498, 501, 505 | 0.000218204 |
| 460, 477, 478, 481, 484, 486 | 0.000218204 |
| 440, 477, 478, 481, 484, 486, 498, 501, 505 | 0.000207814 |
| 440, 460 | 0.000207814 |
| 477, 478, 481, 484, 486 | 0.000207814 |
| 440, 445, 446, 455, 460, 477, 478, 481, 484, 486, 498, 501, 505 | 0.000166251 |
| 440, 445, 446, 450, 452, 455, 460, 477, 478, 482, 486, 498, 501, 505 | 0.00015586 |
| 440, 445, 446, 450, 452, 455, 460, 477, 478, 481, 484, 486, 498, 500, 501, 505 | 0.00015586 |
| 477, 478 | 0.00014547 |
| 440, 445, 446, 450, 452, 455, 460, 476, 477, 478, 481, 484, 486, 498, 501, 505 | 0.00014547 |
| 498, 501 | 0.00014547 |
| 455, 460, 477, 478, 481, 484, 486, 498, 501, 505 | 0.00014547 |
| 440, 445, 446, 450, 452, 455, 456, 460, 477, 478, 498, 501, 505 | 0.000135079 |
| 440, 460, 498 | 0.000135079 |
| 440, 445, 446, 450, 452, 455, 460, 477, 478, 481, 484, 486, 498 | 0.000114298 |
| 440, 445, 446, 477, 478, 481, 484, 486, 498, 501, 505 | 0.000114298 |
| 501, 505 | 0.000114298 |
| 440, 445, 446, 450, 452, 455, 460, 477, 478, 482, 484, 486, 498, 501, 505 | 0.000114298 |
| 440, 445, 446, 450, 455, 456, 460, 477, 478, 481, 498, 501, 505 | 0.000114298 |
| 440, 444, 445, 446, 450, 452, 455, 456, 460, 477, 478, 481, 484, 486, 498, 501, 505 | 0.000114298 |
| 440, 445, 446, 450, 452, 455, 460, 477, 478 | 0.000103907 |
| 450, 452, 455, 456, 460, 477, 478, 481, 484, 486, 498, 501, 505 | 0.000103907 |
| 440, 445, 446, 450, 452, 455, 460, 477, 478, 481, 484, 486, 498, 501, 503, 505 | 9.35162E-05 |
| 460 | 9.35162E-05 |
| 440, 445, 446, 450, 460, 477, 478, 481, 484, 486, 498, 501, 505 | 9.35162E-05 |
| 501 | 9.35162E-05 |
| 440, 445, 446, 450, 452, 455, 456, 460, 477, 478, 481, 484, 498, 501, 505 | 9.35162E-05 |
| 440, 445, 446, 450, 452, 455, 456, 460, 477, 478, 481, 486, 498, 501, 505 | 9.35162E-05 |
| 440, 445, 446, 477, 478, 486, 498, 501, 505 | 8.31255E-05 |
| 440, 445, 446, 450, 452, 455, 460, 470, 477, 478, 481, 484, 486, 498, 501, 505 | 8.31255E-05 |
| 440, 460, 477, 478, 481, 484, 486, 498, 501, 505 | 8.31255E-05 |
| 440, 445, 446, 450, 452, 455, 460, 477, 478, 481, 484, 485, 486, 498, 501, 505 | 8.31255E-05 |
| 440, 445, 446, 450, 452, 455, 456, 460, 477, 478, 481, 484, 486, 498, 500, 501, 505 | 8.31255E-05 |
| 450, 477, 478, 481, 484, 486, 498, 501, 505 | 7.27348E-05 |
| 440, 455, 460, 477, 478, 481, 484, 486, 498, 501, 505 | 7.27348E-05 |
| 498 | 7.27348E-05 |
| 440, 445, 446, 450, 452, 455, 477, 478, 498, 501, 505 | 7.27348E-05 |
| 440 | 7.27348E-05 |
| 440, 445, 446, 450, 452, 455, 458, 460, 477, 478, 481, 484, 486, 498, 501, 505 | 7.27348E-05 |
| 440, 460, 498, 505 | 7.27348E-05 |
| 455, 460, 477, 478, 498, 501, 505 | 6.23441E-05 |
| 440, 445, 446, 450, 452, 455, 498, 501, 505 | 6.23441E-05 |
| 455, 460, 477, 478, 481, 484, 486, 498, 501 | 6.23441E-05 |
| 455, 460, 477, 478, 481, 484, 486 | 6.23441E-05 |
| 440, 445, 446, 450, 452, 455, 460, 462, 477, 478, 481, 484, 486, 498, 501, 505 | 6.23441E-05 |
| 460, 477, 478 | 6.23441E-05 |
| 440, 445, 446, 450, 452, 455, 460, 477, 478, 481, 484, 486, 501, 505 | 6.23441E-05 |
| 455, 498, 501, 505 | 6.23441E-05 |
| 440, 446, 450, 452, 455, 456, 460, 477, 478, 481, 484, 486, 498, 501, 505 | 6.23441E-05 |
| 440, 445, 446, 450, 452, 455, 456, 460, 477, 478, 482, 483, 484, 486, 498, 501, 505 | 6.23441E-05 |
| 440, 445, 446, 450, 455, 460, 477, 478, 486, 498, 501, 505 | 5.19534E-05 |
| 460, 477, 478, 481 | 5.19534E-05 |
| 440, 445, 477, 478, 481, 484, 486, 498, 501, 505 | 5.19534E-05 |
| 440, 445, 446, 450, 452, 455, 460, 477, 478, 483, 484, 486, 498, 501, 505 | 5.19534E-05 |
| 440, 445, 446, 450, 452, 477, 478, 481, 484, 486, 498, 501, 505 | 5.19534E-05 |
| 440, 445, 446, 452, 455, 460, 477, 478, 481, 484, 486, 498, 501, 505 | 5.19534E-05 |
| 450, 455, 460, 477, 478, 481, 484, 486, 498, 501, 505 | 5.19534E-05 |
| 440, 445, 446, 460, 477, 478, 481, 484, 486, 498, 501, 505 | 5.19534E-05 |
| 440, 450, 460, 477, 478, 481, 484, 486, 498, 501, 505 | 5.19534E-05 |
| 477, 478, 481 | 5.19534E-05 |
| 440, 460, 505 | 5.19534E-05 |
| 445, 446, 450, 452, 455, 456, 460, 477, 478, 481, 484, 486, 498, 501, 505 | 5.19534E-05 |
| 440, 445, 446, 460, 477, 478, 486, 498, 501, 505 | 4.15628E-05 |
| 445, 446, 450, 452, 455, 460, 477, 478, 481, 484, 486 | 4.15628E-05 |
| 450, 455, 498, 501, 505 | 4.15628E-05 |
| 440, 445, 446, 450, 452, 455 | 4.15628E-05 |
| 477, 478, 481, 498, 501, 505 | 4.15628E-05 |
| 460, 477, 478, 498, 501, 505 | 4.15628E-05 |
| 477, 478, 481, 484, 486, 498, 501 | 4.15628E-05 |
| 440, 450, 455, 460, 477, 478, 481, 484, 486, 498, 501, 505 | 4.15628E-05 |
| 440, 445, 446, 450, 452, 498, 501, 505 | 4.15628E-05 |
| 440, 445, 446, 450, 455, 460, 477, 478, 484, 486, 498, 501, 505 | 4.15628E-05 |
| 440, 445, 446, 450, 452, 455, 460, 477, 478, 481, 484, 486, 494, 498, 501, 505 | 4.15628E-05 |
| 440, 450, 455, 460, 477, 478, 498, 501, 505 | 4.15628E-05 |
| 450, 452, 455, 460, 477, 478, 498, 501, 505 | 4.15628E-05 |
| 440, 445, 446, 450, 452, 455, 460, 477, 498, 501, 505 | 4.15628E-05 |
| 440, 445, 446, 450, 452, 456, 460, 477, 478, 481, 484, 486, 498, 501, 505 | 4.15628E-05 |
| 440, 445, 446, 450, 452, 455, 456, 460, 477, 478, 481, 498, 501, 505 | 4.15628E-05 |
| 440, 445, 446, 450, 452, 455, 460, 477, 478, 484, 498, 501, 505 | 4.15628E-05 |
| 440, 450, 452, 455, 456, 460, 477, 478, 481, 484, 486, 498, 501, 505 | 4.15628E-05 |
| 440, 445, 446, 450, 452, 455, 456, 460, 477, 478, 484, 486, 498, 501, 505 | 4.15628E-05 |
| 440, 445, 446, 450, 460, 477, 478, 486, 498, 501, 505 | 3.11721E-05 |
| 440, 445, 460, 477, 478, 481, 484, 486, 498, 501, 505 | 3.11721E-05 |
| 440, 445, 446, 456, 460, 477, 478, 484, 486, 490, 498, 501, 505 | 3.11721E-05 |
| 440, 445, 446, 452, 460, 477, 478, 484, 486, 498, 501, 505 | 3.11721E-05 |
| 440, 445, 446, 452, 456, 460, 477, 478, 484, 486, 490, 498, 501, 505 | 3.11721E-05 |
| 440, 460, 477, 478, 486, 498, 501, 505 | 3.11721E-05 |
| 455, 460, 498, 501, 505 | 3.11721E-05 |
| 440, 445, 446, 450, 452, 498, 501 | 3.11721E-05 |
| 440, 445, 446, 450, 452, 455, 456, 460, 477, 478, 484, 486, 490, 498, 501, 505 | 3.11721E-05 |
| 455 | 3.11721E-05 |
| 440, 450, 452, 455, 460, 477, 478 | 3.11721E-05 |
| 450, 452, 455, 460, 477, 478, 481, 484, 486 | 3.11721E-05 |
| 440, 445, 446, 450, 477, 478, 481, 484, 486, 498, 501, 505 | 3.11721E-05 |
| 477, 498, 501, 505 | 3.11721E-05 |
| 440, 445, 446, 450, 452, 455, 460, 474, 477, 478, 481, 484, 486, 498, 501, 505 | 3.11721E-05 |
| 440, 450, 477, 478, 481, 484, 486, 498, 501, 505 | 3.11721E-05 |
| 450, 452, 455, 460, 477, 478, 481, 484, 486, 498, 501 | 3.11721E-05 |
| 440, 450, 452, 455, 460, 477, 478, 481, 484, 486, 498, 501 | 3.11721E-05 |
| 450, 452, 455, 460, 477, 478 | 3.11721E-05 |
| 440, 445, 446, 450, 452, 455, 456, 460, 477, 478, 481, 483, 484, 486, 498, 501, 505 | 3.11721E-05 |
| 450, 455, 460, 477, 478, 498, 501, 505 | 3.11721E-05 |
| 440, 445, 446, 450, 452, 477, 478, 498, 501, 505 | 3.11721E-05 |
| 440, 445, 446, 450, 452, 477, 478, 486, 498, 501, 505 | 3.11721E-05 |
| 440, 445, 446, 450, 452, 453, 455, 460, 477, 478, 481, 484, 486, 498, 501, 505 | 3.11721E-05 |
| 440, 445, 446, 450, 452, 455, 456, 460, 476, 477, 478, 481, 484, 486, 498, 501, 505 | 3.11721E-05 |
| 440, 445, 446, 450, 452, 455, 456, 460, 477, 478, 481, 484, 485, 486, 498, 501, 505 | 3.11721E-05 |
| 440, 445, 446, 455, 456, 460, 477, 478, 484, 486, 490, 498, 501, 505 | 2.07814E-05 |
| 455, 460, 477, 478 | 2.07814E-05 |
| 440, 445, 446, 450, 452, 455, 460, 505 | 2.07814E-05 |
| 440, 455, 460, 477, 478, 498, 501, 505 | 2.07814E-05 |
| 440, 445, 446, 455, 460, 477, 478, 484, 486, 490, 498, 501, 505 | 2.07814E-05 |
| 440, 445, 446, 455, 456, 460, 477, 478, 481, 484, 486, 498, 501, 505 | 2.07814E-05 |
| 450, 452, 455, 477, 478, 481, 484, 486, 498, 501, 505 | 2.07814E-05 |
| 440, 445, 446, 450, 455, 460, 477, 478, 481, 484, 486, 498, 501 | 2.07814E-05 |
| 477, 478, 484, 486, 498, 501, 505 | 2.07814E-05 |
| 440, 445, 446, 450, 477, 478, 486, 498, 501, 505 | 2.07814E-05 |
| 481, 484, 486, 498, 501, 505 | 2.07814E-05 |
| 440, 445, 446, 460, 477, 478, 484, 486, 498, 501, 505 | 2.07814E-05 |
| 440, 445, 446, 450, 452, 460, 477, 478, 486, 498, 501, 505 | 2.07814E-05 |
| 486, 498, 501, 505 | 2.07814E-05 |
| 440, 445, 446, 450, 452, 455, 460, 477, 478, 498, 501 | 2.07814E-05 |
| 450, 452 | 2.07814E-05 |
| 440, 445, 446, 450, 452, 455, 460, 477, 478, 481, 484, 486, 502, 503 | 2.07814E-05 |
| 460, 498, 501, 505 | 2.07814E-05 |
| 460, 477, 478, 481, 484, 486, 498, 501 | 2.07814E-05 |
| 440, 445, 446, 452, 460, 477, 478, 486, 498, 501, 505 | 2.07814E-05 |
| 440, 450, 455, 460, 477, 478, 481, 484, 486, 498 | 2.07814E-05 |
| 440, 445, 446, 450, 452, 455, 460, 477, 478, 501, 505 | 2.07814E-05 |
| 450, 452, 455, 460, 477, 478, 481 | 2.07814E-05 |
| 455, 477, 478, 481, 484, 486, 498, 501, 505 | 2.07814E-05 |
| 440, 450, 452, 460, 477, 478 | 2.07814E-05 |
| 450, 460, 477, 478 | 2.07814E-05 |
| 445, 446, 450, 452, 455, 477, 478, 481, 484, 486, 498, 501, 505 | 2.07814E-05 |
| 440, 445, 446, 450, 452, 455, 460, 477, 478, 481, 484, 486, 505 | 2.07814E-05 |
| 450, 460, 477, 478, 481, 484, 486, 498, 501, 505 | 2.07814E-05 |
| 440, 445, 450, 452, 455, 460, 477, 478, 481, 484, 486, 498, 501, 505 | 2.07814E-05 |
| 450, 452, 477, 478, 481, 484, 486, 498, 501, 505 | 2.07814E-05 |
| 440, 446, 450, 452, 455, 460, 477, 478, 498, 501, 505 | 2.07814E-05 |
| 445, 446, 450, 452, 455, 460, 477, 478, 481, 498, 501, 505 | 2.07814E-05 |
| 477, 478, 498, 501 | 2.07814E-05 |
| 440, 445, 446, 450, 452, 455, 460, 477, 478, 481, 484, 486, 501 | 2.07814E-05 |
| 440, 498, 501, 505 | 2.07814E-05 |
| 440, 445, 446, 450, 452, 455, 460, 486, 498, 501, 505 | 2.07814E-05 |
| 455, 460 | 2.07814E-05 |
| 455, 460, 477, 478, 481, 498, 501, 505 | 2.07814E-05 |
| 440, 445, 446, 450, 452, 455, 460, 477, 478, 481, 485, 498, 501, 505 | 2.07814E-05 |
| 460, 498 | 2.07814E-05 |
| 440, 450, 455, 477, 478, 481, 484, 486, 498, 501, 505 | 2.07814E-05 |
| 477, 478, 481, 486, 498, 501, 505 | 2.07814E-05 |
| 477, 478, 501 | 2.07814E-05 |
| 440, 445, 446, 450, 452, 455, 460, 477, 478, 481, 484, 486, 498, 501, 505, 508 | 2.07814E-05 |
| 440, 450, 452, 455, 477, 478, 481, 484, 486, 498, 501, 505 | 2.07814E-05 |
| 440, 445, 446, 450, 452, 460, 477, 478, 481, 484, 498, 501, 505 | 2.07814E-05 |
| 440, 445, 446, 450, 452, 455, 477, 478, 486, 498, 501, 505 | 2.07814E-05 |
| 445, 446, 450, 452, 498, 501, 505 | 2.07814E-05 |
| 440, 445, 446, 450, 452, 477, 478, 484, 486, 498, 501, 505 | 2.07814E-05 |
| 450, 452, 455, 460, 498, 501, 505 | 2.07814E-05 |
| 440, 445, 446, 450, 452, 455, 460, 477, 478, 481, 484, 486, 498, 501, 504, 505 | 2.07814E-05 |
| 440, 477, 478, 498, 501, 505 | 2.07814E-05 |
| 477, 478, 481, 484, 498, 501, 505 | 2.07814E-05 |
| 440, 445, 446, 450, 452, 455, 460, 477, 478, 481, 484, 486, 493, 498, 501, 505 | 2.07814E-05 |
| 450, 452, 455, 456, 460, 477, 478, 498, 501, 505 | 2.07814E-05 |
| 440, 445, 446, 450, 452, 455, 456, 460, 477, 478, 482, 484, 486, 498, 501, 505 | 2.07814E-05 |
| 455, 456, 460 | 2.07814E-05 |
| 455, 456, 460, 477, 478, 481, 484, 486, 498, 501, 505 | 2.07814E-05 |
| 440, 445, 446, 455, 456, 460, 477, 478, 481, 498, 501, 505 | 2.07814E-05 |
| 440, 445, 446, 450, 452, 453, 455, 456, 460, 477, 478, 481, 484, 486, 498, 501, 505 | 2.07814E-05 |
| 440, 445, 446, 450, 452, 455, 456, 498, 501, 505 | 2.07814E-05 |
| 440, 445, 446, 450, 452, 455, 456, 460, 468, 477, 478, 481, 484, 486, 498, 501, 505 | 2.07814E-05 |
| 450, 452, 455, 456, 498, 501, 505 | 2.07814E-05 |
| 440, 445, 446, 450, 452, 455, 456, 460, 477, 498, 501, 505 | 2.07814E-05 |
| 440, 445, 446, 450, 455, 456, 460, 477, 478, 481, 484, 486, 498, 501, 505 | 2.07814E-05 |
| 456, 460, 477, 478, 481, 484, 486, 498, 501, 505 | 2.07814E-05 |
| 440, 445, 446, 450, 452, 455, 456, 460, 477, 478, 479, 481, 484, 486, 498, 501, 505 | 2.07814E-05 |
| 440, 445, 446, 450, 452, 460, 477, 478, 498, 501, 505 | 1.03907E-05 |
| 440, 445, 446, 456, 460, 484, 486, 490, 498, 501, 505 | 1.03907E-05 |
| 440, 452, 460, 477, 478, 481, 484, 486 | 1.03907E-05 |
| 440, 445, 446, 450, 452, 455, 460, 477, 478, 481, 484, 501, 505 | 1.03907E-05 |
| 440, 445, 446, 477, 486, 498, 501, 505 | 1.03907E-05 |
| 440, 445, 446, 450, 452, 455, 460, 498, 501 | 1.03907E-05 |
| 440, 445, 477 | 1.03907E-05 |
| 440, 445, 446, 460, 477, 478, 501, 505 | 1.03907E-05 |
| 440, 450, 460, 477, 478, 486, 498, 501, 505 | 1.03907E-05 |
| 445, 446, 450, 452, 455, 460, 477, 478, 481, 484, 486, 498 | 1.03907E-05 |
| 440, 445, 446, 477, 478, 498, 501, 505 | 1.03907E-05 |
| 440, 450, 452, 455, 460, 477, 478, 481, 484, 486 | 1.03907E-05 |
| 440, 445, 446, 477, 498, 501, 505 | 1.03907E-05 |
| 455, 456, 475, 477, 478, 484, 486, 490 | 1.03907E-05 |
| 440, 441, 445, 446, 450, 455, 460, 477, 478, 481, 484, 486, 498, 501, 505 | 1.03907E-05 |
| 440, 445, 446, 450, 452, 455, 460, 475, 477, 478, 498, 501, 505 | 1.03907E-05 |
| 440, 445, 446, 450, 452, 455, 477, 498, 501, 505 | 1.03907E-05 |
| 450, 455, 477, 478, 481, 484, 486, 498, 501, 505 | 1.03907E-05 |
| 440, 445, 446, 452, 477, 478, 481, 484, 486, 498, 501, 505 | 1.03907E-05 |
| 440, 450, 455, 498, 501, 505 | 1.03907E-05 |
| 440, 445, 446, 452, 455, 460, 477, 478, 484, 486, 498, 501, 505 | 1.03907E-05 |
| 440, 445, 446, 450, 477, 486, 498, 501, 505 | 1.03907E-05 |
| 452, 455, 460, 477, 478, 481, 484, 486 | 1.03907E-05 |
| 440, 445, 446, 452, 455, 477, 478, 481, 484, 486, 498, 501, 505 | 1.03907E-05 |
| 440, 455, 460, 477, 478, 481, 486, 498, 501, 505 | 1.03907E-05 |
| 450, 452, 455, 498, 501 | 1.03907E-05 |
| 440, 445, 446, 450, 452, 455, 475, 477, 478, 481, 484, 486 | 1.03907E-05 |
| 440, 452, 455, 460, 477, 478, 481, 484, 486 | 1.03907E-05 |
| 440, 445, 446, 450, 455, 460, 477, 478, 481, 484, 486 | 1.03907E-05 |
| 440, 445, 446, 450, 455, 477, 478, 486, 498, 501, 505 | 1.03907E-05 |
| 440, 445, 446, 460, 477, 478, 484, 486, 490, 498, 501, 505 | 1.03907E-05 |
| 450, 455, 460, 477, 478 | 1.03907E-05 |
| 440, 445, 446, 450, 455, 460, 477, 478, 481, 484, 486, 501, 505 | 1.03907E-05 |
| 440, 446, 455, 460, 477, 478, 481, 484, 486, 498, 501 | 1.03907E-05 |
| 476, 477, 478, 481, 484, 486, 498, 501, 505 | 1.03907E-05 |
| 440, 445, 446, 450, 452, 455, 476, 477, 478, 481, 484, 486, 498, 501, 505 | 1.03907E-05 |
| 440, 445, 446, 456, 460 | 1.03907E-05 |
| 440, 450, 477, 478, 481, 484, 486 | 1.03907E-05 |
| 450, 455, 477, 498, 501, 505 | 1.03907E-05 |
| 440, 445, 446, 450, 452, 455, 460, 478, 484, 486, 498, 501, 505 | 1.03907E-05 |
| 440, 445, 446, 450, 452, 455, 477, 478, 481, 498, 501, 505 | 1.03907E-05 |
| 440, 445, 446, 450, 455, 460, 498, 501, 505 | 1.03907E-05 |
| 460, 477, 478, 484, 498 | 1.03907E-05 |
| 477, 505 | 1.03907E-05 |
| 440, 446, 450, 452, 455, 460, 477, 478, 481 | 1.03907E-05 |
| 440, 446, 477, 478, 486, 498, 501, 505 | 1.03907E-05 |
| 440, 445, 446, 450, 452, 455, 460, 477, 478, 479, 498, 501, 505 | 1.03907E-05 |
| 455, 460, 477, 478, 481, 498 | 1.03907E-05 |
| 440, 445, 446, 450, 452, 460, 477, 478, 481 | 1.03907E-05 |
| 484 | 1.03907E-05 |
| 477, 478, 481, 484, 486, 498, 505 | 1.03907E-05 |
| 440, 445, 446, 456, 460, 477, 478, 484, 486, 498, 501, 505 | 1.03907E-05 |
| 450, 452, 455, 460 | 1.03907E-05 |
| 440, 445, 446, 450, 452 | 1.03907E-05 |
| 446, 450, 452, 455, 460, 477, 478, 498, 501, 505 | 1.03907E-05 |
| 440, 446, 450, 452, 455, 460, 477, 478, 484, 486, 490, 498, 501, 505 | 1.03907E-05 |
| 440, 445, 446, 452, 477, 486, 498, 501, 505 | 1.03907E-05 |
| 440, 445, 446, 450, 455, 460, 477, 478, 481 | 1.03907E-05 |
| 440, 445, 446, 452, 455, 477, 478, 481, 486, 498, 501, 505 | 1.03907E-05 |
| 440, 450, 452, 455, 477, 478, 498, 501, 505 | 1.03907E-05 |
| 440, 445, 446, 452, 455, 460, 477, 478, 486, 498, 501, 505 | 1.03907E-05 |
| 477, 478, 498 | 1.03907E-05 |
| 477, 478, 481, 501 | 1.03907E-05 |
| 450, 452, 455, 460, 498, 501 | 1.03907E-05 |
| 440, 445, 446, 450, 460, 477, 478, 481, 486, 498, 501, 505 | 1.03907E-05 |
| 445, 446, 450, 452, 455, 477, 478, 498, 501, 505 | 1.03907E-05 |
| 440, 446, 460, 477, 478, 484, 486, 498, 501, 505 | 1.03907E-05 |
| 440, 445, 450, 452, 455, 498, 501, 505 | 1.03907E-05 |
| 440, 450, 452, 455, 460, 477, 478, 498, 501, 505 | 1.03907E-05 |
| 445, 446, 450, 452, 460, 477, 478, 481, 484, 486, 498, 501, 505 | 1.03907E-05 |
| 445, 446, 452, 455, 460, 477, 478, 481, 484, 486, 498, 501, 505 | 1.03907E-05 |
| 477, 478, 481, 484, 486, 501 | 1.03907E-05 |
| 440, 445, 446, 450, 460, 477, 478, 484, 486, 498, 501, 505 | 1.03907E-05 |
| 440, 446, 452, 460, 477, 478, 486, 498, 501, 505 | 1.03907E-05 |
| 440, 446, 460, 477, 478, 486, 498, 501 | 1.03907E-05 |
| 450 | 1.03907E-05 |
| 440, 445, 446, 450, 452, 455, 460, 477, 478, 482, 483, 484, 486, 498, 501 | 1.03907E-05 |
| 440, 445, 446, 452, 460, 477, 478, 484, 486, 490, 498, 501, 505 | 1.03907E-05 |
| 455, 477, 478 | 1.03907E-05 |
| 450, 498, 501, 505 | 1.03907E-05 |
| 446, 450, 452, 455, 460, 477, 478, 481, 484, 486, 498, 501 | 1.03907E-05 |
| 440, 446, 455, 460, 477, 478, 486, 498, 501, 505 | 1.03907E-05 |
| 440, 445, 450, 498, 501, 505 | 1.03907E-05 |
| 445, 446, 450, 452, 455, 460, 498, 501, 505 | 1.03907E-05 |
| 478, 481, 501, 505 | 1.03907E-05 |
| 440, 445, 452, 456, 460, 477, 478, 484, 486, 498, 501, 505 | 1.03907E-05 |
| 445, 446, 450, 452, 455, 460, 477, 478, 481, 486 | 1.03907E-05 |
| 445, 446, 450, 452, 477, 478, 481, 498, 501, 505 | 1.03907E-05 |
| 440, 445, 446, 450, 452, 455, 477, 478, 481, 486, 498, 501, 505 | 1.03907E-05 |
| 477 | 1.03907E-05 |
| 460, 477, 478, 481, 484, 486, 498 | 1.03907E-05 |
| 445, 446, 450, 452, 460, 477, 478, 481, 501, 505 | 1.03907E-05 |
| 440, 446, 450, 460, 477, 478, 486, 498, 501, 505 | 1.03907E-05 |
| 461, 477, 478, 481, 484, 486, 498, 501, 505 | 1.03907E-05 |
| 445, 446, 450, 477, 478, 481 | 1.03907E-05 |
| 440, 445, 446, 450, 452, 455, 460, 477, 478, 484, 486, 498, 505 | 1.03907E-05 |
| 440, 445, 446, 450, 455, 460, 477, 486, 498, 501, 505 | 1.03907E-05 |
| 440, 445, 446, 450, 455, 460, 477, 478, 481, 486, 498, 501, 505 | 1.03907E-05 |
| 445, 446, 455, 460, 477, 478, 481 | 1.03907E-05 |
| 440, 450, 452, 455, 460, 477, 498, 501, 505 | 1.03907E-05 |
| 440, 445, 446, 450, 452, 455, 477, 486, 498, 501, 505 | 1.03907E-05 |
| 440, 450, 452, 455, 460, 477, 478, 481 | 1.03907E-05 |
| 440, 445, 446, 450, 452, 455, 460, 477, 486, 498, 501, 505 | 1.03907E-05 |
| 440, 445, 446, 450, 455, 477, 486, 498, 501, 505 | 1.03907E-05 |
| 450, 452, 455, 460, 477, 478, 481, 484, 486, 498 | 1.03907E-05 |
| 440, 445, 446, 450, 452, 477, 478, 498, 501 | 1.03907E-05 |
| 460, 505 | 1.03907E-05 |
| 445, 450, 452, 455, 460, 477, 478 | 1.03907E-05 |
| 455, 498 | 1.03907E-05 |
| 440, 445, 446, 450, 452, 455, 460, 477, 478, 486, 498, 501 | 1.03907E-05 |
| 440, 460, 498, 501, 505 | 1.03907E-05 |
| 440, 445, 446, 450, 455, 477, 478, 498, 501, 505 | 1.03907E-05 |
| 440, 445, 446, 450, 452, 455, 460, 477, 478, 481, 484, 498, 501 | 1.03907E-05 |
| 450, 460, 498, 501 | 1.03907E-05 |
| 455, 460, 477, 478, 481, 486, 501 | 1.03907E-05 |
| 440, 450, 455, 460, 477, 478, 481, 498, 501, 505 | 1.03907E-05 |
| 440, 452, 455, 460, 477, 478, 481, 484, 486, 498, 501 | 1.03907E-05 |
| 440, 445, 446, 452, 455, 460, 477, 478, 481, 498, 501, 505 | 1.03907E-05 |
| 440, 445, 446, 450, 455, 477, 478, 481, 484, 486, 498, 501, 505 | 1.03907E-05 |
| 445, 446, 460, 477, 478, 481, 484, 486, 498, 501 | 1.03907E-05 |
| 440, 445, 446, 450, 452, 455, 460, 477, 478, 481, 484, 486, 498, 505 | 1.03907E-05 |
| 440, 441, 445, 446, 450, 452, 455, 460, 477, 478, 481 | 1.03907E-05 |
| 450, 452, 455, 460, 477, 478, 501 | 1.03907E-05 |
| 450, 452, 460, 477, 478, 498, 501, 505 | 1.03907E-05 |
| 440, 450, 452, 455, 460, 477, 478, 481, 498, 501, 505 | 1.03907E-05 |
| 440, 445, 446, 450, 452, 455, 477, 478, 498, 501 | 1.03907E-05 |
| 440, 445, 446, 450, 452, 455, 460, 475, 477, 478, 481 | 1.03907E-05 |
| 440, 445, 446, 450, 452, 455, 456, 460, 475, 477, 478, 481, 484, 498, 501, 505 | 1.03907E-05 |
| 440, 445, 446, 450, 452, 455, 460, 478, 481, 484, 486, 498, 501, 505 | 1.03907E-05 |
| 440, 445, 446, 450, 452, 455, 460, 477, 478, 481, 498, 501 | 1.03907E-05 |
| 440, 445, 446, 450, 452, 455, 460, 477, 478, 481, 484, 486, 498, 499, 501 | 1.03907E-05 |
| 440, 445, 446, 450, 452, 455, 460, 477, 478, 482, 484, 486, 498, 501 | 1.03907E-05 |
| 460, 501 | 1.03907E-05 |
| 455, 460, 505 | 1.03907E-05 |
| 440, 455, 456, 460, 477, 478, 481, 484, 486, 498, 501 | 1.03907E-05 |
| 440, 486, 498, 501, 505 | 1.03907E-05 |
| 445, 446, 450, 452, 455, 460, 477, 478, 481, 501 | 1.03907E-05 |
| 440, 452, 455, 460, 477, 478, 481, 484, 486, 498, 501, 505 | 1.03907E-05 |
| 460, 477, 478, 481, 498, 501, 505 | 1.03907E-05 |
| 440, 445, 446, 450, 452, 455, 460, 461, 477, 478, 481, 484, 486, 498, 501, 505 | 1.03907E-05 |
| 445, 446, 450, 455, 460, 477, 478, 481, 484, 486, 498, 501, 505 | 1.03907E-05 |
| 498, 505 | 1.03907E-05 |
| 440, 445, 446, 450, 452, 455, 456, 460, 486, 498, 501, 505 | 1.03907E-05 |
| 440, 445, 446, 450, 452, 455, 477, 478, 484, 486, 498, 501, 505 | 1.03907E-05 |
| 440, 445, 446, 448, 450, 452, 455, 460, 477, 478, 481, 484, 486, 498, 501, 505 | 1.03907E-05 |
| 477, 478, 481, 484 | 1.03907E-05 |
| 440, 445, 446, 455, 477, 478, 481, 484, 486, 498, 501, 505 | 1.03907E-05 |
| 440, 445, 446, 455, 460, 477, 478, 486, 498, 501, 505 | 1.03907E-05 |
| 440, 445, 446, 450, 452, 455, 460, 477, 478, 481, 484, 486, 498, 499, 501, 505 | 1.03907E-05 |
| 440, 450, 452, 475, 477, 478, 481, 484, 486, 498, 501, 505 | 1.03907E-05 |
| 440, 450, 452, 460, 498, 501, 505 | 1.03907E-05 |
| 440, 445, 446, 450, 452, 460, 477, 478, 481, 484, 486, 498, 501 | 1.03907E-05 |
| 440, 445, 446, 450, 452, 455, 477, 478, 481, 484, 486 | 1.03907E-05 |
| 450, 452, 455 | 1.03907E-05 |
| 450, 452, 455, 460, 477, 478, 498, 501 | 1.03907E-05 |
| 450, 460, 477, 478, 498, 501, 505 | 1.03907E-05 |
| 440, 445, 446, 450, 452, 455, 460, 477, 478, 479, 484, 486, 490, 498, 501, 505 | 1.03907E-05 |
| 440, 450, 452, 477, 478, 481, 484, 486, 498, 501, 505 | 1.03907E-05 |
| 440, 450, 452, 455, 456, 477, 478, 481, 484, 486, 498, 501, 505 | 1.03907E-05 |
| 450, 455, 460 | 1.03907E-05 |
| 440, 445, 446, 450, 452, 455, 460, 477, 478, 481, 483, 484, 485, 486, 498, 501, 505 | 1.03907E-05 |
| 440, 445, 446, 450, 477, 478, 481, 484, 486 | 1.03907E-05 |
| 445, 446, 450, 455, 460, 477, 478, 481, 484, 486, 498, 501 | 1.03907E-05 |
| 450, 455 | 1.03907E-05 |
| 440, 445, 446, 450, 452, 455, 460, 477, 478, 505 | 1.03907E-05 |
| 477, 478, 479, 481, 484, 486, 498, 501, 505 | 1.03907E-05 |
| 440, 460, 477, 478 | 1.03907E-05 |
| 440, 445, 446, 450, 460 | 1.03907E-05 |
| 440, 445, 446, 450, 452, 455, 460, 477, 478, 481, 484, 493, 498, 501, 505 | 1.03907E-05 |
| 440, 446, 450, 452, 455, 460, 477, 478, 481, 484, 501, 505 | 1.03907E-05 |
| 460, 477 | 1.03907E-05 |
| 440, 445, 446, 450, 452, 477, 478, 481, 486, 498, 501, 505 | 1.03907E-05 |
| 477, 478, 501, 505 | 1.03907E-05 |
| 445, 446, 450, 452 | 1.03907E-05 |
| 440, 445, 446, 450, 452, 455, 460, 477, 478, 498 | 1.03907E-05 |
| 446, 450, 452, 455, 460, 477, 478, 481, 484, 501, 505 | 1.03907E-05 |
| 450, 452, 455, 460, 486, 498, 501, 505 | 1.03907E-05 |
| 440, 445, 446, 450, 452, 455, 456, 460, 477, 478, 481, 484, 486, 498, 501, 503, 505 | 1.03907E-05 |
| 440, 445, 446, 450, 460, 477, 478, 481, 484, 486, 498, 501 | 1.03907E-05 |
| 445, 446, 450, 452, 455, 460, 477, 478, 481, 484, 486, 501, 505 | 1.03907E-05 |
| 440, 450, 455, 460, 477, 478, 481, 484, 486, 498, 501 | 1.03907E-05 |
| 440, 445, 446, 450, 452, 455, 456, 460, 477, 478, 481, 484, 486, 501, 505 | 1.03907E-05 |
| 445, 446, 455, 498, 501, 505 | 1.03907E-05 |
| 446, 450, 452, 455, 460, 477, 478, 481, 484, 486, 498, 501, 505 | 1.03907E-05 |
| 440, 446, 450, 452, 455, 477, 478, 484, 486, 498, 501, 505 | 1.03907E-05 |
| 440, 446, 450, 452, 455, 460, 477, 478, 481, 484, 498, 501, 505 | 1.03907E-05 |
| 440, 445, 446, 450, 452, 455, 456, 460, 461, 477, 478, 481, 484, 486, 498, 501, 505 | 1.03907E-05 |
| 445, 446, 477, 478, 505 | 1.03907E-05 |
| 440, 445, 446, 450, 452, 455, 456, 460, 471, 477, 478, 481, 484, 486, 498, 501, 505 | 1.03907E-05 |
| 440, 445, 446, 452, 455, 460, 477, 478, 481, 484, 486, 498, 501 | 1.03907E-05 |
| 440, 445, 446, 450, 452, 455, 456, 460, 477, 478, 481, 485, 498, 501, 505 | 1.03907E-05 |
| 452, 455, 460, 477, 478 | 1.03907E-05 |
| 440, 445, 446, 450, 452, 455, 481, 484, 486, 498, 501, 505 | 1.03907E-05 |
| 450, 452, 455, 498, 501, 505 | 1.03907E-05 |
| 440, 445, 446, 450, 452, 455, 456, 460, 477, 478, 481, 484, 486, 498, 505 | 1.03907E-05 |
| 440, 450, 477, 478, 498, 501, 505 | 1.03907E-05 |
| 440, 455, 477, 478, 498, 501, 505 | 1.03907E-05 |
| 503, 505 | 1.03907E-05 |
| 446, 450, 452, 455, 477, 478, 481, 486, 498, 501, 505 | 1.03907E-05 |
| 445, 446, 450, 452, 455, 460, 477, 478, 498, 501, 505 | 1.03907E-05 |
| 440, 445, 446, 450, 452, 455, 460, 463, 477, 478, 481, 484, 486, 498, 501, 505 | 1.03907E-05 |
| 440, 445, 446, 450, 452, 455, 456, 460, 477, 478, 481, 486 | 1.03907E-05 |
| 440, 445, 446, 450, 452, 455, 456, 460, 477, 478, 481, 484, 486, 498, 499, 501, 505 | 1.03907E-05 |
| 452, 455, 456, 460, 477, 478, 481, 484, 486 | 1.03907E-05 |
| 455, 456, 460, 477, 478, 481, 484, 486, 498, 501 | 1.03907E-05 |
| 450, 452, 455, 456, 460, 477, 478, 481, 484, 486 | 1.03907E-05 |
| 440, 450, 452, 455, 456, 498, 501, 505 | 1.03907E-05 |
| 440, 460, 477, 478, 481, 484, 486, 498, 501 | 1.03907E-05 |
| 440, 446, 450, 455, 460, 477, 478, 481, 484, 486, 498, 501, 505 | 1.03907E-05 |
| 440, 445, 446, 450, 452, 455, 456, 477, 478, 481, 484, 486, 498, 501, 505 | 1.03907E-05 |
| 440, 450, 452, 455, 456, 460, 477, 478, 481, 484, 486, 498 | 1.03907E-05 |
| 440, 450, 452, 460, 477, 478, 498, 501, 505 | 1.03907E-05 |
| 440, 450, 455, 456, 460, 477, 478, 481, 484, 486, 498, 501, 503, 505 | 1.03907E-05 |
| 440, 445, 446, 450, 452, 455, 456, 477, 478, 486, 498, 501, 505 | 1.03907E-05 |
| 440, 441, 445, 446, 450, 452, 460, 477, 478, 481, 484, 486, 498, 501, 505 | 1.03907E-05 |
| 455, 456, 460, 477, 481, 484, 486, 498, 501, 505 | 1.03907E-05 |
| 440, 450, 455, 456, 460, 477, 478, 481, 484, 486, 498, 501, 505 | 1.03907E-05 |
| 440, 450, 456, 460, 477, 478 | 1.03907E-05 |
| 445, 446, 450, 452, 455, 456, 460, 477, 478, 498, 501, 505 | 1.03907E-05 |
| 460, 477, 481, 484, 486, 498, 501, 505 | 1.03907E-05 |
| 440, 446, 450, 452, 455, 456, 460, 477, 478, 498, 501, 505 | 1.03907E-05 |
| 460, 477, 478, 481, 498 | 1.03907E-05 |
| 440, 445, 446, 450, 455, 456, 460, 498, 501, 505 | 1.03907E-05 |
| 455, 456, 460, 477, 478, 481, 484, 486 | 1.03907E-05 |
| 440, 455, 460, 477, 478, 486, 498, 501, 505 | 1.03907E-05 |
| 440, 481, 484, 486, 498, 501, 505 | 1.03907E-05 |
| 455, 477, 478, 481, 486, 498, 501, 505 | 1.03907E-05 |
| 440, 445, 446, 450, 452, 455, 456, 460, 473, 477, 478, 481, 484, 486, 498, 501, 505 | 1.03907E-05 |
| 440, 445, 446, 450, 455, 456, 460, 477, 478, 498, 501, 505 | 1.03907E-05 |
| 445, 446, 455, 456, 460, 477, 478, 481, 484, 486, 498, 501, 505 | 1.03907E-05 |
| 440, 450, 452 | 1.03907E-05 |
| 440, 445, 446, 455, 456, 460, 477, 478, 498, 501, 505 | 1.03907E-05 |
| 440, 441, 445, 446, 450, 452, 455, 456, 460, 477, 478, 481, 484, 486 | 1.03907E-05 |
| 440, 445, 446, 450, 498, 501, 505 | 1.03907E-05 |
| 450, 456, 460, 477, 478 | 1.03907E-05 |
| 440, 445, 446, 450, 452, 455, 456, 460, 498, 501, 505 | 1.03907E-05 |
| 456, 460, 477, 478, 481, 486, 498, 501, 505 | 1.03907E-05 |
| 446, 450, 452 | 1.03907E-05 |
| 445, 446, 498, 501, 505 | 1.03907E-05 |
| 440, 452, 455, 456, 460, 498, 501, 505 | 1.03907E-05 |
| 440, 456, 460, 477, 478, 481, 484, 486, 498, 501, 505 | 1.03907E-05 |
| 440, 445, 446, 450, 452, 455, 456, 460, 477, 478 | 1.03907E-05 |
| 440, 452, 455, 456, 460, 477, 478, 498, 501, 505 | 1.03907E-05 |
| 440, 445, 446, 450, 452, 455, 456, 460, 477, 478, 486, 498, 501, 505 | 1.03907E-05 |
| 460, 477, 478, 481, 486, 498, 501, 505 | 1.03907E-05 |
| 455, 456, 460, 477, 478, 481 | 1.03907E-05 |
| 440, 450, 455, 456, 460 | 1.03907E-05 |
| 450, 477, 478, 481, 498, 501, 505 | 1.03907E-05 |
| 460, 477, 498, 501, 505 | 1.03907E-05 |
| 460, 477, 478, 498, 501 | 1.03907E-05 |
| 440, 445, 446, 450, 452, 455, 456, 460, 477, 478, 481, 484, 486, 490, 498, 501, 505 | 1.03907E-05 |
| 440, 441, 445, 446, 450, 452, 455, 456, 460 | 1.03907E-05 |
| 450, 455, 456, 460, 477, 478, 481, 484, 486 | 1.03907E-05 |
| 440, 455, 456, 460, 477, 478, 481, 484, 486, 498, 501, 505 | 1.03907E-05 |
| 460, 477, 478, 486, 498, 501 | 1.03907E-05 |
| 460, 477, 478, 481, 484, 486, 505 | 1.03907E-05 |
| 440, 450, 452, 455, 456, 460, 477, 478, 481, 484, 486, 498, 501 | 1.03907E-05 |
| 440, 446, 477, 478, 484, 498, 501, 505 | 1.03907E-05 |
| 440, 445, 446, 450, 452, 455, 456, 460, 474, 477, 478, 481, 484, 486, 498, 501, 505 | 1.03907E-05 |
| 440, 445, 446, 450, 452, 455, 456, 460, 477, 478, 481, 484, 486, 498, 501, 504, 505 | 1.03907E-05 |
| 452, 455, 456, 460, 477, 478, 481, 484, 486, 498, 501, 505 | 1.03907E-05 |
| 440, 445, 450, 452, 455, 456, 460, 477, 478, 481, 484, 486, 493, 498, 501, 505 | 1.03907E-05 |
| 440, 446, 450, 452, 455, 460, 477, 478, 481, 484, 486, 501, 505 | 1.03907E-05 |
| 455, 456, 460, 477, 478, 481, 498, 501, 505 | 1.03907E-05 |
| 440, 445, 446, 450, 452, 455, 456, 460, 462, 477, 478, 481, 484, 486 | 1.03907E-05 |
| 440, 445, 446, 450, 452, 455, 456, 460, 470, 477, 478, 481, 484, 486, 498, 501, 505 | 1.03907E-05 |
| 440, 452, 455, 456, 460, 477, 478, 481, 484, 486, 498 | 1.03907E-05 |
| 440, 445, 446, 450, 452, 455, 456, 460, 477, 478, 498, 501 | 1.03907E-05 |
| **Training set 6** | |
| **mutation combination** | **frequency** |
| 440, 445, 446, 450, 452, 455, 460, 477, 478, 481, 484, 486, 498, 501, 505 | 0.740305486 |
| 440, 445, 446, 450, 452, 455, 456, 460, 477, 478, 481, 484, 486, 498, 501, 505 | 0.182689111 |
| N/A | 0.033697007 |
| 477, 478, 481, 484, 486, 498, 501, 505 | 0.006494181 |
| 440, 445, 446, 450, 452, 455, 460, 477, 478, 481, 484, 486, 498, 501 | 0.004291355 |
| 440, 445, 446, 450, 452, 455, 460, 477, 478, 481, 486, 498, 501, 505 | 0.001818371 |
| 440, 445, 446, 450, 452, 455, 460, 477, 478, 481, 484, 486 | 0.001724855 |
| 440, 445, 446, 450, 452, 460, 477, 478, 481, 484, 486, 498, 501, 505 | 0.001704073 |
| 498, 501, 505 | 0.001381962 |
| 440, 445, 446, 450, 452, 455, 460, 477, 478, 498, 501, 505 | 0.001007897 |
| 440, 441, 445, 446, 450, 452, 455, 460, 477, 478, 481, 484, 486, 498, 501, 505 | 0.000924771 |
| 440, 444, 445, 446, 450, 452, 455, 460, 477, 478, 481, 484, 486, 498, 501, 505 | 0.000696176 |
| 440, 445, 446, 450, 452, 455, 456, 460, 477, 481, 484, 486, 498, 501, 505 | 0.000613051 |
| 440, 441, 445, 446, 450, 452, 455, 456, 460, 477, 478, 481, 484, 486, 498, 501, 505 | 0.000550707 |
| 440, 445, 446, 450, 452, 455, 456, 460, 475, 477, 478, 481, 484, 486, 498, 501, 505 | 0.000540316 |
| 440, 445, 446, 450, 452, 455, 460, 477, 478, 486, 498, 501, 505 | 0.000509144 |
| 440, 445, 446, 450, 452, 455, 460, 477, 478, 481, 483, 484, 486, 498, 501, 505 | 0.000498753 |
| 440, 445, 446, 450, 452, 455, 460, 477, 478, 484, 486, 490, 498, 501, 505 | 0.000477972 |
| 440, 445, 446, 450, 452, 455, 456, 460, 477, 478, 481, 484, 486 | 0.000477972 |
| 460, 477, 478, 481, 484, 486, 498, 501, 505 | 0.00045719 |
| 440, 445, 446, 450, 452, 455, 460, 475, 477, 478, 481, 484, 486, 498, 501, 505 | 0.000426018 |
| 440, 445, 446, 450, 452, 455, 460, 477, 478, 484, 486, 498, 501, 505 | 0.000415628 |
| 477, 478, 486, 498, 501, 505 | 0.000415628 |
| 440, 446, 450, 452, 455, 460, 477, 478, 481, 484, 486, 498, 501, 505 | 0.000405237 |
| 505 | 0.000405237 |
| 440, 445, 446, 450, 452, 455, 456, 460, 477, 478, 481, 484, 486, 498, 501 | 0.000384456 |
| 440, 445, 446, 450, 452, 455, 460, 477, 478, 481 | 0.000363674 |
| 440, 445, 446, 450, 455, 460, 477, 478, 481, 484, 486, 498, 501, 505 | 0.000353283 |
| 440, 445, 446, 450, 452, 455, 460, 477, 478, 481, 484, 498, 501, 505 | 0.000342893 |
| 440, 445, 446, 450, 452, 455, 477, 478, 481, 484, 486, 498, 501, 505 | 0.000332502 |
| 440, 445, 446, 450, 452, 455, 460, 477, 481, 484, 486, 498, 501, 505 | 0.000322111 |
| 450, 452, 455, 460, 477, 478, 481, 484, 486, 498, 501, 505 | 0.000311721 |
| 440, 450, 452, 455, 460, 477, 478, 481, 484, 486, 498, 501, 505 | 0.00030133 |
| 440, 444, 445, 446, 450, 452, 453, 455, 460, 477, 478, 481, 484, 486, 498, 501, 505 | 0.00030133 |
| 440, 445, 446, 450, 452, 455, 460 | 0.000290939 |
| 477, 478, 498, 501, 505 | 0.000290939 |
| 440, 445, 446, 450, 452, 455, 460, 498, 501, 505 | 0.000270158 |
| 445, 446, 450, 452, 455, 460, 477, 478, 481, 484, 486, 498, 501, 505 | 0.000270158 |
| 440, 445, 446, 450, 452, 455, 460, 477, 478, 482, 483, 484, 486, 498, 501, 505 | 0.000259767 |
| 440, 445, 446, 450, 452, 455, 460, 477, 478, 479, 481, 484, 486, 498, 501, 505 | 0.000238986 |
| 440, 445, 446, 450, 452, 455, 456, 460, 477, 478, 481, 484, 486, 493, 498, 501, 505 | 0.000238986 |
| 440, 445, 446, 450, 452, 455, 456, 460, 477, 478, 481 | 0.000228595 |
| 440, 445, 446, 450, 452, 455, 460, 477, 478, 481, 498, 501, 505 | 0.000218204 |
| 460, 477, 478, 481, 484, 486 | 0.000218204 |
| 440, 477, 478, 481, 484, 486, 498, 501, 505 | 0.000207814 |
| 440, 460 | 0.000207814 |
| 477, 478, 481, 484, 486 | 0.000207814 |
| 440, 445, 446, 455, 460, 477, 478, 481, 484, 486, 498, 501, 505 | 0.000166251 |
| 440, 445, 446, 450, 452, 455, 460, 477, 478, 482, 486, 498, 501, 505 | 0.00015586 |
| 440, 445, 446, 450, 452, 455, 460, 477, 478, 481, 484, 486, 498, 500, 501, 505 | 0.00015586 |
| 477, 478 | 0.00014547 |
| 440, 445, 446, 450, 452, 455, 460, 476, 477, 478, 481, 484, 486, 498, 501, 505 | 0.00014547 |
| 498, 501 | 0.00014547 |
| 455, 460, 477, 478, 481, 484, 486, 498, 501, 505 | 0.00014547 |
| 440, 445, 446, 450, 452, 455, 456, 460, 477, 478, 498, 501, 505 | 0.000135079 |
| 440, 460, 498 | 0.000135079 |
| 440, 445, 446, 450, 452, 455, 460, 477, 478, 481, 484, 486, 498 | 0.000114298 |
| 440, 445, 446, 477, 478, 481, 484, 486, 498, 501, 505 | 0.000114298 |
| 501, 505 | 0.000114298 |
| 440, 445, 446, 450, 452, 455, 460, 477, 478, 482, 484, 486, 498, 501, 505 | 0.000114298 |
| 440, 445, 446, 450, 455, 456, 460, 477, 478, 481, 498, 501, 505 | 0.000114298 |
| 440, 444, 445, 446, 450, 452, 455, 456, 460, 477, 478, 481, 484, 486, 498, 501, 505 | 0.000114298 |
| 440, 445, 446, 450, 452, 455, 460, 477, 478 | 0.000103907 |
| 450, 452, 455, 456, 460, 477, 478, 481, 484, 486, 498, 501, 505 | 0.000103907 |
| 440, 445, 446, 450, 452, 455, 460, 477, 478, 481, 484, 486, 498, 501, 503, 505 | 9.35162E-05 |
| 460 | 9.35162E-05 |
| 440, 445, 446, 450, 460, 477, 478, 481, 484, 486, 498, 501, 505 | 9.35162E-05 |
| 501 | 9.35162E-05 |
| 440, 445, 446, 450, 452, 455, 456, 460, 477, 478, 481, 484, 498, 501, 505 | 9.35162E-05 |
| 440, 445, 446, 450, 452, 455, 456, 460, 477, 478, 481, 486, 498, 501, 505 | 9.35162E-05 |
| 440, 445, 446, 477, 478, 486, 498, 501, 505 | 8.31255E-05 |
| 440, 445, 446, 450, 452, 455, 460, 470, 477, 478, 481, 484, 486, 498, 501, 505 | 8.31255E-05 |
| 440, 460, 477, 478, 481, 484, 486, 498, 501, 505 | 8.31255E-05 |
| 440, 445, 446, 450, 452, 455, 460, 477, 478, 481, 484, 485, 486, 498, 501, 505 | 8.31255E-05 |
| 440, 445, 446, 450, 452, 455, 456, 460, 477, 478, 481, 484, 486, 498, 500, 501, 505 | 8.31255E-05 |
| 450, 477, 478, 481, 484, 486, 498, 501, 505 | 7.27348E-05 |
| 440, 455, 460, 477, 478, 481, 484, 486, 498, 501, 505 | 7.27348E-05 |
| 498 | 7.27348E-05 |
| 440, 445, 446, 450, 452, 455, 477, 478, 498, 501, 505 | 7.27348E-05 |
| 440 | 7.27348E-05 |
| 440, 445, 446, 450, 452, 455, 458, 460, 477, 478, 481, 484, 486, 498, 501, 505 | 7.27348E-05 |
| 440, 460, 498, 505 | 7.27348E-05 |
| 455, 460, 477, 478, 498, 501, 505 | 6.23441E-05 |
| 440, 445, 446, 450, 452, 455, 498, 501, 505 | 6.23441E-05 |
| 455, 460, 477, 478, 481, 484, 486, 498, 501 | 6.23441E-05 |
| 455, 460, 477, 478, 481, 484, 486 | 6.23441E-05 |
| 440, 445, 446, 450, 452, 455, 460, 462, 477, 478, 481, 484, 486, 498, 501, 505 | 6.23441E-05 |
| 460, 477, 478 | 6.23441E-05 |
| 440, 445, 446, 450, 452, 455, 460, 477, 478, 481, 484, 486, 501, 505 | 6.23441E-05 |
| 455, 498, 501, 505 | 6.23441E-05 |
| 440, 446, 450, 452, 455, 456, 460, 477, 478, 481, 484, 486, 498, 501, 505 | 6.23441E-05 |
| 440, 445, 446, 450, 452, 455, 456, 460, 477, 478, 482, 483, 484, 486, 498, 501, 505 | 6.23441E-05 |
| 440, 445, 446, 450, 455, 460, 477, 478, 486, 498, 501, 505 | 5.19534E-05 |
| 460, 477, 478, 481 | 5.19534E-05 |
| 440, 445, 477, 478, 481, 484, 486, 498, 501, 505 | 5.19534E-05 |
| 440, 445, 446, 450, 452, 455, 460, 477, 478, 483, 484, 486, 498, 501, 505 | 5.19534E-05 |
| 440, 445, 446, 450, 452, 477, 478, 481, 484, 486, 498, 501, 505 | 5.19534E-05 |
| 440, 445, 446, 452, 455, 460, 477, 478, 481, 484, 486, 498, 501, 505 | 5.19534E-05 |
| 450, 455, 460, 477, 478, 481, 484, 486, 498, 501, 505 | 5.19534E-05 |
| 440, 445, 446, 460, 477, 478, 481, 484, 486, 498, 501, 505 | 5.19534E-05 |
| 440, 450, 460, 477, 478, 481, 484, 486, 498, 501, 505 | 5.19534E-05 |
| 477, 478, 481 | 5.19534E-05 |
| 440, 460, 505 | 5.19534E-05 |
| 445, 446, 450, 452, 455, 456, 460, 477, 478, 481, 484, 486, 498, 501, 505 | 5.19534E-05 |
| 440, 445, 446, 450, 452, 455, 456, 459, 460, 477, 478, 481, 484, 486, 498, 501, 505 | 5.19534E-05 |
| 440, 445, 446, 460, 477, 478, 486, 498, 501, 505 | 4.15628E-05 |
| 445, 446, 450, 452, 455, 460, 477, 478, 481, 484, 486 | 4.15628E-05 |
| 450, 455, 498, 501, 505 | 4.15628E-05 |
| 440, 445, 446, 450, 452, 455 | 4.15628E-05 |
| 477, 478, 481, 498, 501, 505 | 4.15628E-05 |
| 440, 445, 446, 450, 452, 455, 460, 477, 478, 481, 484, 486, 487, 498, 501, 505 | 4.15628E-05 |
| 460, 477, 478, 498, 501, 505 | 4.15628E-05 |
| 477, 478, 481, 484, 486, 498, 501 | 4.15628E-05 |
| 440, 450, 455, 460, 477, 478, 481, 484, 486, 498, 501, 505 | 4.15628E-05 |
| 440, 445, 446, 450, 452, 498, 501, 505 | 4.15628E-05 |
| 440, 445, 446, 450, 455, 460, 477, 478, 484, 486, 498, 501, 505 | 4.15628E-05 |
| 440, 445, 446, 450, 452, 455, 460, 477, 478, 481, 484, 486, 494, 498, 501, 505 | 4.15628E-05 |
| 440, 450, 455, 460, 477, 478, 498, 501, 505 | 4.15628E-05 |
| 450, 452, 455, 460, 477, 478, 498, 501, 505 | 4.15628E-05 |
| 440, 445, 446, 450, 452, 455, 460, 477, 498, 501, 505 | 4.15628E-05 |
| 440, 445, 446, 450, 452, 456, 460, 477, 478, 481, 484, 486, 498, 501, 505 | 4.15628E-05 |
| 440, 445, 446, 450, 452, 455, 456, 460, 477, 478, 481, 498, 501, 505 | 4.15628E-05 |
| 440, 445, 446, 450, 452, 455, 460, 477, 478, 484, 498, 501, 505 | 4.15628E-05 |
| 440, 450, 452, 455, 456, 460, 477, 478, 481, 484, 486, 498, 501, 505 | 4.15628E-05 |
| 440, 445, 446, 450, 452, 455, 456, 460, 477, 478, 484, 486, 498, 501, 505 | 4.15628E-05 |
| 440, 445, 446, 450, 460, 477, 478, 486, 498, 501, 505 | 3.11721E-05 |
| 440, 445, 460, 477, 478, 481, 484, 486, 498, 501, 505 | 3.11721E-05 |
| 440, 445, 446, 456, 460, 477, 478, 484, 486, 490, 498, 501, 505 | 3.11721E-05 |
| 440, 445, 446, 452, 460, 477, 478, 484, 486, 498, 501, 505 | 3.11721E-05 |
| 440, 445, 446, 452, 456, 460, 477, 478, 484, 486, 490, 498, 501, 505 | 3.11721E-05 |
| 440, 460, 477, 478, 486, 498, 501, 505 | 3.11721E-05 |
| 455, 460, 498, 501, 505 | 3.11721E-05 |
| 440, 445, 446, 450, 452, 455, 459, 460, 477, 478, 481, 484, 486, 498, 501, 505 | 3.11721E-05 |
| 440, 445, 446, 450, 452, 498, 501 | 3.11721E-05 |
| 440, 445, 446, 450, 452, 455, 456, 460, 477, 478, 484, 486, 490, 498, 501, 505 | 3.11721E-05 |
| 455 | 3.11721E-05 |
| 440, 450, 452, 455, 460, 477, 478 | 3.11721E-05 |
| 450, 452, 455, 460, 477, 478, 481, 484, 486 | 3.11721E-05 |
| 440, 445, 446, 450, 477, 478, 481, 484, 486, 498, 501, 505 | 3.11721E-05 |
| 477, 498, 501, 505 | 3.11721E-05 |
| 440, 445, 446, 450, 452, 455, 460, 474, 477, 478, 481, 484, 486, 498, 501, 505 | 3.11721E-05 |
| 440, 450, 477, 478, 481, 484, 486, 498, 501, 505 | 3.11721E-05 |
| 450, 452, 455, 460, 477, 478, 481, 484, 486, 498, 501 | 3.11721E-05 |
| 440, 443, 445, 446, 450, 452, 455, 460, 477, 478, 481, 484, 486, 498, 501, 505 | 3.11721E-05 |
| 440, 450, 452, 455, 460, 477, 478, 481, 484, 486, 498, 501 | 3.11721E-05 |
| 450, 452, 455, 460, 477, 478 | 3.11721E-05 |
| 440, 445, 446, 450, 452, 455, 456, 460, 477, 478, 481, 483, 484, 486, 498, 501, 505 | 3.11721E-05 |
| 450, 455, 460, 477, 478, 498, 501, 505 | 3.11721E-05 |
| 440, 445, 446, 450, 452, 477, 478, 498, 501, 505 | 3.11721E-05 |
| 440, 445, 446, 450, 452, 477, 478, 486, 498, 501, 505 | 3.11721E-05 |
| 440, 445, 446, 450, 452, 453, 455, 460, 477, 478, 481, 484, 486, 498, 501, 505 | 3.11721E-05 |
| 440, 445, 446, 450, 452, 455, 456, 460, 476, 477, 478, 481, 484, 486, 498, 501, 505 | 3.11721E-05 |
| 440, 445, 446, 450, 452, 455, 456, 460, 477, 478, 481, 484, 485, 486, 498, 501, 505 | 3.11721E-05 |
| 440, 445, 446, 455, 456, 460, 477, 478, 484, 486, 490, 498, 501, 505 | 2.07814E-05 |
| 455, 460, 477, 478 | 2.07814E-05 |
| 440, 445, 446, 450, 452, 455, 460, 505 | 2.07814E-05 |
| 440, 455, 460, 477, 478, 498, 501, 505 | 2.07814E-05 |
| 440, 445, 446, 455, 460, 477, 478, 484, 486, 490, 498, 501, 505 | 2.07814E-05 |
| 440, 445, 446, 455, 456, 460, 477, 478, 481, 484, 486, 498, 501, 505 | 2.07814E-05 |
| 450, 452, 455, 477, 478, 481, 484, 486, 498, 501, 505 | 2.07814E-05 |
| 440, 445, 446, 450, 455, 460, 477, 478, 481, 484, 486, 498, 501 | 2.07814E-05 |
| 477, 478, 484, 486, 498, 501, 505 | 2.07814E-05 |
| 440, 445, 446, 450, 477, 478, 486, 498, 501, 505 | 2.07814E-05 |
| 481, 484, 486, 498, 501, 505 | 2.07814E-05 |
| 440, 445, 446, 460, 477, 478, 484, 486, 498, 501, 505 | 2.07814E-05 |
| 440, 445, 446, 450, 452, 460, 477, 478, 486, 498, 501, 505 | 2.07814E-05 |
| 486, 498, 501, 505 | 2.07814E-05 |
| 440, 445, 446, 450, 452, 455, 460, 477, 478, 498, 501 | 2.07814E-05 |
| 450, 452 | 2.07814E-05 |
| 440, 445, 446, 450, 452, 455, 460, 477, 478, 481, 484, 486, 502, 503 | 2.07814E-05 |
| 460, 498, 501, 505 | 2.07814E-05 |
| 460, 477, 478, 481, 484, 486, 498, 501 | 2.07814E-05 |
| 440, 445, 446, 452, 460, 477, 478, 486, 498, 501, 505 | 2.07814E-05 |
| 440, 450, 455, 460, 477, 478, 481, 484, 486, 498 | 2.07814E-05 |
| 440, 445, 446, 450, 452, 455, 460, 477, 478, 501, 505 | 2.07814E-05 |
| 450, 452, 455, 460, 477, 478, 481 | 2.07814E-05 |
| 455, 477, 478, 481, 484, 486, 498, 501, 505 | 2.07814E-05 |
| 440, 450, 452, 460, 477, 478 | 2.07814E-05 |
| 450, 460, 477, 478 | 2.07814E-05 |
| 445, 446, 450, 452, 455, 477, 478, 481, 484, 486, 498, 501, 505 | 2.07814E-05 |
| 440, 445, 446, 450, 452, 455, 460, 477, 478, 481, 484, 486, 505 | 2.07814E-05 |
| 450, 460, 477, 478, 481, 484, 486, 498, 501, 505 | 2.07814E-05 |
| 440, 445, 450, 452, 455, 460, 477, 478, 481, 484, 486, 498, 501, 505 | 2.07814E-05 |
| 450, 452, 477, 478, 481, 484, 486, 498, 501, 505 | 2.07814E-05 |
| 440, 446, 450, 452, 455, 460, 477, 478, 498, 501, 505 | 2.07814E-05 |
| 445, 446, 450, 452, 455, 460, 477, 478, 481, 498, 501, 505 | 2.07814E-05 |
| 477, 478, 498, 501 | 2.07814E-05 |
| 440, 445, 446, 450, 452, 455, 460, 477, 478, 481, 484, 486, 501 | 2.07814E-05 |
| 440, 498, 501, 505 | 2.07814E-05 |
| 440, 445, 446, 450, 452, 455, 460, 486, 498, 501, 505 | 2.07814E-05 |
| 455, 460 | 2.07814E-05 |
| 455, 460, 477, 478, 481, 498, 501, 505 | 2.07814E-05 |
| 440, 445, 446, 450, 452, 455, 460, 477, 478, 481, 485, 498, 501, 505 | 2.07814E-05 |
| 460, 498 | 2.07814E-05 |
| 440, 450, 455, 477, 478, 481, 484, 486, 498, 501, 505 | 2.07814E-05 |
| 477, 478, 481, 486, 498, 501, 505 | 2.07814E-05 |
| 477, 478, 501 | 2.07814E-05 |
| 440, 445, 446, 450, 452, 455, 460, 477, 478, 481, 484, 486, 498, 501, 505, 508 | 2.07814E-05 |
| 440, 450, 452, 455, 477, 478, 481, 484, 486, 498, 501, 505 | 2.07814E-05 |
| 440, 445, 446, 450, 452, 460, 477, 478, 481, 484, 498, 501, 505 | 2.07814E-05 |
| 440, 445, 446, 450, 452, 455, 477, 478, 486, 498, 501, 505 | 2.07814E-05 |
| 445, 446, 450, 452, 498, 501, 505 | 2.07814E-05 |
| 440, 445, 446, 450, 452, 477, 478, 484, 486, 498, 501, 505 | 2.07814E-05 |
| 450, 452, 455, 460, 498, 501, 505 | 2.07814E-05 |
| 440, 445, 446, 450, 452, 455, 460, 477, 478, 481, 484, 486, 498, 501, 504, 505 | 2.07814E-05 |
| 440, 477, 478, 498, 501, 505 | 2.07814E-05 |
| 477, 478, 481, 484, 498, 501, 505 | 2.07814E-05 |
| 440, 445, 446, 450, 452, 455, 460, 477, 478, 481, 484, 486, 493, 498, 501, 505 | 2.07814E-05 |
| 450, 452, 455, 456, 460, 477, 478, 498, 501, 505 | 2.07814E-05 |
| 440, 445, 446, 450, 452, 455, 456, 460, 477, 478, 482, 484, 486, 498, 501, 505 | 2.07814E-05 |
| 455, 456, 460 | 2.07814E-05 |
| 455, 456, 460, 477, 478, 481, 484, 486, 498, 501, 505 | 2.07814E-05 |
| 440, 445, 446, 455, 456, 460, 477, 478, 481, 498, 501, 505 | 2.07814E-05 |
| 440, 445, 446, 450, 452, 453, 455, 456, 460, 477, 478, 481, 484, 486, 498, 501, 505 | 2.07814E-05 |
| 440, 445, 446, 450, 452, 455, 456, 498, 501, 505 | 2.07814E-05 |
| 440, 445, 446, 450, 452, 455, 456, 460, 468, 477, 478, 481, 484, 486, 498, 501, 505 | 2.07814E-05 |
| 450, 452, 455, 456, 498, 501, 505 | 2.07814E-05 |
| 440, 445, 446, 450, 452, 455, 456, 460, 477, 498, 501, 505 | 2.07814E-05 |
| 440, 445, 446, 450, 455, 456, 460, 477, 478, 481, 484, 486, 498, 501, 505 | 2.07814E-05 |
| 456, 460, 477, 478, 481, 484, 486, 498, 501, 505 | 2.07814E-05 |
| 440, 445, 446, 450, 452, 455, 456, 460, 477, 478, 479, 481, 484, 486, 498, 501, 505 | 2.07814E-05 |
| 440, 445, 446, 450, 452, 460, 477, 478, 498, 501, 505 | 1.03907E-05 |
| 440, 445, 446, 456, 460, 484, 486, 490, 498, 501, 505 | 1.03907E-05 |
| 440, 452, 460, 477, 478, 481, 484, 486 | 1.03907E-05 |
| 440, 445, 446, 450, 452, 455, 460, 477, 478, 481, 484, 501, 505 | 1.03907E-05 |
| 440, 445, 446, 477, 486, 498, 501, 505 | 1.03907E-05 |
| 440, 445, 446, 450, 452, 455, 460, 498, 501 | 1.03907E-05 |
| 440, 445, 477 | 1.03907E-05 |
| 440, 445, 446, 460, 477, 478, 501, 505 | 1.03907E-05 |
| 440, 450, 460, 477, 478, 486, 498, 501, 505 | 1.03907E-05 |
| 445, 446, 450, 452, 455, 460, 477, 478, 481, 484, 486, 498 | 1.03907E-05 |
| 440, 445, 446, 477, 478, 498, 501, 505 | 1.03907E-05 |
| 440, 450, 452, 455, 460, 477, 478, 481, 484, 486 | 1.03907E-05 |
| 440, 445, 446, 477, 498, 501, 505 | 1.03907E-05 |
| 455, 456, 475, 477, 478, 484, 486, 490 | 1.03907E-05 |
| 440, 441, 445, 446, 450, 455, 460, 477, 478, 481, 484, 486, 498, 501, 505 | 1.03907E-05 |
| 440, 445, 446, 450, 452, 455, 460, 475, 477, 478, 498, 501, 505 | 1.03907E-05 |
| 440, 445, 446, 450, 452, 455, 477, 498, 501, 505 | 1.03907E-05 |
| 450, 455, 477, 478, 481, 484, 486, 498, 501, 505 | 1.03907E-05 |
| 440, 445, 446, 452, 477, 478, 481, 484, 486, 498, 501, 505 | 1.03907E-05 |
| 440, 450, 455, 498, 501, 505 | 1.03907E-05 |
| 440, 445, 446, 452, 455, 460, 477, 478, 484, 486, 498, 501, 505 | 1.03907E-05 |
| 440, 445, 446, 450, 477, 486, 498, 501, 505 | 1.03907E-05 |
| 452, 455, 460, 477, 478, 481, 484, 486 | 1.03907E-05 |
| 440, 445, 446, 452, 455, 477, 478, 481, 484, 486, 498, 501, 505 | 1.03907E-05 |
| 440, 455, 460, 477, 478, 481, 486, 498, 501, 505 | 1.03907E-05 |
| 450, 452, 455, 498, 501 | 1.03907E-05 |
| 440, 445, 446, 450, 452, 455, 475, 477, 478, 481, 484, 486 | 1.03907E-05 |
| 440, 452, 455, 460, 477, 478, 481, 484, 486 | 1.03907E-05 |
| 440, 445, 446, 450, 455, 460, 477, 478, 481, 484, 486 | 1.03907E-05 |
| 440, 445, 446, 450, 455, 477, 478, 486, 498, 501, 505 | 1.03907E-05 |
| 440, 445, 446, 460, 477, 478, 484, 486, 490, 498, 501, 505 | 1.03907E-05 |
| 450, 455, 460, 477, 478 | 1.03907E-05 |
| 440, 445, 446, 450, 455, 460, 477, 478, 481, 484, 486, 501, 505 | 1.03907E-05 |
| 440, 446, 455, 460, 477, 478, 481, 484, 486, 498, 501 | 1.03907E-05 |
| 476, 477, 478, 481, 484, 486, 498, 501, 505 | 1.03907E-05 |
| 440, 445, 446, 450, 452, 455, 476, 477, 478, 481, 484, 486, 498, 501, 505 | 1.03907E-05 |
| 440, 445, 446, 456, 460 | 1.03907E-05 |
| 440, 450, 477, 478, 481, 484, 486 | 1.03907E-05 |
| 450, 455, 477, 498, 501, 505 | 1.03907E-05 |
| 440, 445, 446, 450, 452, 455, 460, 478, 484, 486, 498, 501, 505 | 1.03907E-05 |
| 440, 445, 446, 450, 452, 455, 477, 478, 481, 498, 501, 505 | 1.03907E-05 |
| 440, 445, 446, 450, 455, 460, 498, 501, 505 | 1.03907E-05 |
| 460, 477, 478, 484, 498 | 1.03907E-05 |
| 477, 505 | 1.03907E-05 |
| 440, 446, 450, 452, 455, 460, 477, 478, 481 | 1.03907E-05 |
| 440, 446, 477, 478, 486, 498, 501, 505 | 1.03907E-05 |
| 440, 445, 446, 450, 452, 455, 460, 477, 478, 479, 498, 501, 505 | 1.03907E-05 |
| 455, 460, 477, 478, 481, 498 | 1.03907E-05 |
| 440, 445, 446, 450, 452, 460, 477, 478, 481 | 1.03907E-05 |
| 484 | 1.03907E-05 |
| 477, 478, 481, 484, 486, 498, 505 | 1.03907E-05 |
| 440, 445, 446, 456, 460, 477, 478, 484, 486, 498, 501, 505 | 1.03907E-05 |
| 450, 452, 455, 460 | 1.03907E-05 |
| 440, 445, 446, 450, 452 | 1.03907E-05 |
| 446, 450, 452, 455, 460, 477, 478, 498, 501, 505 | 1.03907E-05 |
| 440, 446, 450, 452, 455, 460, 477, 478, 484, 486, 490, 498, 501, 505 | 1.03907E-05 |
| 440, 445, 446, 452, 477, 486, 498, 501, 505 | 1.03907E-05 |
| 440, 445, 446, 450, 455, 460, 477, 478, 481 | 1.03907E-05 |
| 440, 445, 446, 452, 455, 477, 478, 481, 486, 498, 501, 505 | 1.03907E-05 |
| 440, 450, 452, 455, 477, 478, 498, 501, 505 | 1.03907E-05 |
| 440, 445, 446, 452, 455, 460, 477, 478, 486, 498, 501, 505 | 1.03907E-05 |
| 477, 478, 498 | 1.03907E-05 |
| 477, 478, 481, 501 | 1.03907E-05 |
| 450, 452, 455, 460, 498, 501 | 1.03907E-05 |
| 440, 445, 446, 450, 460, 477, 478, 481, 486, 498, 501, 505 | 1.03907E-05 |
| 445, 446, 450, 452, 455, 477, 478, 498, 501, 505 | 1.03907E-05 |
| 440, 446, 460, 477, 478, 484, 486, 498, 501, 505 | 1.03907E-05 |
| 440, 445, 450, 452, 455, 498, 501, 505 | 1.03907E-05 |
| 440, 450, 452, 455, 460, 477, 478, 498, 501, 505 | 1.03907E-05 |
| 445, 446, 450, 452, 460, 477, 478, 481, 484, 486, 498, 501, 505 | 1.03907E-05 |
| 445, 446, 452, 455, 460, 477, 478, 481, 484, 486, 498, 501, 505 | 1.03907E-05 |
| 477, 478, 481, 484, 486, 501 | 1.03907E-05 |
| 440, 445, 446, 450, 460, 477, 478, 484, 486, 498, 501, 505 | 1.03907E-05 |
| 440, 446, 452, 460, 477, 478, 486, 498, 501, 505 | 1.03907E-05 |
| 440, 446, 460, 477, 478, 486, 498, 501 | 1.03907E-05 |
| 450 | 1.03907E-05 |
| 440, 445, 446, 450, 452, 455, 460, 477, 478, 482, 483, 484, 486, 498, 501 | 1.03907E-05 |
| 440, 445, 446, 452, 460, 477, 478, 484, 486, 490, 498, 501, 505 | 1.03907E-05 |
| 455, 477, 478 | 1.03907E-05 |
| 450, 498, 501, 505 | 1.03907E-05 |
| 446, 450, 452, 455, 460, 477, 478, 481, 484, 486, 498, 501 | 1.03907E-05 |
| 440, 446, 455, 460, 477, 478, 486, 498, 501, 505 | 1.03907E-05 |
| 440, 445, 450, 498, 501, 505 | 1.03907E-05 |
| 445, 446, 450, 452, 455, 460, 498, 501, 505 | 1.03907E-05 |
| 478, 481, 501, 505 | 1.03907E-05 |
| 440, 445, 452, 456, 460, 477, 478, 484, 486, 498, 501, 505 | 1.03907E-05 |
| 445, 446, 450, 452, 455, 460, 477, 478, 481, 486 | 1.03907E-05 |
| 445, 446, 450, 452, 477, 478, 481, 498, 501, 505 | 1.03907E-05 |
| 440, 445, 446, 450, 452, 455, 477, 478, 481, 486, 498, 501, 505 | 1.03907E-05 |
| 477 | 1.03907E-05 |
| 460, 477, 478, 481, 484, 486, 498 | 1.03907E-05 |
| 445, 446, 450, 452, 460, 477, 478, 481, 501, 505 | 1.03907E-05 |
| 440, 446, 450, 460, 477, 478, 486, 498, 501, 505 | 1.03907E-05 |
| 461, 477, 478, 481, 484, 486, 498, 501, 505 | 1.03907E-05 |
| 445, 446, 450, 477, 478, 481 | 1.03907E-05 |
| 440, 445, 446, 450, 452, 455, 460, 477, 478, 484, 486, 498, 505 | 1.03907E-05 |
| 440, 445, 446, 450, 455, 460, 477, 486, 498, 501, 505 | 1.03907E-05 |
| 440, 445, 446, 450, 455, 460, 477, 478, 481, 486, 498, 501, 505 | 1.03907E-05 |
| 445, 446, 455, 460, 477, 478, 481 | 1.03907E-05 |
| 440, 450, 452, 455, 460, 477, 498, 501, 505 | 1.03907E-05 |
| 440, 445, 446, 450, 452, 455, 477, 486, 498, 501, 505 | 1.03907E-05 |
| 440, 450, 452, 455, 460, 477, 478, 481 | 1.03907E-05 |
| 440, 445, 446, 450, 452, 455, 460, 477, 486, 498, 501, 505 | 1.03907E-05 |
| 440, 445, 446, 450, 455, 477, 486, 498, 501, 505 | 1.03907E-05 |
| 450, 452, 455, 460, 477, 478, 481, 484, 486, 498 | 1.03907E-05 |
| 440, 445, 446, 450, 452, 477, 478, 498, 501 | 1.03907E-05 |
| 460, 505 | 1.03907E-05 |
| 445, 450, 452, 455, 460, 477, 478 | 1.03907E-05 |
| 455, 498 | 1.03907E-05 |
| 440, 445, 446, 450, 452, 455, 460, 477, 478, 486, 498, 501 | 1.03907E-05 |
| 440, 460, 498, 501, 505 | 1.03907E-05 |
| 440, 445, 446, 450, 455, 477, 478, 498, 501, 505 | 1.03907E-05 |
| 440, 445, 446, 450, 452, 455, 460, 477, 478, 481, 484, 498, 501 | 1.03907E-05 |
| 450, 460, 498, 501 | 1.03907E-05 |
| 455, 460, 477, 478, 481, 486, 501 | 1.03907E-05 |
| 440, 450, 455, 460, 477, 478, 481, 498, 501, 505 | 1.03907E-05 |
| 440, 452, 455, 460, 477, 478, 481, 484, 486, 498, 501 | 1.03907E-05 |
| 440, 445, 446, 452, 455, 460, 477, 478, 481, 498, 501, 505 | 1.03907E-05 |
| 440, 445, 446, 450, 455, 477, 478, 481, 484, 486, 498, 501, 505 | 1.03907E-05 |
| 445, 446, 460, 477, 478, 481, 484, 486, 498, 501 | 1.03907E-05 |
| 440, 445, 446, 450, 452, 455, 460, 477, 478, 481, 484, 486, 498, 505 | 1.03907E-05 |
| 440, 441, 445, 446, 450, 452, 455, 460, 477, 478, 481 | 1.03907E-05 |
| 450, 452, 455, 460, 477, 478, 501 | 1.03907E-05 |
| 450, 452, 460, 477, 478, 498, 501, 505 | 1.03907E-05 |
| 440, 450, 452, 455, 460, 477, 478, 481, 498, 501, 505 | 1.03907E-05 |
| 440, 445, 446, 450, 452, 455, 477, 478, 498, 501 | 1.03907E-05 |
| 440, 445, 446, 450, 452, 455, 460, 475, 477, 478, 481 | 1.03907E-05 |
| 440, 445, 446, 450, 452, 455, 456, 460, 475, 477, 478, 481, 484, 498, 501, 505 | 1.03907E-05 |
| 440, 445, 446, 450, 452, 455, 460, 478, 481, 484, 486, 498, 501, 505 | 1.03907E-05 |
| 440, 445, 446, 450, 452, 455, 460, 477, 478, 481, 498, 501 | 1.03907E-05 |
| 440, 445, 446, 450, 452, 455, 460, 477, 478, 481, 484, 486, 498, 499, 501 | 1.03907E-05 |
| 440, 445, 446, 450, 452, 455, 460, 477, 478, 482, 484, 486, 498, 501 | 1.03907E-05 |
| 460, 501 | 1.03907E-05 |
| 455, 460, 505 | 1.03907E-05 |
| 440, 455, 456, 460, 477, 478, 481, 484, 486, 498, 501 | 1.03907E-05 |
| 440, 486, 498, 501, 505 | 1.03907E-05 |
| 445, 446, 450, 452, 455, 460, 477, 478, 481, 501 | 1.03907E-05 |
| 440, 452, 455, 460, 477, 478, 481, 484, 486, 498, 501, 505 | 1.03907E-05 |
| 460, 477, 478, 481, 498, 501, 505 | 1.03907E-05 |
| 440, 445, 446, 450, 452, 455, 460, 461, 477, 478, 481, 484, 486, 498, 501, 505 | 1.03907E-05 |
| 445, 446, 450, 455, 460, 477, 478, 481, 484, 486, 498, 501, 505 | 1.03907E-05 |
| 498, 505 | 1.03907E-05 |
| 440, 445, 446, 450, 452, 455, 456, 460, 486, 498, 501, 505 | 1.03907E-05 |
| 440, 445, 446, 450, 452, 455, 477, 478, 484, 486, 498, 501, 505 | 1.03907E-05 |
| 440, 445, 446, 448, 450, 452, 455, 460, 477, 478, 481, 484, 486, 498, 501, 505 | 1.03907E-05 |
| 477, 478, 481, 484 | 1.03907E-05 |
| 440, 445, 446, 455, 477, 478, 481, 484, 486, 498, 501, 505 | 1.03907E-05 |
| 440, 445, 446, 455, 460, 477, 478, 486, 498, 501, 505 | 1.03907E-05 |
| 440, 445, 446, 450, 452, 455, 460, 477, 478, 481, 484, 486, 498, 499, 501, 505 | 1.03907E-05 |
| 440, 450, 452, 475, 477, 478, 481, 484, 486, 498, 501, 505 | 1.03907E-05 |
| 440, 450, 452, 460, 498, 501, 505 | 1.03907E-05 |
| 440, 445, 446, 450, 452, 460, 477, 478, 481, 484, 486, 498, 501 | 1.03907E-05 |
| 440, 445, 446, 450, 452, 455, 477, 478, 481, 484, 486 | 1.03907E-05 |
| 450, 452, 455 | 1.03907E-05 |
| 450, 452, 455, 460, 477, 478, 498, 501 | 1.03907E-05 |
| 450, 460, 477, 478, 498, 501, 505 | 1.03907E-05 |
| 440, 445, 446, 450, 452, 455, 460, 477, 478, 479, 484, 486, 490, 498, 501, 505 | 1.03907E-05 |
| 440, 450, 452, 477, 478, 481, 484, 486, 498, 501, 505 | 1.03907E-05 |
| 440, 450, 452, 455, 456, 477, 478, 481, 484, 486, 498, 501, 505 | 1.03907E-05 |
| 450, 455, 460 | 1.03907E-05 |
| 440, 445, 446, 450, 452, 455, 460, 477, 478, 481, 483, 484, 485, 486, 498, 501, 505 | 1.03907E-05 |
| 440, 445, 446, 450, 477, 478, 481, 484, 486 | 1.03907E-05 |
| 445, 446, 450, 455, 460, 477, 478, 481, 484, 486, 498, 501 | 1.03907E-05 |
| 450, 455 | 1.03907E-05 |
| 440, 445, 446, 450, 452, 455, 460, 477, 478, 505 | 1.03907E-05 |
| 477, 478, 479, 481, 484, 486, 498, 501, 505 | 1.03907E-05 |
| 440, 460, 477, 478 | 1.03907E-05 |
| 440, 445, 446, 450, 460 | 1.03907E-05 |
| 440, 445, 446, 450, 452, 455, 460, 477, 478, 481, 484, 493, 498, 501, 505 | 1.03907E-05 |
| 440, 446, 450, 452, 455, 460, 477, 478, 481, 484, 501, 505 | 1.03907E-05 |
| 460, 477 | 1.03907E-05 |
| 440, 445, 446, 450, 452, 477, 478, 481, 486, 498, 501, 505 | 1.03907E-05 |
| 477, 478, 501, 505 | 1.03907E-05 |
| 445, 446, 450, 452 | 1.03907E-05 |
| 440, 445, 446, 450, 452, 455, 460, 477, 478, 498 | 1.03907E-05 |
| 446, 450, 452, 455, 460, 477, 478, 481, 484, 501, 505 | 1.03907E-05 |
| 450, 452, 455, 460, 486, 498, 501, 505 | 1.03907E-05 |
| 440, 445, 446, 450, 452, 455, 456, 460, 477, 478, 481, 484, 486, 498, 501, 503, 505 | 1.03907E-05 |
| 440, 445, 446, 450, 460, 477, 478, 481, 484, 486, 498, 501 | 1.03907E-05 |
| 445, 446, 450, 452, 455, 460, 477, 478, 481, 484, 486, 501, 505 | 1.03907E-05 |
| 440, 450, 455, 460, 477, 478, 481, 484, 486, 498, 501 | 1.03907E-05 |
| 440, 445, 446, 450, 452, 455, 456, 460, 477, 478, 481, 484, 486, 501, 505 | 1.03907E-05 |
| 445, 446, 455, 498, 501, 505 | 1.03907E-05 |
| 446, 450, 452, 455, 460, 477, 478, 481, 484, 486, 498, 501, 505 | 1.03907E-05 |
| 440, 446, 450, 452, 455, 477, 478, 484, 486, 498, 501, 505 | 1.03907E-05 |
| 440, 446, 450, 452, 455, 460, 477, 478, 481, 484, 498, 501, 505 | 1.03907E-05 |
| 440, 445, 446, 450, 452, 455, 456, 460, 461, 477, 478, 481, 484, 486, 498, 501, 505 | 1.03907E-05 |
| 445, 446, 477, 478, 505 | 1.03907E-05 |
| 440, 445, 446, 450, 452, 455, 456, 460, 471, 477, 478, 481, 484, 486, 498, 501, 505 | 1.03907E-05 |
| 440, 445, 446, 452, 455, 460, 477, 478, 481, 484, 486, 498, 501 | 1.03907E-05 |
| 440, 445, 446, 450, 452, 455, 456, 460, 477, 478, 481, 485, 498, 501, 505 | 1.03907E-05 |
| 452, 455, 460, 477, 478 | 1.03907E-05 |
| 440, 445, 446, 450, 452, 455, 481, 484, 486, 498, 501, 505 | 1.03907E-05 |
| 450, 452, 455, 498, 501, 505 | 1.03907E-05 |
| 440, 445, 446, 450, 452, 455, 456, 460, 477, 478, 481, 484, 486, 498, 505 | 1.03907E-05 |
| 440, 450, 477, 478, 498, 501, 505 | 1.03907E-05 |
| 440, 455, 477, 478, 498, 501, 505 | 1.03907E-05 |
| 503, 505 | 1.03907E-05 |
| 446, 450, 452, 455, 477, 478, 481, 486, 498, 501, 505 | 1.03907E-05 |
| 445, 446, 450, 452, 455, 460, 477, 478, 498, 501, 505 | 1.03907E-05 |
| 440, 445, 446, 450, 452, 455, 460, 463, 477, 478, 481, 484, 486, 498, 501, 505 | 1.03907E-05 |
| 440, 445, 446, 450, 452, 455, 456, 460, 477, 478, 481, 486 | 1.03907E-05 |
| 440, 445, 446, 450, 452, 455, 456, 460, 477, 478, 481, 484, 486, 498, 499, 501, 505 | 1.03907E-05 |
| 452, 455, 456, 460, 477, 478, 481, 484, 486 | 1.03907E-05 |
| 455, 456, 460, 477, 478, 481, 484, 486, 498, 501 | 1.03907E-05 |
| 450, 452, 455, 456, 460, 477, 478, 481, 484, 486 | 1.03907E-05 |
| 440, 450, 452, 455, 456, 498, 501, 505 | 1.03907E-05 |
| 440, 460, 477, 478, 481, 484, 486, 498, 501 | 1.03907E-05 |
| 440, 446, 450, 455, 460, 477, 478, 481, 484, 486, 498, 501, 505 | 1.03907E-05 |
| 440, 445, 446, 450, 452, 455, 456, 477, 478, 481, 484, 486, 498, 501, 505 | 1.03907E-05 |
| 440, 450, 452, 455, 456, 460, 477, 478, 481, 484, 486, 498 | 1.03907E-05 |
| 440, 450, 452, 460, 477, 478, 498, 501, 505 | 1.03907E-05 |
| 440, 450, 455, 456, 460, 477, 478, 481, 484, 486, 498, 501, 503, 505 | 1.03907E-05 |
| 440, 445, 446, 450, 452, 455, 456, 477, 478, 486, 498, 501, 505 | 1.03907E-05 |
| 440, 441, 445, 446, 450, 452, 460, 477, 478, 481, 484, 486, 498, 501, 505 | 1.03907E-05 |
| 455, 456, 460, 477, 481, 484, 486, 498, 501, 505 | 1.03907E-05 |
| 440, 450, 455, 456, 460, 477, 478, 481, 484, 486, 498, 501, 505 | 1.03907E-05 |
| 440, 450, 456, 460, 477, 478 | 1.03907E-05 |
| 445, 446, 450, 452, 455, 456, 460, 477, 478, 498, 501, 505 | 1.03907E-05 |
| 460, 477, 481, 484, 486, 498, 501, 505 | 1.03907E-05 |
| 440, 446, 450, 452, 455, 456, 460, 477, 478, 498, 501, 505 | 1.03907E-05 |
| 460, 477, 478, 481, 498 | 1.03907E-05 |
| 440, 445, 446, 450, 455, 456, 460, 498, 501, 505 | 1.03907E-05 |
| 455, 456, 460, 477, 478, 481, 484, 486 | 1.03907E-05 |
| 440, 455, 460, 477, 478, 486, 498, 501, 505 | 1.03907E-05 |
| 440, 481, 484, 486, 498, 501, 505 | 1.03907E-05 |
| 455, 477, 478, 481, 486, 498, 501, 505 | 1.03907E-05 |
| 440, 445, 446, 450, 452, 455, 456, 460, 473, 477, 478, 481, 484, 486, 498, 501, 505 | 1.03907E-05 |
| 440, 445, 446, 450, 455, 456, 460, 477, 478, 498, 501, 505 | 1.03907E-05 |
| 445, 446, 455, 456, 460, 477, 478, 481, 484, 486, 498, 501, 505 | 1.03907E-05 |
| 440, 450, 452 | 1.03907E-05 |
| 440, 445, 446, 455, 456, 460, 477, 478, 498, 501, 505 | 1.03907E-05 |
| 440, 441, 445, 446, 450, 452, 455, 456, 460, 477, 478, 481, 484, 486 | 1.03907E-05 |
| 440, 445, 446, 450, 498, 501, 505 | 1.03907E-05 |
| 450, 456, 460, 477, 478 | 1.03907E-05 |
| 440, 445, 446, 450, 452, 455, 456, 460, 498, 501, 505 | 1.03907E-05 |
| 456, 460, 477, 478, 481, 486, 498, 501, 505 | 1.03907E-05 |
| 446, 450, 452 | 1.03907E-05 |
| 445, 446, 498, 501, 505 | 1.03907E-05 |
| 440, 452, 455, 456, 460, 498, 501, 505 | 1.03907E-05 |
| 440, 456, 460, 477, 478, 481, 484, 486, 498, 501, 505 | 1.03907E-05 |
| 440, 445, 446, 450, 452, 455, 456, 460, 477, 478 | 1.03907E-05 |
| 440, 452, 455, 456, 460, 477, 478, 498, 501, 505 | 1.03907E-05 |
| 440, 445, 446, 450, 452, 455, 456, 460, 477, 478, 486, 498, 501, 505 | 1.03907E-05 |
| 460, 477, 478, 481, 486, 498, 501, 505 | 1.03907E-05 |
| 455, 456, 460, 477, 478, 481 | 1.03907E-05 |
| 440, 450, 455, 456, 460 | 1.03907E-05 |
| 450, 477, 478, 481, 498, 501, 505 | 1.03907E-05 |
| 460, 477, 498, 501, 505 | 1.03907E-05 |
| 460, 477, 478, 498, 501 | 1.03907E-05 |
| 440, 445, 446, 450, 452, 455, 456, 460, 477, 478, 481, 484, 486, 490, 498, 501, 505 | 1.03907E-05 |
| 440, 441, 445, 446, 450, 452, 455, 456, 460 | 1.03907E-05 |
| 450, 455, 456, 460, 477, 478, 481, 484, 486 | 1.03907E-05 |
| 440, 455, 456, 460, 477, 478, 481, 484, 486, 498, 501, 505 | 1.03907E-05 |
| 460, 477, 478, 486, 498, 501 | 1.03907E-05 |
| 460, 477, 478, 481, 484, 486, 505 | 1.03907E-05 |
| 440, 450, 452, 455, 456, 460, 477, 478, 481, 484, 486, 498, 501 | 1.03907E-05 |
| 440, 446, 477, 478, 484, 498, 501, 505 | 1.03907E-05 |
| 440, 445, 446, 450, 452, 455, 456, 460, 474, 477, 478, 481, 484, 486, 498, 501, 505 | 1.03907E-05 |
| 440, 445, 446, 450, 452, 455, 456, 460, 477, 478, 481, 484, 486, 498, 501, 504, 505 | 1.03907E-05 |
| 452, 455, 456, 460, 477, 478, 481, 484, 486, 498, 501, 505 | 1.03907E-05 |
| 440, 445, 450, 452, 455, 456, 460, 477, 478, 481, 484, 486, 493, 498, 501, 505 | 1.03907E-05 |
| 440, 446, 450, 452, 455, 460, 477, 478, 481, 484, 486, 501, 505 | 1.03907E-05 |
| 455, 456, 460, 477, 478, 481, 498, 501, 505 | 1.03907E-05 |
| 440, 445, 446, 450, 452, 455, 456, 460, 462, 477, 478, 481, 484, 486 | 1.03907E-05 |
| 440, 445, 446, 450, 452, 455, 456, 460, 470, 477, 478, 481, 484, 486, 498, 501, 505 | 1.03907E-05 |
| 440, 452, 455, 456, 460, 477, 478, 481, 484, 486, 498 | 1.03907E-05 |
| 440, 445, 446, 450, 452, 455, 456, 460, 477, 478, 498, 501 | 1.03907E-05 |

**Supplementary Table 5. The classification performance metrics results for each training set across different models**

| **XGBoost** | | | | |
| --- | --- | --- | --- | --- |
| Model | Accuracy | Precision | Recall | F1 Score |
| training set 1 | 0.99999 | 0.999994 | 0.999994 | 0.999994 |
| training set 2 | 0.999999 | 1 | 1 | 1 |
| training set 3 | 0.999998 | 1 | 1 | 1 |
| training set 4 | 0.999999 | 1 | 1 | 1 |
| training set 5 | 0.999957 | 0.999995 | 0.999995 | 0.999995 |
| training set 6 | 0.999977 | 0.999998 | 0.999997 | 0.999997 |
| **Random forest** | | | | |
| Model | Accuracy | Precision | Recall | F1 Score |
| training set 1 | 0.978058 | 0.732839 | 0.71293 | 0.654134 |
| training set 2 | 0.994329 | 0.93625 | 0.915855 | 0.909607 |
| training set 3 | 0.996356 | 0.953762 | 0.934814 | 0.933226 |
| training set 4 | 0.996655 | 0.945775 | 0.928999 | 0.923774 |
| training set 5 | 0.998334 | 0.997347 | 0.99833 | 0.997518 |
| training set 6 | 0.999016 | 0.986429 | 0.986609 | 0.98624 |
| **LightGBM** | | | | |
| Model | Accuracy | Precision | Recall | F1 Score |
| training set 1 | 0.978058 | 0.733422 | 0.712744 | 0.653694 |
| training set 2 | 0.994329 | 0.935541 | 0.915792 | 0.909311 |
| training set 3 | 0.996356 | 0.953297 | 0.934754 | 0.933018 |
| training set 4 | 0.996655 | 0.945386 | 0.928969 | 0.923584 |
| training set 5 | 0.998334 | 0.996671 | 0.998334 | 0.997502 |
| training set 6 | 0.999016 | 0.985981 | 0.986582 | 0.986084 |
| **GRU** | | | | |
| Model | Accuracy | Precision | Recall | F1 Score |
| training set 1 | 0.76555109 | 1 | 0.76555109 | 0.867209218 |
| training set 2 | 0.639761939 | 1 | 0.639761939 | 0.780310756 |
| training set 3 | 0.604919024 | 1 | 0.604919024 | 0.753831209 |
| training set 4 | 0.593415947 | 1 | 0.593415947 | 0.744834955 |
| training set 5 | 0.999966 | 1 | 0.999966 | 0.999983 |
| training set 6 | 0.999981 | 1 | 0.999981 | 0.999991 |

**Supplementary Table 6. The mutation frequency for each clade in the RBM region**

**Supplementary Methods. The mutation frequency for each clade in the RBM region**

**Cloning and Cell culture**

The full-length SARS-CoV-2 spike protein (Wuhan-Hu-1 strain), optimized for human codons, was supplied by Sino Biological Inc. (Beijing, China). PlatinumTM SuperFi ll PCR Master Mix (Invitrogen, Waltham, MA, USA) was used to create mutation Q493R.

Cells HEK293T and 293T-hACE2 were cultured in Dulbecco’s Modified Eagle Medium (DMEM, Welgene, South Korea) with 10% fetal bovine serum (FBS, Gibco) and 1% penicillin-streptomycin (Gibco) (37 ℃ and 5% CO2). A stable HEK293T cell line expressing human ACE2, referred to as 293T-hACE2, was created by introducing the ACE2 gene using a lentiviral gene transduction system, with hygromycin used as a selective pressure.

**viral entry measurement**

Viral supernatants were collected 48 hours post-transfection and were then standardized using the Lenti-X reverse transcription-quantitative PCR (qRT-PCR) titration kit (Cat. 631235) as per the manufacturer’s instructions.

Forty-eight hours post-infection, 293T-hACE2 cells were lysed to assess the efficiency of viral entry by comparing the luciferase activities of pseudotyped viruses containing either the wild-type or mutant S protein. A lentivirus pseudotyped with VSV-G served as a positive control. The relative luciferase activity within the cell lysates was quantified using a luciferase assay kit from Promega.s
